# A Genomic and Bioinformatics View of the Classification and Evolution of *Morganella* Species and Their Chromosomal Accessory Genetic Elements Harboring Antimicrobial Resistance Genes

**DOI:** 10.1128/spectrum.02650-21

**Published:** 2022-02-23

**Authors:** Ying Jing, Zhe Yin, Peng Wang, Jiayao Guan, Fangzhou Chen, Lingling Wang, Xinyue Li, Xiaofei Mu, Dongsheng Zhou

**Affiliations:** a State Key Laboratory of Pathogen and Biosecurity, Beijing Institute of Microbiology and Epidemiology, Beijing, China; Forschungszentrum Jülich GmbH

**Keywords:** *Morganella*, accessory genetic elements, antimicrobial resistance, genome sequencing, species classification

## Abstract

In this study, draft-genome sequencing was conducted for 60 Chinese *Morganella* isolates, and furthermore, 12 of them were fully sequenced. Then, a total of 166 global sequenced *Morganella* isolates, including the above 60, were collected to perform average nucleotide identity-based genomic classification and core single nucleotide polymorphism-based phylogenomic analysis. A genome sequence-based species classification scheme for *Morganella* was established, and accordingly, the two conventional *Morganella* species were redefined as two complexes and further divided into four and two genospecies, respectively. At least 88 acquired antimicrobial resistance genes (ARGs) were disseminated in these 166 isolates and were prevalent mostly in the isolates from hospital settings. IS*26*/IS*15DI*, IS*10* and IS*1R*, and Tn*3-*, Tn*21*-, and Tn*7*-subfamily unit transposons were frequently presented in these 166 isolates. Furthermore, a detailed sequence comparison was applied to 18 *Morganella* chromosomal accessory genetic elements (AGEs) from the fully sequenced 12 isolates, together with 5 prototype AGEs from GenBank. These 23 AGEs were divided into eight different groups belonging to composite/unit transposons, transposable prophages, integrative and mobilizable elements, and integrative and conjugative elements, and they harbored at least 52 ARGs involved in resistance to 15 categories of antimicrobials. Eleven of these 23 AGEs acquired large accessory modules, which exhibited complex mosaic structures and contained many antimicrobial resistance loci and associated ARGs. Integration of ARG-containing AGEs into *Morganella* chromosomes would contribute to the accumulation and dissemination of ARGs in *Morganella* and enhance the adaption and survival of *Morganella* under complex and diverse antimicrobial selection pressures.

**IMPORTANCE** This study presents a comprehensive genomic epidemiology analysis on global sequenced *Morganella* isolates. First, a genome sequence-based species classification scheme for *Morganella* is established with a higher resolution and accuracy than those of the conventional scheme. Second, the prevalence of accessory genetic elements (AGEs) and associated antimicrobial resistance genes (ARGs) among *Morganella* isolates is disclosed based on genome sequences. Finally, a detailed sequence comparison of eight groups of 23 AGEs (including 19 *Morganella* chromosomal AGEs) reveals that *Morganella* chromosomes have evolved to acquire diverse AGEs harboring different profiles of ARGs and that some of these AGEs harbor large accessory modules that exhibit complex mosaic structures and contain a large number of ARGs. Data presented here provide a deeper understanding of the classification and evolution of *Morganella* species and also those of ARG-containing AGEs in *Morganella* at the genomic scale.

## INTRODUCTION

*Morganella* is ubiquitously present and belongs to the *Morganellaceae* family (1). *Morganella* includes only two described species, Morganella morganii and Morganella psychrotolerans, based on DNA–DNA hybridization (2), and M. morganii is furthermore divided into two subspecies, *morganii* and *sibonii*, according to trehalose fermentation ability (3). The 16S rRNA gene nucleotide similarity between M. morganii and M. psychrotolerans isolates is 98.6% (2), which is above the threshold of 97% generally used to separate species (4), indicating that these two species cannot be steadily distinguished by 16S rRNA gene sequences.

*M. psychrotolerans* is occasionally isolated from chilled seafood and is recognized as a rainbow trout pathogen (5). M. morganii is frequently isolated from hospital settings and represents an important opportunistic pathogen (6). M. morganii is naturally resistant to penicillins, the first/second-generation cephalosporins, nitrofurantoin, tigecycline, macrolides, lincosamides, fusidic acid, polymyxins, and glycopeptides (6). M. morganii can acquire diverse accessory genetic elements (AGEs), such as unit transposons (7, 8), integrative and conjugative elements (ICEs) (9), and integrative and mobilizable elements (IMEs) (10, 11). These AGEs carry diverse antimicrobial resistance genes (ARGs), such as *bla*_KPC-2_ (8), *aadA1* (10), and *qnrD* (12), and thus greatly contribute to the dissemination of antimicrobial resistance in M. morganii. Although there are a plenty of reports on identifying AGEs and associated ARGs in M. morganii, only few of them are devoted to genetically dissecting their modular structures (7, 10).

This study presented a genomic epidemiology analysis on 166 global sequenced *Morganella* isolates, including 60 sequenced in this study. We established a genome sequence-based species classification scheme for *Morganella* to give a genomic view of *Morganella* species classification and, moreover, disclosed the prevalence of ARG-associated AGEs among *Morganella* isolates. We further performed a detailed sequence comparison of 18 *Morganella* chromosomal AGEs sequenced in this study together with 5 prototype AGEs from GenBank to provide a deeper understanding of *Morganella* AGE diversification.

## RESULTS

### Genomic classification and evolution of *Morganella* species.

**FIG 1:**
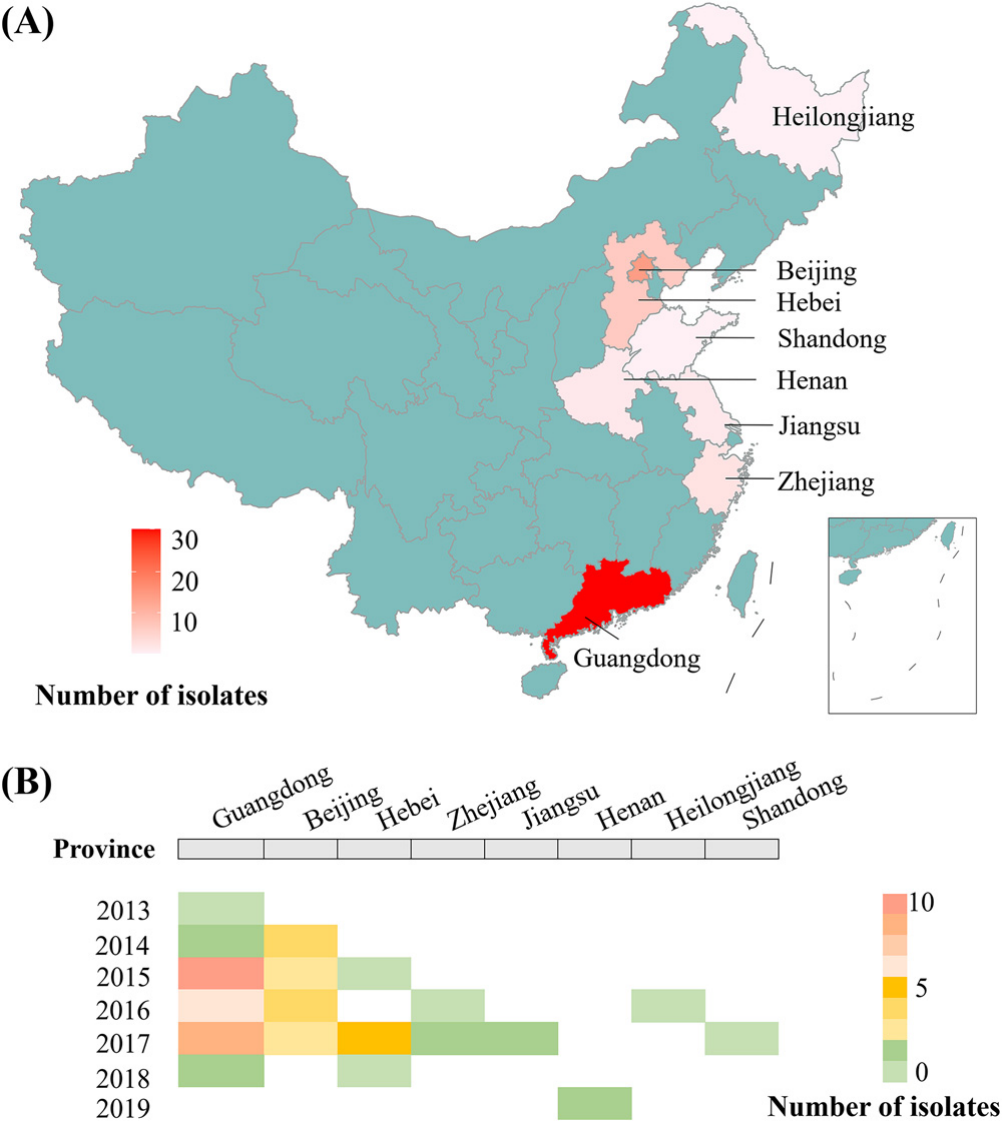
Spatial-temporal distribution of *Morganella* isolates from China. (A) Distribution of 60 *Morganella* isolates collected in this study in different provinces. (B) Distribution of 60 *Morganella* isolates in different years of different provinces.

**FIG 2:**
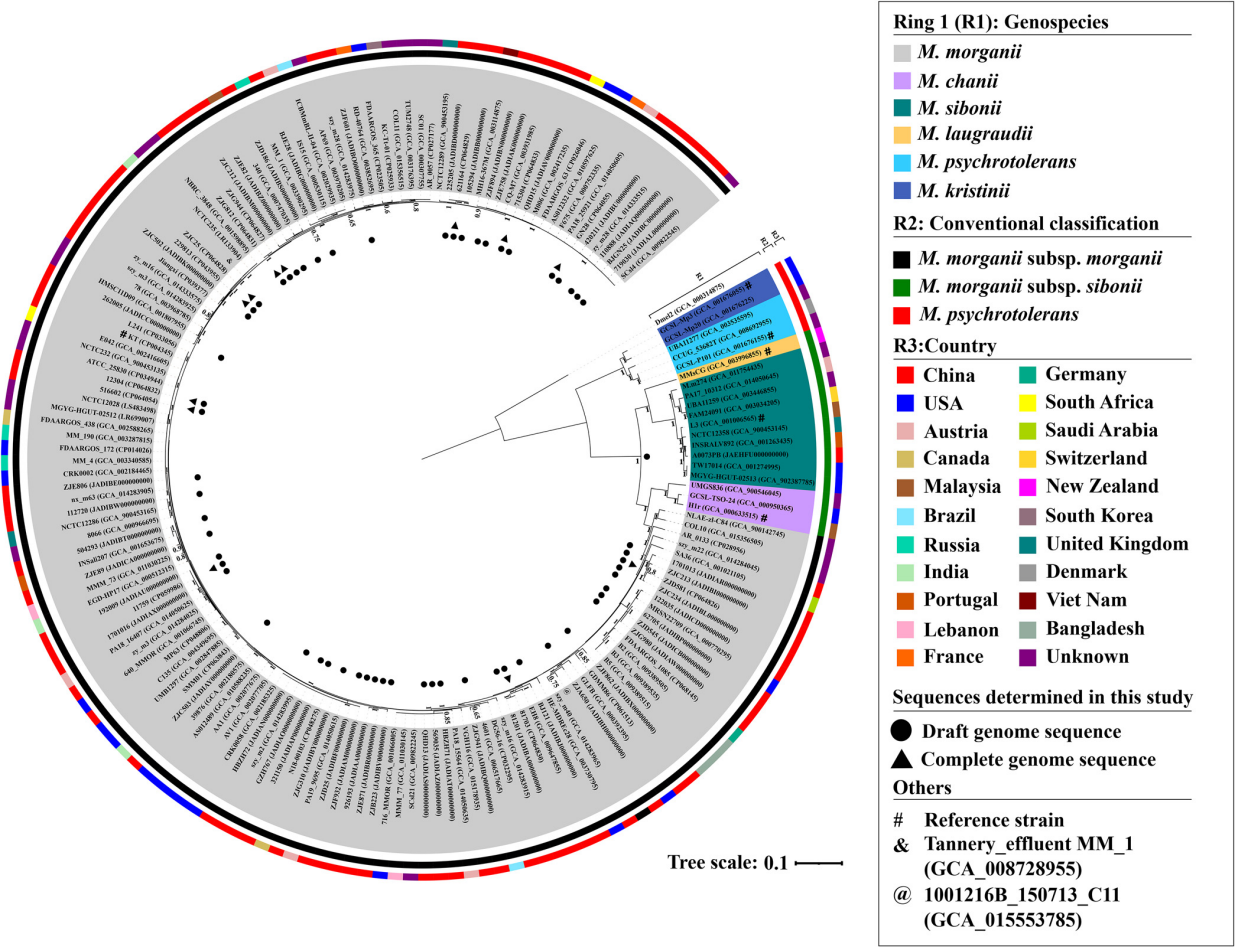
A maximum-likelihood phylogenetic tree of *Morganella* isolates. Degree of support (percentage) for each cluster of associated taxa, as determined by bootstrap analysis, is shown next to each branch. Bar corresponds to scale of sequence divergence. Providencia alcalifaciens isolate Dmel2 (accession number GCA_000314875) is used as the outgroup. For each genospecies, the isolate is designated the reference if its genome sequence is first uploaded in GenBank. Following the well-established binomial nomenclature principles (52), the genospecies name is designed by using the surname of the person submitting its reference isolate’s genome sequence to GenBank.

We determined the draft-genome sequences of 60 Chinese *Morganella* isolates (Fig. 1 and Table S1) and also the complete genome sequences of 12 of these 60 isolates (see Table S2 for quality control results). We then performed the species classification and phylogenomic analysis on a collection of 166 global sequenced *Morganella* isolates, including the above 60 together with the other 106 from GenBank (last accessed February 1 2021). Based on the conventional scheme for classifying *Morganella* species (2), 161 (96.99%) of them were assigned into M. morganii while the remaining 5 (3.01%) were assigned into *M. psychrotolerans*, indicating that the overwhelming majority of *Morganella* isolates belonged to M. morganii. Given that the absence and presence of trehalose-utilization operon *treRBP* could be used to distinguish *morganii* and *sibonii* subspecies, respectively (13), it was found here that 147 isolates were assigned into *morganii* subspecies and the remaining 14 were assigned into *sibonii* subspecies (Fig. 2 and Table S1).

To perform genomic classification and phylogeny of *Morganella*, the pairwise average nucleotide identity (ANI) values of these 166 isolates were calculated (Fig. S1 and Table S3). Based on the threshold of 95% ANI for genospecies delineation (14), a total of six genospecies could be classified and then designated M. morganii, M. chanii, M. sibonii, M. laugraudii, M. psychrotolerans, and M. kristinii, respectively. The former four genospecies were assigned into M. morganii complex, while the latter two were assigned into *M. psychrotolerans* complex. These two complexes displayed ≤84.4% ANI with each other, while the genospecies within each complex displayed ≥90.5% ANI. The 60 isolates sequenced in this study could be assigned into the two genospecies M. morganii (*n* = 59) and *M. sibonii* (*n* = 1) of the M. morganii complex.

For further phylogenomic analysis, a total of 3,538 core single nucleotide polymorphisms (SNPs) were identified from these 166 chromosome sequences. The recombination relative to point mutation (*r/m*) value was calculated to evaluate the impact of homologous recombination on sequence diversification (15). An *r*/*m* value of 2.6 was inferred at the genome level, indicating that homologous recombination introduced 2.6 times more nucleotide substitution than point mutation and, thereby, recombination events frequently occurred during genomic evolution and classification of *Morganella* (15). To avoid the influence of homologous recombination on phylogenetic reconstruction, a collection of 1,299 recombination-free SNPs was generated, and a maximum-likelihood phylogenetic tree was constructed using these recombination-free SNPs (Fig. 2). Almost all of the branches in this tree had bootstrap values of ≥70%, suggesting that this recombination-free tree could accurately reflect the evolutionary relatedness and population structure of *Morganella* (16). In this tree, the isolates from M. morganii and *M. psychrotolerans* complexes were clustered into two primary phylogroups that split earliest and emerged independently, indicating very distinct evolutionary histories of these two complexes. These two primary phylogroups could be further divided into four and two subphylogroups, respectively; as expected, they showed perfect correspondence to the above six genospecies, illustrating the consistency between ANI-based genospecies classification and phylogenomic analysis. The population (*n* = 147) of M. morganii, much larger than that of the other five genospecies, exhibited a highly clonal structure independent of geographic locations, time, and specimens of these isolates (Fig. 2 and Table S1).

### Distribution of acquired ARGs among *Morganella* isolates.

At least 88 kinds of acquired ARGs, involved in resistance to 16 different categories of antimicrobials, were identified in these 166 *Morganella* isolates (Fig. S2). All these ARGs were distributed in M. morganii complex (116/166, 67.47%), including genospecies M. morganii (103/166, 62.04%), *M. sibonii* (10/166, 6.02%), and *M. chanii* (3/166,1.81%) (Fig. S3 and Table S4). The most prevalent acquired ARGs were tetracycline-resistance genes (99/166, 59.64%), followed by aminoglycoside-resistance genes (70/166, 42.17%), sulfonamide-resistance genes (62/166, 37.35%), trimethoprim-resistance genes (56/166, 33.73%), and β-lactam-resistance genes (54/166, 32.53%) (Fig. S3 and Table S4).

**FIG 3:**
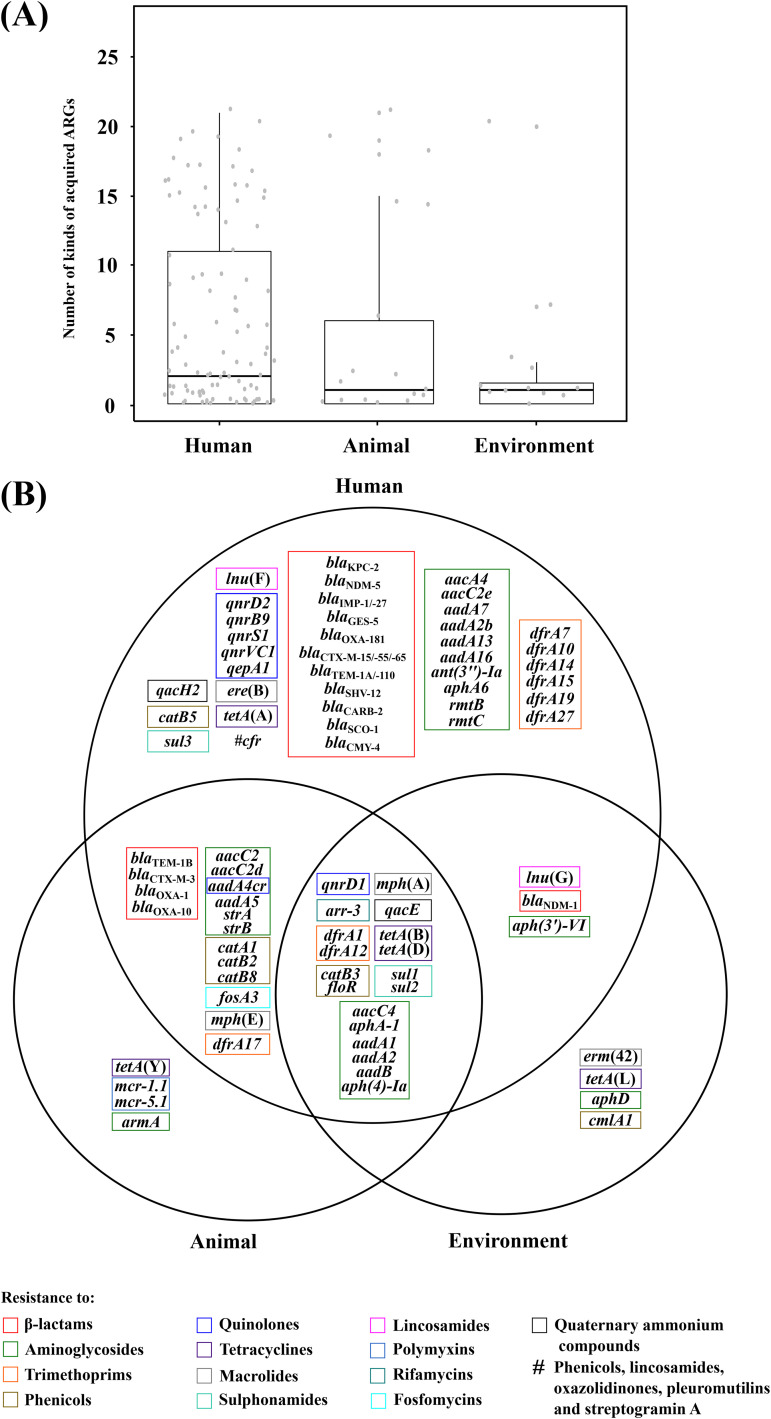
Distribution of acquired ARGs among *Morganella* isolates. (A) Boxplot displays the number of kinds of acquired ARGs in three reservoirs. (B) Venn diagram shows the distribution of different classes of acquired ARGs in three reservoirs.

These 88 acquired ARGs were further assigned into the reservoirs (humans, animals, and the environment) of the 149 isolates with source information (Table S1). *Morganella* isolates from humans contained many more acquired ARGs than those from animals and the environment (Fig. 3A), and moreover, 80 of 88 acquired ARGs (especially including aminoglycoside-resistance genes [*n* = 23, *P* < 0.0186] and β-lactam-resistance genes [*n* = 15, *P* < 0.0264]) could be found in human reservoirs (Fig. 3B), indicating that *Morganella* from hospitalized patients had evolved to acquire many more ARGs to encounter complex and high selection of antimicrobials in hospital settings. A total of 18 acquired ARGs, involved in resistance to nine different categories of antimicrobials, were shared by *Morganella* isolates from all the above three reservoirs, denoting a long history of acquisition and wide dissemination of these ARGs in *Morganella*.

Forty-nine of the 60 isolates sequenced here harbored 63 of the above-mentioned 88 ARGs, and these 63 ARGs were involved in resistance to 15 (except for polymyxin) of the above-mentioned 16 antimicrobials (Fig. S2). These 63 ARGs were found in genospecies M. morganii (48/60, 90%) and *M. sibonii* (1/60, 1.67%). The top 5 ARGs in these 60 isolates were tetracycline-resistance genes (46/60, 81.67%), aminoglycoside-resistance genes (42/60, 70%), sulfonamide-resistance genes (34/60, 56.67%), chloramphenicol-resistance genes (30/60, 50%), and β-lactam-resistance genes (29, 48.33%); this observation was highly similar to the prevalence of 88 acquired ARGs in the 166 isolates described above.

**FIG 4:**
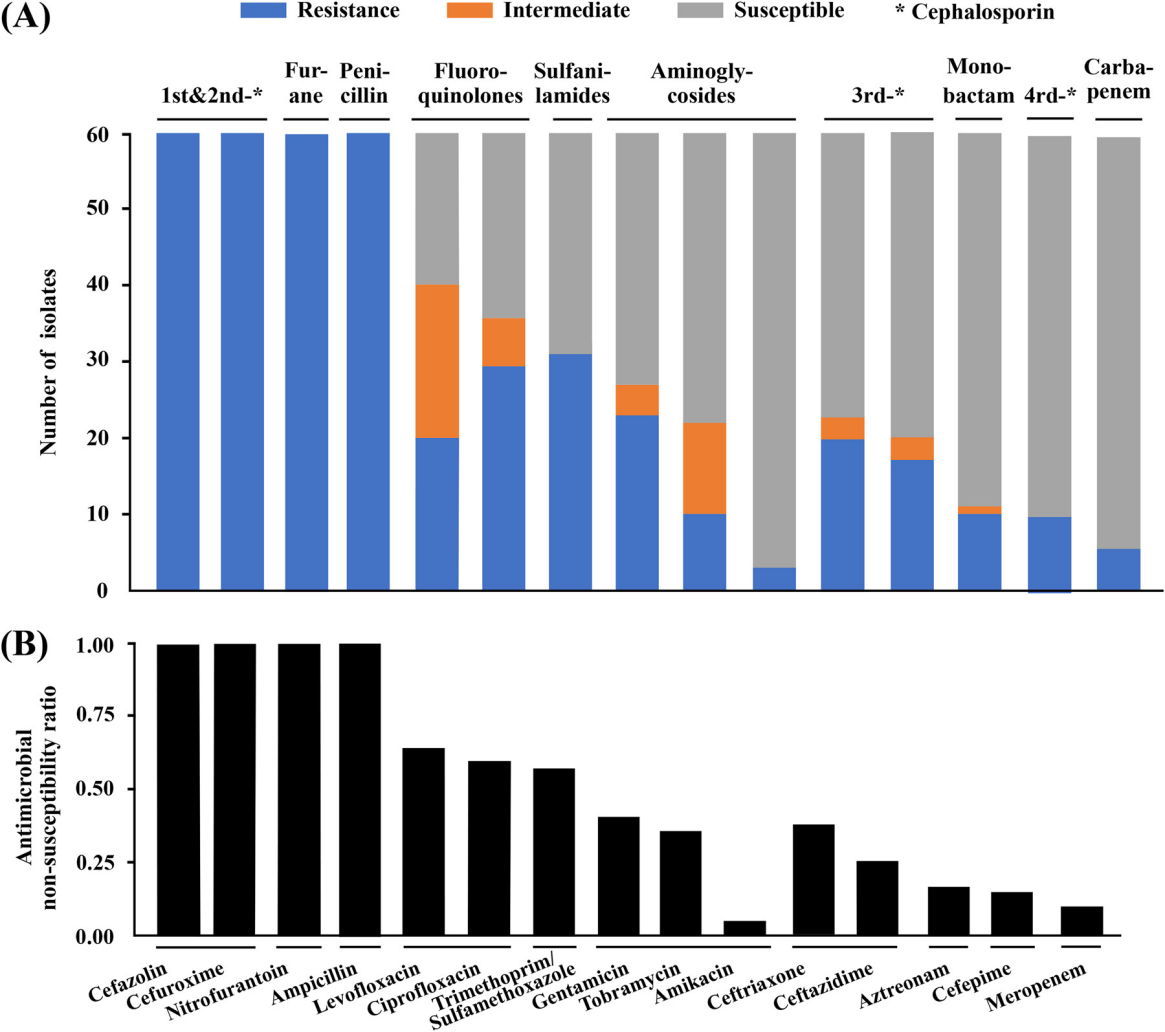
Antimicrobial susceptibility data of 60 *Morganella* isolates. (A) Shown are the antimicrobial resistance profiles of 60 *Morganella* isolates collected in this study. (B) Shown are the nonsusceptibility rates [(resistant + intermediate)/(sensitivity + intermediate + resistant)] of 60 *Morganella* isolates for each antimicrobial. Original data are shown in Table S1.

The antimicrobial susceptibility/resistance profiles of these 60 isolates were determined using 15 different antimicrobials (Fig. 4 and Table S1). As expected, all 60 of these isolates were highly resistance to ampicillin, cefazolin, cefuroxime, and nitrofurantoin due to intrinsic resistance. These 60 isolates displayed nonsusceptibility rates of >50% for three antimicrobials, 50% to 20% for four antimicrobials, and <20% for the remaining four antimicrobials, including aztreonam (18.34%, 11/60), cefepime (16.67%, 10/60), meropenem (10%, 6/60), and amikacin (5%, 3/60). *Morganella* isolates in China showed the highest nonsusceptibility rate (*n* = 40, 66.67%) for fluoroquinolones, including levofloxacin and ciprofloxacin. Meropenem and amikacin could be the first choice for experiential treatment of *Morganella*-induced infections in China because they have the lowest detected nonsusceptibility rates (≤10%). All six of the meropenem-resistance *Morganella* isolates discussed herein acquired the carbapenemase gene *bla*_KPC-2_ (*n* = 4) or *bla*_NDM-1_ (*n* = 2) (Table S1) and were confirmed to have carbapenemase activity in bacterial cell extracts. There were 25 of these 60 isolates that carried multiple aminoglycoside-modifying enzyme genes and thereby displayed resistance to gentamicin and tobramycin, but only 3 of these 25 isolates were nonsusceptible to amikacin due to the following two reasons: (i) amikacin was insensitive against these enzymes (17) and (ii) these 3 amikacin-resistant isolates additionally acquired the 16S rRNA methyltransferase gene *rmtB*, therefore mediating high-level amikacin resistance (18).

### A global view of AGEs in the 166 sequenced *Morganella* isolates.

AGEs acted as the vectors of ARGs and thus were responsible for the accumulation and dissemination of ARGs in different bacterial isolates by intracellular/intercellular transfer (19). To understand the prevalence of ARG-containing AGEs in *Morganella*, we screened the 166 sequenced *Morganella* isolates for the prevalence of the 17 major AGE groups frequently found in Gram-negative bacteria (Table 1). Detected were 6 of the above 17 groups: IS*26*/IS*15DI*, IS*10*, and IS*1R* and Tn*3-*, Tn*21*-, and Tn*7*-subfamily unit transposons were found in 69 (41.75%), 65 (39.16%), 39 (23.49%), 12 (7.23%), 33 (19.88%), and 23 (13.86%) of these 166 isolates. These six groups of AGEs were identified in the genospecies M. morganii (*n* = 82) and *M. sibonii* (*n* = 3) belonging to M. morganii complex and the genospecies *M. kristinii* (*n* = 1) belonging to *M. psychrotolerans* complex. Accordingly, the selection of 12 nonredundant isolates for whole-genome sequencing (see above) was based on the reason that they probably carried at least one of IS*26*/IS*15DI*-, IS*10*-, or IS*1R*-composite transposons and Tn*21*- and Tn*7*-subfamily unit transposons.

**TABLE 1:** The distribution of 17 major AGE groups in 166 global *Morganella* isolates

Family and subfamily	Core transposition determinant	Genospecies	No. of positive strains	%
IS				
IS*26*/IS*15DI*	*tnpA*	*Morganella. morganii*	67	41.75
IS*26*/IS*15DI*	*tnpA*	*M. sibonii*	1	
IS*26*/IS*15DI*	*tnpA*	*M. kristinii*	1	
IS*10*	*tnpA*	M. morganii	65	39.16
IS*1R*	*tnpA*	M. morganii	38	23.49
IS*1R*	*tnpA*	*M. sibonii*	1	
Tn*3*				
Tn*3*	*tnpAR*	M. morganii	12	7.23
Tn*21*	*tnpAR*	M. morganii	32	19.88
Tn*21*	*tnpAR*	*M. sibonii*	1	
Tn*163*	*tnpAR*		0	0
Tn*4430*	*tnpAR*		0	0
Tn*4651*	*tnpAR*		0	0
Tn*4401*	*tnpAR*		0	0
Tn*7*				
Tn*7*	*tnsABCDE*	M. morganii	23	13.86
Tn*6230*	*tnsABCD*		0	0
Tn*552*	*tnsCBR*		0	0
Tn*6022*	*tnsABCDE*		0	0
Tn*5053*	*tniABQR*		0	0
Tn*554*				
Tn*554*	*tnpABC*		0	0
Tn*6488*	*ginABCD*		0	0
Tn*6571*	*ginABCD*		0	0

### A collection of 23 AGEs for detailed sequence comparison.

Each of these fully sequenced 12 isolates harbored 1 to 3 kinds of chromosomal AGEs, giving a total of 18 identified (Table S5). Additionally, a total of 11 plasmids were identified from 7 of these 12 isolates (see Table S6 for details). Subsequent analysis was then focused on these 18 chromosomal AGEs, further dividing into eight distinct groups: (i) two IS*26*/IS*15DI*-composite transposons, Tn*6759* and Tn*6760* from strains 11759 and 621164, respectively, (ii) three IS*10*-composite transposons, Tn*10*, Tn*6798*, and Tn*6799* from strains ZJC25/229813/516602/11759, 621164, and 715304, respectively, together with a 43.1-kb Tn*10*-related element from strain GN28 designated T10RE_GN28_, (iii) two Tn*7*-related unit transposons, Tn*7* and Tn*6800* from strains 229813 and ZJC25, respectively, together with a 42.3-kb Tn*7*-related element T7RE_621164_ from strain 621164, (iv) four Tn*1696*-related unit transposons, Tn*6913a*, Tn*6913b*, Tn*6914*, and Tn*6915* from strains ZJG812, ZJG944, ZJC25, and 229813, respectively, together with a 63.8-kb Tn*1696*-related element T1696RE_ZJD581_ from strain ZJD581, (v) a Tn*6963*-related transposable prophage Tn*6964* from strain 12304, (vi) a Tn*6872*-related IME Tn*6966* from strain 81703, (vii) a Tn*6397*-related ICE Tn*6967* from strain ZJG944, and (viii) a 35.8-kb Tn*2670*-related element T2670RE_11759_ from strain 11759. All of these T10RE, T7RE, T1696RE, and T2670RE elements could not be recognized as intact transposons due to truncation of relevant core transposition modules. A detailed sequence comparison was applied to these 18 AGEs together with five prototype AGEs, Tn*1696*, Tn*6963*, Tn6*872*, Tn*6397*, and Tn*2670* from GenBank (Table 2). At least 52 ARGs, involved in resistance to 15 different categories of antimicrobials, were identified in these 23 AGEs (Table S5).

**TABLE 2:** Major features of AGEs characterized in this study

Group	AGE^*a*^	Accession no.	Chromosomal nucleotide position	Length (bp)	Host bacterium	Reference
IS*26*/IS*15DI*-composite transposons	Tn*6759*	CP059986	3662767–3668698	5,932	Morganella morganii 11759	This study
	Tn*6760*	CP064829	2991196–3002147	10,952	M. morganii 621164	This study
Tn*10*-related elements	Tn*10*_ZJC25_	CP064828	1486140–1495286	9,147	M. morganii ZJC25	This study
	Tn*10*_229813_	CP043955	1519262–1528408	9,147	M. morganii 229813	This study
	Tn*10*_516602_	CP064054	2606745–2615891	9,147	M. morganii 516602	This study
	Tn*10*_11759_	CP059986	1720225–1729371	9,147	M. morganii 11759	This study
	Tn*6798*	CP064829	1802073–1811996	9,924	M. morganii 621164	This study
	Tn*6799*	CP064833	1990256–1990256	38,672	M. morganii 715304	This study
	T10RE_GN28_	CP064055	2305754–2348095	42,342	M. morganii GN28	This study
Tn*7*-related elements	Tn*7*_ZJC25_	CP064828	19506–33572	14,067	M. morganii ZJC25	This study
	Tn*7*_229813_	CP043955	19469–33535	14,067	M. morganii 229813	This study
	Tn*6800*	CP064830	22578–37741	15,164	M. morganii 81703	This study
	T7RE_621164_	CP064829	20656–62976	42,321	M. morganii 621164	This study
Tn*1696*-related elements	Tn*1696*	U12338	Not applicable	16,318	Pseudomonas aeruginosa R1033	23
	Tn*6913a*	CP064831	1916898–1963812	46,915	M. morganii ZJG812	This study
	Tn*6913b*	CP064827	2071073–2117588	46,516	M. morganii ZJG944	This study
	Tn*6914*	CP064828	1968547–1982455	13,909	M. morganii ZJC25	This study
	Tn*6915*	CP043955	2001667–2051662	49,996	M. morganii 229813	This study
	T1696RE_ZJD581_	CP064826	2049692–2113491	63,800	M. morganii ZJD581	This study
Tn*6963*-related transposable prophages	Tn*6963*	CP033056	2191792–2236217	44,426	M. morganii L241	24
	Tn*6964*	CP064832	2277663–2371532	93,870	M. morganii 12304	This study
Tn*6872*-related IMEs	Tn*6872*	LR134189	3025117–3045606	20,490	Providencia rustigianii NCTC6933	Not applicable
	Tn*6966*	CP064830	3371703–3442829	71,127	M. morganii 81703	This study
Tn*6397*-related ICEs	Tn*6397*	CP021851	386444–510401	123,958	Enterobacter cloacae A1137	27
	Tn*6967*	CP064827	628561–738154	109,594	M. morganii ZJG944	This study
Tn*2670*-related elements	Tn*2670*	AP000342	Not applicable	22,760	Shigella flexneri R100	28
	T2670RE_11759_	CP059986	2289505–2325366	35,862	M. morganii 11759	This study

a T10RE_GN28_, T2670RE_11759_, T7RE_621164_, and T1696RE_ZJD581_ would lose their intracellular mobility due to the lesion of their core transposition determinants IS*10*, IS*1R*, *tnsABCDE*, and *tnpAR*, respectively. The remaining AGEs are intact and would have intracellular or intercellular mobility.

### Two IS*26*/IS*15DI*-composite transposons, Tn*6759* and Tn*6760*.

**FIG 5:**
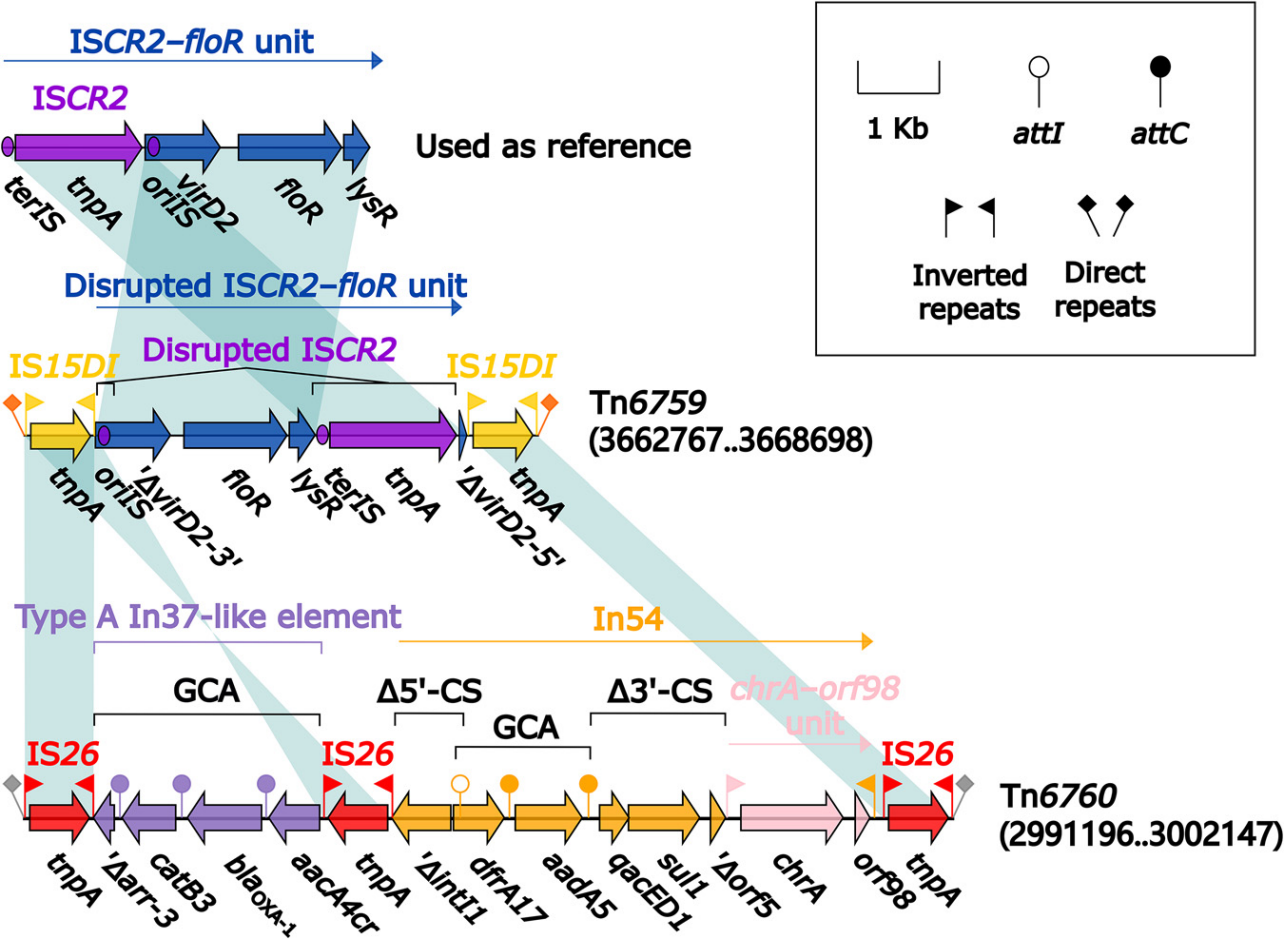
Comparison of two IS*26*/IS*15DI*-composite transposons. Genes are denoted by arrows. Genes, AGEs, and other features are colored based on their functional classification. Shading denotes regions of homology (nucleotide identity ≥ 95%). Numbers in brackets indicate nucleotide positions within the chromosomes of strains 11759 and 21164, respectively. Accession number of IS*CR2*-*floR* unit (53) used as reference is CP042857.

Tn*6759* and Tn*6760* (Fig. 5) from two *Morganella* isolates were inserted at different chromosomal locations and bracketed by 8-bp direct repeats (DRs; target site duplication signals for transposition). Tn*6759* was bound by two copies of IS*26*, while Tn*6760* was bound by two copies of IS*15DI*, and these two IS elements belonged to IS2*6* family and had only three point variation sites on their nucleotide sequences. Tn*6759* and Tn*6760* carried completely different antimicrobial resistance loci (ARLs): (i) a disrupted IS*CR2*–*floR* unit in Tn*6759* and (ii) a type A In37-like element with a truncated gene cassette array (GCA) *aacA4cr*–*bla*_OXA-1_–*catB3*–Δ*arr-3*, plus a concise class 1 integron In54 with a GCA *dfrA17*–*aadA5* in Tn*6760*.

### Tn*10* and its derivatives Tn*6798*, Tn*6799*, and T10RE_GN28_.

**FIG 6:**
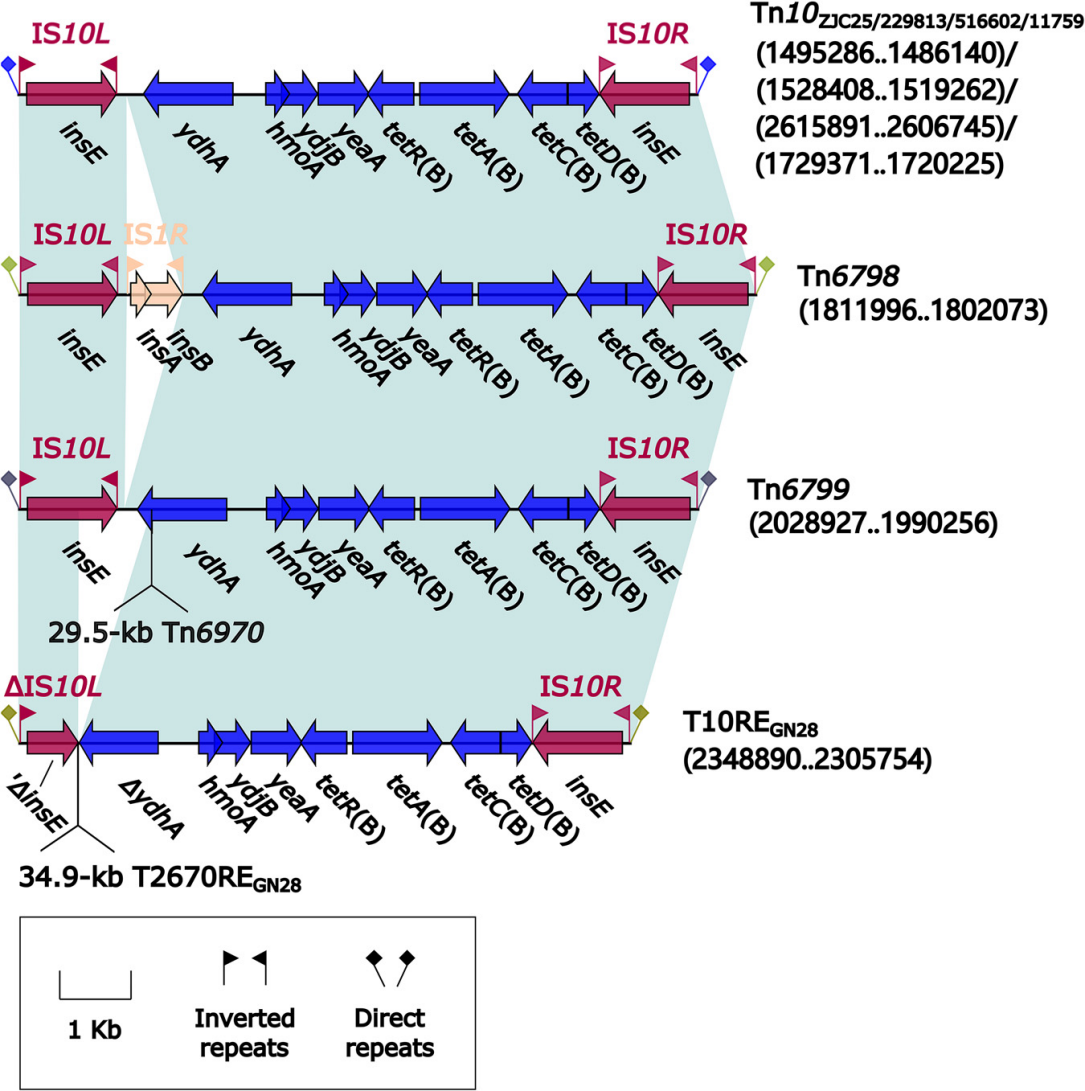
Comparison of Tn*10* and its three derivatives. Genes are denoted by arrows. Genes, AGEs, and other features are colored based on their functional classification. Shading denotes regions of homology (nucleotide identity ≥ 95%). Numbers in brackets indicate nucleotide positions within the chromosomes of strains ZJC25, 229813, 516602, 11759, 621164, 715304, and GN28, respectively.

Tn*10* was initially described in Shigella flexneri plasmid R100, and it was a prototype IS*10*-composite transposon carrying a class B tetracycline-resistance module *tetRACD* (20). Here, Tn*10* and its three derivatives (Fig. 6) from seven *Morganella* isolates were inserted at five different chromosomal locations and bracketed by 9-bp DRs. Compared to Tn*10*, its three derivatives underwent two major insertion events: (i) IS*1R* was inserted downstream of *ydhA* in Tn*6798* and (ii) Tn*2670-*related transposon Tn*6970* (see below) and 34.9-kb T2670RE_GN28_ (see below) were inserted at the same site within *ydhA* in Tn*6799* and T10RE_GN28_, respectively, leading to truncation of *ydhA* in Tn*6799* and that of *ydhA* plus IS*10L* in T10RE_GN28_.

### Tn*7* and its derivatives Tn*6800* and T7RE_621164_.

**FIG 7:**
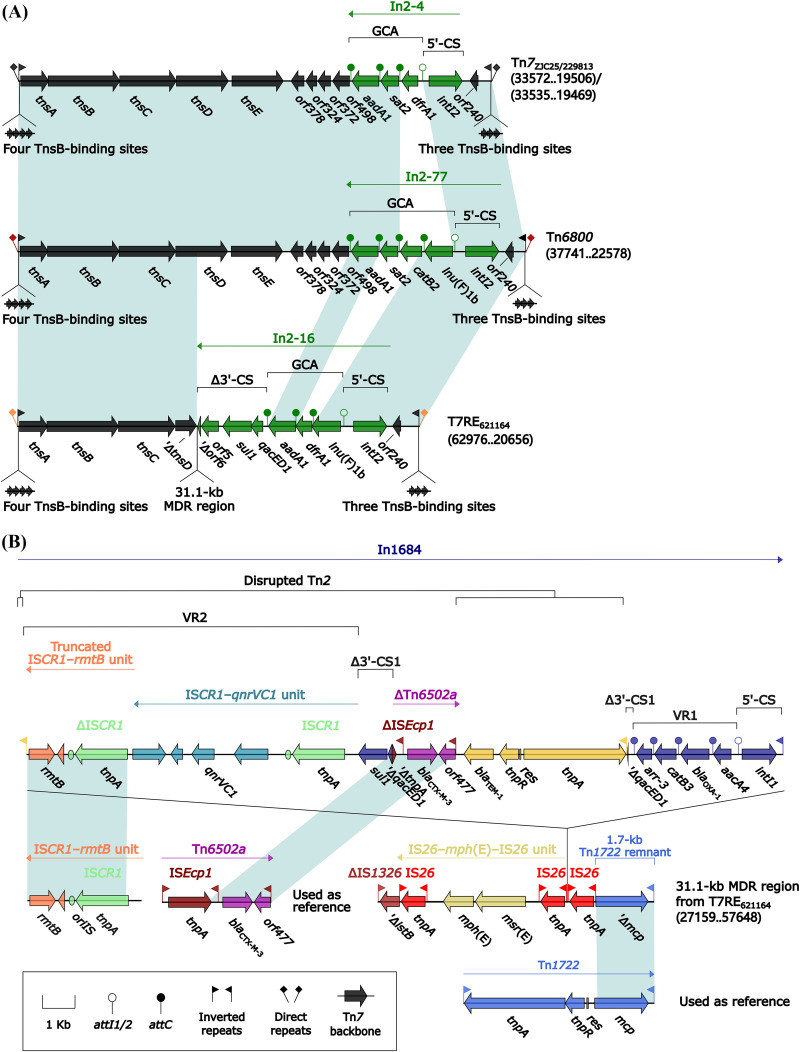
Comparison of Tn*7* and its two derivatives. (A) Organization of Tn*7* and its two derivatives. (B) Organization of 31.1-kb MDR region from 42.3-kb T*7*RE_621164_. Genes are denoted by arrows. Genes, AGEs, and other features are colored based on their functional classification. Shading denotes regions of homology (nucleotide identity ≥ 95%). Numbers in brackets indicate nucleotide positions within the chromosomes of strains ZJC25, 229813, 81703, and 621164, respectively. Accession numbers of IS*CR1*–*rmtB* unit, Tn*6502a* (54), and Tn*1722* (55) are CP059348, KF914891, and X61367, respectively.

The prototype unit transposon Tn*7* was initially found in Escherichia coli plasmid R483 and composed of the core transposition module *tnsABCDE* and a class 2 integron In2-4 (GCA: *dfrA1*–*sat2*–*aadA1*) (21). Here, Tn*7* and its two derivatives (Fig. 7) from four *Morganella* isolates were integrated at the same chromosomal location and bracketed by 5-bp DRs. Tn*6800* or T7RE_621164_ differed from Tn*7* by acquisition of In2-77 with GCA *lnu*(F)*1b*–*catB2*–*sat2*–*aadA1* or In2-16 with GCA *lnu*(F)*1b*–*catB2*–*sat2*–*aadA1*, respectively, instead of In2-4 (Fig. 5A). T7RE_621164_ underwent an additional insertion event: a 31.1-kb multidrug resistance (MDR) region was inserted within *tnsD* (Tn*7* target-site selection protein), leading to truncation of *tnsABCDE* (Fig. 7A). This MDR region (Fig. 7B) harbored two ARLs: IS*26*–*mph*(E)–IS*26* unit and In1684. In1684 was a complex class 1 integron carrying *aacA4*–*bla*_OXA-1_–*catB3*–*arr-3* (GCA/VR1: variable region 1), a disrupted Tn*2* containing *bla*_TEM-1_, ΔTn*6502a* containing *bla*_CTX-M-3_, and VR2 (IS*CR1*–*qnrVC1* unit plus truncated IS*CR1*–*rmtB* unit).

### Five Tn*1696* derivatives, Tn*6913a*, Tn*6913b*, Tn*6914*, Tn*6915*, and T1696RE_ZJD581_.

**FIG 8:**
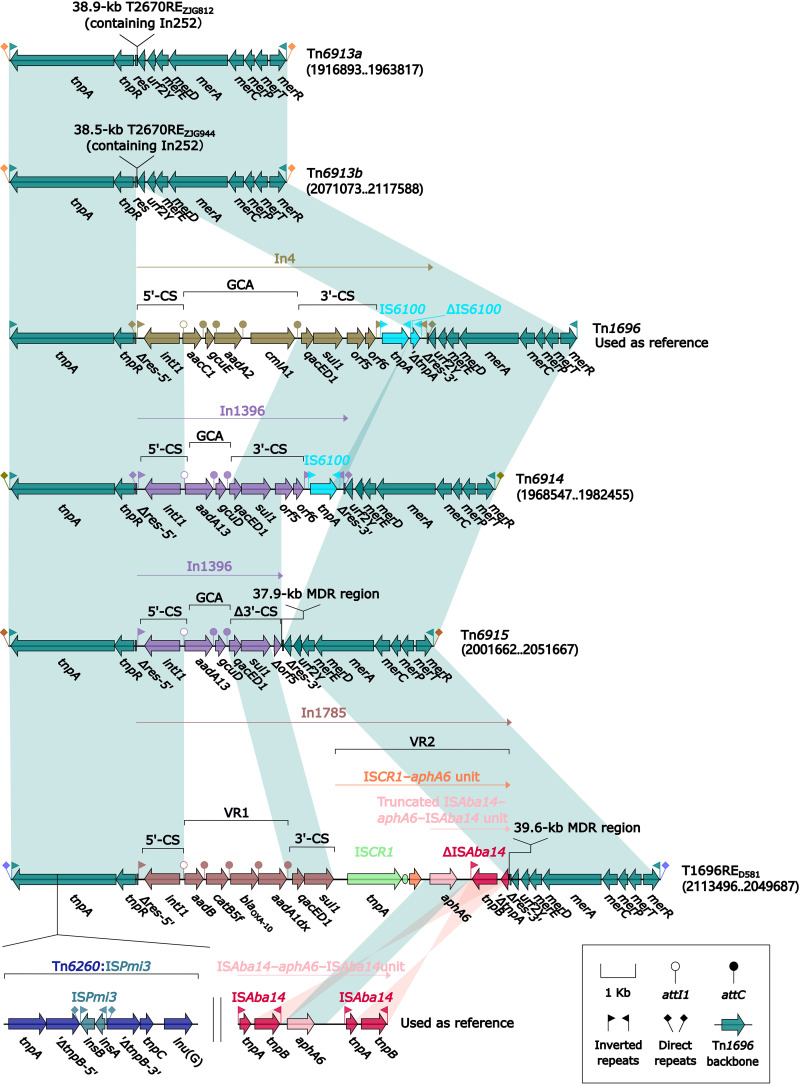
Comparison of Tn*1696* and its five derivatives. Genes are denoted by arrows. Genes, AGEs, and other features are colored based on their functional classification. Shading denotes regions of homology (nucleotide identity ≥ 95%). Numbers in brackets indicate nucleotide positions within the chromosomes of strains ZJG944, ZJG812, ZJC25, 229813, and ZJD581, respectively. Accession numbers of Tn*1696* (22) and IS*Aba14*–*aphA6*–IS*Aba14* unit (56) used as reference are U12338 and CP046406, respectively.

Tn*1696* was initially found in Pseudomonas aeruginosa plasmid R1033 (22). It was one of the Tn*21*-subfamily prototype unit transposons and had a backbone structure, IRL (inverted repeat left)–*tnpA* (transposase)–*tnpR* (resolvase)–*res* (resolution site)–*mer* (mercury resistance operon)–IRR (inverted repeat right), with integration of a class 1 integron In4 (GCA: *aacC1*–*gcuE*–*aadA2*–*cmlA1*) into *res* (23). Here, the five Tn*1696* derivatives (Fig. 8) from five *Morganella* isolates were inserted into three different chromosomal locations and bracketed by 5-bp DRs. Each of these five derivatives acquired a unique ARL instead of In4 in Tn*1696*: 38.9-kb T2670RE_ZJG812_ (see below) in Tn*6913a*, 38.5-kb T2670RE_ZJG944_ (see below) in Tn*6913b*, In1396 (GCA: *aadA13*–*gcuD*) in Tn*6914*, In1396 plus a 37.9-kb MDR region (see below) in Tn*6915*, and In1785 (VR1: *aadB*–*catB5f*–*bla*_OXA-10_–*aadA1dx*; VR2: IS*CR1*–*aphA6* unit) plus 39.6-kb MDR region (see below) in T1696RE_ZJD581_. Additionally, *tnpA* of T1696RE_ZJD581_ was interrupted by Tn*6260*:IS*Pmi3*.

### Two transposable prophages, Tn*6963* and Tn*6964*.

**FIG 9:**
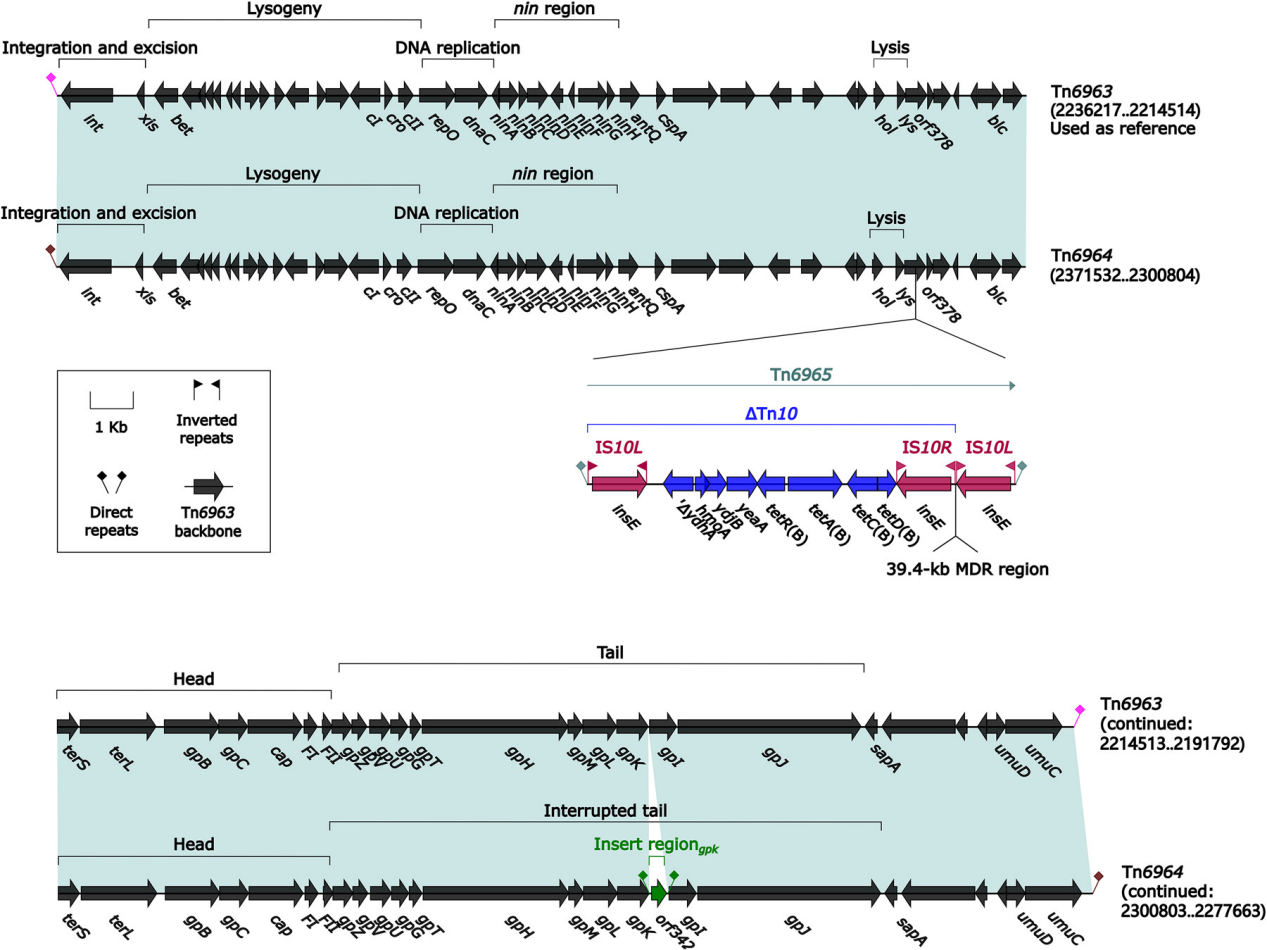
Comparison of two Tn*6963*-related transposable prophages. Genes are denoted by arrows. Genes, AGEs, and other features are colored based on their functional classification. Shading denotes regions of homology (nucleotide identity ≥ 95%). Numbers in brackets indicate nucleotide positions within the chromosomes of strains L241 and 12034, respectively. Accession number of Tn*6963* (24) used as reference is CP033056.

The prototype transposable prophage Tn*6963* was initially found in M. morganii L241 (24). Tn*6963* and Tn*6964* (Fig. 9) shared conserved λ phage life cycle-related markers *attL*/*attR* (attachment sites at left/right ends), *int*–*xis* (integration and excision), *bet*–to–*cII* (lysogeny), *repO*–*dnaC* (DNA replication), *hol*–*lys* (lysis), *terSL*–*gpBC*–*cap*–*FI*–*FII* (head assembly), and *gpZVUGTHMLKIJ* (tail assembly) (25). Tn*6963* contained no accessory modules, while Tn*6964* acquired two: IS*10*-composite transposon Tn*6965* that harbored truncated Tn*10* carrying *tetRACD*(B) plus 39.4-kb MDR region (see below), and so called “inserted region” (26) that was cryptic and bracketed by 2-bp DRs.

### Two IMEs, Tn*6872* and Tn*6966*.

**FIG 10:**
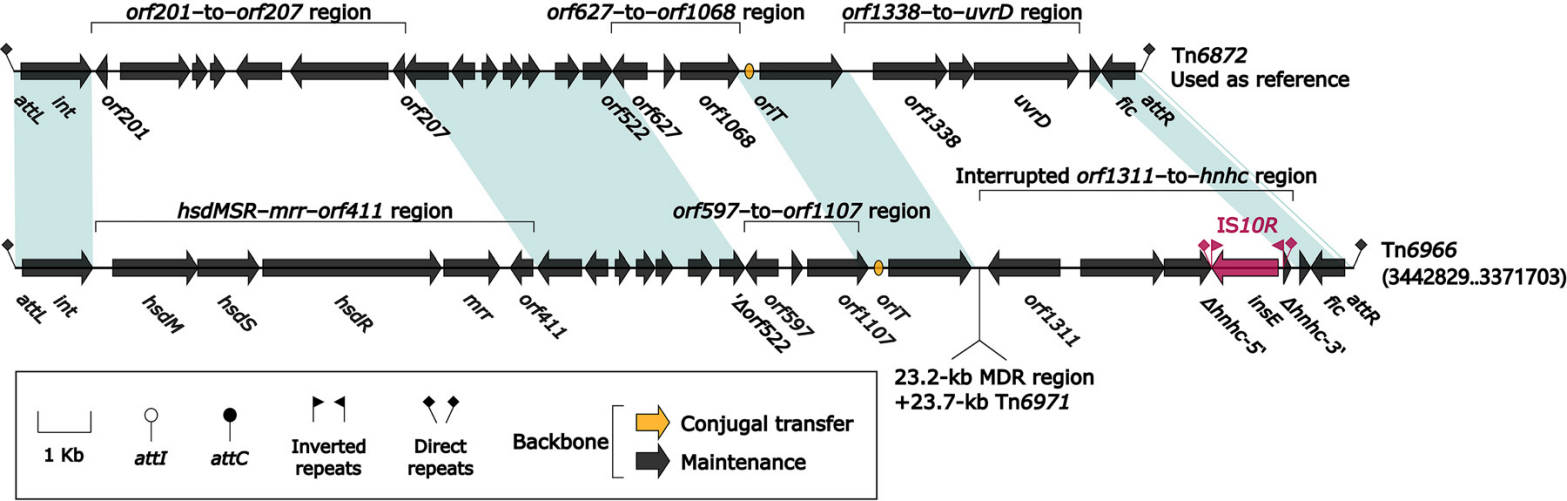
Comparison of two Tn*6872*-related IMEs. Genes are denoted by arrows. Genes, AGEs, and other features are colored based on their functional classification. Shading denotes regions of homology (nucleotide identity ≥ 94%). Numbers in brackets indicate nucleotide positions within the chromosome of strain 81703. Accession number of Tn*6872* used as reference is LR134189.

The prototype IME Tn*6872* was initially found in Providencia rustigianii NCTC6933 (accession number LR134189). Tn*6872* and Tn*6966* (Fig. 10) shared core IME backbone markers *attL/attR*, *int*, and *oriT* (origin of conjugative replication), but they displayed dramatic modular variations cross the backbones: Tn*6872* had its unique regions *orf201* to *orf207*, *orf627* to *orf1068*, and *orf1338* to *uvrD*, while Tn*6966* contained the counterparts *hsdMSR*–*mrr*–*orf411*, *orf597* to *orf1107*, and *orf1311* to *hnhc*, respectively. Tn*6872* carried no accessory modules, while Tn*6966* acquired two: IS*10R* and a region composed of 23.2-kb MDR region (see below) plus Tn*21*-related transposon Tn*6971* (see below).

### Two ICEs, Tn*6397* and Tn*6967*.

**FIG 11:**
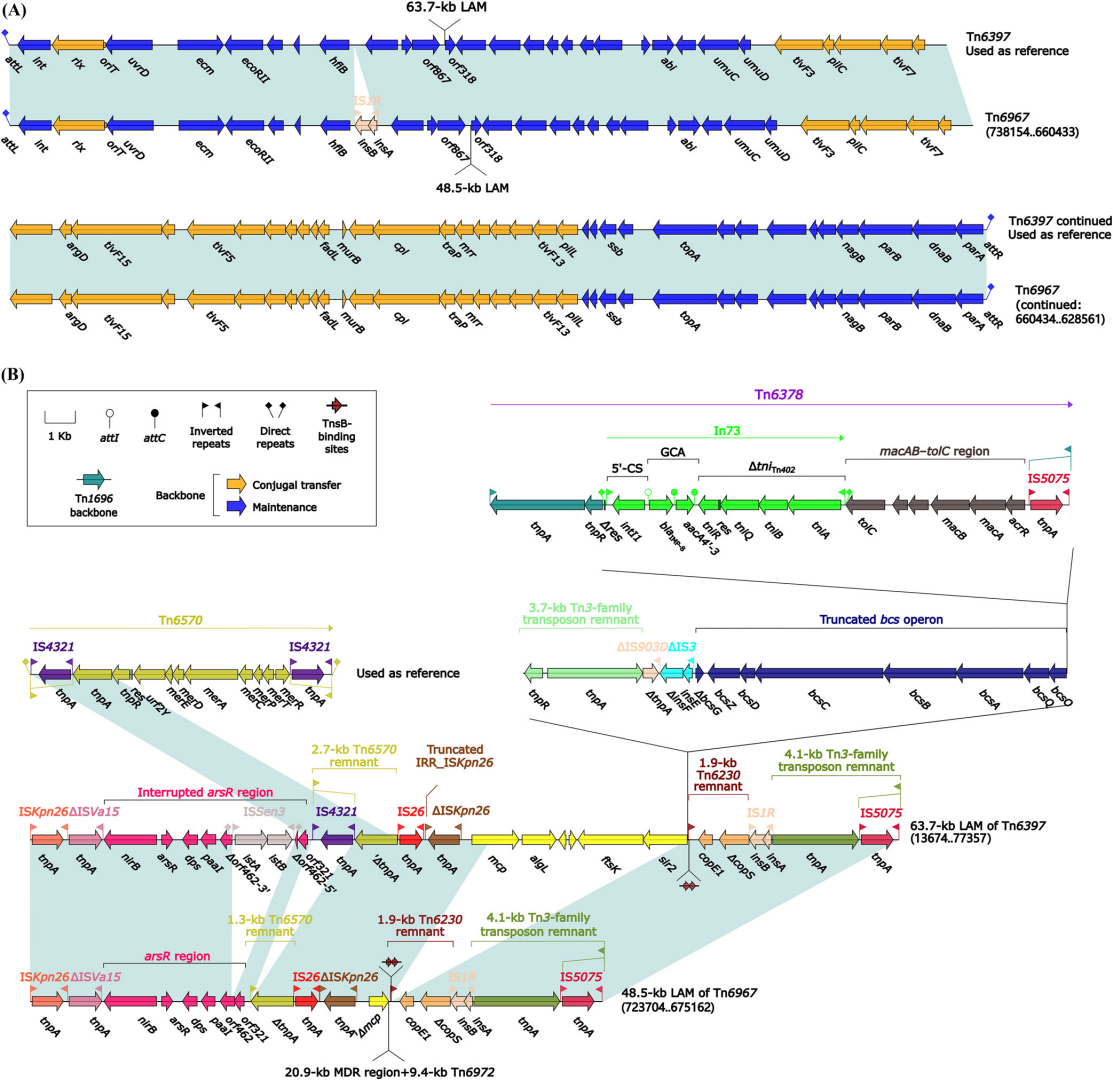
Comparison of two Tn*6397*-related ICEs. (A) Organization of Tn*6397* and Tn*6967*. (B) Organization of LAMs of Tn*6397* and Tn*6967*. Genes are denoted by arrows. Genes, mobile elements, and other features are colored based on their functional classification. Shading denotes regions of homology (nucleotide identity ≥ 95%). Numbers in brackets indicate nucleotide positions within the chromosome of strain ZJG944. Accession numbers of Tn*6397* (27) and Tn*6570* used as reference are CP021851 and CP043397, respectively.

The prototype ICE Tn*6397* was initially found in *Enterobacter* spp. A1137 (27). Tn*6397* and Tn*6967* (Fig. 11A) shared conserved ICE backbone markers *attL/attR*, *int*, *xis*, *rlx* (relaxase), *oriT*, *cpl* (coupling protein), and F (TivF)-type type IV secretion system gene set (mating pair formation). Tn*6397* and Tn*6967* each harbored a unique large accessory module (LAM) inserted at the same site within ICE backbones. These two LAMs (Fig. 11B) had similarity in gene organizations but exhibited totally different profiles of ARLs: (i) Tn*1696*-related transposon Tn*6378* carrying In73 (GCA: *bla*_IMP-8_–*aacA4'-3*) plus macrolide-resistance locus *macAB*-*tolC* in 63.7-kb LAM of Tn*6397* and (ii) a 20.9-kb MDR region (see below) plus a Tn*21*-related transposon Tn*6972* (see below) in 48.5-kb LAM of Tn*6967*.

### Two Tn*21*-subfamily transposons, Tn*6971* and Tn*6972*.

**FIG 12:**
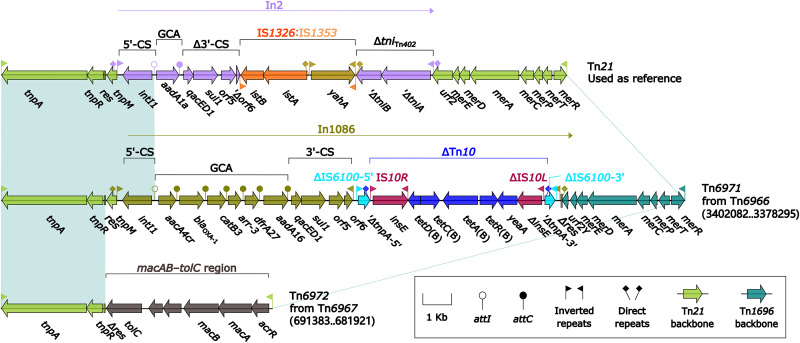
Comparison of Tn*21* and its two derivatives. Genes are denoted by arrows. Genes, AGEs, and other features are colored based on their functional classification. Shading denotes regions of homology (nucleotide identity ≥ 95%). Numbers in brackets indicate nucleotide positions within the chromosomes of strains 81703 and ZJG944, respectively. Accession number of Tn*21* (28) is AF071413.

The sequence comparison (Fig. 12) was also applied to the two Tn*21* derivatives Tn*6971* and Tn*6972* (identified as the inner components of Tn*6966* and Tn*6967*, respectively; see above), together with Tn*21* (28). Tn*21*, initially found in Shigella flexneri plasmid R100 (28), was another Tn*21*-subfamily prototype transposon, and it displayed the backbone structure IRL–*tnpAR*–*res*–*mer*–IRR with the integration of In2. Tn*6971* and Tn*6972* (Fig. 12) harbored Tn*21* core transposition determinants *tnpAR* and IRL/IRR, but Tn*1696 mer* locus, instead of that in Tn*21*, was found in Tn*6971*, while Tn*6972* did not contain *mer*. Tn6971 and Tn6972 each acquired a unique ARL: In1086 [which had GCA *aacA4cr*–*bla*_OXA-1_–*catB3*–*arr-3*–*dfrA27*–*aadA16* and was additionally inserted with *tetRACD*(B)-carrying ΔTn*10* in Tn*6971* and *macAB-tolC* in Tn*6972*].

### Five Tn*2670* derivatives, Tn*6970*, T2670RE_11759_, T2670RE_GN28_, T2670RE_ZJG812_, and T2670RE_ZJG944_.

**FIG 13:**
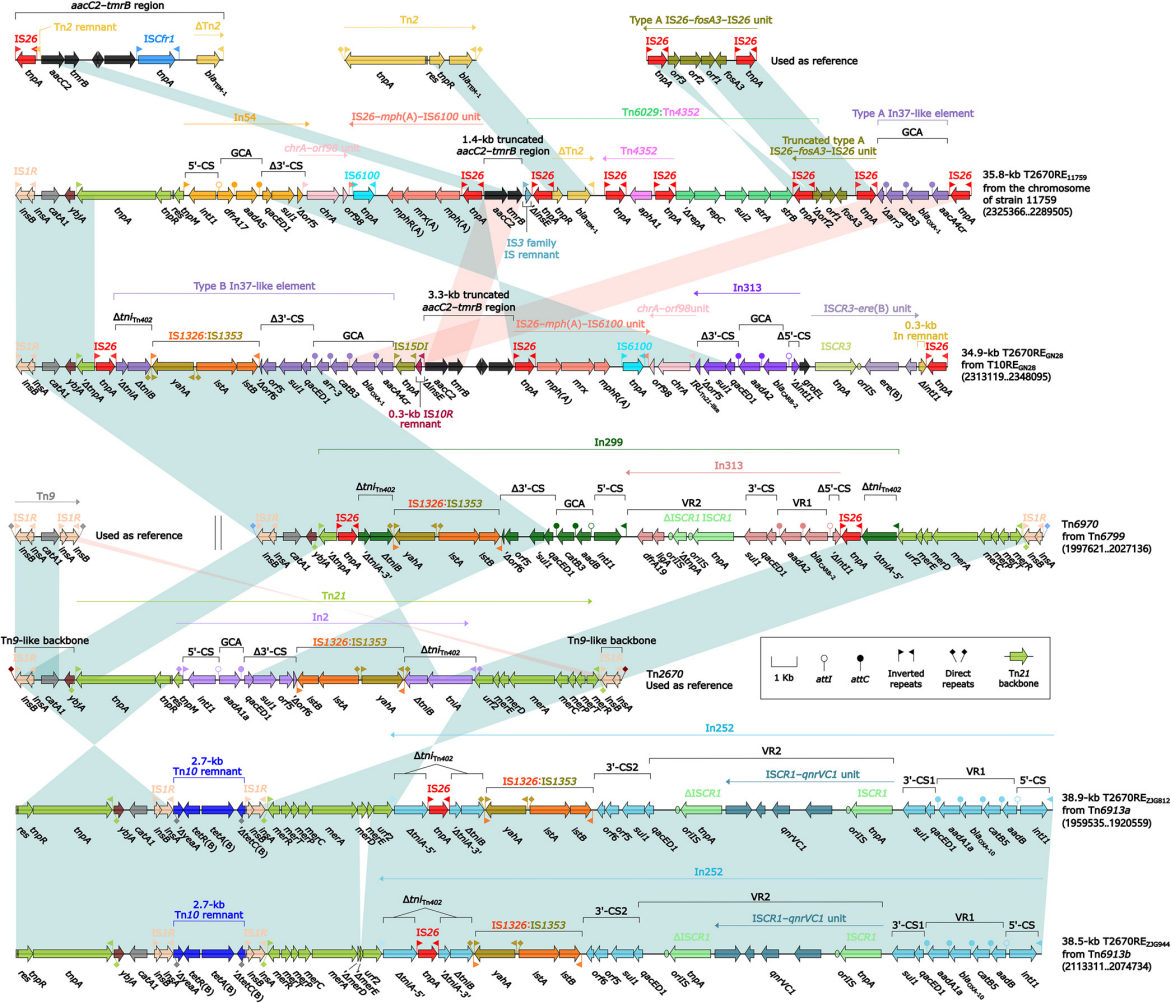
Comparison of Tn*2670* and its five derivatives. Genes are denoted by arrows. Genes, AGEs, and other features are colored based on their functional classification. Shading denotes regions of homology (nucleotide identity ≥ 95%). Numbers in brackets indicate nucleotide positions within the chromosomes of strains 11759, GN28, 81703, ZJG944, and ZJG812, respectively. Accession numbers of *aacC2*–*tmrB* region (57), Tn*2* (58), type A IS*26*–*fosA3*–IS*26* unit (59), Tn*9* (29), Tn*2670* (28), and Tn*10* (20) used as reference are JX101693, X64367, KP987215, V00622, AP000342, and AP000342, respectively.

The sequence comparison (Fig. 13) was also applied to five Tn*2670* derivatives, Tn*6970*, T2670RE_11759_, T2670RE_GN28_, T2670RE_ZJG812_, and T2670RE_ZJG944_, together with Tn*2670* (28). T2670RE_11759_ was directly integrated into the chromosome, while the remaining four were recognized as the inner components of Tn*10* derivatives Tn*6799* and T10RE_GN28_ and Tn*1696* derivatives Tn*6913a* and Tn*6913b* (see above). Tn*2670* was an IS*1R*-composite transposon composed of a Tn*9*-like backbone (29) with the integration of Tn*21* (30), and it was initially found in Shigella flexneri plasmid R100 (28). Compared to Tn*2670*, Tn*6970*, T2670RE_ZJG812_, and T2670RE_ZJG944_ harbored the same intact Tn*9*-like backbone but contained different versions of truncated Tn*21* with integration of two different integrons: (i) In299 [GCA: *lnu*(F)*1b*–*catB2*–*sat2*–*aadA1*] inserted with In313 (VR1: *bla*_CARB-2_–*aadA2* and VR2: IS*CR1*–ΔIS*CR1*–*ligA*–*dfrA19*) in Tn*6970* and (ii) In252 (VR1: *aadB*–*catB5*–*bla*_OXA-10_–*aadA1a*; VR2: IS*CR1*–*qnrVC1* unit) in T2670RE_ZJG812_ and T2670RE_ZJG944_. T2670RE_11759_ and T2670RE_GN28_ contained the whole 5′-terminal Tn*9*-like backbone and a very small Tn*21* remnant; moreover, each of them acquired a unique LAM, the resulting two LAMs shared one ARL [IS*26*–*mph*(A)–IS*6100* unit], and each LAM further acquired five or four different ARLs: (i) In54, 1.4-kb truncated *aacC2*–*tmrB* region, Tn*6029* (containing *bla*_TEM-1_, *sul2*, *strA*, and *strB*) interrupted by Tn*4352* (containing *aphA1*), truncated type A IS*26*–*fosA3*–IS*26* unit, and type A In37-like element in T2670RE_11759_ and (ii) type B In37-like element (GCA: *aacA4cr*–*bla*_OXA-1_–*catB3*–*arr-3*), 3.3-kb truncated *aacC2*–*tmrB* region, In313 (GCA: *bla*_CARB-2_–*aadA2*), and IS*CR3*–*ereB* unit in T2670RE_GN28_. Notably, eight and five copies of IS*26*/IS*15DI* and IS*6100* were presented in T2670RE_11759_ and T2670RE_GN28_, respectively; all these IS elements belonged to IS*6* family and possessed almost identical 14-bp IR sequences, and thus they would be together involved in complex homologous recombination events, promoting the assembly of LAMs in T2670RE_11759_ and T2670RE_GN28_.

### Five *aacC4*/*aph(4)-Ia*/*sul2*/*floR*-carrying MDR regions.

**FIG 14:**
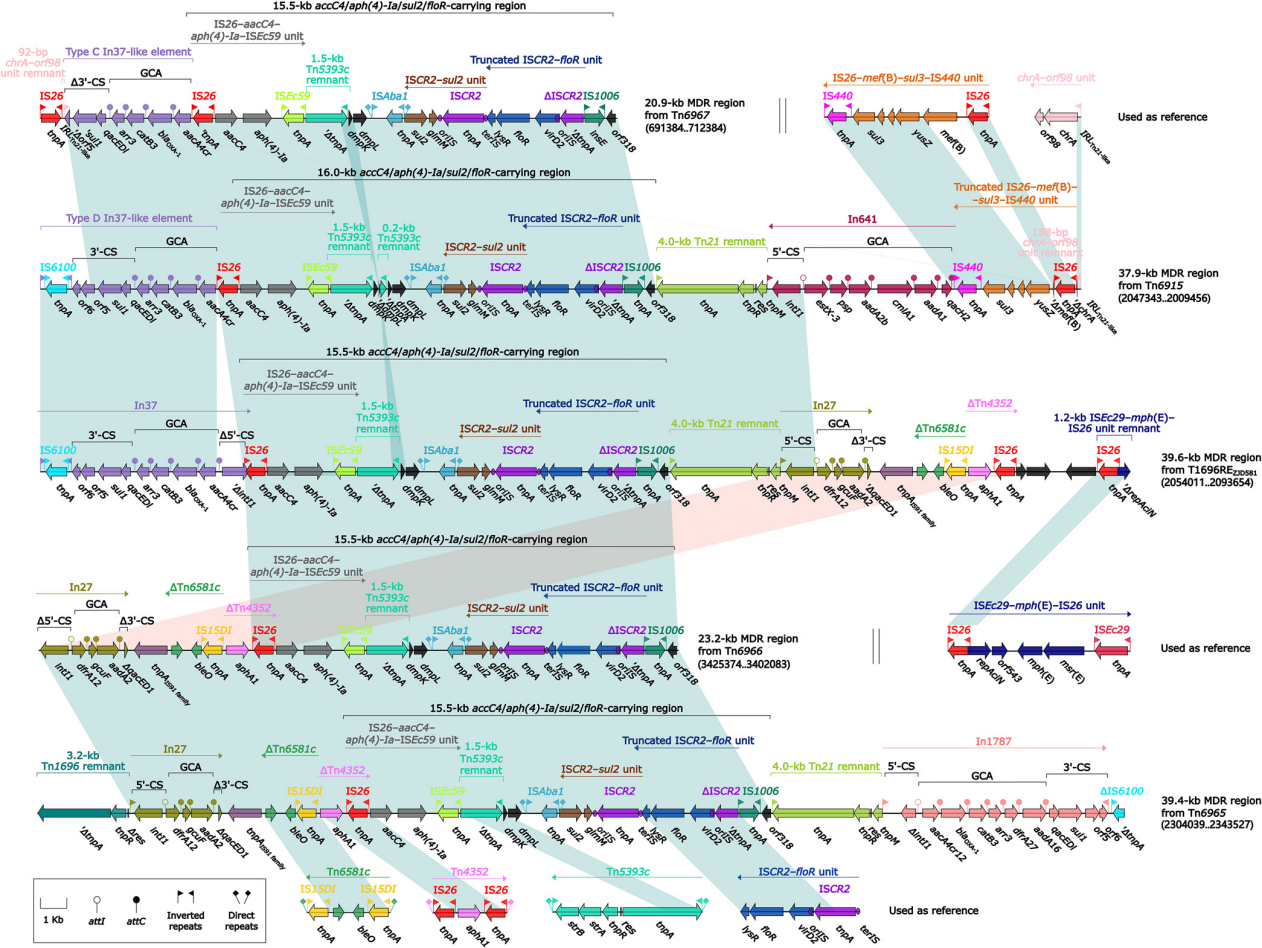
Comparison of five *aacC4*/*aph(4)-Ia*/*sul2*/*floR*-carrying MDR regions. Genes are denoted by arrows. Genes, AGEs, and other features are colored based on their functional classification. Shading denotes regions of homology (nucleotide identity ≥ 95%). Numbers in brackets indicate nucleotide positions within the chromosomes of strains ZJG944, 229813, ZJD581, 81703, and 12034, respectively. Accession numbers of IS*26*–*mef*(B)–*sul3*–IS*440* unit (60), *chrA*–*orf98* unit (53), IS*Ec29*–*mph*(E)–IS26 unit (61), Tn*6581c*, Tn*4352* (62), Tn*5393c* (63), and IS*CR2*–*floR* unit (53) are FJ196385, CP042858, AF550415, CP042857, CP042858, AF262622, and CP042857, respectively.

The sequence comparison (Fig. 14) was also applied to five different MDR regions (identified as the inner components of Tn*6967*, Tn*6915*, T1696RE_ZJD581_, Tn*6966*, and Tn*6965*, respectively; see above), which shared 15.5/16.0-kb *aacC4*/*aph(4)-Ia*/*sul2*/*floR*-carrying region. All these 15.5/16.0-kb regions comprised three major ARLs: IS*26*–*aacC4*–*aph(4)-Ia*–IS*Ec59*, IS*CR2*–*sul2* unit, and truncated IS*CR2*–*floR* unit. In addition, each of these five MDR regions integrated one or more additional ARLs: (i) In37, type C In37-like element, and type D (all contained the intact GCA *aacA4cr*–*bla*_OXA-1_–*catB3*–*arr-3*) in MDR regions from T1696RE_ZJD581_, Tn*6967*, and Tn*6915*, respectively, (ii) In27 (GCA: *dfrA12*–*aadA2*) plus *aphA1*-containing ΔTn*4352* as shared by MDR regions from T1696RE_ZJD581_, Tn*6966*, and Tn*6965*, (iii) truncated IS*26*–*mef*(B)–*sul3*–IS*440* unit and In641 (GCA: *aadA2*–*cmlA1a*–*aadA1a*–*qacH2*) in MDR region from Tn*6915*, and (iv) In1787 (GCA: *aacA4cr12*–*bla*_OXA-1_–*catB3*–*arr-3*–*dfrA27*–*aadA16*) in MDR region from Tn*6965*.

### Newly identified or designated AGEs.

There were 19 newly identified AGEs in total: (i) 12 of them directly integrated into the chromosomes and included 4 composite transposons, Tn*6759*, Tn*6760*, Tn*6798*, and Tn*6799*, 5 unit transposons, Tn*6800*, Tn*6913a*, Tn*6913b*, Tn*6914*, and Tn*6915*, 1 transposable prophage, Tn*6964*, 1 IME, Tn*6966*, and 1 ICE, Tn*6967*, and (ii) the remaining 7 were the inner components of the above 12 and comprised 2 composite transposons, Tn*6965* and Tn*6970*, 2 unit transposons, Tn*6971* and Tn*6972*, and 3 integrons, In2-77, In1785, and In1787. Additionally, there were two newly designated (first designated in this study but with previously determined sequences) AGEs: 1 putative resistance unit, IS*CR3*-*ere*(B) unit, and 1 transposable prophage, Tn*6963*. All these 21 newly identified or designated AGEs were first identified in the 166 global *Morganella* isolates. Additionally, Tn*6872*-related IME, Tn*6397*-related ICE, and ARG-harboring Tn*6963-*related transposable prophage were reported for the first time in *Morganella*.

## DISCUSSION

There are few reports on characterizing ARGs in individual *Morganella* isolates or a small collection of *Morganella* isolates (*n* ≤ 22 in each study) (31–33). This study provides a genomic and bioinformatics view of the classification and evolution of *Morganella* species and the global prevalence of AGEs and associated ARGs in *Morganella*, based on a collection of 166 sequenced isolates, including 60 sequenced in this study.

We establish a systematic genome sequence-based species classification scheme for *Morganella* based on ANI analysis plus phylogenomic analysis. The two conventional *Morganella* species, M. morganii and *M. psychrotolerans*, should be redefined as two complexes: M. morganii and *M. psychrotolerans*, which can be further divided into four and two genospecies, respectively. These two complexes display a very long-distance phylogenomic relationship, being consistent with a previous phylogenetic analysis based on seven *Morganella* housekeeping genes (2). Moreover, the six genospecies display the obvious segregation at genome scale between each other, as revealed by ANI analysis and further confirmed by phylogenomic assay. Notably, all these genospecies cannot be distinguished based on the sequence variation of 16S rRNA genes or that of the seven housekeeping genes (2, 34). Isolates of M. morganii genospecies can be mostly frequently recovered from patients, animals, and the environment, accounting for 147 (91.9%) of the total 160 strains studied. Our phylogenomic analysis on these 147 global isolates of M. morganii genospecies shows that this genospecies exhibits a highly clonal population disseminated across at least 16 countries of the five continents (Table S1). These 147 isolates gather at the farthest position from the root in the phylogenetic tree, and therefore M. morganii genospecies represents the latest differentiated clone among all the six genospecies.

Of these 88 acquired ARGs, the most prevalent are tetracycline-resistance *tetAR* genes, which are found in 99 (59.64%) of the 166 isolates studied. Tetracycline antibiotics have been extensively used in treatment of human and animal infections for at least 60 years (35). The long-term use of tetracycline antibiotics promotes the acquisition and dissemination of tetracycline-resistance genes in various bacteria, such as *Acinetobacter*, *Escherichia*, and *Salmonella* (36), and also *Morganella*, here. In addition, the wide prevalence of β-lactam-resistance genes is also observed in *Morganella* isolates (*n* = 58, 34.93%), which might be due to the frequent empirical use of cephalosporins for clinical therapy of M. morganii-induced infections (37).

The 60 isolates sequenced in this study exhibit the highest nonsusceptibility rate (*n* = 40, 66.67%) for levofloxacin and ciprofloxacin. There are three major mechanisms of fluoroquinolone resistance in *Morganella*: (i) acquisition of quinolone-resistance genes *qnr* (12), *qepA* (38), and *aacA4cr* (39), (ii) mutation of type II topoisomerases genes *gyrAB* and *parCE* (40, 41), and (iii) enhancement of proton-dependent active effluxes (42). A total of 31 of these 60 isolates carry acquired *qnr* and/or *aacA4cr* genes, but only 17 of these 31 isolates are nonsusceptible to fluoroquinolones (Table S1). Commonly, the carriage of *qnr*, *qepA*, and *aacA4cr* genes only mediates limited decreased susceptibility to fluoroquinolones but cannot guarantee that the isolates exhibit the fluoroquinolone resistance with a breakpoint of 0.5 μg/mL provided by Clinical and Laboratory Standards Institute (CLSI) (43, 44). It is speculated that the extensive nonsusceptibility for fluoroquinolones in *Morganella* isolates is most likely caused by the combination of the above multiple resistance mechanisms.

The lowest nonsusceptibility rates were observed for meropenem (*n* = 6, 10%) and amikacin (*n* = 3, 5%) in these 60 Chinese isolates. Similar results were found for the 692 *Morganella* isolates from China Antimicrobial Surveillance Network (Table S7). These denote that carbapenems and amikacin will be the most effective antimicrobials against *Morganella* in China. The observed meropenem resistance and amikacin resistance in the above six and three *Morganella* isolates are mediated by the acquisition of carbapenemase gene (*bla*_KPC-2_ [*n* = 4] or *bla*_NDM-1_ [*n* = 2]) and 16S rRNA methyltransferase gene (*rmtB*), respectively. The presence of carbapenemase genes or *rmt* gene can be identified in only 21 of these 166 global *Morganella* isolates (Table S1). Taken together, meropenem resistance or amikacin resistance is still not widely disseminated in *Morganella*.

This study presents the full sequences of 18 AGEs located within *Morganella* chromosomes. Subsequently, a detailed sequence comparison was applied to these 18 AGEs, together with 5 additional prototype AGEs from GenBank. These 23 AGEs could be divided into eight distinct groups: two IS*26*/IS*15DI*-composite transposons, Tn*10* and its three derivatives, Tn*7* and its two derivatives, Tn*1696* and its five derivatives, Tn*6963* and its one derivative, Tn*6872* and its one derivative, Tn*6397* and its one derivative, and Tn*2670* and its one derivative. Eleven of these 23 AGEs each carries a distinct LAM (>29.5 kb) with complex mosaic structure: (i) LAMs of Tn*6799*, T10RE_GN28_, Tn*6913a*, and Tn*6913b* manifest as distinct Tn*2670*-related elements, (ii) those of T7RE_621164_, Tn*6915*, and T1696RE_ZJD581_ contained different MDR regions, (iii) that of Tn*6964* manifests as Tn*6965* integrated with MDR region, (iv) that of Tn*6966* comprises Tn*6971* and MDR region, (v) that of Tn*6967* contains Tn*6972* and MDR region, and (vi) T2670RE_11759_ as a LAM directly integrates into the chromosome. These LAMs are likely assembled from complex transposition and homologous recombination and, notably, comprise various ARLs, including composite/unit transposons, integrons, and putative resistance units, resulting in accumulation of at least 44 ARGs in *Morganella* chromosomes.

In summary, a genomic epidemiology analysis on 166 global sequenced *Morganella* isolates, including 60 sequenced here, was conducted in this study. First, a genome sequence-based species classification scheme for *Morganella* was established, and the two conventional *Morganella* species were redefined as two complexes, which were further divided into four and two genospecies, respectively. Second, the prevalence of acquired ARGs was screened based on genome sequences, demonstrating that at least 88 acquired ARGs are accumulated and disseminated in *Morganella*. Finally, a detailed sequence comparison of eight groups of 23 AGEs (including 18 *Morganella* chromosomal AGEs sequenced in this study) was performed. There are LAMs in 11 of these 23 AGEs, and these LAMs have complex mosaic structures and contain many ARLs and associated ARGs. Integration of these ARG-containing AGEs into *Morganella* chromosomes would contribute to the accumulation and dissemination of ARGs in *Morganella* and enhance the adaption and survival of *Morganella* under complex and diverse antimicrobial selection pressures.

## MATERIALS AND METHODS

### Bacterial isolates and identification.

A total of 60 *Morganella* isolates were collected from 2013 to 2019, including 56 from hospitalized patients in seven Chinese public hospitals and 4 from animals in four Chinese farms (Table S1). The 16S rRNA genes and the carbapenemase genes *bla*_NDM-1_ and *bla*_KPC-2_ were detected as described previously (45). The activity of class A/B/D carbapenemases in bacterial cell extracts was detected by a modified CarbaNP test (45). The bacterial antimicrobial susceptibility was tested using bioMérieux Vitek 2 and interpreted as per the 2020 CLSI guidelines (44).

### Genomic DNA sequencing and sequence assembly and annotation.

All these 60 isolates were subjected to draft-genome sequencing using a paired-end library with an average insert size of 350 bp (ranged from 150 bp to 600 bp) on a HiSeq sequencer (Illumina, CA, USA). In addition, 12 (Table S1) of them were subjected to complete-genome sequencing with a sheared DNA library with an average size of 15 kb (ranged from 10 kb to 20 kb) on a PacBio RSII sequencer (Pacific Biosciences, CA, USA). The quality control analysis of sequencing data was conducted using NanoPack (46) and FastQC (https://www.bioinformatics.babraham.ac.uk/projects/fastqc). Sequence assembly and annotation were performed as described previously (47).

### ANI analysis and phylogenomic analysis.

The pairwise ANI values of *Morganella* genome sequences were calculated using FastANI (48). *Morganella* genome sequences were aligned to the complete chromosome sequence (accession number CP004345) of M. morganii subsp. *morganii* isolate KT used as reference, and the SNPs were identified by Mummer v3.25 (49). All the SNPs in the repetitive DNA regions were identified and filtered by RepeatMasker (http://www.repeatmasker.org/). Homologous recombination at a genome-wide level was predicted using ClonalFrameML (50), followed by removal of all putative recombinant SNP sites. Based on the final recombination-free core SNPs, a maximum-likelihood phylogenetic tree was constructed using RAxML (51) with a bootstrap iteration of 1,000 and displayed using iTOL (https://itol.embl.de).

### *In silico* analysis of prevalence of AGEs.

We collected the core transposition determinants (encoding transposases and their auxiliary factors) of the 17 major AGE groups, which were frequently found in Gram-negative bacteria (19). These 17 groups included IS*26*/IS*15DI*, IS*10*, and IS*1R* (highly associated with composite transposons) and Tn*3*-family (including Tn*3*, Tn*21*, Tn*163*, Tn*4430*, Tn*4651*, and Tn*4401* subfamilies), Tn*7*-family (composed of Tn*7*, Tn*6230*, Tn*552*, Tn*6022*, and Tn*5035* subfamilies), and Tn*554*-family (comprising Tn*554*, Tn*6488*, and Tn*6571* subfamilies) unit transposons, as shown in our unpublished DANMEL database (https://39.100.87.11/danmel_V1.0/index.php). The sequence alignment of these core transposition determinants was conducted on the draft-genome sequences of these 166 *Morganella* isolates, screening for the prevalence of these 17 major group AGEs in *Morganella*.

### Statistical analysis.

The statistical differences for ARGs among reservoirs were tested by Pearson’s χ^2^ test. Statistical computations and figures were plotted with R package v.3.271 (http://www.r-project.org) and visualized with Adobe Illustrator.

### Data availability.

The complete chromosome sequences of the 12 fully sequenced isolates 11759, 516602, ZJC25, 229813, 621164, 715394, GN28, 81703, ZJG944, ZJG812, ZJD581, and 12304 were submitted to GenBank under accession numbers CP059986, CP064054, CP064828, CP043955, CP064829, CP064833, CP064055, CP064830, CP064827, CP064831, CP064826, and CP064832, respectively. The GenBank accession numbers of all the plasmids of these 12 isolates are listed in Table S1. The draft-genome sequences were submitted to GenBank under BioProject PRJNA671578.
