# Supplementary material for: A Genomic and Bioinformatics View of the Classification and Evolution of Morganella Species and Their Chromosomal Accessory Genetic Elements Harboring Antimicrobial Resistance Genes
Source: Microbiol Spectr. 2022 Feb 23;10(1):e02650-21. doi: 10.1128/spectrum.02650-21 (PMC8865565; doi:10.1128/spectrum.02650-21)
Supplement: SUPPLEMENTAL FILE 1 — Supplemental material. Download SPECTRUM02650-21_Supp_1_seq21.pdf, PDF file, 5.8 MB [file spectrum02650-21_supp_1_seq21.pdf]

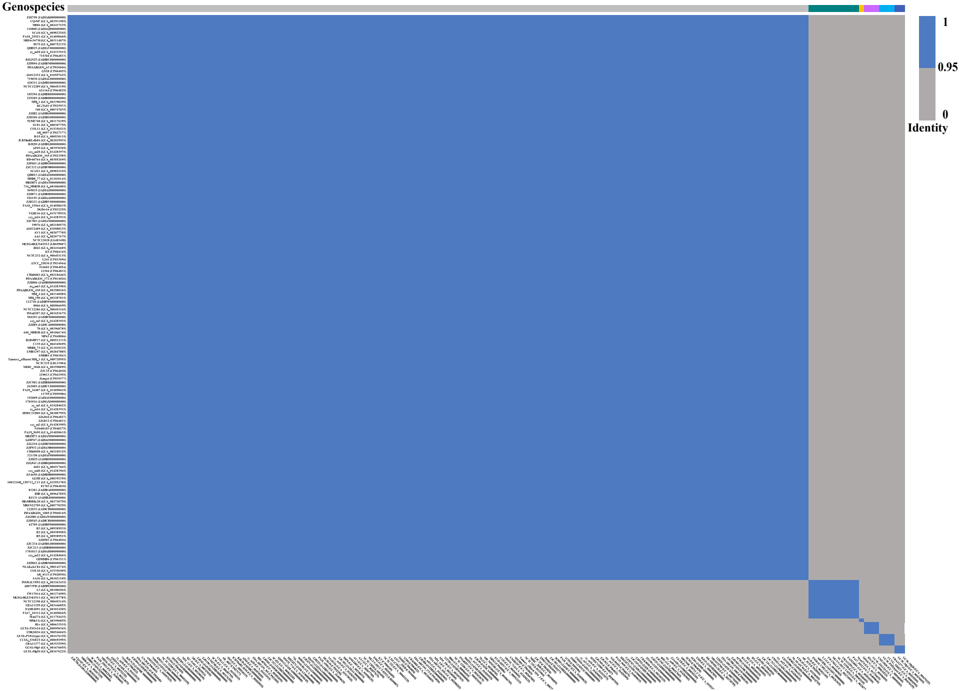

FIG S1 Heatmap of ANI value of *Morganella* isolates. Original data are shown in Table S3.

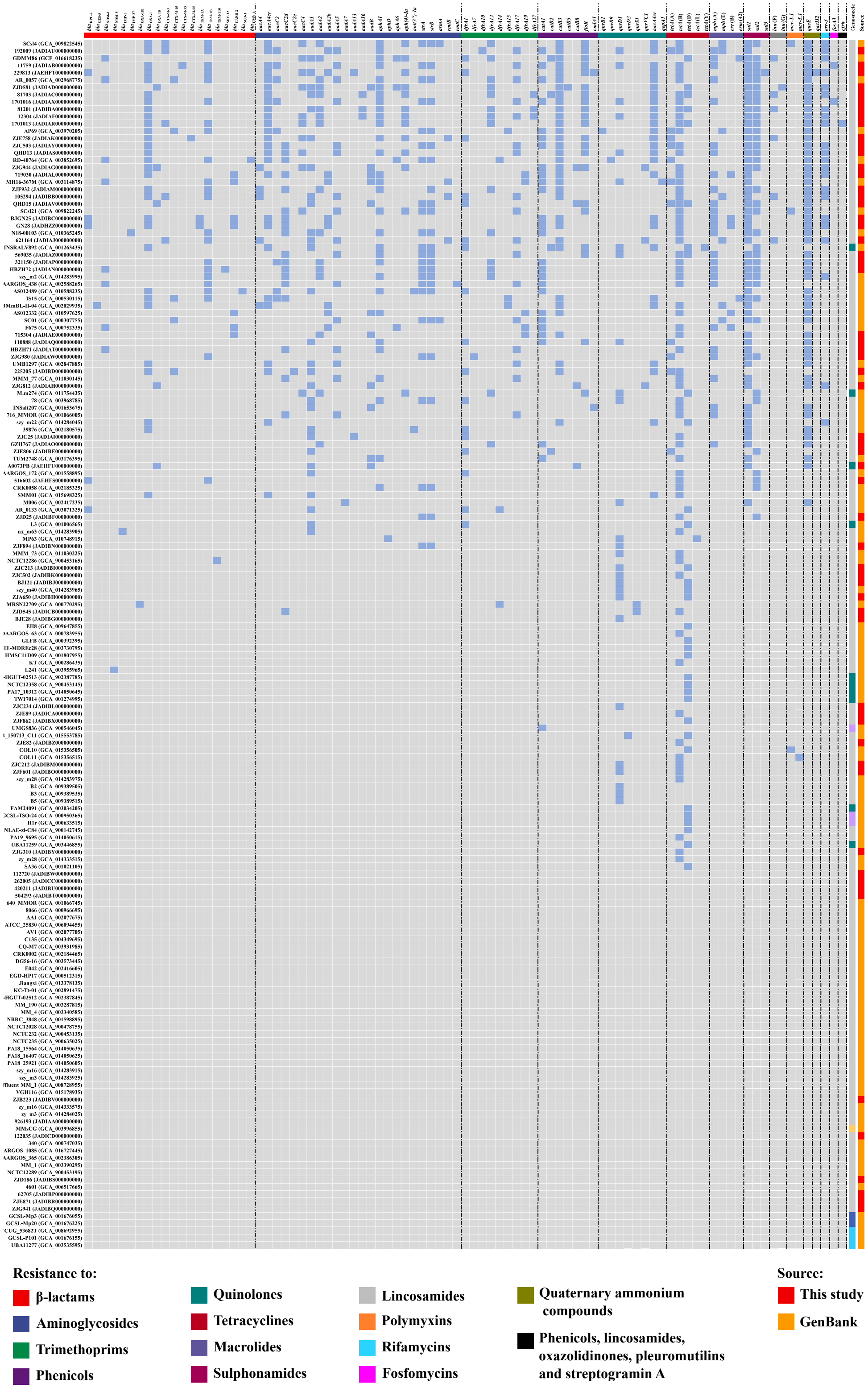

FIG S2 Heatmap of acquired ARGs of *Morganella* isolates. Original data are shown in Table S1.

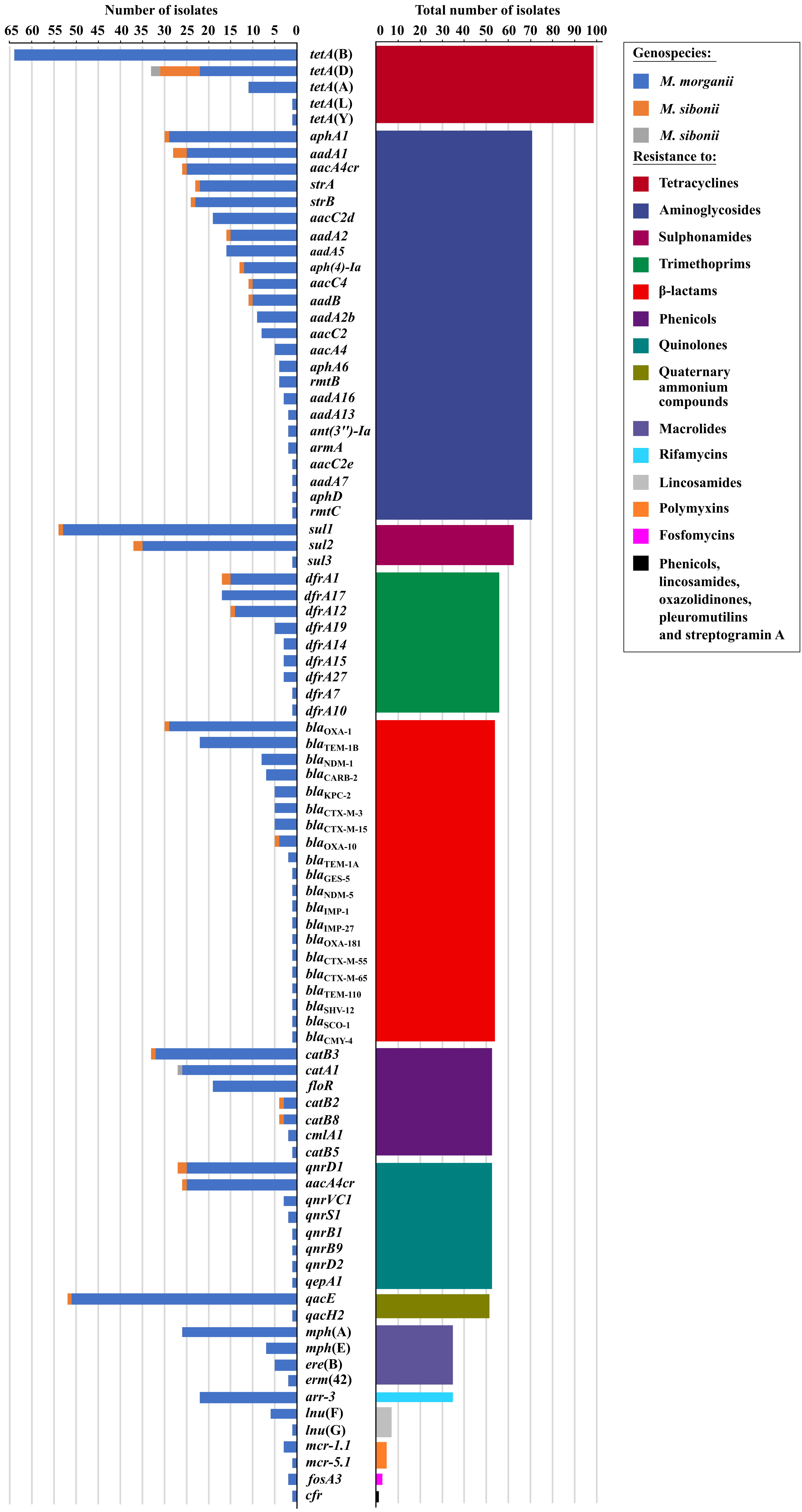

FIG S3 The prevalence of the 88 ARGs in the 166 global *Morganella* isolates. Original data are shown in Table S4.

Table S1. Background information of 166 global *Morganella* isolates

Isolate	Complex	Genospecies	Country	Province	Hospital	Year	Specimen	Host	Source
11759	*Morganella morganii*	*M. morganii*	China	Beijing	H08	2014	Drainage	Homo sapiens	This study
516602	*M. morganii*	*M. morganii*	China	Jiangsu	H07	2017	Drainage	Homo sapiens	This study
ZJC25	*M. morganii*	*M. morganii*	China	Guangdong	H01	2017	Blood	Homo sapiens	This study
229813	*M. morganii*	*M. morganii*	China	Jiangsu	H07	2017	Blood	Homo sapiens	This study
621164	*M. morganii*	*M. morganii*	China	Beijing	H02	2014	Urine	Homo sapiens	This study
715304	*M. morganii*	*M. morganii*	China	Beijing	H02	2015	Secretion	Homo sapiens	This study
GN28	*M. morganii*	*M. morganii*	China	Beijing	H02	2015	Sputum	Homo sapiens	This study
81703	*M. morganii*	*M. morganii*	China	Hebei	H03	2015	Urine	Homo sapiens	This study
ZJG944	*M. morganii*	*M. morganii*	China	Guangdong	H01	2013	Secretion	Homo sapiens	This study
ZJG812	*M. morganii*	*M. morganii*	China	Guangdong	H01	2017		Homo sapiens	This study
ZJD581	*M. morganii*	*M. morganii*	China	Guangdong	H01	2017	Bile	Homo sapiens	This study
12304	*M. morganii*	*M. morganii*	China	Zhejiang	H13	2016	Urine	Homo sapiens	This study

ZJE758	*M. morganii*	*M. morganii*	China	Guangdong	H01	2016	Sputum	Homo sapiens	This study
719030	*M. morganii*	*M. morganii*	China	Beijing	H02	2016		Homo sapiens	This study
ZJF932	*M. morganii*	*M. morganii*	China	Guangdong	H01	2015	Bile	Homo sapiens	This study
HBZH72	*M. morganii*	*M. morganii*	China	Hebei	H06	2017	Urine	Homo sapiens	This study
926193	*M. morganii*	*M. morganii*	China	Beijing	H02	2017	Sputum	Homo sapiens	This study
GZH767	*M. morganii*	*M. morganii*	China	Guangdong	H10	2016	Sputum	Homo sapiens	This study
321150	*M. morganii*	*M. morganii*	China	Beijing	H02	2014	Sputum	Homo sapiens	This study
110888	*M. morganii*	*M. morganii*	China	Guangdong	H04	2018	Secretion	Homo sapiens	This study
1701013	*M. morganii*	*M. morganii*	China	Zhejiang	Animal	2017	Stool	Pig	This study
QHD13	*M. morganii*	*M. morganii*	China	Hebei	H11	2018	Drainage	Homo sapiens	This study
HBZH71	*M. morganii*	*M. morganii*	China	Hebei	H06	2017	Blood	Homo sapiens	This study
192009	*M. morganii*	*M. morganii*	China	Shandong	H12	2017	Secretion	Homo sapiens	This study
QHD15	*M. morganii*	*M. morganii*	China	Hebei	H11	2017	Blood	Homo sapiens	This study
ZJG980	*M. morganii*	*M. morganii*	China	Guangdong	H01	2017	Urine	Homo sapiens	This study
1701016	*M. morganii*	*M. morganii*	China	Zhejiang	Animal	2017	Stool	Pig	This study
ZJC503	*M. morganii*	*M. morganii*	China	Guangdong	H01	2015	Urine	Homo sapiens	This study
569035	*M. morganii*	*M. morganii*	China	Guangdong	H09	2017	Urine	Homo sapiens	This study
A0073PB	*M. morganii*	*M. sibonii*	China	Heilongjiang	Animal	2016	Tissue	Animal	This study
81201	*M. morganii*	*M. morganii*	China	Hebei	H03	2017	Secretion	Homo sapiens	This study
105294	*M. morganii*	*M. morganii*	China	Beijing	H02	2016	Urine	Homo sapiens	This study
BJGN25	*M. morganii*	*M. morganii*	China	Beijing	H02	2015	Sputum	Homo sapiens	This study
225205	*M. morganii*	*M. morganii*	China	Beijing	H02	2016	Urine	Homo sapiens	This study
ZJE806	*M. morganii*	*M. morganii*	China	Guangdong	H01	2016	Urine	Homo sapiens	This study
ZJD25	*M. morganii*	*M. morganii*	China	Guangdong	H01	2015	Urine	Homo sapiens	This study
BJE28	*M. morganii*	*M. morganii*	China	Beijing	H02	2014	Urine	Homo sapiens	This study
ZJA650	*M. morganii*	*M. morganii*	China	Guangdong	H01	2014	Secretion	Homo sapiens	This study

ZJC213	*M. morganii*	*M. morganii*	China	Guangdong	H01	2015	Urine	Homo sapiens	This study
BJ121	*M. morganii*	*M. morganii*	China	Beijing	Animal	2017		Animal	This study
ZJC502	*M. morganii*	*M. morganii*	China	Guangdong	H01	2015	Secretion	Homo sapiens	This study
ZJC234	*M. morganii*	*M. morganii*	China	Guangdong	H01	2015	Urine	Homo sapiens	This study
ZJC212	*M. morganii*	*M. morganii*	China	Guangdong	H01	2015	Urine	Homo sapiens	This study
ZJF894	*M. morganii*	*M. morganii*	China	Guangdong	H01	2017	Blood	Homo sapiens	This study
ZJF601	*M. morganii*	*M. morganii*	China	Guangdong	H01	2016		Homo sapiens	This study
62705	*M. morganii*	*M. morganii*	China	Hebei	H03	2017	Bile	Homo sapiens	This study
ZJG941	*M. morganii*	*M. morganii*	China	Guangdong	H01	2017		Homo sapiens	This study
ZJE871	*M. morganii*	*M. morganii*	China	Guangdong	H01	2016	Urine	Homo sapiens	This study
ZJD186	*M. morganii*	*M. morganii*	China	Guangdong	H01	2015	Urine	Homo sapiens	This study
504293	*M. morganii*	*M. morganii*	China	Beijing	H02	2017	Puncture fluid	Homo sapiens	This study
420211	*M. morganii*	*M. morganii*	China	Beijing	H02	2016	Drainage	Homo sapiens	This study
ZJB223	*M. morganii*	*M. morganii*	China	Guangdong	H01	2014	Urine	Homo sapiens	This study
112720	*M. morganii*	*M. morganii*	China	Guangdong	H04	2018	Sputum	Homo sapiens	This study
ZJF862	*M. morganii*	*M. morganii*	China	Guangdong	H01	2016		Homo sapiens	This study
ZJG310	*M. morganii*	*M. morganii*	China	Guangdong	H01	2017	Urine	Homo sapiens	This study
ZJE82	*M. morganii*	*M. morganii*	China	Guangdong	H01	2015	Urine	Homo sapiens	This study
ZJE89	*M. morganii*	*M. morganii*	China	Guangdong	H01	2015	Secretion	Homo sapiens	This study
ZJD545	*M. morganii*	*M. morganii*	China	Guangdong	H01	2015		Homo sapiens	This study
262005	*M. morganii*	*M. morganii*	China	Henan	H05	2019	Urine	Homo sapiens	This study
122035	*M. morganii*	*M. morganii*	China	Henan	H05	2019	Urine	Homo sapiens	This study
GCSL-Mp3	*M. psychrotolerans*	*M. kristinii*	USA	Alabama		2015		Dolphinfish	GenBank
GCSL-Mp20	*M. psychrotolerans*	*M. kristinii*	USA	Alabama		2015		Dolphinfish	GenBank
UBA11277	*M. psychrotolerans*	*M. psychrotoleransis*							GenBank

CCUG_53682T	*M. psychrotolerans*	*M. psychrotoleransis*	Denmark	Copenhagen			Cold smoked tuna	Environment	GenBank
GCSL-P101	*M. psychrotolerans*	*M. psychrotoleransis*				2004	Cold smoked tuna	Environment	GenBank
MMsCG	*M. morganii*	*M. laugraudii*	New Zealand			2010		Costelytra giveni	GenBank
UMGS836	*M. morganii*	*M. chanii*				2019	Gut	Homo sapiens	GenBank
GCSL-TSO-24	*M. morganii*	*M. chanii*	USA	Alabama		2013	Tuna salad	Environment	GenBank
H1r	*M. morganii*	*M. chanii*	Malaysia				Carnivorous plant phytotelma	Environment	GenBank
M.m274	*M. morganii*	*M. sibonii*							GenBank
PA17_10312	*M. morganii*	*M. sibonii*	Austria	Vienna			Brain	Goose	GenBank
FAM24091	*M. morganii*	*M. sibonii*	Switzerland			2017	Cheese	Environment	GenBank
UBA11259	*M. morganii*	*M. sibonii*							GenBank
NCTC12358	*M. morganii*	*M. sibonii*	United Kingdom			1900	Stool		GenBank
MGYG-HGUT-02513	*M. morganii*	*M. sibonii*	USA				Human gut	Homo sapiens	GenBank
TW17014	*M. morganii*	*M. sibonii*	USA	Michigan		2013	Stool	Homo sapiens	GenBank
L3	*M. morganii*	*M. sibonii*	Malaysia			2013	Lettuce Leaves	Environment	GenBank
INSRALV892	*M. morganii*	*M. sibonii*	Portugal					Animal	GenBank
FDAARGOS_172	*M. morganii*	*M. morganii*	USA	Washington		2014	Urine	Homo sapiens	GenBank
FDAARGOS_365	*M. morganii*	*M. morganii*	USA	Washington		2014	Stool	Homo sapiens	GenBank

KC-Tt-01	*M. morganii*	*M. morganii*	South Korea	Ulsan		2017	Pericardial fluid	Tursiops truncatus	GenBank
FDAARGOS_63	*M. morganii*	*M. morganii*	USA			2013	Wound	Homo sapiens	GenBank
AR_0057	*M. morganii*	*M. morganii*							GenBank
AR_0133	*M. morganii*	*M. morganii*							GenBank
DG56-16	*M. morganii*	*M. morganii*	China	Guangxi		2018	Liver	Crocodile lizard	GenBank
L241	*M. morganii*	*M. morganii*	China	Hangzhou		2016	Stool	Homo sapiens	GenBank
ATCC_25830	*M. morganii*	*M. morganii*							GenBank
Jiangxi	*M. morganii*	*M. morganii*	China	Jiangxi		2016		Misgurnus anguillicaudatus	GenBank
N18-00103	*M. morganii*	*M. morganii*	Canada	Winnipeg		2018	Sputum	Homo sapiens	GenBank
MP63	*M. morganii*	*M. morganii*	China	Jinan		2018	Wastewater	Environment	GenBank
NCTC235	*M. morganii*	*M. morganii*							GenBank
MGYG-HGUT-02512	*M. morganii*	*M. morganii*					Human gut	Homo sapiens	GenBank
NCTC12028	*M. morganii*	*M. morganii*					Stool	Homo sapiens	GenBank
KT	*M. morganii*	*M. morganii*	China	Taiwan		2009	Blood	Homo sapiens	GenBank
MH16-367M	*M. morganii*	*M. morganii*	Viet Nam	Hanoi		2016	Blood	Homo sapiens	GenBank
78	*M. morganii*	*M. morganii*							GenBank
340	*M. morganii*	*M. morganii*	Malaysia			2013	Wound swab	Homo sapiens	GenBank

4601	*M. morganii*	*M. morganii*	Brazil	Sao Paulo		2016	Swab	Homo sapiens	GenBank
8066	*M. morganii*	*M. morganii*						Homo sapiens	GenBank
39876	*M. morganii*	*M. morganii*	USA			2013	Blood	Homo sapiens	GenBank
640_MMOR	*M. morganii*	*M. morganii*	USA	Washington				Homo sapiens	GenBank
716_MMOR	*M. morganii*	*M. morganii*	USA	Washington				Homo sapiens	GenBank
AA1	*M. morganii*	*M. morganii*	USA	Central Florida		2015	Water	Environment	GenBank
AP69	*M. morganii*	*M. morganii*							GenBank
AS012332	*M. morganii*	*M. morganii*	USA			2014	Lung	Homo sapiens	GenBank
AS012489	*M. morganii*	*M. morganii*	USA			2016	Lung	Homo sapiens	GenBank
AV1	*M. morganii*	*M. morganii*	USA	Central Florida		2015	Roots	Pontederia crassipes	GenBank
B2	*M. morganii*	*M. morganii*	Banglade sh	Dhaka		2017		Cattle	GenBank
B3	*M. morganii*	*M. morganii*	Banglade sh	Dhaka		2017		Environment	GenBank
B5	*M. morganii*	*M. morganii*	Banglade sh	Dhaka		2018		Environment	GenBank
C135	*M. morganii*	*M. morganii*	USA			2018		Homo sapiens	GenBank
CQ-M7	*M. morganii*	*M. morganii*	China	Chongqing		2017	Wound	Chinese giant salamander	GenBank
CRK0002	*M. morganii*	*M. morganii*	USA			2015	Blood	Homo sapiens	GenBank
CRK0058	*M. morganii*	*M. morganii*	USA			2013	Blood	Homo sapiens	GenBank
E042	*M. morganii*	*M. morganii*	South Africa	Pretoria		2013	Urine	Homo sapiens	GenBank
EH8	*M. morganii*	*M. morganii*	China	Guangdong		2018		Homo sapiens	GenBank
F675	*M. morganii*	*M. morganii*	France			2014			GenBank

FDAARGOS_438	*M. morganii*	*M. morganii*	Canada	British Columbia		2014	Sputum	Homo sapiens	GenBank
HE-MDREc28	*M. morganii*	*M. morganii*	USA			2014		Homo sapiens	GenBank
ICBMmBL-II-04	*M. morganii*	*M. morganii*	Brazil	Amazon		2013	Rectal swab	Homo sapiens	GenBank
INSali207	*M. morganii*	*M. morganii*	Portugal	Lisbon		2013	Vegetable	Environment	GenBank
IS15	*M. morganii*	*M. morganii*	Austria	Vienna					GenBank
M006	*M. morganii*	*M. morganii*	South Africa	Pretoria		2013	Urine	Homo sapiens	GenBank
MM_1	*M. morganii*	*M. morganii*	Russia	Kazan		2014		Homo sapiens	GenBank
MM_190	*M. morganii*	*M. morganii*	Russia	Kazan		2015	Urine	Homo sapiens	GenBank
MM_4	*M. morganii*	*M. morganii*	Russia	Kazan		2014	Urine	Homo sapiens	GenBank
MMM_73	*M. morganii*	*M. morganii*	Lebanon			2018		Homo sapiens	GenBank
MMM_77	*M. morganii*	*M. morganii*	Lebanon			2019		Homo sapiens	GenBank
MRSN22709	*M. morganii*	*M. morganii*	USA			2014	Wound	Homo sapiens	GenBank
NBRC_3848	*M. morganii*	*M. morganii*							GenBank
NCTC12286	*M. morganii*	*M. morganii*	United Kingdom	Dublin		1900	Urine	Homo sapiens	GenBank
NCTC12289	*M. morganii*	*M. morganii*	United Kingdom	Dublin		1900		Homo sapiens	GenBank
NCTC232	*M. morganii*	*M. morganii*				1900			GenBank
NLAE-zl-C84	*M. morganii*	*M. morganii*							GenBank
nx_m63	*M. morganii*	*M. morganii*	China	Ningxia		2014	Urine	Homo sapiens	GenBank
PA18_15564	*M. morganii*	*M. morganii*	Austria	Vienna			Heart	Goose	GenBank
PA18_16407	*M. morganii*	*M. morganii*	Austria	Vienna			Heart, Liver, Intestine, Lung	Goose	GenBank
PA18_25921	*M. morganii*	*M. morganii*	Austria	Vienna			Heart	Turkey	GenBank

PA19_9695	*M. morganii*	*M. morganii*	Austria	Vienna			Heart	Chicken	GenBank
RD-40764	*M. morganii*	*M. morganii*	France			2018	Blood	Homo sapiens	GenBank
SA36	*M. morganii*	*M. morganii*	Saudi Arabia	Al Qurayyat		2014	Water	Environment	GenBank
SC01	*M. morganii*	*M. morganii*							GenBank
SCsl21	*M. morganii*	*M. morganii*						Pig	GenBank
SCsl4	*M. morganii*	*M. morganii*					Liver	Pig	GenBank
szy_m16	*M. morganii*	*M. morganii*	China	Guangdong		2015		Homo sapiens	GenBank
szy_m2	*M. morganii*	*M. morganii*	China	Guangdong		2014	Sputum	Homo sapiens	GenBank
szy_m22	*M. morganii*	*M. morganii*	China	Guangdong		2011		Homo sapiens	GenBank
szy_m28	*M. morganii*	*M. morganii*	China	Guangdong		2016	Urine	Homo sapiens	GenBank
szy_m3	*M. morganii*	*M. morganii*	China	Guangdong		2015	Blood	Homo sapiens	GenBank
szy_m40	*M. morganii*	*M. morganii*	China	Guangdong		2014	Sputum	Homo sapiens	GenBank
Tannery_effluent_MM_1	*M. morganii*	*M. morganii*	India	Tamil Nadu		2015	Water	Environment	GenBank
TUM2748	*M. morganii*	*M. morganii*				2005		Homo sapiens	GenBank
UMB1297	*M. morganii*	*M. morganii*	USA	Maywood		2015	Urine	Homo sapiens	GenBank
zy_m16	*M. morganii*	*M. morganii*	China	Guangdong		2016	Urine	Homo sapiens	GenBank
zy_m28	*M. morganii*	*M. morganii*	China	Guangdong		2018	Drainage	Homo sapiens	GenBank
zy_m3	*M. morganii*	*M. morganii*	China	Guangdong		2016	Pus	Homo sapiens	GenBank
VGH116	*M. morganii*	*M. morganii*	China	Taiwan		2015	Tissue	Homo sapiens	GenBank
COL11	*M. morganii*	*M. morganii*				2019		Broiler carcass	GenBank
COL10	*M. morganii*	*M. morganii*				2019		Broiler carcass	GenBank
1001216B_150713_C11	*M. morganii*	*M. morganii*	USA			2015	Stool	Homo sapiens	GenBank
SMM01	*M. morganii*	*M. morganii*	India	Puttaparthi		2018	Urine	Homo sapiens	GenBank
GDMM86	*M. morganii*	*M. morganii*	China			2020		Environment	GenBank

FDAARGOS_1085	*M. morganii*	*M. morganii*	Germany	Langen					GenBank
EGD-HP17	*M. morganii*	*M. morganii*	India	Nagpur		2010	Toilet	Environment	GenBank
HMSC11D09	*M. morganii*	*M. morganii*					Urinary tract	Homo sapiens	GenBank
GLFB	*M. morganii*	*M. morganii*	USA	Texas		2011	Soil	Environment	GenBank

Table S1. (continued)

Isolate	Designation of chromosome and plasmids	Complete/draft genome sequence	Accession number	Characterized in this study
11759	c11759	Complete	CP059986	Yes
	p11759-FII	Complete	MZ848139	No
	p11759-1	Complete	MZ848136	No
	p11759-2	Complete	MZ848137	No
	p11759-3	Complete	MZ848138	No
516602	c516602	Complete	CP064054	Yes
	p516602-FII	Complete	MN310367	No
ZJC25	cZJC25	Complete	CP064828	Yes
229813	c229813	Complete	CP043955	Yes
	p229813-FII	Complete	MN310368	No
621164	c621164	Complete	CP064829	Yes
	p621164-FII	Complete	MT701512	No
715304	c715304	Complete	CP064833	Yes
GN28	cGN28	Complete	CP064055	Yes
	pGN28-KPC	Complete	MF156712	No
81703	c81703	Complete	CP064830	Yes
	p81703-FII	Complete	MT679665	No
	p81703-2	Complete	MZ848141	No
ZJG944	cZJG944	Complete	CP064827	Yes
ZJG812	cZJG812	Complete	CP064831	Yes
ZJD581	cZJD581	Complete	CP064826	Yes
12304	c12304	Complete	CP064832	Yes
	p12304-1	Complete	MZ848140	No

ZJE758		Draft	JADIAK000000000	
719030		Draft	JADIAL000000000	
ZJF932		Draft	JADIAM000000000	
HBZH72		Draft	JADIAN000000000	
926193		Draft	JADIAA000000000	
GZH767		Draft	JADIAO000000000	
321150		Draft	JADIAP000000000	
110888		Draft	JADIAQ000000000	
1701013		Draft	JADIAR000000000	
QHD13		Draft	JADIAS000000000	
HBZH71		Draft	JADIAT000000000	
192009		Draft	JADIAU000000000	
QHD15		Draft	JADIAV000000000	
ZJG980		Draft	JADIAW000000000	
1701016		Draft	JADIAX000000000	
ZJC503		Draft	JADIAY000000000	
569035		Draft	JADIAZ000000000	
A0073PB		Draft	JAEHFU000000000	
81201		Draft	JADIBA000000000	
105294		Draft	JADIBB000000000	
BJGN25		Draft	JADIBC000000000	
225205		Draft	JADIBD000000000	
ZJE806		Draft	JADIBE000000000	
ZJD25		Draft	JADIBF000000000	
BJE28		Draft	JADIBG000000000	
ZJA650		Draft	JADIBH000000000	

ZJC213		Draft	JADIBI000000000		
BJ121		Draft	JADIBJ000000000		
ZJC502		Draft	JADIBK000000000		
ZJC234		Draft	JADIBL000000000		
ZJC212		Draft	JADIBM000000000		
ZJF894		Draft	JADIBN000000000		
ZJF601		Draft	JADIBO000000000		
62705		Draft	JADIBP000000000		
ZJG941		Draft	JADIBQ000000000		
ZJE871		Draft	JADIBR000000000		
ZJD186		Draft	JADIBS000000000		
504293		Draft	JADIBT000000000		
420211		Draft	JADIBU000000000		
ZJB223		Draft	JADIBV000000000		
112720		Draft	JADIBW000000000		
ZJF862		Draft	JADIBX000000000		
ZJG310		Draft	JADIBY000000000		
ZJE82		Draft	JADIBZ000000000		
ZJE89		Draft	JADICA000000000		
ZJD545		Draft	JADICB000000000		
262005		Draft	JADICC000000000		
122035		Draft	JADICD000000000		
GCSL-Mp3		Draft	GCA_001676055		
GCSL-Mp20		Draft	GCA_001676225		
UBA11277		Draft	GCA_003535595		
CCUG_53682T		Draft	GCA_008692955		

GCSL-P101		Draft	GCA_001676155	
MMsCG		Draft	GCA_003996855	
UMGS836		Draft	GCA_900546045	
GCSL-TSO-24		Draft	GCA_000950365	
H1r		Draft	GCA_000633515	
M.m274		Draft	GCA_011754435	
PA17_10312		Draft	GCA_014050645	
FAM24091		Draft	GCA_003034205	
UBA11259		Draft	GCA_003446855	
NCTC12358		Draft	GCA_900453145	
MGYG-HGUT-02513		Draft	GCA_902387785	
TW17014		Draft	GCA_001274995	
L3		Draft	GCA_001006565	
INSRALV892		Draft	GCA_001263435	
FDAARGOS_172		Complete	CP014026	
FDAARGOS_365		Complete	CP023505	
		Complete	CP023506	
KC-Tt-01		Complete	CP025933	
FDAARGOS_63		Complete	CP026046	
AR_0057		Complete	CP027177	
AR_0133		Complete	CP028956	
		Complete	CP028957	
		Complete	CP028958	
DG56-16		Complete	CP032295	
L241		Complete	CP033056	
		Complete	CP034944	

ATCC_25830		Complete	CP039377	
Jiangxi		Complete	CP048275	
N18-00103		Complete	CP048806	
MP63		Complete	CP048807	
		Complete	CP048808	
		Complete	CP048809	
NCTC235		Complete	LR133904	
MGYG-HGUT-02512		Complete	LR699007	
NCTC12028		Complete	LS483498	
KT		Complete	CP004345	
MH16-367M		Draft	GCA_003114875	
78		Draft	GCA_003968785	
340		Draft	GCA_000747035	
4601		Draft	GCA_006517665	
8066		Draft	GCA_000966695	
39876		Draft	GCA_002180575	
640_MMOR		Draft	GCA_001066745	
716_MMOR		Draft	GCA_001066005	
AA1		Draft	GCA_002077675	
AP69		Draft	GCA_003970205	
AS012332		Draft	GCA_010597625	
AS012489		Draft	GCA_010588235	
AV1		Draft	GCA_002077705	
B2		Draft	GCA_009389505	
B3		Draft	GCA_009389535	
B5		Draft	GCA_009389515	

C135		Draft	GCA_004349695		
CQ-M7		Draft	GCA_003931985		
CRK0002		Draft	GCA_002184465		
CRK0058		Draft	GCA_002185325		
E042		Draft	GCA_002416605		
EH8		Draft	GCA_009647855		
F675		Draft	GCA_000752335		
FDAARGOS_438		Draft	GCA_002588265		
HE-MDREc28		Draft	GCA_003730795		
ICBMmBL-II-04		Draft	GCA_002029935		
INSali207		Draft	GCA_001653675		
IS15		Draft	GCA_000530115		
M006		Draft	GCA_002417235		
MM_1		Draft	GCA_003390295		
MM_190		Draft	GCA_003287815		
MM_4		Draft	GCA_003340585		
MMM_73		Draft	GCA_011030225		
MMM_77		Draft	GCA_011030145		
MRSN22709		Draft	GCA_000770295		
NBRC_3848		Draft	GCA_001598895		
NCTC12286		Draft	GCA_900453165		
NCTC12289		Draft	GCA_900453195		
NCTC232		Draft	GCA_900453135		
NLAE-zl-C84		Draft	GCA_900142745		
nx_m63		Draft	GCA_014283905		
PA18_15564		Draft	GCA_014050635		

PA18_16407		Draft	GCA_014050625	
PA18_25921		Draft	GCA_014050605	
PA19_9695		Draft	GCA_014050615	
RD-40764		Draft	GCA_003852695	
SA36		Draft	GCA_001021105	
SC01		Draft	GCA_000307755	
SCsl21		Draft	GCA_009822245	
SCsl4		Draft	GCA_009822545	
szy_m16		Draft	GCA_014283915	
szy_m2		Draft	GCA_014283995	
szy_m22		Draft	GCA_014284045	
szy_m28		Draft	GCA_014283975	
szy_m3		Draft	GCA_014283925	
szy_m40		Draft	GCA_014283965	
Tannery_effluent_MM_1		Draft	GCA_008728955	
TUM2748		Draft	GCA_003176395	
UMB1297		Draft	GCA_002847885	
zy_m16		Draft	GCA_014333575	
zy_m28		Draft	GCA_014333515	
zy_m3		Draft	GCA_014284025	
VGH116		Draft	GCA_015178935	
COL11		Draft	GCA_015356515	
COL10		Draft	GCA_015356505	
1001216B_150713_C11		Draft	GCA_015553785	
SMM01		Complete	CP063843	
GDMM86		Complete	CP061513	

FDAARGOS_1085		Complete	CP068145	
EGD-HP17		Draft	GCA_000512315	
HMSC11D09		Draft	GCA_001807955	
GLFB		Draft	GCA_000392395	

Table S1. (continued)

Isolate	Illumina Sequencing									
	**Sequencing**	**Raw Paired Reads Number**	**Clean Paired Reads Number**	**Q20 (%)**	**Q30 (%)**	**Depth (X)**	**Assembled bases (bp)**	**Numbers of contigs**	**Contig N50 (bp)**	**%GC content**
11759	Yes	7,525,764	7,517,330	97.39	92.89	294.21	3,891,811	61	382,213	50.86
516602	Yes	11,031,842	11,020,716	98.04	94.30	401.94	4,111,694	53	289,094	50.90
ZJC25	Yes	6,268,900	6,258,736	97.38	92.85	243.47	3,853,809	66	365,199	51.06
229813	Yes	11,225,004	111,214,303	98.03	90.33	422.33	3,986,751	44	265,855	51.10
621164	Yes	7,560,200	7,550,212	97.43	93.00	287.71	3,962,521	75	210,735	50.94
715304	Yes	9,144,120	9,133,670	97.47	93.04	350.15	3,922,960	53	309,918	51.03
GN28	Yes	9,461,830	9,442,160	97.31	92.71	348.82	4,088,144	58	192,516	50.82
81703	Yes	7,735,288	7,718,544	97.43	92.98	287.51	4,053,172	61	279,783	50.95
ZJG944	Yes	8,626,725	8,616,686	97.41	92.93	334.08	3,877,419	45	404,811	51.23
ZJG812	Yes	9,188,892	9,177,074	97.50	93.12	368.34	3,738,509	28	521,960	51.23
ZJD581	Yes	10,713,104	10,701,784	97.46	92.99	399.61	4,034,186	71	216,437	50.92

12304	Yes	8,490,336	8,478,714	97.32	92.74	324.99	3,912,969	39	363,338	51.13
ZJE758	Yes	7,941,630	7,932,526	97.47	93.01	295.49	4,061,842	61	401,213	50.97
719030	Yes	8,169,396	8,158,672	97.44	92.98	296.05	4,153,091	57	319,905	50.80
ZJF932	Yes	9,844,586	9,826,382	97.50	93.09	376.68	3,921,092	70	269,460	51.13
HBZH72	Yes	11,313,446	11,291,474	96.86	91.67	417.36	4,079,124	87	382,012	51.12
926193	Yes	7,460,618	7,446,142	97.36	92.81	301.12	3,712,444	18	2,520,020	51.15
GZH767	Yes	9,140,028	9,122,700	97.47	93.04	360.47	3,804,251	30	685,378	51.13
321150	Yes	10,436,438	10,421,228	97.32	92.71	394.76	3,991,062	66	618,294	50.87
110888	Yes	7,963,334	7,952,312	97.54	93.20	302.08	3,970,629	40	617,441	51.02
1701013	Yes	10,954,234	10,934,920	97.30	92.70	407.09	4,043,026	61	277,342	50.50
QHD13	Yes	9,653,710	9,644,396	97.09	91.97	374.55	3,894,538	46	249,110	51.03
HBZH71	Yes	9,604,430	9,591,700	97.38	92.83	370.03	3,897,731	32	597,958	51.01
192009	Yes	8,977,548	8,964,722	97.54	93.15	342.89	3,948,779	84	487,276	50.96
QHD15	Yes	10,288,848	10,280,072	96.78	91.32	373.88	4,141,015	92	371,702	50.98
ZJG980	Yes	8,113,782	8,101,606	97.44	92.99	313.15	3,900,939	23	682,563	51.09
1701016	Yes	8,258,552	8,249,042	97.49	93.07	318.50	3,922,853	50	402,560	50.91
ZJC503	Yes	6,864,726	6,855,594	97.46	93.02	273.91	3,768,364	41	694,669	51.19
569035	Yes	7,855,848	7,847,444	97.49	93.09	304.38	3,890,816	42	388,332	51.00
A0073PB	Yes	7,997,726	7,988,532	97.41	92.94	286.58	4,174,237	25	601,182	50.47
81201	Yes	9,600,210	9,582,478	97.40	92.94	357.62	4,053,450	61	279,783	50.96
105294	Yes	7,640,064	7,631,672	97.44	92.95	296.46	3,899,596	71	210,738	51.02
BJGN25	Yes	8,489,766	8,477,180	97.30	92.70	312.86	4,087,923	58	215,387	50.82
225205	Yes	8,736,742	8,726,904	97.38	92.88	324.72	4,049,189	74	143,009	51.03
ZJE806	Yes	7,406,888	7,396,280	97.42	92.96	277.90	3,992,412	58	322,694	50.95

ZJD25	Yes	8,796,930	8,783,578	97.35	92.79	343.26	3,846,229	36	647,518	51.10
BJE28	Yes	8,297,444	8,285,636	97.39	92.89	323.93	3,848,226	29	397,853	51.04
ZJA650	Yes	9,430,006	9,418,152	97.49	93.09	347.75	4,087,076	51	523,414	51.00
ZJC213	Yes	8,101,286	8,092,567	97.52	93.11	302.92	4,030,402	54	312,096	50.92
BJ121	Yes	6,983,242	6,972,014	97.46	93.01	271.20	3,868,452	31	2,130,594	51.11
ZJC502	Yes	7,621,272	7,611,616	97.41	92.95	301.39	3,800,489	29	643,067	51.13
ZJC234	Yes	10,128,844	10,116,220	97.46	93.05	378.25	4,030,114	45	314,743	50.75
ZJC212	Yes	6,832,636	6,824,200	97.24	92.46	261.61	3,941,434	50	303,624	50.79
ZJF894	Yes	10,502,584	10,489,992	97.42	92.93	401.08	3,927,847	31	633,741	51.03
ZJF601	Yes	7,471,604	7,461,342	97.49	93.05	275.14	4,071,859	80	404,341	50.88
62705	Yes	8,952,944	8,938,918	97.46	93.03	370.51	3,618,144	16	1,834,809	51.24
ZJG941	Yes	9,065,456	9,054,268	97.48	93.05	350.17	3,878,188	30	636,681	51.05
ZJE871	Yes	8,413,028	8,404,844	97.91	93.84	334.33	3,766,798	21	949,773	51.08
ZJD186	Yes	9,693,080	9,679,664	97.51	93.11	363.61	3,992,639	47	403,382	51.07
504293	Yes	8,659,348	8,647,378	97.36	92.80	340.82	3,807,041	32	1,093,246	51.00
420211	Yes	6,291,040	6,282,820	97.54	93.15	233.30	4,057,531	38	650,544	50.86
ZJB223	Yes	11,033,560	11,008,490	97.49	93.07	431.36	3,827,888	28	629,790	50.90
112720	Yes	11,231,236	11,218,932	97.48	93.03	451.64	3,737,320	26	629,962	51.04
ZJF862	Yes	9,203,378	9,191,070	97.46	93.00	327.26	4,220,933	46	313,750	50.72
ZJG310	Yes	10,537,020	10,516,604	97.44	92.99	418.08	3,786,643	32	406,825	51.04
ZJE82	Yes	7,548,644	7,539,344	97.47	92.99	300.29	3,770,970	19	1,090,638	51.13
ZJE89	Yes	7,681,048	7,668,278	97.53	93.16	300.48	3,822,173	28	555,623	51.00
ZJD545	Yes	7,433,442	7,423,322	97.46	93.06	289.65	3,855,699	45	553,088	50.94
262005	Yes	14,146,806	14,127,840	98.62	95.70	562.08	3,775,739	22	19,309	51.08
122035	Yes	10,115,682	10,103,220	98.39	95.18	393.63	3,842,112	39	760,723	51.07

Table S1. (continued)

Isolate	PacBio Sequencing								
	**Sequencing**	**Number of Reads**	**Mean Read Length (bp)**	**Number of Bases (bp)**	**Reads N50 (bp)**	**Depth (X)**	**Q20 (%)**	**Q30 (%)**	**Assembled bases (bp)**
11759	Yes	778,066	13096	835,825,800	15,076	212.90	97.27	92.39	3,842,715
									38,408
									33,731
									2,683
									8,439
516602	Yes	668,741	11532	612,043,650	15,312				4,125,669
						146.00	96.02	90.01	52,180
ZJC25	Yes	670,768	12794	734,112,450	14,744	186.24	96.71	91.28	3,941,832
229813	Yes	557,014	12365	701,890,350	14,563				3,982,591
						174.00	95.40	88.66	50,842
621164	Yes	473,424	11895	377,503,500	13,275	93.84	97.96	93.93	3,968,418
									54,395
715304	Yes	865,036	12699	965,676,600	14,586	239.77	97.39	92.69	4,027,489
GN28	Yes	1,057,836	14011	371,239,050	17,160	92.17	98.11	94.19	4,094,154
									46,193
81703	Yes	435,754	12087	773,486,700	13,852	188.64	98.09	94.35	4,018,641
									73,394
									8,282
ZJG944	Yes	389,320	12934	752,676,000	15,028	190.09	97.32	92.55	3,959,489
ZJG812	Yes	1,697,344	11388	645,959,400	12,819	169.73	97.35	92.51	3,805,695
ZJD581	Yes	768,126	12535	840,603,450	14,386	205.79	97.38	92.65	4,084,708
12304	Yes	976,058	11765	705,615,750	13,251	178.48	97.37	92.56	3,950,614
									2,671

Table S1. (continued)

Isolate	Trehalose utilization operon	Carbapenemase activity (Ambler class)
	treRBP	
11759		-
516602		A
ZJC25		-
229813		A
621164		-
715304		-
GN28		A
81703		-
ZJG944		-
ZJG812		-
ZJD581		-
12304		-

ZJE758		-
719030		-
ZJF932		-
HBZH72		B
926193		-
GZH767		-
321150		-
110888		-
1701013		-
QHD13		-
HBZH71		B
192009		-
QHD15		-
ZJG980		-
1701016		-
ZJC503		-
569035		-
A0073PB	+	-
81201		-
105294		-
BJGN25		-
225205		-
ZJE806		-
ZJD25		-
BJE28		-
ZJA650		-

ZJC213		-
BJ121		-
ZJC502		-
ZJC234		-
ZJC212		-
ZJF894		-
ZJF601		-
62705		-
ZJG941		-
ZJE871		-
ZJD186		-
504293		-
420211		-
ZJB223		-
112720		-
ZJF862		-
ZJG310		-
ZJE82		-
ZJE89		-
ZJD545		-
262005		-
122035		-
GCSL-Mp3		
GCSL-Mp20		
UBA11277		
CCUG_53682T		

GCSL-P101		
MMsCG	+	
UMGS836	+	
GCSL-TSO-24	+	
H1r	+	
M.m274	+	
PA17_10312	+	
FAM24091	+	
UBA11259	+	
NCTC12358	+	
MGYG-HGUT-02513	+	
TW17014	+	
L3	+	
INSRALV892	+	
FDAARGOS_172		
FDAARGOS_365		
KC-Tt-01		
FDAARGOS_63		
AR_0057		
AR_0133		
DG56-16		
L241		
ATCC_25830		
Jiangxi		

N18-00103		
MP63		
NCTC235		
MGYG-HGUT-02512		
NCTC12028		
KT		
MH16-367M		
78		
340		
4601		
8066		
39876		
640_MMOR		
716_MMOR		
AA1		
AP69		
AS012332		
AS012489		
AV1		
B2		
B3		
B5		
C135		
CQ-M7		
CRK0002		

CRK0058		
E042		
EH8		
F675		
FDAARGOS_438		
HE-MDREc28		
ICBMmBL-II-04		
INSali207		
IS15		
M006		
MM_1		
MM_190		
MM_4		
MMM_73		
MMM_77		
MRSN22709		
NBRC_3848		
NCTC12286		
NCTC12289		
NCTC232		
NLAE-zl-C84		
nx_m63		
PA18_15564		
PA18_16407		
PA18_25921		
PA19_9695		

RD-40764		
SA36		
SC01		
SCsl21		
SCsl4		
szy_m16		
szy_m2		
szy_m22		
szy_m28		
szy_m3		
szy_m40		
Tannery_effluent_MM_1		
TUM2748		
UMB1297		
zy_m16		
zy_m28		
zy_m3		
VGH116		
COL11		
COL10		
1001216B_150713_C11		
SMM01		
GDMM86		
FDAARGOS_1085		
EGD-HP17		
HMSC11D09		

GLFB		

Table S1. (continued)

Isolate	Acquired antimicrobial resistance genes						
	**β-lactams**	**Aminoglycosides**	**Trimethoprims**	**Phenicols**	**Quinolones**	**Tetracyclines**	**Macrolides**
11759	*bla*$_{\text{TEM-1B}}$, *bla*$_{\text{OXA-1}}$, *bla*$_{\text{CTX-M-55}}$	*aadA5, strA, strB, aacC2, aacA4cr, aphA1*	*dfrA17*	*catA1, catB3, floR*	*aacA4cr, qnrD1*	*tetA*(B)	*mph*(A)
516602	*bla*$_{\text{KPC-2}}$, *bla*$_{\text{TEM-1B}}$					*tetA*(B)	
ZJC25		*aadA1, aadA13*	*dfrA1*			*tetA*(B)	
229813	*bla*$_{\text{KPC-2}}$, *bla*$_{\text{OXA-1}}$, *bla*$_{\text{TEM-1B}}$	*aadA1, aadA13, aacA4cr, aadA2b, aacC4, aph(4)-Ia*	*dfrA1*	*floR, catB3, cmlA1*	*aacA4cr*	*tetA*(B)	
621164	*bla*$_{\text{CTX-M-3}}$, *bla*$_{\text{OXA-1}}$, *bla*$_{\text{TEM-1B}}$	*aadA1, aadA5, aacA4, rmtB*	*dfrA1, dfrA17*	*catB3*	*qnrVC1*	*tetA*(B)	*mph*(E)
715304	*bla*$_{\text{CARB-2}}$	*aadA2, aadB*	*dfrA19*	*catA1, catB3*		*tetA*(B)	
GN28	*bla*$_{\text{KPC-2}}$, *bla*$_{\text{TEM-1A}}$, *bla*$_{\text{OXA-1}}$, *bla*$_{\text{CARB-2}}$	*aacC2d, aacA4cr, aadA2b*		*catA1, catB3*	*aacA4cr*	*tetA*(B)	*ere*(B), *mph*(A)
81703	*bla*$_{\text{OXA-1}}$	*aadA1, aadA2, aacA4cr, aadA16, aphA1, aph(4)-Ia*	*dfrA12, dfrA27*	*floR, catB2, catB3*	*aacA4cr*	*tetA*(B), *tetA*(D)	
ZJG944	*bla*$_{\text{OXA-10}}$, *bla*$_{\text{OXA-1}}$	*aadA1, aacA4, aadB, aacC4, aph(4)-Ia*		*floR, catA1, catB8, catB3*	*qnrVC1*	*tetA*(B)	
ZJG812	*bla*$_{\text{OXA-10}}$	*aadA1, aadB*		*catA1, catB8*	*qnrVC1*	*tetA*(B)	
ZJD581	*bla*$_{\text{OXA-1}}$, *bla*$_{\text{OXA-10}}$	*aadA2, aacA4cr, aadA1, aadB, aacC4, aphA1, aphA6, aph(4)-Ia*	*dfrA12*	*floR, catB3, catB5*	*aacA4cr*	*tetA*(D)	

12304	*bla*$_{\text{OXA-1}}$	*aadA16*, *aadA2*, *aphA1*, *aph(4)-Ia*, *aacC4*, *aacA4cr*	*dfrA12*, *dfrA17*, *dfrA27*	*floR*, *catB3*	*aacA4cr*	*tetA*(B)	
					qnrD1		
ZJE758	*bla*$_{\text{CTX-M-65}}$, *bla*$_{\text{OXA-1}}$	*aadA1*, *aacC4*, *aph(4)-Ia*, *aacA4cr*, *aphA1*, *rmtB*	*dfrA1*	*floR*, *catA1*, *catB3*	*aacA4cr*	*tetA*(A)	
719030	*bla*$_{\text{OXA-1}}$, *bla*$_{\text{CARB-2}}$	*aadA2b*, *aacC2d*, *aadB*, *aphA1*, *aacA4cr*	*dfrA19*	*catA1*, *catB3*	*aacA4cr*	*tetA*(B)	*ere*(B), *mph*(A)
ZJF932	*bla*$_{\text{TEM-1B}}$, *bla*$_{\text{OXA-1}}$	*aadA2*, *aacA4*, *aphA1*, *strB*, *aacC2d*, *aphA1*	*dfrA12*	*catA1*, *catB3*		*tetA*(B)	*mph*(A)
HBZH72	*bla*$_{\text{NDM-1}}$, *bla*$_{\text{SHV-12}}$, *bla*$_{\text{TEM-1B}}$	*aadA2*, *strA*, *strB*, *aacC2d*	*dfrA12*	*catA1*		*tetA*(B)	*mph*(A)
926193							
GZH767		*aadA2*	*dfrA12*	*catA1*		*tetA*(B)	*mph*(A)
321150	*bla*$_{\text{TEM-1B}}$	*aadA2*, *strA*, *strB*, *aacC2d*, *aphA1*, *aacC2*	*dfrA12*	*catA1*		*tetA*(B)	*mph*(A)
110888		*aadA1*, *aphA1*	*dfrA1*	*floR*, *catA1*	*qnrD1*	*tetA*(A)	
1701013	*bla*$_{\text{TEM-1B}}$, *bla*$_{\text{CTX-M-3}}$, *bla*$_{\text{OXA-1}}$	*aadA2*, *aacA4cr*, *aacC4*, *aphA1*, *aph(4)-Ia*	*dfrA12*	*floR*, *catB3*	*aacA4cr*	*tetA*(B), *tetA*(D)	
QHD13	*bla*$_{\text{OXA-1}}$	*aadA5*, *aacA4cr*, *aph(4)-Ia*, *aacC4*, *aacC2d*, *aphA1*	*dfrA17*	*floR*, *catB3*	*qnrD1*, *aacA4cr*	*tetA*(B)	*mph*(A)
HBZH71	*bla*$_{\text{NDM-1}}$	*aadA5*, *aacC2d*, *aphA1*	*dfrA17*			*tetA*(B)	*mph*(A)
192009	*bla*$_{\text{TEM-1B}}$, *bla*$_{\text{CTX-M-3}}$, *bla*$_{\text{OXA-1}}$	*aacC2*, *aadA2b*, *aphA1*, *aacA4cr*, *aadA5*	*dfrA10*, *dfrA17*	*floR*, *catA1*, *catB3*	*qnrD1*, *aacA4cr*	*tetA*(A), *tetA*(B)	*mph*(A), *mph*(E)
QHD15	*bla*$_{\text{OXA-10}}$	*aadA1*, *aadB*, *strA*, *strB*	*dfrA1*	*floR*, *catB8*		*tetA*(A), *tetA*(B), *tetA*(D)	*mph*(A)

ZJG980	$bla_{\text{TEM-1B}}$	*strA*, *strB*	*dfrA7*			*tetA*(A)	*mph*(A)
1701016	$bla_{\text{TEM-1B}}$, $bla_{\text{OXA-1}}$	*aadA5*, *aphA1*, *aacC2*, *aacA4cr*, *strA*, *strB*	*dfrA17*	*floR*, *catA1*, *catB3*	*qnrD1*, *aacA4cr*	*tetA*(B)	*mph*(A)
ZJC503	$bla_{\text{OXA-1}}$	*aadA5*, *aacC2d*, *aacA4cr*, *strA*, *strB*	*dfrA17*	*floR*, *catA1*, *catB3*	*aacA4cr*	*tetA*(A), *tetA*(B)	*mph*(A)
569035		*aadA5*, *strA*, *strB*, *aacC2d*, *aphA1*	*dfrA17*	*floR*	*qnrD1*	*tetA*(B), *tetA*(D)	*mph*(A)
A0073PB	$bla_{\text{OXA-10}}$	*aadA1*, *aadB*		*catB8*			
81201	$bla_{\text{OXA-1}}$	*aadA1*, *aadA2*, *aacA4cr*, *aadA16*, *aphA1*, *aph(4)-Ia*, *aacA4cr*	*dfrA12*, *dfrA27*	*floR*, *catB2*, *catB3*	*aacA4cr*	*tetA*(B), *tetA*(D)	
105294	$bla_{\text{CTX-M-3}}$, $bla_{\text{TEM-1B}}$, $bla_{\text{OXA-1}}$	*aadA1*, *aadA5*, *aacA4*, *rmtB*	*dfrA1*, *dfrA17*	*catB3*		*tetA*(B)	*mph*(E)
BJGN25	$bla_{\text{KPC-2}}$, $bla_{\text{TEM-1A}}$, $bla_{\text{CARB-2}}$, $bla_{\text{OXA-1}}$	*aadA2b*, *aacA4cr*, *aacC2d*		*catA1*, *catB3*	*aacA4cr*	*tetA*(B)	*ere*(B), *mph*(A)
225205	$bla_{\text{CTX-M-15}}$, $bla_{\text{OXA-1}}$	*aadA1*, *aacA4cr*, *aacC2e*	*dfrA1*	*catB3*	*aacA4cr*	*tetA*(A), *tetA*(B)	
ZJE806		*aadA1*	*dfrA1*	*catB2*		*tetA*(A)	
ZJD25		*strA*, *strB*				*tetA*(B)	
BJE28					*qnrD1*		
ZJA650					*qnrD1*	*tetA*(D)	
ZJC213					*qnrD1*	*tetA*(D)	
BJ121					*qnrD1*	*tetA*(D)	
ZJC502					*qnrD1*	*tetA*(B)	
ZJC234					*qnrD1*		
ZJC212					*qnrD1*	*tetA*(B)	
ZJF894		*strA*, *strB*			*qnrD1*		
ZJF601					*qnrD1*	*tetA*(B)	

62705							
ZJG941							
ZJE871							
ZJD186							
504293							
420211							
ZJB223							
112720							
ZJF862						*tetA*(D)	
ZJG310						*tetA*(B)	
ZJE82						*tetA*(B)	
ZJE89						*tetA*(B)	
ZJD545		*aacC2d*			*qnrS1*	*tetA*(B)	
262005							
122035							
GCSL-Mp3							
GCSL-Mp20							
UBA11277							
CCUG_53682 T							
GCSL-P101							
MMsCG							
UMGS836							
GCSL-TSO-24						*tetA*(D)	
H1r						*tetA*(D)	
M.m274		*aacC4, aadA2, aph(4)-Ia*	*dfrA12*	*floR*	*qnrD1*	*tetA*(D)	

PA17_10312							*tetA*(D)	
FAM24091							*tetA*(D)	
UBA11259							*tetA*(D)	
NCTC12358							*tetA*(D)	
MGYG-HGUT-02513							*tetA*(D)	
TW17014							*tetA*(D)	
L3		*aadA1*	*dfrA1*			*tetA*(D)	
INSRALV892	$bla_{\text{OXA-1}}$	*strA, strB, aacA4cr, aadA1, aphA1*	*dfrA1*	*floR, catB2, catB3*	*qnrD1, aacA4cr*	*tetA*(Y), *tetA*(D)	
FDAARGOS_172		*aadA1*	*dfrA1*			*tetA*(B)	
FDAARGOS_365								
KC-Tt-01								
FDAARGOS_63							*tetA*(B)	
AR_0057	$bla_{\text{NDM-1}}$, $bla_{\text{OXA-1}}$, $bla_{\text{CTX-M-15}}$, $bla_{\text{TEM-1B}}$	*aacC2, aacA4cr, aphA1, strA, strB, aadA2, aadA5*	*dfrA12, dfrA17*	*catA1, catB3*	*qnrD1, aacA4cr*	*tetA*(B)	*mph*(A)
AR_0133		*aadA1*	*dfrA1*			*tetA*(D)	
	$bla_{\text{KPC-2}}$		*dfrA14*			*tetA*(D)	
DG56-16								
L241	$bla_{\text{NDM-5}}$							
ATCC_25830								

Jiangxi	*bla*$_{\text{IMP-27}}$, *bla*$_{\text{TEM-1B}}$	*aphA1, aadA1, aadA2, strA, aacC2d, strB*	*dfrA12*	*catA1*		*tetA*(B)	*mph*(A)
N18-00103		*aphD*			*qnrD1*	*tetA*(L)	
MP63							
NCTC235							
MGYG-HGUT-02512							
NCTC12028							
KT						*tetA*(B)	
MH16-367M	*bla*$_{\text{NDM-1}}$, *bla*$_{\text{TEM-1B}}$, *bla*$_{\text{CARB-2}}$	*aadA2b, aadB, rmtB, aphA1*	*dfrA19*	*catA1, catB3*	*qepA1, qnrD1*	*tetA*(A), *tetA*(B)	
78		*strB, aadA1, aphA1, strA*	*dfrA1*		*qnrD1*	*tetA*(B)	
340							
4601							
8066							
39876	*bla*$_{\text{OXA-1}}$	*aadA1, ant(3'')-Ia*	*dfrA1*				
640_MMOR							
716_MMOR		*aadA5, aacC2d*	*dfrA17*			*tetA*(B)	*mph*(A)
AA1							
AP69	*bla*$_{\text{TEM-1B}}$, *bla*$_{\text{CTX-M-15}}$, *bla*$_{\text{OXA-1}}$	*aacC2, aacA4cr, strA, strB*	*dfrA15*	*catA1, catB3*	*qnrB1, aacA4cr*	*tetA*(A), *tetA*(B)	*mph*(E)
AS012332	*bla*$_{\text{CARB-2}}$	*aadB, aphA1, aadA2b*	*dfrA19*	*catA1, catB3*		*tetA*(B)	*ere*(B)
AS012489	*bla*$_{\text{TEM-1B}}$, *bla*$_{\text{SCO-1}}$, *bla*$_{\text{OXA-1}}$	*aacC2, ant(3'')-Ia, strA, aphA1, strB, aadA1*	*dfrA1*	*catA1*			
AV1							

B2					*qnrD1*		
B3					*qnrD1*		
B5					*qnrD1*		
C135							
CQ-M7							
CRK0002							
CRK0058		*aphA1, strA, strB*				*tetA*(B)	
E042							
EH8						*tetA*(D)	
F675	*bla*$_{NDM-1}$, *bla*$_{CARB-2}$	*aadA2b, aphA6*	*dfrA19*	*catA1*		*tetA*(B)	*mph*(A), *ere*(A), *ere*(B)
FDAARGOS_438	*bla*$_{NDM-1}$, *bla*$_{TEM-1B}$	*strA, strB, aadA5, rmtC, aacC2d*	*dfrA17*	*catA1*		*tetA*(B)	*mph*(A)
HE-MDREc28						*tetA*(D)	
ICBMmBL-II-04	*bla*$_{GES-5}$, *bla*$_{OXA-1}$	*aacA4cr, aacA4, aadA2b*	*dfrA15*	*catB3*	*aacA4cr*	*tetA*(B)	*mph*(E)
INSali207		*aadB, aadA1*		*cmlA1*		*tetA*(B)	*mph*(A)
IS15	*bla*$_{CTX-M-15}$, *bla*$_{OXA-1}$, *bla*$_{TEM-1B}$	*aacA4cr, aacC2, aacC2d*	*dfrA15*	*catB3*	*aacA4cr*		*erm*(42)
M006		*aadA7*			*qnrD1*		
MM_1							
MM_190							
MM_4							
MMM_73					*qnrD1*	*tetA*(B)	
MMM_77		*aacC2d, aadA5, aphA1*	*dfrA17*			*tetA*(B)	*mph*(A)
MRSN22709	*bla*$_{OXA-181}$		*dfrA14*		*qnrS1*		
NBRC_3848							

NCTC12286	*bla*$_{TEM-110}$					*tetA*(B)	
NCTC12289							
NCTC232							
NLAE-zl-C84						*tetA*(D)	
nx_m63	*bla*$_{IMP-1}$	*aadA1*				*tetA*(B)	
PA18_15564							
PA18_16407							
PA18_25921							
PA19_9695						*tetA*(B)	
RD-40764	*bla*$_{NDM-1}$, *bla*$_{OXA-1}$, *bla*$_{CMY-4}$, *bla*$_{TEM-1B}$	*strB, strA, aacA4cr, aacC2d, aphA6*	*dfrA14*	*catB3*	*qnrB9, aacA4cr*	*tetA*(A)	
SA36						*tetA*(D)	
SC01	*bla*$_{CTX-M-15}$	*aadA5, armA, strB, strA*	*dfrA17*	*catA1*			*mph*(E)
SCsl21		*aacC2d, strB, aphA1, strA, aadA5, aacC4, aph(4)-Ia*	*dfrA17*	*floR*		*tetA*(B)	*mph*(A)
SCsl4	*bla*$_{OXA-1}$, *bla*$_{TEM-1B}$, *bla*$_{CTX-M-3}$	*strB, aphA1, strA, aacA4cr, aacC4, aph(4)-Ia, aadA2, armA*	*dfrA12*	*floR, catB3*	*aacA4cr*	*tetA*(B)	*mph*(E)
szy_m16							
szy_m2	*bla*$_{TEM-1B}$	*strA, strB, aacC2d, aphA1, aadA2*	*dfrA12*	*catA1*		*tetA*(B)	*mph*(A)
szy_m22	*bla*$_{OXA-1}$	*aacA4cr*		*catB3*	*aacA4cr*	*tetA*(D)	
szy_m28						*tetA*(B)	
szy_m3							
szy_m40					*qnrD1*	*tetA*(D)	
Tannery_effluent_MM_1							
TUM2748		*aphA1, aadB*		*catA1*		*tetA*(B)	
UMB1297	*bla*$_{OXA-1}$	*aadA1, aadA5, aacA4cr*	*dfrA17*	*catB3*	*aacA4cr*		

zy_m16							
zy_m28						*tetA*(B)	
zy_m3							
VGH116							
COL11						*tetA*(B)	
COL10						*tetA*(D)	
1001216B_150713_C11					*qnrD2*	*tetA*(D)	
SMM01	$bla_{\text{OXA-1}}$	*aadA1, aacA4cr*			*aacA4cr*	*tetA*(B)	
GDMM86	$bla_{\text{NDM-1}}$	*aadA1, aadA2, aphA6, aphA1, aph(4)-Ia, aacC4*	*dfrA1, dfrA12*	*catB3, floR*		*tetA*(D)	*mph*(A), *erm*(42)
FDAARGOS_1085							
EGD-HP17							
HMSC11D09						*tetA*(D)	
GLFB						*tetA*(D)	

Table S1. (continued)

Isolate	Acquired antimicrobial resistance genes						
	**Sulphonamides**	**Lincosamides**	**Polymyxins**	**Quaternary ammonium compounds**	**Rifamycin**	**Fosfomycins**	**Phenicols, lincosamides, oxazolidinones, pleuromutilins, and streptogramin A**
11759	*sul1, sul2*			*qacED1*		*fosA3*	
516602	*sul2*						
ZJC25	*sul1*			*qacED1*			
229813	*sul1, sul2, sul3*			*qacED1, qacH2*	*arr-3*		
621164	*sul1*	*lnu*(F)		*qacED1*	*arr-3*		
715304	*sul1*			*qacED1*			
GN28	*sul1*			*qacED1*	*arr-3*		
81703	*sul1, sul2*	*lnu*(F)		*qacED1*	*arr-3*		
ZJG944	*sul1, sul2*			*qacED1*	*arr-3*		
ZJG812	*sul1*			*qacED1*	*arr-3*		
ZJD581	*sul1, sul2*	*lnu*(G)		*qacED1*	*arr-3*		

12304	*sul1*, *sul2*			*qacED1*	*arr-3*		
ZJE758	*sul1*, *sul2*	*lnu*(F)		*qacED1*			
719030	*sul1*			*qacED1*	*arr-3*		
ZJF932	*sul1*, *sul2*			*qacED1*	*arr-3*		
HBZH72	*sul1*, *sul2*			*qacED1*	-		
926193					-		
GZH767	*sul1*			*qacED1*	-		
321150	*sul1*, *sul2*			*qacED1*	-		
110888	*sul1*			*qacED1*	-		
1701013	*sul1*, *sul2*			*qacED1*	*arr-3*		*cfr*
QHD13	*sul1*, *sul2*			*qacED1*	*arr-3*		
HBZH71	*sul1*			*qacED1*	-		
192009	*sul1*			*qacED1*	*arr-3*		
QHD15	*sul1*, *sul2*			*qacED1*	-		
ZJG980	*sul1*, *sul2*			*qacED1*	-		
1701016	*sul1*, *sul2*			*qacED1*	-	*fosA3*	
ZJC503	*sul1*, *sul2*			*qacED1*	*arr-3*		
569035	*sul1*, *sul2*			*qacED1*	-		
A0073PB	*sul1*			*qacED1*	-		
81201	*sul1*, *sul2*	*lnu*(F)		*qacED1*	*arr-3*		
105294	*sul1*	*lnu*(F)		*qacED1*	*arr-3*		
BJGN25	*sul1*			*qacED1*	*arr-3*		
225205					-		
ZJE806	*sul2*				-		
ZJD25	*sul2*				-		
BJE28					-		

ZJA650					-		
ZJC213					-		
BJ121					-		
ZJC502					-		
ZJC234					-		
ZJC212					-		
ZJF894					-		
ZJF601					-		
62705					-		
ZJG941					-		
ZJE871					-		
ZJD186					-		
504293					-		
420211					-		
ZJB223					-		
112720					-		
ZJF862					-		
ZJG310					-		
ZJE82					-		
ZJE89					-		
ZJD545					-		
262005					-		
122035					-		
GCSL-Mp3					-		
GCSL-Mp20					-		
UBA11277					-		

CCUG_53682T					-		
GCSL-P101					-		
MMsCG					-		
UMGS836					-		
GCSL-TSO-24					-		
H1r					-		
M.m274	*sul2*				-		
PA17_10312					-		
FAM24091					-		
UBA11259					-		
NCTC12358					-		
MGYG-HGUT-02513					-		
TW17014					-		
L3					-		
INSRALV892	*sul2*				-		
FDAARGOS_172	*sul2*				-		
FDAARGOS_365					-		
					-		
KC-Tt-01					-		
FDAARGOS_63					-		
AR_0057	*sul1, sul2*			*qacED1*	-		
AR_0133					-		
					-		
					-		
DG56-16					-		
L241					-		

					-		
ATCC_25830					-		
Jiangxi	*sul1*, *sul2*			*qacED1*	-		
N18-00103					-		
MP63					-		
					-		
					-		
NCTC235					-		
MGYG-HGUT-02512					-		
NCTC12028					-		
KT					-		
MH16-367M	*sul1*			*qacED1*	-		
78	*sul2*				-		
340					-		
4601					-		
8066					-		
39876	*sul1*			*qacED1*	-		
640_MMOR					-		
716_MMOR	*sul1*			*qacED1*	-		
AA1					-		
AP69	*sul1*, *sul2*			*qacED1*	-		
AS012332	*sul1*			*qacED1*	-		
AS012489	*sul1*			*qacED1*	-		
AV1					-		
B2					-		
B3					-		

B5					-		
C135					-		
CQ-M7					-		
CRK0002					-		
CRK0058	*sul2*				-		
E042					-		
EH8					-		
F675					-		
FDAARGOS_438	*sul1*, *sul2*				-		
HE-MDREc28					-		
ICBMmBL-II-04	*sul1*			*qacED1*	-		
INSali207	*sul1*			*qacED1*	-		
IS15	*sul1*, *sul2*			*qacED1*	-		
M006	*sul1*			*qacED1*	-		
MM_1					-		
MM_190					-		
MM_4					-		
MMM_73					-		
MMM_77	*sul1*			*qacED1*	-		
MRSN22709					-		
NBRC_3848					-		
NCTC12286					-		
NCTC12289					-		
NCTC232					-		
NLAE-zl-C84					-		
nx_m63					-		

PA18_15564					-		
PA18_16407					-		
PA18_25921					-		
PA19_9695					-		
RD-40764	*sul1, sul2*			*qacED1*	*arr-3*		
SA36					-		
SC01	*sul1, sul2*			*qacED1*	-		
SCsl21	*sul1, sul2*		*mcr-1.1*	*qacED1*	-		
SCsl4	*sul1, sul2*		*mcr-1.1*	*qacED1*	*arr-3*		
szy_m16					-		
szy_m2	*sul1, sul2*			*qacED1*	*arr-3*		
szy_m22	*sul1*				*arr-3*		
szy_m28					-		
szy_m3					-		
szy_m40					-		
Tannery_effluent_MM_1					-		
TUM2748	*sul1*			*qacED1*	-		
UMB1297	*sul1*			*qacED1*	-		
zy_m16					-		
zy_m28					-		
zy_m3					-		
VGH116					-		
COL11			*mcr-5.1*		-		
COL10			*mcr-1.1*		-		
1001216B_150713_C11					-		

SMM01					-		
GDMM86	*sul1*, *sul2*	*lnu*(F)		*qacED1*	*arr-3*		
FDAARGOS_1085					-		
EGD-HP17					-		
HMSC11D09					-		
GLFB					-		

Table S1. (continued)

Isolate	Minimum inhibitory concentration (μg/ml)/Antimicrobial susceptibility						
	**Cefazolin**	**Cefuroxime**	**Nitrofurantoin**	**Ampicillin**	**Ciprofloxacin**	**Levofloxacin**	**Trimethoprim/Sulfamethoxazole**
11759	>=64/R	>=64/R	64/R	>=32/R	>=4/R	2/R	>=320/R
516602	>=64/R	>=64/R	64/R	>=32/R	2/R	2/R	>=80/R
ZJC25	>=64/R	>=64/R	64/R	>=32/R	1/I	1/I	>=320/R
229813	>=64/R	>=64/R	128/R	>=32/R	>=4/R	>=8/R	>=320/R
621164	>=64/R	>=64/R	64/R	>=32/R	>=4/R	>=8/R	>=320/R
715304	>=64/R	>=64/R	128/R	>=32/R	>=4/R	>=8/R	>=320/R
GN28	>=64/R	>=64/R	64/R	>=32/R	>=4/R	>=8/R	>=80/R
81703	>=64/R	16/R	64/R	>=32/R	<=0.5/S	<=0.5/S	>=320/R
ZJG944	>=64/R	>=64/R	64/R	>=32/R	<=0.5/S	<=0.5/S	>=320/R
ZJG812	>=64/R	>=64/R	64/R	>=32/R	<=0.5/S	<=0.5/S	>=320/R
ZJD581	>=64/R	>=64/R	64/R	>=32/R	>=4/R	>=8/R	>=320/R
12304	>=64/R	>=64/R	128/R	>=32/R	>=4/R	>=8/R	>=320/R

ZJE758	>=64/R	>=64/R	64/R	>=32/R	>=4/R	1/I	>=320/R
719030	>=64/R	>=64/R	64/R	>=32/R	>=4/R	4/R	>=320/R
ZJF932	>=64/R	>=64/R	64/R	>=32/R	>=4/R	1/I	>=320/R
HBZH72	>=64/R	>=64/R	128/R	>=32/R	>=4/R	>=8/R	>=320/R
926193	>=64/R	>=64/R	64/R	>=32/R	0.5/I	1/I	<=20/S
GZH767	>=64/R	>=64/R	64/R	>=32/R	<=0.5/S	<=0.5/S	>=320/R
321150	>=64/R	>=64/R	128/R	>=32/R	2/R	1/I	>=320/R
110888	>=64/R	>=64/R	128/R	>=32/R	<=0.5/S	0.5/I	>=320/R
1701013	>=64/R	>=64/R	64/R	>=32/R	>=4/R	2/R	>=320/R
QHD13	>=64/R	>=64/R	128/R	>=32/R	>=4/R	>=8/R	>=320/R
HBZH71	>=64/R	>=64/R	128/R	>=32/R	1/I	1/I	>=320/R
192009	>=64/R	>=64/R	64/R	>=32/R	>=4/R	2/R	>=320/R
QHD15	>=64/R	>=64/R	64/R	>=32/R	0.5/I	1/I	>=320/R
ZJG980	>=64/R	>=64/R	64/R	>=32/R	1/R	1/I	>=320/R
1701016	>=64/R	>=64/R	64/R	>=32/R	>=4/R	>=8/R	>=320/R
ZJC503	>=64/R	>=64/R	64/R	>=32/R	>=4/R	1/I	>=320/R
569035	>=64/R	>=64/R	64/R	>=32/R	>=4/R	4/R	>=320/R
A0073PB	>=64/R	>=64/R	64/R	>=32/R	2/R	1/I	>=320/R
81201	>=64/R	>=64/R	128/R	>=32/R	<=0.5/S	<=0.5/S	>=320/R
105294	>=64/R	>=64/R	64/R	>=32/R	>=4/R	>=8/R	>=320/R
BJGN25	32/R	16/R	64/R	>=32/R	>=4/R	>=8/R	>=320/R
225205	>=64/R	>=64/R	128/R	>=32/R	>=4/R	>=8/R	<=20/S
ZJE806	>=64/R	16/R	64/R	>=32/R	<=0.5/S	<=0.5/S	>=320/R
ZJD25	>=64/R	>=64/R	128/R	>=32/R	2/R	1/I	>=320/R
BJE28	>=64/R	>=64/R	256/R	>=32/R	<=0.5/S	0.5/I	<=20/S
ZJA650	>=64/R	>=64/R	64/R	>=32/R	2/R	2/R	<=20/S

ZJC213	>=64/R	>=64/R	64/R	>=32/R	<=0.5/S	<=0.5/S	<=20/S
BJ121	>=64/R	32/R	64/R	>=32/R	<=0.5/S	0.5/I	<=20/S
ZJC502	>=64/R	16/R	64/R	>=32/R	<=0.5/S	<=0.5/S	<=20/S
ZJC234	>=64/R	>=64/R	128/R	>=32/R	<=0.5/S	<=0.5/S	<=20/S
ZJC212	>=64/R	>=64/R	64/R	>=32/R	<=0.5/S	0.5/I	<=20/S
ZJF894	>=64/R	>=64/R	64/R	>=32/R	<=0.5/S	<=0.5/S	<=20/S
ZJF601	>=64/R	>=64/R	128/R	>=32/R	<=0.5/S	<=0.5/S	<=20/S
62705	>=64/R	>=64/R	128/R	>=32/R	1/I	1/I	<=20/S
ZJG941	>=64/R	>=64/R	128/R	>=32/R	>=4/R	1/I	<=20/S
ZJE871	>=64/R	32/R	64/R	>=32/R	<=0.5/S	<=0.5/S	<=20/S
ZJD186	>=64/R	>=64/R	128/R	>=32/R	<=0.5/S	<=0.5/S	<=20/S
504293	>=64/R	32/R	64/R	>=32/R	1/I	1/I	<=20/S
420211	>=64/R	>=64/R	128/R	>=32/R	<=0.5/S	<=0.5/S	<=20/S
ZJB223	>=64/R	32/R	64/R	>=32/R	<=0.5/S	<=0.5/S	<=20/S
112720	>=64/R	>=64/R	128/R	>=32/R	1/I	1/I	<=20/S
ZJF862	>=64/R	16/R	64/R	>=32/R	<=0.5/S	<=0.5/S	<=20/S
ZJG310	>=64/R	>=64/R	128/R	>=32/R	>=4/R	2/R	<=20/S
ZJE82	>=64/R	>=64/R	128/R	>=32/R	>=4/R	1/I	<=20/S
ZJE89	>=64/R	>=64/R	64/R	>=32/R	<=0.5/S	<=0.5/S	<=20/S
ZJD545	>=64/R	>=64/R	64/R	>=32/R	<=0.5/S	<=0.5/S	<=20/S
262005	>=64/R	>=64/R	128/R	>=32/R	<=0.5/S	<=0.5/S	<=20/S
122035	>=64/R	>=64/R	128/R	>=32/R	<=0.5/S	<=0.5/S	<=20/S

Table S1. (continued)

Isolate	Minimum inhibitory concentration (µg/ml)/Antimicrobial susceptibility							
	**Gentamicin**	**Tobramycin**	**Ceftazidime**	**Ceftriaxone**	**Aztreonam**	**Cefepime**	**Meropenem**	**Amikacin**
11759	>=16/R	8/I	8/I	>=64/R	32/R	32/R	<=0.5/S	<=2/S
516602	<=1/S	<=1/S	16/R	32/R	4/S	>=64/R	4/R	<=2/S
ZJC25	<=1/S	<=1/S	4/S	<=1/S	<=1/S	<=1/S	<=0.5/S	<=2/S
229813	8/I	>=16/R	16/R	8/R	4/S	>=64/R	4/R	<=2/S
621164	>=16/R	>=16/R	<=1/S	>=64/R	<=1/S	<=1/S	<=0.5/S	>=64/R
715304	4/S	2/S	>=64/R	>=64/R	16/R	<=1/S	1/S	4/S
GN28	>=16/R	8/I	16/R	32/R	<=1/S	>=64/R	4/R	4/S
81703	<=1/S	<=1/S	<=1/S	<=1/S	<=1/S	<=1/S	<=0.5/S	<=2/S
ZJG944	>=16/R	>=16/R	<=1/S	<=1/S	<=1/S	<=1/S	<=0.5/S	4/S
ZJG812	<=2/S	2/S	<=1/S	<=1/S	<=1/S	<=1/S	<=0.5/S	<=2/S
ZJD581	>=16/R	8/I	>=64/R	16/R	>=64/R	<=1/S	<=0.5/S	16/S
12304	>=16/R	>=16/R	8/I	2/I	<=1/S	4/S	1/S	4/S

ZJE758	>=16/R	>=16/R	<=1/S	<=1/S	<=1/S	<=1/S	<=0.5/S	>=64/R
719030	4/S	8/I	16/R	2/I	<=1/S	<=1/S	<=0.5/S	4/S
ZJF932	>=16/R	8/I	<=1/S	<=1/S	<=1/S	<=1/S	<=0.5/S	8/S
HBZH72	>=16/R	4/S	>=64/R	>=64/R	32/R	>=64/R	>=16/R	<=2/S
926193	2/S	<=1/S	>=64/R	32/R	32/R	<=1/S	0.5/S	16/S
GZH767	<=1/S	<=1/S	32/R	8/R	<=1/S	8/S	<=0.5/S	4/S
321150	>=16/R	4/S	32/R	8/R	32/R	16/R	<=0.5/S	4/S
110888	<=1/S	<=1/S	<=1/S	<=1/S	<=1/S	<=1/S	<=0.5/S	<=2/S
1701013	>=16/R	>=16/R	<=1/S	>=64/R	<=1/S	<=1/S	<=0.5/S	4/S
QHD13	>=16/R	>=16/R	<=1/S	<=1/S	<=1/S	<=1/S	<=0.5/S	4/S
HBZH71	>=16/R	<=2/S	>=64/R	32/R	<=1/S	>=4/R	>=16/R	<=2/S
192009	>=16/R	8/I	<=1/S	2/I	<=1/S	<=1/S	<=0.5/S	4/S
QHD15	8/I	8/I	<=1/S	<=1/S	<=1/S	<=1/S	0.5/S	<=2/S
ZJG980	<=1/S	<=1/S	<=1/S	<=1/S	<=1/S	<=1/S	<=0.5/S	<=2/S
1701016	>=16/R	8/I	<=1/S	<=1/S	<=1/S	<=1/S	<=0.5/S	<=2/S
ZJC503	>=16/R	8/I	<=1/S	<=1/S	<=1/S	<=1/S	<=0.5/S	<=2/S
569035	>=16/R	2/S	>=64/R	32/R	16/R	<=1/S	<=0.5/S	<=2/S
A0073PB	8/I	8/I	<=1/S	<=1/S	<=1/S	<=1/S	<=0.5/S	<=2/S
81201	<=1/S	<=1/S	8/I	<=1/S	16/R	4/S	<=0.5/S	<=2/S
105294	>=16/R	>=16/R	<=1/S	>=64/R	<=1/S	<=1/S	<=0.5/S	>=64/R
BJGN25	>=16/R	8/I	16/R	32/R	4/S	>=64/R	4/R	8/S
225205	>=16/R	8/I	32/R	>=64/R	32/R	>=64/R	<=0.5/S	4/S
ZJE806	<=1/S	<=1/S	<=1/S	<=1/S	<=1/S	<=1/S	<=0.5/S	<=2/S
ZJD25	<=1/S	<=1/S	>=64/R	32/R	>=64/R	32/R	<=0.5/S	8/S
BJE28	<=1/S	<=1/S	<=1/S	<=1/S	<=1/S	<=1/S	<=0.5/S	<=2/S
ZJA650	<=1/S	<=1/S	<=1/S	<=1/S	<=1/S	<=1/S	<=0.5/S	<=2/S

ZJC213	<=1/S	<=1/S	<=1/S	<=1/S	<=1/S	<=1/S	<=0.5/S	<=2/S
BJ121	<=1/S	<=1/S	<=1/S	<=1/S	<=1/S	<=1/S	<=0.5/S	<=2/S
ZJC502	<=1/S	<=1/S	<=1/S	<=1/S	<=1/S	<=1/S	<=0.5/S	<=2/S
ZJC234	<=1/S	<=1/S	<=1/S	<=1/S	<=1/S	<=1/S	<=0.5/S	<=2/S
ZJC212	<=1/S	<=1/S	<=1/S	<=1/S	<=1/S	<=1/S	<=0.5/S	<=2/S
ZJF894	<=1/S	<=1/S	<=1/S	<=1/S	<=1/S	<=1/S	<=0.5/S	<=2/S
ZJF601	<=1/S	<=1/S	<=1/S	<=1/S	<=1/S	<=1/S	<=0.5/S	<=2/S
62705	<=1/S	<=1/S	<=1/S	<=1/S	<=1/S	<=1/S	<=0.5/S	<=2/S
ZJG941	>=16/R	>=16/R	<=1/S	<=1/S	<=1/S	<=1/S	<=0.5/S	<=2/S
ZJE871	<=1/S	<=1/S	<=1/S	<=1/S	<=1/S	<=1/S	<=0.5/S	<=2/S
ZJD186	<=1/S	<=1/S	<=1/S	<=1/S	<=1/S	<=1/S	<=0.5/S	<=2/S
504293	<=1/S	<=1/S	32/R	16/R	8/I	8/S	<=0.5/S	4/S
420211	<=1/S	<=1/S	<=1/S	<=1/S	<=1/S	<=1/S	<=0.5/S	<=2/S
ZJB223	<=1/S	<=1/S	<=1/S	<=1/S	<=1/S	<=1/S	<=0.5/S	<=2/S
112720	<=1/S	<=1/S	32/R	8/R	<=1/S	<=1/S	<=0.5/S	<=2/S
ZJF862	<=1/S	<=1/S	<=1/S	<=1/S	<=1/S	<=1/S	<=0.5/S	<=2/S
ZJG310	<=1/S	<=1/S	<=1/S	<=1/S	<=1/S	<=1/S	<=0.5/S	16/S
ZJE82	>=16/R	>=16/R	<=1/S	<=1/S	<=1/S	<=1/S	<=0.5/S	4/S
ZJE89	<=1/S	<=1/S	<=1/S	<=1/S	<=1/S	<=1/S	<=0.5/S	<=2/S
ZJD545	>=16/R	<=1/S	<=1/S	<=1/S	<=1/S	<=1/S	<=0.5/S	<=2/S
262005	<=1/S	<=1/S	<=1/S	<=1/S	<=1/S	<=1/S	<=0.5/S	<=2/S
122035	<=1/S	<=1/S	<=1/S	<=1/S	<=1/S	<=1/S	<=0.5/S	<=2/S

Table S1. (continued)

**Isolate**	**IS*26*/IS*15DI***	**IS*10***	**IS*1***	**Tn*3*-family transposons**					
				**Tn*3*-subfamily**	**Tn*21*-subfamily**	**Tn*163*-subfamily**	**Tn*4430*-subfamily**	**Tn*4651*-subfamily**	**Tn*4401*-subfamily**
11759	+	+	+		+				
516602	+	+							
ZJC25		+							
229813	+	+			+				
621164	+	+	+	+					
715304	+	+	+						
GN28	+	+	+						
81703	+	+			+				
ZJG944	+		+		+				
ZJG812	+		+		+				
ZJD581	+	+			+				
12304	+	+			+				

ZJE758	+	+	+		+				
719030	+	+	+						
ZJF932	+	+	+		+				
HBZH72	+	+	+		+				
926193		+							
GZH767	+	+	+		+				
321150	+	+	+	+	+				
110888	+	+	+		+				
1701013	+			+	+				
QHD13	+	+							
HBZH71	+	+							
192009	+	+	+		+				
QHD15	+	+							
ZJG980	+								
1701016	+	+	+		+				
ZJC503	+	+	+		+				
569035	+	+			+				
A0073PB									
81201	+	+			+				
105294	+	+	+	+					
BJGN25	+	+	+	+					
225205	+	+	+						
ZJE806									
ZJD25	+	+	+						
BJE28		+							
ZJA650									

ZJC213	+								
BJ121									
ZJC502		+							
ZJC234									
ZJC212		+							
ZJF894									
ZJF601	+	+							
62705									
ZJG941									
ZJE871									
ZJD186									
504293									
420211									
ZJB223									
112720									
ZJF862									
ZJG310	+	+							
ZJE82		+							
ZJE89		+							
ZJD545	+	+							
262005									
122035									
GCSL-Mp3	+								
GCSL-Mp20									
UBA11277									
CCUG_53682T									

GCSL-P101									
MMsCG									
UMGS836									
GCSL-TSO-24									
H1r									
M.m274	+				+				
PA17_10312									
FAM24091			+						
UBA11259									
NCTC12358									
MGYG-HGUT-02513									
TW17014									
L3									
INSRALV892	+								
FDAARGOS_172		+							
FDAARGOS_365									
KC-Tt-01									
FDAARGOS_63		+							
AR_0057	+	+	+	+	+				
AR_0133	+								
DG56-16									
L241	+								

ATCC_25830									
Jiangxi	+	+	+		+				
N18-00103									
MP63									
NCTC235									
MGYG-HGUT-02512									
NCTC12028									
KT		+							
MH16-367M	+	+	+	+					
78	+	+							
340	+								
4601									
8066									
39876	+		+		+				
640_MMOR									
716_MMOR	+	+							
AA1									
AP69	+	+	+	+	+				
AS012332	+	+	+						
AS012489	+	+	+	+	+				
AV1									
B2									
B3									

B5									
C135									
CQ-M7	+		+						
CRK0002									
CRK0058	+	+	+						
E042									
EH8									
F675									
FDAARGOS_438	+	+	+		+				
HE-MDREc28									
ICBMmBL-II-04	+	+							
INSali207	+	+							
IS15	+	+		+					
M006		+			+				
MM_1									
MM_190									
MM_4									
MMM_73		+							
MMM_77	+	+							
MRSN22709	+				+				
NBRC_3848									
NCTC12286		+		+					
NCTC12289									
NCTC232									
NLAE-zl-C84									
nx_m63	+	+							

PA18_15564									
PA18_16407									
PA18_25921									
PA19_9695		+	+						
RD-40764	+		+		+				
SA36									
SC01	+	+	+	+	+				
SCsl21									
SCsl4									
szy_m16									
szy_m2	+	+	+		+				
szy_m22									
szy_m28		+							
szy_m3									
szy_m40									
Tannery_effluent_MM_1									
TUM2748	+	+	+		+				
UMB1297	+		+						
zy_m16									
zy_m28	+								
zy_m3									
VGH116									
COL11		+							
COL10	+		+						
1001216B_150713_C11									

SMM01	+		+						
GDMM86	+				+				
FDAARGOS_1085									
EGD-HP17									
HMSC11D09									
GLFB									

Table S1. (continued)

Isolate	Tn*7*-related transposons					Tn*554*-related transposons		
	**Tn*7*-subfamily**	**Tn*6230*-subfamily**	**Tn*552*-subfamily**	**Tn*6022*-subfamily**	**Tn*5053*-subfamily**	**Tn*554*-subfamily**	**Tn*6488*-subfamily**	**Tn*6571*-subfamily**
11759								
516602								
ZJC25	+							
229813	+							
621164	+							
715304								
GN28								
81703	+							
ZJG944								
ZJG812								
ZJD581								
12304								

ZJE758	+							
719030								
ZJF932								
HBZH72								
926193								
GZH767								
321150								
110888	+							
1701013								
QHD13								
HBZH71								
192009								
QHD15	+							
ZJG980								
1701016								
ZJC503								
569035								
A0073PB								
81201	+							
105294	+							
BJGN25								
225205	+							
ZJE806	+							
ZJD25								
BJE28								
ZJA650								

ZJC213								
BJ121								
ZJC502								
ZJC234								
ZJC212								
ZJF894								
ZJF601								
62705								
ZJG941								
ZJE871								
ZJD186								
504293								
420211								
ZJB223								
112720								
ZJF862								
ZJG310								
ZJE82								
ZJE89								
ZJD545								
262005								
122035								
GCSL-Mp3								
GCSL-Mp20								
UBA11277								
CCUG_53682T								

GCSL-P101								
MMsCG								
UMGS836								
GCSL-TSO-24								
H1r								
M.m274								
PA17_10312								
FAM24091								
UBA11259								
NCTC12358								
MGYG-HGUT-02513								
TW17014								
L3	+							
INSRALV892	+							
FDAARGOS_172	+							
FDAARGOS_365								
KC-Tt-01								
FDAARGOS_63								
AR_0057								
AR_0133	+							
DG56-16								
L241								

ATCC_25830								
Jiangxi	+							
N18-00103								
MP63								
NCTC235								
MGYG-HGUT-02512								
NCTC12028								
KT								
MH16-367M								
78	+							
340								
4601								
8066								
39876	+							
640_MMOR								
716_MMOR								
AA1								
AP69								
AS012332								
AS012489	+							
AV1								
B2								
B3								

B5								
C135								
CQ-M7								
CRK0002								
CRK0058								
E042								
EH8								
F675								
FDAARGOS_438								
HE-MDREc28								
ICBMmBL-II-04								
INSali207								
IS15								
M006								
MM_1								
MM_190								
MM_4								
MMM_73								
MMM_77								
MRSN22709								
NBRC_3848								
NCTC12286								
NCTC12289								
NCTC232								
NLAE-zl-C84								
nx_m63	+							

PA18_15564								
PA18_16407								
PA18_25921								
PA19_9695								
RD-40764								
SA36								
SC01								
SCsl21								
SCsl4								
szy_m16								
szy_m2								
szy_m22								
szy_m28								
szy_m3								
szy_m40								
Tannery_effluent_MM_1								
TUM2748								
UMB1297	+							
zy_m16								
zy_m28								
zy_m3								
VGH116								
COL11								
COL10								
1001216B_150713_C11								

SMM01	+							
GDMM86	+							
FDAARGOS_1085								
EGD-HP17								
HMSC11D09								
GLFB								

Table S2. The quality control of genome sequences of *Morganella* isolates determined in this study

Category	Statistics	Draft genome sequences (n=60)[a]	Complete genome sequences (n=12)[b]
Depth (fold)	Minimum	233.3	173.14
	Maximum	562.08	92.17
	Average	340.26	239.77
Q20 (%)	Minimum	96.78	95.4
	Maximum	98.62	98.11
	Average	97.47	97.2
Q30 (%)	Minimum	90.33	88.66
	Maximum	95.7	94.35
	Average	92.97	92.31
%GC content	Minimum	50.57	
	Maximum	51.24	
	Average	50.99	
Contig N50 (%)	Minimum	19,303	
	Maximum	2,520,020	
	Average	539,659	
Read N50 (%)	Minimum		12,819
	Maximum		17,160
	Average		14,504

[a]The draft genome sequences of 60 *Morganella* isolates are determined herein. The lowest depth value of them is much larger than the threshold of 35 fold for genome sequence assembly and that of 50 fold for single nucleotide polymorphisms identification (1). The lowest Q20 value and Q30 value are higher than the commonly used quality control criterions Q20⩾90% and Q30⩾85% (2). The mean GC contents are consistent to the previously sequenced *Morganella* genomes (3).

[b]The complete genome sequences are further determined for 12 *Morganella* isolates herein. The average sequencing depth and read N50 value are qualified for assembly of mosaic accessory genetic elements based on the thresholds of sequencing depth⩾40-fold and read N50⩾11,000 bp (4).

REFERENCES

1. Desai A, Marwah VS, Yadav A, Jha V, Dhaygude K, Bangar U, Kulkarni V, Jere A. 2013. Identification of optimum sequencing depth especially for de novo genome assembly of small genomes using next generation sequencing data. *PLoS One* 8:e60204.
2. Fox EJ, Reid-Bayliss KS, Emond MJ, Loeb LA. 2014. Accuracy of Next Generation Sequencing Platforms. *Next Gener Seq Appl* 1:1000106.
3. Minnullina L, Pudova D, Shagimardanova E, Shigapova L, Sharipova M, Mardanova A. 2019. Comparative Genome Analysis of Uropathogenic *Morganella morganii* Strains. *Front Cell Infect Microbiol* 9:167.
4. Ou S, Liu J, Chougule KM, Fungtammasan A, Seetharam AS, Stein JC, Llaca V, Manchanda N, Gilbert AM, Wei S, Chin C-S, Hufnagel DE, Pedersen S, Snodgrass SJ, Fengler K, Woodhouse M, Walenz BP, Koren S, Phillippy AM, Hannigan BT, Dawe RK, Hirsch CN, Hufford MB, Ware D. 2020. Effect of sequence depth and length in long-read assembly of the maize inbred NC358. *Nat Commun* 11:2288.

TableS3. Pairwise comparison of the genome sequences of 166 global *Morganella* isolates using ANI analysis

Isolate	1	2	3	4	5	6	7	8	9	10
**1**	100.00	99.93	99.73	99.97	99.65	99.80	99.50	99.70	99.80	98.74
**2**	99.93	100.00	99.76	99.91	99.74	99.77	99.54	99.68	99.76	98.73
**3**	99.73	99.76	100.00	99.76	99.72	99.76	99.52	99.68	99.86	98.77
**4**	99.97	99.91	99.76	100.00	99.76	99.87	99.53	99.77	99.80	98.78
**5**	99.65	99.74	99.72	99.76	100.00	99.77	99.55	99.76	99.70	98.75
**6**	99.80	99.77	99.76	99.87	99.77	100.00	99.56	99.68	99.81	98.80
**7**	99.50	99.54	99.52	99.53	99.55	99.56	100.00	99.54	99.52	98.62
**8**	99.70	99.68	99.68	99.77	99.76	99.68	99.54	100.00	99.73	98.74
**9**	99.80	99.76	99.86	99.80	99.70	99.81	99.52	99.73	100.00	98.73
**10**	98.74	98.73	98.77	98.78	98.75	98.80	98.62	98.74	98.73	100.00
**11**	99.75	99.74	99.75	99.80	99.77	99.76	99.63	99.73	99.75	98.76
**12**	99.69	99.71	99.71	99.72	99.75	99.82	99.57	99.85	99.72	98.72
**13**	99.81	99.78	99.73	99.82	99.73	99.80	99.57	99.71	99.80	98.78
**14**	99.75	99.71	99.90	99.81	99.69	99.82	99.52	99.68	99.86	98.71
**15**	99.66	99.68	99.71	99.72	99.72	99.76	99.60	99.81	99.72	98.68
**16**	99.67	99.60	99.72	99.71	99.73	99.69	99.64	99.78	99.75	98.68
**17**	99.72	99.70	99.68	99.77	99.79	99.74	99.67	99.75	99.75	98.76
**18**	99.70	99.71	99.65	99.80	99.70	99.73	99.54	99.66	99.73	98.71
**19**	99.12	99.07	99.12	99.21	99.09	99.15	98.93	99.10	99.10	98.70
**20**	98.63	98.63	98.61	98.67	98.61	98.66	98.57	98.66	98.60	98.61
**21**	98.66	98.67	98.61	98.71	98.66	98.68	98.53	98.68	98.64	98.63
**22**	98.66	98.61	98.58	98.72	98.58	98.61	98.47	98.63	98.56	98.63
**23**	98.93	98.91	98.89	98.96	98.95	98.94	98.82	98.91	98.90	98.84
**24**	98.88	98.87	98.86	98.88	98.85	98.89	98.76	98.87	98.81	98.75
**25**	98.92	98.89	98.88	98.90	98.90	98.91	98.83	98.89	98.88	98.82
**26**	98.87	98.87	98.92	98.92	98.91	98.94	98.83	98.86	98.93	98.85
**27**	98.75	98.68	98.70	98.79	98.74	98.77	98.64	98.69	98.70	98.78
**28**	98.77	98.77	98.76	98.83	98.72	98.78	98.68	98.75	98.66	98.82
**29**	98.65	98.68	98.68	98.78	98.64	98.76	98.58	98.64	98.68	98.78
**30**	98.66	98.70	98.73	98.78	98.65	98.73	98.61	98.70	98.67	98.78
**31**	98.57	98.61	98.66	98.68	98.61	98.73	98.54	98.60	98.59	98.78
**32**	98.58	98.63	98.69	98.59	98.60	98.70	98.50	98.58	98.61	98.83
**33**	98.66	98.68	98.63	98.68	98.58	98.75	98.54	98.63	98.65	98.88
**34**	98.80	98.77	98.75	98.80	98.78	98.85	98.73	98.73	98.80	99.02
**35**	98.68	98.71	98.70	98.69	98.64	98.69	98.51	98.58	98.59	98.89
**36**	99.79	99.85	99.75	99.86	99.76	99.76	99.55	99.75	99.81	98.84
**37**	98.73	98.61	98.70	98.79	98.63	98.76	98.57	98.61	98.63	98.66
**38**	98.73	98.75	98.80	98.83	98.85	98.88	98.63	98.74	98.80	98.83
**39**	98.71	98.78	98.77	98.78	98.79	98.89	98.70	98.77	98.76	98.82
**40**	98.73	98.71	98.70	98.77	98.62	98.71	98.54	98.70	98.70	98.75

**41**	98.29	98.35	98.33	98.37	98.32	98.33	98.20	98.31	98.26	98.37
**42**	98.25	98.23	98.35	98.30	98.31	98.35	98.22	98.28	98.25	98.33
**43**	98.28	98.25	98.31	98.32	98.29	98.32	98.20	98.34	98.29	98.39
**44**	98.27	98.23	98.30	98.32	98.28	98.30	98.18	98.36	98.27	98.35
**45**	98.44	98.42	98.37	98.44	98.41	98.45	98.35	98.41	98.45	98.46
**46**	98.29	98.27	98.33	98.36	98.24	98.31	98.21	98.32	98.28	98.39
**47**	98.40	98.38	98.36	98.45	98.42	98.41	98.30	98.33	98.39	98.36
**48**	98.49	98.43	98.43	98.49	98.48	98.46	98.44	98.47	98.47	98.50
**49**	98.36	98.34	98.31	98.43	98.33	98.38	98.28	98.35	98.35	98.36
**50**	98.47	98.43	98.48	98.51	98.47	98.55	98.35	98.49	98.44	98.47
**51**	98.43	98.39	98.39	98.47	98.44	98.44	98.32	98.45	98.40	98.36
**52**	98.27	98.30	98.28	98.38	98.30	98.36	98.20	98.23	98.13	98.35
**53**	98.53	98.51	98.50	98.53	98.51	98.52	98.44	98.49	98.54	98.55
**54**	98.48	98.50	98.51	98.51	98.42	98.51	98.36	98.49	98.53	98.54
**55**	98.31	98.33	98.40	98.39	98.36	98.42	98.27	98.29	98.29	98.34
**56**	98.25	98.21	98.30	98.32	98.26	98.35	98.16	98.26	98.29	98.28
**57**	98.49	98.48	98.51	98.50	98.50	98.56	98.45	98.44	98.53	98.48
**58**	98.49	98.49	98.51	98.50	98.51	98.55	98.44	98.44	98.54	98.50
**59**	98.43	98.45	98.45	98.50	98.43	98.45	98.32	98.48	98.45	98.37
**60**	98.43	98.45	98.45	98.50	98.43	98.45	98.32	98.48	98.45	98.37
**61**	98.46	98.48	98.43	98.50	98.44	98.46	98.36	98.42	98.46	98.50
**62**	98.46	98.40	98.40	98.46	98.39	98.45	98.28	98.41	98.37	98.47
**63**	98.43	98.38	98.48	98.48	98.46	98.51	98.39	98.42	98.40	98.39
**64**	98.45	98.46	98.39	98.48	98.41	98.46	98.33	98.42	98.34	98.44
**65**	98.42	98.40	98.43	98.48	98.45	98.47	98.37	98.39	98.39	98.39
**66**	98.36	98.38	98.35	98.36	98.37	98.38	98.26	98.33	98.39	98.37
**67**	98.35	98.38	98.37	98.42	98.33	98.37	98.27	98.33	98.32	98.37
**68**	98.41	98.35	98.40	98.47	98.37	98.42	98.34	98.33	98.41	98.42
**69**	98.37	98.32	98.33	98.44	98.37	98.47	98.27	98.40	98.37	98.40
**70**	98.43	98.41	98.43	98.48	98.40	98.48	98.39	98.36	98.42	98.45
**71**	98.33	98.32	98.24	98.32	98.21	98.39	98.16	98.24	98.27	98.35
**72**	98.28	98.33	98.26	98.35	98.24	98.37	98.18	98.27	98.32	98.33
**73**	98.49	98.45	98.48	98.53	98.49	98.53	98.41	98.44	98.46	98.47
**74**	98.28	98.23	98.33	98.34	98.33	98.42	98.24	98.29	98.40	98.31
**75**	98.52	98.46	98.44	98.52	98.49	98.54	98.45	98.49	98.46	98.52
**76**	98.39	98.36	98.46	98.44	98.38	98.33	98.29	98.35	98.35	98.36
**77**	98.30	98.30	98.37	98.38	98.32	98.40	98.26	98.34	98.29	98.29
**78**	98.39	98.43	98.45	98.40	98.38	98.51	98.32	98.44	98.45	98.46
**79**	98.44	98.40	98.43	98.45	98.36	98.45	98.32	98.38	98.46	98.36
**80**	98.39	98.38	98.33	98.42	98.41	98.38	98.33	98.33	98.40	98.45
**81**	98.46	98.41	98.39	98.49	98.45	98.48	98.40	98.42	98.40	98.46
**82**	98.30	98.28	98.31	98.34	98.33	98.37	98.23	98.37	98.26	98.30
**83**	98.28	98.30	98.21	98.35	98.24	98.37	98.23	98.22	98.28	98.39

**84**	98.36	98.35	98.34	98.43	98.31	98.38	98.26	98.35	98.32	98.35
**85**	98.42	98.38	98.38	98.41	98.35	98.47	98.26	98.37	98.39	98.34
**86**	98.41	98.45	98.41	98.52	98.38	98.47	98.32	98.38	98.40	98.42
**87**	98.35	98.38	98.38	98.42	98.32	98.35	98.30	98.35	98.35	98.41
**88**	98.40	98.40	98.43	98.42	98.36	98.43	98.34	98.37	98.41	98.38
**89**	98.36	98.34	98.43	98.34	98.34	98.43	98.30	98.36	98.40	98.35
**90**	98.37	98.38	98.35	98.46	98.35	98.39	98.29	98.35	98.38	98.43
**91**	98.32	98.30	98.33	98.35	98.28	98.33	98.22	98.30	98.29	98.33
**92**	98.38	98.38	98.36	98.45	98.36	98.41	98.26	98.36	98.36	98.41
**93**	98.32	98.25	98.25	98.35	98.21	98.37	98.19	98.30	98.26	98.33
**94**	98.27	98.27	98.28	98.36	98.19	98.35	98.14	98.31	98.26	98.38
**95**	98.47	98.38	98.49	98.48	98.49	98.55	98.45	98.42	98.49	98.50
**96**	98.45	98.45	98.48	98.51	98.45	98.52	98.39	98.40	98.47	98.55
**97**	98.44	98.40	98.47	98.50	98.41	98.45	98.38	98.45	98.45	98.47
**98**	98.54	98.49	98.55	98.57	98.57	98.60	98.49	98.50	98.54	98.59
**99**	98.47	98.46	98.46	98.49	98.49	98.56	98.37	98.52	98.42	98.54
**100**	98.47	98.44	98.45	98.49	98.40	98.50	98.36	98.47	98.35	98.50
**101**	98.42	98.42	98.42	98.47	98.38	98.50	98.25	98.39	98.34	98.50
**102**	98.46	98.41	98.45	98.51	98.43	98.47	98.34	98.47	98.44	98.55
**103**	98.43	98.45	98.37	98.44	98.35	98.45	98.36	98.46	98.39	98.42
**104**	98.33	98.29	98.36	98.43	98.38	98.39	98.27	98.35	98.35	98.39
**105**	98.50	98.48	98.45	98.49	98.39	98.49	98.29	98.46	98.39	98.50
**106**	98.49	98.45	98.50	98.50	98.44	98.54	98.39	98.48	98.50	98.49
**107**	98.35	98.30	98.34	98.42	98.25	98.31	98.22	98.29	98.24	98.29
**108**	98.30	98.30	98.36	98.40	98.20	98.31	98.14	98.29	98.24	98.31
**109**	98.44	98.38	98.45	98.52	98.41	98.43	98.35	98.46	98.42	98.47
**110**	98.32	98.29	98.36	98.40	98.32	98.39	98.27	98.32	98.32	98.34
**111**	98.44	98.36	98.41	98.49	98.32	98.40	98.29	98.35	98.34	98.41
**112**	98.42	98.34	98.41	98.42	98.35	98.37	98.31	98.34	98.38	98.34
**113**	98.31	98.26	98.33	98.34	98.31	98.37	98.29	98.28	98.18	98.33
**114**	98.38	98.32	98.39	98.42	98.28	98.36	98.29	98.35	98.34	98.36
**115**	98.38	98.33	98.41	98.43	98.31	98.40	98.31	98.38	98.34	98.41
**116**	98.43	98.35	98.40	98.44	98.37	98.40	98.30	98.44	98.41	98.47
**117**	98.30	98.24	98.25	98.37	98.30	98.31	98.18	98.30	98.21	98.26
**118**	98.35	98.30	98.34	98.35	98.29	98.38	98.21	98.31	98.23	98.30
**119**	97.01	96.93	96.99	97.01	97.00	97.02	96.97	96.92	97.01	97.02
**120**	97.01	96.98	97.03	97.06	96.96	97.03	96.96	97.03	96.99	97.06
**121**	96.29	96.18	96.09	96.36	96.10	96.17	95.83	95.99	95.98	96.32
**122**	96.99	96.93	96.94	97.05	96.95	96.94	96.98	96.92	96.97	96.97
**123**	97.11	97.14	97.15	97.14	97.15	97.21	97.11	97.18	97.18	97.15
**124**	97.15	97.14	97.09	97.16	97.12	97.23	97.12	97.15	97.14	97.23
**125**	97.16	97.19	97.22	97.19	97.18	97.28	97.14	97.19	97.18	97.24
**126**	97.25	97.21	97.20	97.29	97.19	97.29	97.19	97.27	97.23	97.27

**127**	97.22	97.15	97.13	97.25	97.21	97.28	97.22	97.25	97.22	97.28
**128**	96.82	96.80	96.82	96.89	96.73	96.92	96.80	96.80	96.78	96.84
**129**	97.01	96.97	97.06	97.06	96.97	97.03	96.98	97.03	96.93	97.12
**130**	96.97	96.93	96.96	96.99	97.00	97.02	96.98	96.98	97.07	97.04
**131**	96.85	96.85	96.89	96.91	96.83	96.88	96.92	96.86	96.90	96.91
**132**	96.90	96.90	96.92	96.95	96.88	96.96	96.88	96.87	96.89	96.93
**133**	97.07	97.04	97.05	97.10	97.07	97.12	97.05	97.05	97.11	97.14
**134**	96.89	96.87	96.99	97.00	96.86	97.01	96.79	96.94	96.96	97.00
**135**	96.93	96.86	96.95	97.00	96.88	97.01	96.81	96.91	96.91	96.94
**136**	96.85	96.81	96.89	96.93	96.79	96.99	96.73	96.85	96.85	96.88
**137**	96.38	96.43	96.42	96.43	96.46	96.51	96.43	96.43	96.34	96.45
**138**	96.50	96.44	96.49	96.61	96.49	96.53	96.47	96.52	96.41	96.47
**139**	96.49	96.44	96.53	96.50	96.46	96.56	96.51	96.49	96.46	96.50
**140**	96.56	96.47	96.51	96.52	96.61	96.58	96.52	96.54	96.51	96.53
**141**	96.22	96.17	96.20	96.21	96.23	96.27	96.23	96.18	96.21	96.21
**142**	96.54	96.52	96.49	96.56	96.51	96.56	96.49	96.55	96.54	96.57
**143**	96.63	96.55	96.61	96.61	96.51	96.64	96.52	96.54	96.61	96.56
**144**	95.48	95.37	95.40	95.45	95.41	95.54	95.57	95.48	95.42	95.48
**145**	95.26	95.22	95.21	95.30	95.26	95.29	95.30	95.21	95.21	95.39
**146**	95.62	95.57	95.53	95.65	95.60	95.69	95.71	95.64	95.77	95.72
**147**	96.59	96.56	96.54	96.45	96.59	96.65	96.53	96.50	96.64	96.58
**148**	91.99	91.86	91.94	92.01	91.93	91.91	92.00	91.99	91.96	91.94
**149**	91.97	91.89	91.88	91.99	91.94	92.02	92.04	91.95	91.96	91.94
**150**	91.95	91.83	91.86	91.91	91.91	91.89	91.96	91.89	91.93	91.93
**151**	91.94	91.85	91.84	91.94	91.89	91.98	91.96	91.95	91.92	91.91
**152**	91.94	91.85	91.84	91.94	91.89	91.98	91.96	91.95	91.92	91.91
**153**	91.88	91.83	91.88	91.93	91.86	91.84	91.98	91.86	91.87	91.90
**154**	91.88	91.73	91.77	91.85	91.84	91.88	91.90	91.88	91.82	91.81
**155**	91.84	91.70	91.75	91.84	91.79	91.77	91.81	91.79	91.77	91.83
**156**	91.89	91.78	91.79	91.90	91.83	91.90	91.95	91.84	91.83	91.88
**157**	91.86	91.70	91.78	91.88	91.78	91.86	91.85	91.86	91.78	91.84
**158**	92.15	92.04	92.11	92.12	92.08	92.08	92.20	92.09	92.08	92.14
**159**	94.10	94.00	94.04	94.10	93.97	94.06	94.15	94.09	93.99	94.11
**160**	93.63	93.59	93.52	93.69	93.58	93.49	93.64	93.55	93.58	93.62
**161**	93.60	93.52	93.51	93.59	93.53	93.62	93.61	93.57	93.53	93.61
**162**	83.80	83.67	83.79	83.79	83.79	83.80	83.81	83.76	83.73	83.78
**163**	83.75	83.62	83.74	83.74	83.80	83.80	83.82	83.78	83.72	83.77
**164**	83.82	83.78	83.84	83.87	83.89	83.83	83.88	83.83	83.88	83.75
**165**	83.85	83.82	83.89	83.93	83.93	83.91	83.93	83.90	83.91	83.89
**166**	83.87	83.85	83.96	83.91	83.94	83.95	83.94	83.86	83.91	83.91

The specific names and accession numbers of 166 global *Morganella* isolates are shown in Fig. S1.

Table S3. (continued)

Isolate	11	12	13	14	15	16	17	18	19	20
**1**	99.75	99.69	99.81	99.75	99.66	99.67	99.72	99.70	99.12	98.63
**2**	99.74	99.71	99.78	99.71	99.68	99.60	99.70	99.71	99.07	98.63
**3**	99.75	99.71	99.73	99.90	99.71	99.72	99.68	99.65	99.12	98.61
**4**	99.80	99.72	99.82	99.81	99.72	99.71	99.77	99.80	99.21	98.67
**5**	99.77	99.75	99.73	99.69	99.72	99.73	99.79	99.70	99.09	98.61
**6**	99.76	99.82	99.80	99.82	99.76	99.69	99.74	99.73	99.15	98.66
**7**	99.63	99.57	99.57	99.52	99.60	99.64	99.67	99.54	98.93	98.57
**8**	99.73	99.85	99.71	99.68	99.81	99.78	99.75	99.66	99.10	98.66
**9**	99.75	99.72	99.80	99.86	99.72	99.75	99.75	99.73	99.10	98.60
**10**	98.76	98.72	98.78	98.71	98.68	98.68	98.76	98.71	98.70	98.61
**11**	100.00	99.83	99.78	99.76	99.84	99.79	99.81	99.64	99.17	98.70
**12**	99.83	100.00	99.73	99.75	99.98	99.87	99.93	99.71	99.12	98.61
**13**	99.78	99.73	100.00	99.72	99.72	99.69	99.82	99.83	99.13	98.67
**14**	99.76	99.75	99.72	100.00	99.72	99.73	99.75	99.70	99.11	98.67
**15**	99.84	99.98	99.72	99.72	100.00	99.86	99.90	99.67	99.12	98.61
**16**	99.79	99.87	99.69	99.73	99.86	100.00	99.81	99.58	99.03	98.57
**17**	99.81	99.93	99.82	99.75	99.90	99.81	100.00	99.62	99.14	98.62
**18**	99.64	99.71	99.83	99.70	99.67	99.58	99.62	100.00	99.08	98.60
**19**	99.17	99.12	99.13	99.11	99.12	99.03	99.14	99.08	100.00	98.55
**20**	98.70	98.61	98.67	98.67	98.61	98.57	98.62	98.60	98.55	100.00
**21**	98.63	98.60	98.68	98.65	98.62	98.65	98.66	98.64	98.56	99.98
**22**	98.61	98.58	98.64	98.57	98.57	98.54	98.58	98.60	98.54	99.81
**23**	98.93	98.91	98.93	98.90	98.91	98.87	98.95	98.88	98.89	98.73
**24**	98.89	98.90	98.87	98.87	98.87	98.80	98.87	98.82	98.82	98.73
**25**	98.87	98.89	98.90	98.87	98.90	98.83	98.90	98.89	98.81	98.76
**26**	98.88	98.90	98.89	98.88	98.89	98.87	98.87	98.90	98.85	98.77
**27**	98.77	98.73	98.80	98.79	98.76	98.67	98.73	98.71	98.76	98.82
**28**	98.80	98.76	98.80	98.79	98.75	98.75	98.79	98.76	98.68	98.60
**29**	98.72	98.70	98.71	98.68	98.67	98.71	98.68	98.65	98.74	98.58
**30**	98.77	98.68	98.73	98.71	98.70	98.69	98.70	98.73	98.71	98.61
**31**	98.69	98.63	98.71	98.71	98.65	98.63	98.60	98.57	98.72	98.62
**32**	98.68	98.61	98.66	98.63	98.61	98.59	98.63	98.62	98.67	98.59
**33**	98.74	98.67	98.65	98.60	98.64	98.60	98.66	98.74	98.69	98.53
**34**	98.77	98.77	98.84	98.78	98.76	98.78	98.76	98.78	98.82	98.71
**35**	98.66	98.61	98.71	98.71	98.65	98.62	98.58	98.66	98.70	98.58
**36**	99.75	99.78	99.83	99.77	99.79	99.74	99.77	99.80	99.10	98.64
**37**	98.69	98.67	98.77	98.74	98.67	98.64	98.64	98.63	98.69	98.59
**38**	98.80	98.79	98.85	98.87	98.75	98.71	98.74	98.76	98.81	98.68
**39**	98.82	98.75	98.84	98.82	98.77	98.74	98.74	98.78	98.81	98.72
**40**	98.72	98.71	98.70	98.69	98.67	98.67	98.70	98.66	98.70	98.57
**41**	98.40	98.35	98.37	98.34	98.29	98.22	98.30	98.35	98.43	98.31

**42**	98.31	98.31	98.35	98.37	98.26	98.22	98.27	98.31	98.42	98.39
**43**	98.35	98.35	98.33	98.33	98.31	98.27	98.35	98.38	98.39	98.38
**44**	98.34	98.30	98.33	98.30	98.27	98.24	98.29	98.37	98.37	98.37
**45**	98.42	98.49	98.44	98.38	98.43	98.41	98.43	98.38	98.51	98.39
**46**	98.36	98.33	98.30	98.34	98.27	98.24	98.33	98.34	98.36	98.36
**47**	98.45	98.43	98.43	98.39	98.43	98.34	98.38	98.41	98.46	98.35
**48**	98.48	98.51	98.49	98.46	98.51	98.45	98.47	98.46	98.49	98.46
**49**	98.39	98.36	98.37	98.35	98.34	98.29	98.31	98.35	98.31	98.32
**50**	98.54	98.46	98.51	98.48	98.48	98.47	98.50	98.46	98.50	98.48
**51**	98.47	98.44	98.44	98.42	98.47	98.39	98.42	98.39	98.47	98.31
**52**	98.29	98.23	98.39	98.32	98.25	98.26	98.11	98.29	98.42	98.32
**53**	98.51	98.52	98.53	98.52	98.52	98.46	98.53	98.54	98.55	98.54
**54**	98.45	98.46	98.52	98.45	98.48	98.44	98.48	98.52	98.56	98.50
**55**	98.33	98.28	98.37	98.37	98.27	98.20	98.21	98.31	98.41	98.31
**56**	98.27	98.27	98.32	98.27	98.27	98.26	98.26	98.30	98.26	98.30
**57**	98.47	98.45	98.57	98.51	98.50	98.50	98.47	98.54	98.60	98.46
**58**	98.46	98.48	98.58	98.53	98.48	98.48	98.49	98.53	98.59	98.45
**59**	98.50	98.43	98.46	98.42	98.41	98.39	98.47	98.49	98.41	98.34
**60**	98.50	98.43	98.46	98.42	98.41	98.39	98.47	98.49	98.41	98.34
**61**	98.44	98.53	98.48	98.40	98.47	98.46	98.49	98.46	98.39	98.38
**62**	98.45	98.44	98.50	98.47	98.44	98.41	98.45	98.44	98.48	98.43
**63**	98.50	98.42	98.49	98.51	98.42	98.42	98.44	98.44	98.50	98.47
**64**	98.47	98.41	98.47	98.38	98.38	98.40	98.47	98.41	98.47	98.38
**65**	98.48	98.43	98.46	98.47	98.41	98.39	98.41	98.42	98.46	98.43
**66**	98.42	98.37	98.39	98.40	98.36	98.38	98.38	98.36	98.40	98.36
**67**	98.40	98.33	98.36	98.38	98.35	98.36	98.31	98.30	98.40	98.39
**68**	98.37	98.41	98.45	98.41	98.39	98.39	98.33	98.40	98.44	98.35
**69**	98.43	98.43	98.48	98.39	98.44	98.39	98.35	98.37	98.48	98.49
**70**	98.43	98.42	98.49	98.42	98.42	98.40	98.42	98.39	98.43	98.42
**71**	98.27	98.31	98.35	98.25	98.30	98.28	98.22	98.35	98.39	98.27
**72**	98.37	98.37	98.35	98.34	98.43	98.29	98.27	98.22	98.43	98.38
**73**	98.49	98.47	98.53	98.49	98.44	98.46	98.48	98.50	98.52	98.49
**74**	98.36	98.37	98.41	98.33	98.36	98.34	98.26	98.29	98.41	98.44
**75**	98.49	98.52	98.54	98.53	98.50	98.47	98.49	98.51	98.56	98.44
**76**	98.38	98.40	98.39	98.40	98.37	98.33	98.35	98.41	98.46	98.36
**77**	98.36	98.30	98.37	98.40	98.31	98.26	98.27	98.31	98.39	98.35
**78**	98.37	98.39	98.47	98.45	98.41	98.38	98.41	98.40	98.48	98.40
**79**	98.43	98.45	98.43	98.43	98.44	98.43	98.44	98.35	98.43	98.36
**80**	98.38	98.39	98.42	98.38	98.38	98.34	98.38	98.38	98.43	98.43
**81**	98.44	98.47	98.48	98.47	98.48	98.38	98.43	98.41	98.47	98.35
**82**	98.35	98.30	98.34	98.34	98.29	98.32	98.38	98.36	98.35	98.24
**83**	98.25	98.28	98.34	98.27	98.30	98.28	98.25	98.33	98.38	98.32
**84**	98.44	98.38	98.38	98.35	98.33	98.30	98.40	98.34	98.35	98.28

**85**	98.41	98.34	98.48	98.41	98.38	98.31	98.42	98.44	98.44	98.35
**86**	98.42	98.43	98.49	98.38	98.40	98.37	98.43	98.39	98.44	98.36
**87**	98.36	98.36	98.44	98.32	98.34	98.31	98.34	98.35	98.41	98.31
**88**	98.39	98.39	98.43	98.44	98.40	98.35	98.38	98.43	98.44	98.46
**89**	98.37	98.31	98.43	98.40	98.33	98.33	98.37	98.42	98.46	98.46
**90**	98.40	98.39	98.40	98.34	98.35	98.34	98.42	98.38	98.35	98.36
**91**	98.37	98.32	98.32	98.28	98.28	98.24	98.33	98.31	98.33	98.32
**92**	98.44	98.36	98.41	98.36	98.35	98.32	98.42	98.41	98.34	98.36
**93**	98.38	98.31	98.31	98.34	98.29	98.21	98.32	98.35	98.36	98.30
**94**	98.35	98.27	98.29	98.30	98.25	98.23	98.24	98.24	98.37	98.33
**95**	98.50	98.48	98.49	98.51	98.48	98.45	98.44	98.42	98.55	98.48
**96**	98.47	98.49	98.52	98.51	98.50	98.47	98.49	98.45	98.53	98.43
**97**	98.49	98.48	98.45	98.42	98.46	98.43	98.46	98.41	98.46	98.39
**98**	98.53	98.57	98.61	98.55	98.53	98.48	98.55	98.53	98.62	98.49
**99**	98.51	98.50	98.51	98.50	98.53	98.36	98.47	98.52	98.53	98.48
**100**	98.44	98.42	98.47	98.43	98.41	98.38	98.45	98.46	98.49	98.39
**101**	98.42	98.41	98.47	98.41	98.40	98.34	98.42	98.41	98.42	98.36
**102**	98.50	98.43	98.47	98.45	98.44	98.46	98.46	98.43	98.46	98.44
**103**	98.44	98.47	98.44	98.43	98.44	98.46	98.44	98.40	98.51	98.39
**104**	98.40	98.41	98.40	98.35	98.37	98.36	98.39	98.32	98.37	98.28
**105**	98.48	98.43	98.50	98.49	98.43	98.46	98.42	98.49	98.51	98.41
**106**	98.50	98.47	98.51	98.49	98.52	98.48	98.49	98.53	98.58	98.48
**107**	98.31	98.32	98.37	98.38	98.31	98.26	98.28	98.29	98.35	98.26
**108**	98.38	98.29	98.38	98.35	98.35	98.29	98.29	98.25	98.34	98.35
**109**	98.51	98.43	98.46	98.42	98.43	98.44	98.45	98.43	98.47	98.42
**110**	98.35	98.31	98.36	98.36	98.36	98.37	98.33	98.31	98.37	98.34
**111**	98.47	98.39	98.42	98.40	98.42	98.40	98.40	98.36	98.42	98.35
**112**	98.36	98.38	98.43	98.39	98.38	98.36	98.36	98.27	98.41	98.37
**113**	98.28	98.36	98.34	98.38	98.34	98.32	98.28	98.25	98.39	98.40
**114**	98.40	98.37	98.39	98.37	98.39	98.46	98.37	98.29	98.37	98.35
**115**	98.41	98.41	98.39	98.42	98.43	98.40	98.42	98.31	98.37	98.42
**116**	98.45	98.44	98.42	98.42	98.40	98.37	98.39	98.36	98.44	98.39
**117**	98.33	98.25	98.27	98.26	98.24	98.16	98.22	98.32	98.30	98.23
**118**	98.36	98.31	98.32	98.29	98.29	98.30	98.31	98.28	98.32	98.27
**119**	96.94	96.98	97.02	97.01	96.93	96.97	96.97	96.93	97.02	96.99
**120**	97.00	97.05	97.03	97.01	97.02	97.08	97.01	97.00	97.01	96.96
**121**	96.24	96.21	96.33	96.34	96.27	95.95	96.14	96.03	96.36	96.15
**122**	96.96	96.96	97.03	96.92	96.94	96.95	96.98	97.00	96.98	96.82
**123**	97.21	97.08	97.16	97.18	97.15	97.10	97.12	97.19	97.17	97.17
**124**	97.16	97.13	97.20	97.14	97.17	97.13	97.20	97.17	97.17	97.17
**125**	97.21	97.21	97.19	97.16	97.21	97.25	97.19	97.19	97.21	97.17
**126**	97.26	97.26	97.28	97.22	97.26	97.27	97.30	97.27	97.25	97.19
**127**	97.23	97.20	97.24	97.20	97.20	97.26	97.21	97.25	97.28	97.21

**128**	96.87	96.86	96.90	96.77	96.82	96.84	96.76	96.77	96.86	96.91
**129**	97.05	96.99	97.05	97.02	96.98	96.99	96.99	96.97	97.01	97.10
**130**	97.02	97.06	96.96	96.97	97.11	97.05	97.04	96.99	97.08	97.15
**131**	96.91	96.97	96.93	96.93	96.92	96.94	96.95	96.83	96.88	97.00
**132**	96.97	96.93	96.97	96.90	96.88	96.84	96.92	96.92	96.98	97.03
**133**	97.06	97.11	97.10	97.08	97.10	97.06	97.09	97.11	97.10	97.17
**134**	96.99	96.95	96.99	96.93	96.89	96.93	96.95	96.96	96.98	97.07
**135**	96.98	96.96	96.99	96.91	96.92	96.95	96.93	96.94	96.96	97.08
**136**	96.92	96.82	96.92	96.84	96.82	96.92	96.89	96.86	96.93	96.98
**137**	96.49	96.43	96.43	96.50	96.44	96.46	96.39	96.47	96.47	96.49
**138**	96.58	96.51	96.56	96.52	96.52	96.53	96.52	96.46	96.53	96.40
**139**	96.51	96.51	96.53	96.49	96.51	96.46	96.48	96.54	96.52	96.45
**140**	96.55	96.54	96.56	96.55	96.52	96.53	96.51	96.56	96.60	96.51
**141**	96.16	96.21	96.24	96.20	96.17	96.25	96.20	96.24	96.28	96.19
**142**	96.61	96.53	96.55	96.53	96.53	96.52	96.55	96.52	96.56	96.59
**143**	96.63	96.62	96.62	96.63	96.62	96.61	96.60	96.57	96.68	96.57
**144**	95.46	95.49	95.51	95.44	95.50	95.54	95.48	95.45	95.61	95.46
**145**	95.28	95.31	95.32	95.27	95.34	95.40	95.22	95.30	95.46	95.30
**146**	95.68	95.71	95.62	95.64	95.73	95.74	95.68	95.57	95.69	95.62
**147**	96.55	96.58	96.56	96.50	96.64	96.50	96.36	96.55	96.51	96.52
**148**	91.95	91.98	91.96	91.92	91.90	91.99	91.95	91.95	91.95	91.87
**149**	91.98	92.01	92.01	91.95	91.97	92.05	91.98	92.00	91.98	91.92
**150**	91.88	91.93	91.92	91.91	91.91	91.99	91.92	91.91	91.86	91.93
**151**	91.94	91.98	91.97	91.92	91.95	91.93	91.96	92.00	91.96	91.85
**152**	91.94	91.98	91.97	91.92	91.95	91.93	91.96	92.00	91.96	91.85
**153**	91.89	91.87	91.90	91.94	91.89	91.90	91.88	91.89	91.87	91.90
**154**	91.85	91.87	91.86	91.82	91.84	91.91	91.82	91.87	91.77	91.80
**155**	91.77	91.81	91.87	91.78	91.81	91.86	91.83	91.86	91.82	91.81
**156**	91.88	91.86	91.89	91.84	91.88	91.92	91.86	91.89	91.92	91.86
**157**	91.92	91.83	91.87	91.92	91.90	91.91	91.81	91.85	91.90	91.82
**158**	92.19	92.14	92.14	92.07	92.10	92.16	92.17	92.13	92.13	92.10
**159**	94.10	94.09	94.12	94.02	94.04	94.10	94.02	94.03	94.06	94.01
**160**	93.58	93.56	93.67	93.65	93.59	93.64	93.61	93.48	93.70	93.52
**161**	93.52	93.57	93.60	93.52	93.53	93.61	93.55	93.57	93.64	93.50
**162**	83.78	83.79	83.81	83.82	83.79	83.79	83.79	83.83	83.79	83.75
**163**	83.80	83.79	83.81	83.84	83.76	83.80	83.75	83.86	83.82	83.75
**164**	83.88	83.87	83.86	83.86	83.78	83.85	83.86	83.89	83.76	83.86
**165**	83.90	83.94	83.97	83.81	83.92	83.97	83.86	83.94	83.86	83.89
**166**	83.86	83.85	83.90	83.89	83.87	83.97	83.87	83.86	83.81	83.89

Table S3. (continued)

**Isolate**	**21**	**22**	**23**	**24**	**25**	**26**	**27**	**28**	**29**	**30**
**1**	98.66	98.66	98.93	98.88	98.92	98.87	98.75	98.77	98.65	98.66
**2**	98.67	98.61	98.91	98.87	98.89	98.87	98.68	98.77	98.68	98.70
**3**	98.61	98.58	98.89	98.86	98.88	98.92	98.70	98.76	98.68	98.73
**4**	98.71	98.72	98.96	98.88	98.90	98.92	98.79	98.83	98.78	98.78
**5**	98.66	98.58	98.95	98.85	98.90	98.91	98.74	98.72	98.64	98.65
**6**	98.68	98.61	98.94	98.89	98.91	98.94	98.77	98.78	98.76	98.73
**7**	98.53	98.47	98.82	98.76	98.83	98.83	98.64	98.68	98.58	98.61
**8**	98.68	98.63	98.91	98.87	98.89	98.86	98.69	98.75	98.64	98.70
**9**	98.64	98.56	98.90	98.81	98.88	98.93	98.70	98.66	98.68	98.67
**10**	98.63	98.63	98.84	98.75	98.82	98.85	98.78	98.82	98.78	98.78
**11**	98.63	98.61	98.93	98.89	98.87	98.88	98.77	98.80	98.72	98.77
**12**	98.60	98.58	98.91	98.90	98.89	98.90	98.73	98.76	98.70	98.68
**13**	98.68	98.64	98.93	98.87	98.90	98.89	98.80	98.80	98.71	98.73
**14**	98.65	98.57	98.90	98.87	98.87	98.88	98.79	98.79	98.68	98.71
**15**	98.62	98.57	98.91	98.87	98.90	98.89	98.76	98.75	98.67	98.70
**16**	98.65	98.54	98.87	98.80	98.83	98.87	98.67	98.75	98.71	98.69
**17**	98.66	98.58	98.95	98.87	98.90	98.87	98.73	98.79	98.68	98.70
**18**	98.64	98.60	98.88	98.82	98.89	98.90	98.71	98.76	98.65	98.73
**19**	98.56	98.54	98.89	98.82	98.81	98.85	98.76	98.68	98.74	98.71
**20**	99.98	99.81	98.73	98.73	98.76	98.77	98.82	98.60	98.58	98.61
**21**	100.00	99.85	98.76	98.75	98.80	98.79	98.88	98.66	98.62	98.60
**22**	99.85	100.00	98.74	98.70	98.81	98.79	98.83	98.69	98.61	98.57
**23**	98.76	98.74	100.00	98.85	98.91	98.95	99.12	98.88	98.91	98.89
**24**	98.75	98.70	98.85	100.00	99.17	98.89	98.77	98.79	98.81	98.76
**25**	98.80	98.81	98.91	99.17	100.00	98.92	98.85	98.85	98.88	98.84
**26**	98.79	98.79	98.95	98.89	98.92	100.00	98.82	98.80	98.82	98.78
**27**	98.88	98.83	99.12	98.77	98.85	98.82	100.00	98.79	98.82	98.80
**28**	98.66	98.69	98.88	98.79	98.85	98.80	98.79	100.00	99.84	99.80
**29**	98.62	98.61	98.91	98.81	98.88	98.82	98.82	99.84	100.00	99.78
**30**	98.60	98.57	98.89	98.76	98.84	98.78	98.80	99.80	99.78	100.00
**31**	98.59	98.54	98.90	98.78	98.85	98.77	98.79	99.84	99.96	99.73
**32**	98.56	98.48	98.77	98.72	98.85	98.78	98.71	98.69	98.61	98.68
**33**	98.59	98.55	98.77	98.75	98.85	98.74	98.73	98.71	98.65	98.67
**34**	98.73	98.74	98.92	98.82	98.86	98.82	98.91	98.87	98.82	98.87
**35**	98.59	98.60	98.76	98.80	98.86	98.78	98.61	98.77	98.66	98.62
**36**	98.64	98.65	98.91	98.90	98.89	98.93	98.75	98.75	98.61	98.70
**37**	98.61	98.57	98.86	98.82	98.88	98.85	98.69	98.72	98.64	98.67
**38**	98.72	98.65	98.98	98.83	98.91	98.91	98.85	98.75	98.80	98.86
**39**	98.74	98.69	98.92	98.78	98.89	98.90	98.86	98.79	98.79	98.84
**40**	98.59	98.58	98.81	98.84	98.93	98.86	98.75	98.73	98.70	98.65
**41**	98.29	98.26	98.46	98.45	98.58	98.52	98.38	98.33	98.39	98.38

**42**	98.37	98.35	98.45	98.44	98.57	98.47	98.42	98.37	98.38	98.36
**43**	98.36	98.34	98.47	98.42	98.56	98.44	98.42	98.37	98.41	98.40
**44**	98.35	98.31	98.45	98.45	98.57	98.43	98.41	98.38	98.32	98.36
**45**	98.37	98.44	98.51	98.48	98.58	98.49	98.48	98.44	98.51	98.47
**46**	98.35	98.35	98.46	98.43	98.57	98.49	98.46	98.40	98.40	98.33
**47**	98.35	98.28	98.47	98.54	98.60	98.48	98.41	98.39	98.39	98.33
**48**	98.43	98.46	98.51	98.51	98.58	98.58	98.48	98.39	98.49	98.48
**49**	98.33	98.28	98.42	98.47	98.62	98.47	98.38	98.31	98.32	98.29
**50**	98.49	98.50	98.50	98.56	98.64	98.57	98.54	98.46	98.49	98.48
**51**	98.31	98.31	98.46	98.55	98.61	98.49	98.42	98.40	98.37	98.40
**52**	98.35	98.29	98.38	98.40	98.54	98.49	98.33	98.31	98.22	98.27
**53**	98.54	98.49	98.56	98.62	98.67	98.56	98.50	98.53	98.53	98.53
**54**	98.48	98.42	98.59	98.64	98.65	98.56	98.56	98.52	98.47	98.49
**55**	98.34	98.29	98.46	98.53	98.58	98.48	98.37	98.38	98.23	98.32
**56**	98.34	98.31	98.40	98.48	98.60	98.41	98.42	98.37	98.21	98.26
**57**	98.46	98.44	98.54	98.63	98.61	98.61	98.54	98.49	98.46	98.52
**58**	98.47	98.45	98.55	98.63	98.61	98.62	98.55	98.49	98.48	98.51
**59**	98.35	98.33	98.54	98.56	98.64	98.53	98.42	98.40	98.43	98.35
**60**	98.35	98.33	98.54	98.56	98.64	98.53	98.42	98.40	98.43	98.35
**61**	98.38	98.41	98.56	98.53	98.67	98.55	98.43	98.40	98.43	98.37
**62**	98.38	98.38	98.52	98.53	98.66	98.52	98.42	98.38	98.44	98.44
**63**	98.48	98.41	98.50	98.54	98.65	98.53	98.47	98.35	98.39	98.34
**64**	98.37	98.36	98.57	98.51	98.65	98.54	98.41	98.42	98.32	98.40
**65**	98.42	98.39	98.50	98.52	98.66	98.51	98.46	98.36	98.37	98.34
**66**	98.30	98.30	98.47	98.50	98.62	98.48	98.36	98.44	98.40	98.38
**67**	98.31	98.32	98.41	98.54	98.62	98.49	98.39	98.33	98.36	98.33
**68**	98.36	98.31	98.46	98.55	98.65	98.48	98.39	98.39	98.34	98.39
**69**	98.46	98.38	98.38	98.46	98.62	98.45	98.54	98.31	98.34	98.39
**70**	98.42	98.40	98.49	98.54	98.62	98.52	98.49	98.30	98.39	98.32
**71**	98.32	98.21	98.42	98.45	98.66	98.46	98.39	98.34	98.33	98.32
**72**	98.30	98.21	98.34	98.47	98.63	98.42	98.37	98.30	98.38	98.35
**73**	98.51	98.47	98.49	98.54	98.66	98.55	98.55	98.44	98.48	98.45
**74**	98.40	98.32	98.45	98.51	98.65	98.43	98.42	98.30	98.25	98.31
**75**	98.48	98.50	98.53	98.56	98.68	98.55	98.47	98.49	98.53	98.50
**76**	98.39	98.35	98.44	98.54	98.65	98.51	98.37	98.44	98.35	98.36
**77**	98.34	98.27	98.39	98.55	98.64	98.49	98.35	98.41	98.31	98.36
**78**	98.41	98.38	98.52	98.50	98.65	98.52	98.54	98.45	98.51	98.54
**79**	98.37	98.36	98.45	98.54	98.63	98.53	98.46	98.41	98.44	98.38
**80**	98.45	98.37	98.46	98.44	98.53	98.46	98.42	98.38	98.44	98.48
**81**	98.38	98.38	98.49	98.55	98.69	98.56	98.41	98.44	98.44	98.42
**82**	98.28	98.23	98.36	98.44	98.54	98.47	98.33	98.37	98.36	98.32
**83**	98.29	98.28	98.47	98.47	98.64	98.45	98.37	98.39	98.22	98.33
**84**	98.35	98.29	98.45	98.53	98.66	98.51	98.39	98.41	98.34	98.33

**85**	98.35	98.33	98.44	98.52	98.63	98.49	98.39	98.33	98.38	98.29
**86**	98.38	98.33	98.55	98.53	98.68	98.53	98.43	98.42	98.42	98.41
**87**	98.35	98.29	98.43	98.52	98.63	98.45	98.38	98.36	98.34	98.32
**88**	98.43	98.39	98.50	98.58	98.72	98.55	98.48	98.47	98.44	98.39
**89**	98.46	98.43	98.46	98.59	98.72	98.51	98.50	98.42	98.31	98.35
**90**	98.36	98.29	98.49	98.47	98.62	98.46	98.44	98.40	98.39	98.38
**91**	98.31	98.24	98.41	98.45	98.57	98.42	98.38	98.35	98.36	98.33
**92**	98.36	98.35	98.51	98.51	98.65	98.49	98.44	98.42	98.39	98.35
**93**	98.30	98.29	98.48	98.45	98.59	98.48	98.32	98.36	98.39	98.36
**94**	98.27	98.25	98.46	98.49	98.61	98.43	98.38	98.35	98.33	98.31
**95**	98.50	98.47	98.55	98.67	98.72	98.57	98.52	98.49	98.45	98.50
**96**	98.46	98.48	98.62	98.55	98.71	98.60	98.58	98.48	98.55	98.51
**97**	98.41	98.39	98.52	98.56	98.70	98.55	98.49	98.43	98.45	98.38
**98**	98.52	98.50	98.62	98.67	98.73	98.63	98.61	98.61	98.58	98.60
**99**	98.42	98.40	98.53	98.62	98.73	98.58	98.51	98.46	98.54	98.54
**100**	98.42	98.35	98.51	98.57	98.72	98.57	98.48	98.46	98.49	98.44
**101**	98.34	98.32	98.51	98.59	98.72	98.55	98.50	98.40	98.45	98.34
**102**	98.38	98.39	98.55	98.62	98.73	98.54	98.50	98.50	98.46	98.49
**103**	98.37	98.42	98.51	98.50	98.67	98.51	98.51	98.41	98.44	98.48
**104**	98.35	98.32	98.45	98.42	98.64	98.50	98.43	98.38	98.35	98.33
**105**	98.36	98.35	98.53	98.64	98.67	98.58	98.47	98.51	98.41	98.37
**106**	98.42	98.40	98.55	98.63	98.66	98.59	98.52	98.50	98.51	98.45
**107**	98.30	98.35	98.42	98.50	98.63	98.49	98.35	98.41	98.37	98.34
**108**	98.33	98.35	98.39	98.53	98.60	98.44	98.40	98.40	98.34	98.23
**109**	98.41	98.41	98.56	98.55	98.63	98.52	98.57	98.45	98.49	98.44
**110**	98.39	98.41	98.40	98.48	98.60	98.50	98.44	98.42	98.38	98.36
**111**	98.39	98.43	98.45	98.53	98.62	98.51	98.43	98.38	98.43	98.38
**112**	98.38	98.46	98.41	98.51	98.61	98.52	98.42	98.42	98.41	98.36
**113**	98.38	98.46	98.42	98.49	98.58	98.53	98.44	98.46	98.42	98.34
**114**	98.36	98.45	98.41	98.47	98.61	98.49	98.47	98.37	98.47	98.34
**115**	98.42	98.49	98.43	98.49	98.61	98.53	98.46	98.45	98.52	98.33
**116**	98.40	98.43	98.51	98.52	98.62	98.49	98.54	98.44	98.42	98.43
**117**	98.30	98.18	98.39	98.39	98.50	98.41	98.29	98.39	98.24	98.31
**118**	98.29	98.25	98.36	98.45	98.52	98.40	98.32	98.29	98.29	98.29
**119**	97.02	97.06	97.03	97.02	97.06	97.05	97.01	96.98	97.00	97.03
**120**	97.03	96.96	97.01	97.00	97.09	97.03	97.01	96.95	96.98	97.02
**121**	96.07	96.15	96.31	96.12	96.42	96.14	95.98	96.07	96.14	96.21
**122**	96.90	96.88	97.04	96.95	97.03	96.95	96.89	97.00	96.90	96.95
**123**	97.13	97.13	97.20	97.26	97.36	97.19	97.15	97.19	97.14	97.15
**124**	97.20	97.18	97.23	97.29	97.39	97.20	97.19	97.21	97.17	97.11
**125**	97.19	97.21	97.21	97.26	97.38	97.27	97.25	97.20	97.20	97.17
**126**	97.19	97.25	97.32	97.26	97.35	97.27	97.26	97.27	97.26	97.21
**127**	97.21	97.21	97.24	97.28	97.32	97.23	97.24	97.23	97.22	97.22

**128**	96.91	96.91	96.96	97.00	97.07	96.98	96.85	96.90	96.79	96.80
**129**	97.15	97.16	97.03	97.09	97.15	97.05	97.03	97.06	96.94	96.97
**130**	97.15	97.13	97.04	97.03	97.13	97.03	97.00	96.98	97.07	97.01
**131**	96.98	97.08	96.95	97.07	97.16	97.02	96.94	97.02	96.97	96.83
**132**	97.04	97.08	97.03	97.00	97.11	97.04	97.01	97.01	96.95	96.95
**133**	97.17	97.15	97.11	97.14	97.18	97.08	97.14	97.13	97.08	97.08
**134**	97.10	97.10	97.05	97.08	97.16	97.07	96.95	97.02	96.92	96.95
**135**	97.11	97.12	97.04	97.08	97.14	97.07	96.95	96.99	96.87	96.91
**136**	97.01	97.05	96.95	96.99	97.08	96.95	96.90	96.93	96.82	96.84
**137**	96.42	96.42	96.48	96.59	96.65	96.53	96.46	96.51	96.37	96.48
**138**	96.44	96.51	96.53	96.60	96.62	96.57	96.48	96.55	96.48	96.43
**139**	96.47	96.49	96.57	96.61	96.65	96.65	96.51	96.57	96.50	96.52
**140**	96.52	96.55	96.59	96.68	96.66	96.61	96.56	96.53	96.54	96.56
**141**	96.21	96.23	96.27	96.32	96.34	96.29	96.20	96.24	96.21	96.19
**142**	96.57	96.51	96.65	96.67	96.69	96.66	96.55	96.63	96.52	96.53
**143**	96.60	96.50	96.67	96.63	96.68	96.62	96.59	96.55	96.60	96.55
**144**	95.44	95.45	95.43	95.53	95.60	95.47	95.46	95.46	95.49	95.45
**145**	95.30	95.29	95.38	95.41	95.48	95.36	95.30	95.36	95.33	95.38
**146**	95.62	95.66	95.71	95.79	95.80	95.69	95.67	95.67	95.83	95.75
**147**	96.44	96.46	96.63	96.58	96.58	96.39	96.50	96.62	96.52	96.58
**148**	91.93	91.94	91.91	91.97	91.92	91.93	91.92	91.89	91.95	91.89
**149**	91.93	91.91	92.01	91.96	91.97	91.96	91.98	91.92	92.00	91.89
**150**	91.93	91.98	91.89	91.88	91.93	91.95	91.89	91.94	91.97	91.85
**151**	91.89	91.87	91.94	91.92	91.91	91.91	91.93	91.88	91.95	91.93
**152**	91.89	91.87	91.94	91.92	91.91	91.91	91.93	91.88	91.95	91.93
**153**	91.88	91.95	91.87	91.96	91.92	91.86	91.84	91.94	91.84	91.80
**154**	91.80	91.83	91.87	91.84	91.81	91.81	91.83	91.77	91.80	91.78
**155**	91.87	91.87	91.85	91.88	91.85	91.83	91.80	91.86	91.80	91.74
**156**	91.86	91.89	91.91	91.91	91.89	91.86	91.85	91.88	91.87	91.85
**157**	91.73	91.78	91.84	91.91	91.84	91.83	91.80	91.86	91.81	91.84
**158**	92.09	92.17	92.12	92.22	92.23	92.19	92.09	92.14	92.16	92.05
**159**	94.05	94.03	94.12	94.21	94.25	94.11	94.03	94.13	94.01	94.01
**160**	93.54	93.54	93.59	93.72	93.80	93.70	93.60	93.72	93.66	93.55
**161**	93.52	93.56	93.60	93.67	93.68	93.59	93.58	93.56	93.51	93.56
**162**	83.83	83.81	83.80	83.82	83.90	83.82	83.78	83.82	83.71	83.73
**163**	83.76	83.75	83.82	83.89	83.87	83.80	83.78	83.78	83.70	83.79
**164**	83.93	83.92	83.86	83.85	83.91	83.86	83.82	83.86	83.85	83.88
**165**	83.90	83.87	83.88	83.95	84.04	83.86	83.85	84.05	83.87	83.88
**166**	83.98	83.97	83.87	83.93	84.04	83.83	83.87	83.91	83.91	83.91

Table S3. (continued)

Isolate	31	32	33	34	35	36	37	38	39	40
**1**	98.57	98.58	98.66	98.80	98.68	99.79	98.73	98.73	98.71	98.73
**2**	98.61	98.63	98.68	98.77	98.71	99.85	98.61	98.75	98.78	98.71
**3**	98.66	98.69	98.63	98.75	98.70	99.75	98.70	98.80	98.77	98.70
**4**	98.68	98.59	98.68	98.80	98.69	99.86	98.79	98.83	98.78	98.77
**5**	98.61	98.60	98.58	98.78	98.64	99.76	98.63	98.85	98.79	98.62
**6**	98.73	98.70	98.75	98.85	98.69	99.76	98.76	98.88	98.89	98.71
**7**	98.54	98.50	98.54	98.73	98.51	99.55	98.57	98.63	98.70	98.54
**8**	98.60	98.58	98.63	98.73	98.58	99.75	98.61	98.74	98.77	98.70
**9**	98.59	98.61	98.65	98.80	98.59	99.81	98.63	98.80	98.76	98.70
**10**	98.78	98.83	98.88	99.02	98.89	98.84	98.66	98.83	98.82	98.75
**11**	98.69	98.68	98.74	98.77	98.66	99.75	98.69	98.80	98.82	98.72
**12**	98.63	98.61	98.67	98.77	98.61	99.78	98.67	98.79	98.75	98.71
**13**	98.71	98.66	98.65	98.84	98.71	99.83	98.77	98.85	98.84	98.70
**14**	98.71	98.63	98.60	98.78	98.71	99.77	98.74	98.87	98.82	98.69
**15**	98.65	98.61	98.64	98.76	98.65	99.79	98.67	98.75	98.77	98.67
**16**	98.63	98.59	98.60	98.78	98.62	99.74	98.64	98.71	98.74	98.67
**17**	98.60	98.63	98.66	98.76	98.58	99.77	98.64	98.74	98.74	98.70
**18**	98.57	98.62	98.74	98.78	98.66	99.80	98.63	98.76	98.78	98.66
**19**	98.72	98.67	98.69	98.82	98.70	99.10	98.69	98.81	98.81	98.70
**20**	98.62	98.59	98.53	98.71	98.58	98.64	98.59	98.68	98.72	98.57
**21**	98.59	98.56	98.59	98.73	98.59	98.64	98.61	98.72	98.74	98.59
**22**	98.54	98.48	98.55	98.74	98.60	98.65	98.57	98.65	98.69	98.58
**23**	98.90	98.77	98.77	98.92	98.76	98.91	98.86	98.98	98.92	98.81
**24**	98.78	98.72	98.75	98.82	98.80	98.90	98.82	98.83	98.78	98.84
**25**	98.85	98.85	98.85	98.86	98.86	98.89	98.88	98.91	98.89	98.93
**26**	98.77	98.78	98.74	98.82	98.78	98.93	98.85	98.91	98.90	98.86
**27**	98.79	98.71	98.73	98.91	98.61	98.75	98.69	98.85	98.86	98.75
**28**	99.84	98.69	98.71	98.87	98.77	98.75	98.72	98.75	98.79	98.73
**29**	99.96	98.61	98.65	98.82	98.66	98.61	98.64	98.80	98.79	98.70
**30**	99.73	98.68	98.67	98.87	98.62	98.70	98.67	98.86	98.84	98.65
**31**	100.00	98.61	98.63	98.81	98.57	98.67	98.68	98.75	98.80	98.69
**32**	98.61	100.00	99.85	99.92	99.69	98.73	98.52	98.69	98.73	98.68
**33**	98.63	99.85	100.00	99.93	99.86	98.72	98.61	98.75	98.77	98.65
**34**	98.81	99.92	99.93	100.00	99.84	98.79	98.76	98.85	98.86	98.79
**35**	98.57	99.69	99.86	99.84	100.00	98.66	98.53	98.61	98.65	98.67
**36**	98.67	98.73	98.72	98.79	98.66	100.00	98.76	98.80	98.83	98.71
**37**	98.68	98.52	98.61	98.76	98.53	98.76	100.00	98.67	98.65	98.72
**38**	98.75	98.69	98.75	98.85	98.61	98.80	98.67	100.00	99.77	98.78
**39**	98.80	98.73	98.77	98.86	98.65	98.83	98.65	99.77	100.00	98.74
**40**	98.69	98.68	98.65	98.79	98.67	98.71	98.72	98.78	98.74	100.00
**41**	98.31	98.25	98.31	98.48	98.20	98.35	98.15	98.35	98.37	98.44

**42**	98.30	98.30	98.34	98.41	98.15	98.39	98.14	98.33	98.34	98.45
**43**	98.34	98.31	98.33	98.44	98.31	98.36	98.19	98.36	98.38	98.47
**44**	98.35	98.27	98.31	98.43	98.30	98.36	98.19	98.35	98.37	98.41
**45**	98.44	98.42	98.41	98.47	98.35	98.42	98.33	98.46	98.43	98.61
**46**	98.34	98.27	98.27	98.46	98.28	98.40	98.21	98.35	98.37	98.46
**47**	98.35	98.35	98.33	98.50	98.28	98.41	98.22	98.32	98.42	98.52
**48**	98.47	98.48	98.49	98.53	98.45	98.49	98.41	98.46	98.46	98.58
**49**	98.31	98.28	98.24	98.42	98.21	98.34	98.24	98.37	98.38	98.46
**50**	98.48	98.48	98.47	98.56	98.48	98.52	98.42	98.53	98.57	98.55
**51**	98.39	98.38	98.39	98.48	98.32	98.40	98.25	98.38	98.44	98.48
**52**	98.27	98.23	98.31	98.37	98.17	98.34	98.25	98.35	98.21	98.45
**53**	98.49	98.51	98.54	98.56	98.54	98.56	98.45	98.55	98.53	98.61
**54**	98.49	98.38	98.43	98.51	98.39	98.51	98.51	98.55	98.47	98.59
**55**	98.31	98.25	98.29	98.43	98.28	98.39	98.25	98.35	98.31	98.49
**56**	98.16	98.20	98.28	98.42	98.29	98.30	98.19	98.33	98.30	98.38
**57**	98.42	98.47	98.52	98.47	98.42	98.53	98.42	98.46	98.49	98.60
**58**	98.45	98.46	98.52	98.49	98.43	98.52	98.43	98.46	98.50	98.60
**59**	98.36	98.36	98.35	98.53	98.37	98.48	98.31	98.45	98.44	98.47
**60**	98.36	98.36	98.35	98.53	98.37	98.48	98.31	98.45	98.44	98.47
**61**	98.32	98.38	98.37	98.52	98.34	98.47	98.27	98.40	98.48	98.48
**62**	98.45	98.44	98.43	98.53	98.34	98.38	98.30	98.42	98.42	98.54
**63**	98.37	98.37	98.38	98.53	98.41	98.47	98.32	98.38	98.47	98.48
**64**	98.37	98.35	98.37	98.48	98.37	98.47	98.30	98.43	98.44	98.43
**65**	98.36	98.32	98.35	98.48	98.34	98.46	98.35	98.38	98.44	98.47
**66**	98.29	98.29	98.27	98.47	98.33	98.41	98.23	98.30	98.31	98.41
**67**	98.36	98.33	98.32	98.45	98.24	98.31	98.23	98.34	98.35	98.41
**68**	98.34	98.32	98.36	98.46	98.36	98.38	98.33	98.40	98.39	98.49
**69**	98.32	98.32	98.37	98.50	98.24	98.31	98.36	98.43	98.39	98.54
**70**	98.41	98.35	98.34	98.49	98.29	98.45	98.36	98.45	98.46	98.51
**71**	98.20	98.23	98.29	98.42	98.25	98.26	98.20	98.32	98.35	98.41
**72**	98.31	98.21	98.27	98.42	98.19	98.27	98.26	98.35	98.38	98.42
**73**	98.47	98.40	98.43	98.55	98.44	98.47	98.44	98.50	98.44	98.57
**74**	98.25	98.25	98.34	98.42	98.16	98.35	98.27	98.35	98.38	98.46
**75**	98.55	98.48	98.48	98.59	98.42	98.47	98.43	98.51	98.46	98.59
**76**	98.28	98.34	98.37	98.48	98.40	98.46	98.26	98.35	98.35	98.48
**77**	98.27	98.28	98.32	98.45	98.39	98.43	98.17	98.35	98.31	98.45
**78**	98.45	98.33	98.37	98.51	98.33	98.46	98.37	98.49	98.48	98.57
**79**	98.42	98.37	98.37	98.53	98.37	98.43	98.33	98.45	98.43	98.43
**80**	98.43	98.42	98.42	98.47	98.34	98.41	98.31	98.47	98.44	98.52
**81**	98.42	98.42	98.44	98.57	98.40	98.42	98.40	98.41	98.50	98.54
**82**	98.30	98.25	98.25	98.44	98.31	98.35	98.28	98.29	98.32	98.49
**83**	98.20	98.30	98.26	98.39	98.25	98.27	98.33	98.34	98.39	98.40
**84**	98.32	98.31	98.31	98.42	98.32	98.41	98.25	98.36	98.41	98.42

**85**	98.30	98.35	98.41	98.45	98.36	98.47	98.30	98.36	98.31	98.45
**86**	98.41	98.31	98.32	98.53	98.33	98.39	98.32	98.40	98.40	98.51
**87**	98.33	98.32	98.33	98.46	98.31	98.37	98.36	98.39	98.36	98.50
**88**	98.40	98.41	98.43	98.51	98.38	98.46	98.27	98.40	98.44	98.49
**89**	98.31	98.35	98.35	98.47	98.41	98.47	98.31	98.41	98.41	98.48
**90**	98.38	98.43	98.44	98.54	98.42	98.43	98.32	98.42	98.43	98.49
**91**	98.36	98.38	98.36	98.50	98.37	98.39	98.23	98.40	98.38	98.43
**92**	98.40	98.44	98.42	98.57	98.43	98.45	98.33	98.45	98.44	98.48
**93**	98.38	98.32	98.30	98.51	98.35	98.31	98.30	98.35	98.31	98.41
**94**	98.29	98.29	98.22	98.49	98.22	98.29	98.24	98.32	98.34	98.42
**95**	98.45	98.43	98.48	98.55	98.41	98.50	98.41	98.48	98.55	98.60
**96**	98.54	98.49	98.48	98.59	98.46	98.45	98.46	98.55	98.44	98.62
**97**	98.42	98.48	98.47	98.62	98.40	98.42	98.39	98.48	98.48	98.55
**98**	98.58	98.60	98.63	98.61	98.56	98.56	98.52	98.58	98.63	98.69
**99**	98.55	98.46	98.49	98.62	98.45	98.49	98.46	98.55	98.52	98.64
**100**	98.43	98.45	98.47	98.64	98.41	98.45	98.39	98.50	98.47	98.56
**101**	98.42	98.44	98.43	98.62	98.45	98.40	98.36	98.52	98.49	98.54
**102**	98.42	98.45	98.43	98.57	98.49	98.44	98.40	98.52	98.53	98.52
**103**	98.45	98.40	98.43	98.52	98.38	98.45	98.40	98.46	98.44	98.53
**104**	98.34	98.35	98.33	98.49	98.29	98.41	98.27	98.33	98.34	98.47
**105**	98.40	98.37	98.39	98.47	98.40	98.43	98.43	98.44	98.50	98.55
**106**	98.51	98.44	98.44	98.50	98.48	98.50	98.51	98.52	98.51	98.58
**107**	98.31	98.25	98.34	98.41	98.30	98.35	98.27	98.32	98.35	98.45
**108**	98.33	98.27	98.27	98.42	98.23	98.29	98.26	98.30	98.29	98.44
**109**	98.50	98.45	98.45	98.52	98.36	98.46	98.38	98.45	98.50	98.51
**110**	98.36	98.26	98.30	98.38	98.31	98.38	98.25	98.35	98.26	98.50
**111**	98.41	98.34	98.37	98.46	98.30	98.37	98.33	98.41	98.36	98.52
**112**	98.39	98.31	98.38	98.40	98.32	98.41	98.32	98.46	98.29	98.51
**113**	98.36	98.29	98.35	98.41	98.35	98.31	98.29	98.33	98.28	98.49
**114**	98.37	98.28	98.32	98.41	98.30	98.32	98.30	98.39	98.31	98.47
**115**	98.46	98.32	98.32	98.42	98.28	98.40	98.34	98.40	98.31	98.51
**116**	98.45	98.34	98.39	98.54	98.40	98.41	98.34	98.49	98.46	98.52
**117**	98.24	98.24	98.20	98.37	98.26	98.34	98.12	98.26	98.33	98.34
**118**	98.28	98.25	98.29	98.40	98.29	98.33	98.23	98.25	98.31	98.46
**119**	96.97	96.91	97.01	97.00	96.89	96.98	96.87	96.96	96.94	97.04
**120**	96.97	96.89	96.94	97.01	96.96	97.01	96.93	97.00	96.99	97.02
**121**	96.29	96.08	96.11	96.28	96.10	96.12	96.15	96.16	96.01	96.36
**122**	96.88	96.87	96.85	96.97	96.89	96.94	96.84	96.91	96.88	96.95
**123**	97.10	97.14	97.14	97.25	97.09	97.22	97.09	97.04	97.14	97.14
**124**	97.10	97.16	97.20	97.26	97.16	97.16	97.10	97.12	97.20	97.14
**125**	97.18	97.17	97.17	97.27	97.10	97.19	97.14	97.27	97.15	97.22
**126**	97.23	97.18	97.21	97.30	97.23	97.28	97.17	97.25	97.28	97.26
**127**	97.24	97.18	97.21	97.27	97.16	97.20	97.13	97.23	97.18	97.29

**128**	96.74	96.79	96.83	96.95	96.76	96.89	96.72	96.80	96.87	96.81
**129**	97.05	96.95	96.99	97.08	97.00	96.99	96.93	97.01	96.94	97.01
**130**	96.98	96.93	96.91	97.01	96.94	96.95	96.98	96.94	96.94	97.02
**131**	96.89	96.86	96.91	97.00	96.84	96.94	96.75	96.83	96.86	96.94
**132**	96.92	96.93	96.94	97.08	96.88	96.89	96.84	96.92	96.90	96.93
**133**	97.07	97.09	97.07	97.13	97.07	97.04	97.02	97.07	97.09	97.08
**134**	96.87	96.86	96.88	97.03	96.91	96.98	96.84	96.90	96.92	96.99
**135**	96.91	96.88	96.91	97.01	96.89	96.96	96.81	96.91	96.91	96.96
**136**	96.77	96.77	96.83	96.95	96.76	96.93	96.77	96.82	96.86	96.92
**137**	96.40	96.42	96.43	96.52	96.34	96.47	96.35	96.44	96.49	96.41
**138**	96.47	96.45	96.44	96.56	96.49	96.46	96.42	96.48	96.47	96.53
**139**	96.49	96.51	96.53	96.57	96.49	96.56	96.47	96.50	96.55	96.54
**140**	96.48	96.49	96.46	96.56	96.45	96.50	96.53	96.49	96.53	96.57
**141**	96.19	96.21	96.22	96.26	96.17	96.26	96.18	96.21	96.22	96.29
**142**	96.51	96.47	96.53	96.60	96.47	96.57	96.51	96.53	96.61	96.54
**143**	96.63	96.55	96.57	96.67	96.46	96.54	96.50	96.60	96.63	96.66
**144**	95.45	95.42	95.41	95.49	95.33	95.43	95.40	95.46	95.40	95.50
**145**	95.35	95.26	95.25	95.46	95.28	95.27	95.31	95.26	95.22	95.33
**146**	95.69	95.58	95.64	95.79	95.58	95.60	95.59	95.67	95.72	95.71
**147**	96.58	96.63	96.62	96.53	96.62	96.52	96.59	96.51	96.61	96.51
**148**	91.91	91.94	91.91	91.98	91.84	91.93	91.83	91.88	91.92	91.91
**149**	91.94	91.95	91.91	92.00	91.84	91.99	91.80	91.93	92.00	91.97
**150**	91.90	91.91	91.94	91.96	91.93	91.96	91.90	91.90	91.95	91.90
**151**	91.94	91.89	91.90	91.90	91.82	91.89	91.86	91.90	91.92	91.96
**152**	91.94	91.89	91.90	91.90	91.82	91.89	91.86	91.90	91.92	91.96
**153**	91.86	91.83	91.81	91.86	91.85	91.90	91.87	91.80	91.86	91.84
**154**	91.83	91.89	91.80	91.84	91.70	91.79	91.72	91.82	91.80	91.85
**155**	91.78	91.78	91.76	91.82	91.76	91.81	91.75	91.73	91.75	91.76
**156**	91.87	91.83	91.81	91.87	91.79	91.83	91.83	91.82	91.77	91.89
**157**	91.92	91.85	91.77	91.86	91.75	91.79	91.83	91.81	91.86	91.85
**158**	92.10	92.09	92.10	92.16	92.12	92.14	92.07	92.10	92.21	92.16
**159**	93.98	94.04	94.04	94.16	94.08	94.11	94.04	94.09	93.95	94.11
**160**	93.54	93.52	93.64	93.66	93.59	93.58	93.59	93.59	93.61	93.65
**161**	93.48	93.60	93.57	93.57	93.51	93.56	93.52	93.55	93.57	93.59
**162**	83.76	83.73	83.70	83.73	83.75	83.77	83.80	83.78	83.75	83.86
**163**	83.82	83.77	83.68	83.78	83.73	83.78	83.79	83.77	83.79	83.82
**164**	83.91	83.90	83.82	83.88	83.95	83.92	83.85	83.81	83.79	83.90
**165**	83.92	83.89	83.91	83.98	84.05	83.88	83.89	83.94	83.91	83.88
**166**	83.99	83.89	83.94	83.97	83.98	83.93	83.96	83.93	83.86	83.89

Table S3. (continued)

**Isolate**	**41**	**42**	**43**	**44**	**45**	**46**	**47**	**48**	**49**	**50**
**1**	98.29	98.25	98.28	98.27	98.44	98.29	98.40	98.49	98.36	98.47
**2**	98.35	98.23	98.25	98.23	98.42	98.27	98.38	98.43	98.34	98.43
**3**	98.33	98.35	98.31	98.30	98.37	98.33	98.36	98.43	98.31	98.48
**4**	98.37	98.30	98.32	98.32	98.44	98.36	98.45	98.49	98.43	98.51
**5**	98.32	98.31	98.29	98.28	98.41	98.24	98.42	98.48	98.33	98.47
**6**	98.33	98.35	98.32	98.30	98.45	98.31	98.41	98.46	98.38	98.55
**7**	98.20	98.22	98.20	98.18	98.35	98.21	98.30	98.44	98.28	98.35
**8**	98.31	98.28	98.34	98.36	98.41	98.32	98.33	98.47	98.35	98.49
**9**	98.26	98.25	98.29	98.27	98.45	98.28	98.39	98.47	98.35	98.44
**10**	98.37	98.33	98.39	98.35	98.46	98.39	98.36	98.50	98.36	98.47
**11**	98.40	98.31	98.35	98.34	98.42	98.36	98.45	98.48	98.39	98.54
**12**	98.35	98.31	98.35	98.30	98.49	98.33	98.43	98.51	98.36	98.46
**13**	98.37	98.35	98.33	98.33	98.44	98.30	98.43	98.49	98.37	98.51
**14**	98.34	98.37	98.33	98.30	98.38	98.34	98.39	98.46	98.35	98.48
**15**	98.29	98.26	98.31	98.27	98.43	98.27	98.43	98.51	98.34	98.48
**16**	98.22	98.22	98.27	98.24	98.41	98.24	98.34	98.45	98.29	98.47
**17**	98.30	98.27	98.35	98.29	98.43	98.33	98.38	98.47	98.31	98.50
**18**	98.35	98.31	98.38	98.37	98.38	98.34	98.41	98.46	98.35	98.46
**19**	98.43	98.42	98.39	98.37	98.51	98.36	98.46	98.49	98.31	98.50
**20**	98.31	98.39	98.38	98.37	98.39	98.36	98.35	98.46	98.32	98.48
**21**	98.29	98.37	98.36	98.35	98.37	98.35	98.35	98.43	98.33	98.49
**22**	98.26	98.35	98.34	98.31	98.44	98.35	98.28	98.46	98.28	98.50
**23**	98.46	98.45	98.47	98.45	98.51	98.46	98.47	98.51	98.42	98.50
**24**	98.45	98.44	98.42	98.45	98.48	98.43	98.54	98.51	98.47	98.56
**25**	98.58	98.57	98.56	98.57	98.58	98.57	98.60	98.58	98.62	98.64
**26**	98.52	98.47	98.44	98.43	98.49	98.49	98.48	98.58	98.47	98.57
**27**	98.38	98.42	98.42	98.41	98.48	98.46	98.41	98.48	98.38	98.54
**28**	98.33	98.37	98.37	98.38	98.44	98.40	98.39	98.39	98.31	98.46
**29**	98.39	98.38	98.41	98.32	98.51	98.40	98.39	98.49	98.32	98.49
**30**	98.38	98.36	98.40	98.36	98.47	98.33	98.33	98.48	98.29	98.48
**31**	98.31	98.30	98.34	98.35	98.44	98.34	98.35	98.47	98.31	98.48
**32**	98.25	98.30	98.31	98.27	98.42	98.27	98.35	98.48	98.28	98.48
**33**	98.31	98.34	98.33	98.31	98.41	98.27	98.33	98.49	98.24	98.47
**34**	98.48	98.41	98.44	98.43	98.47	98.46	98.50	98.53	98.42	98.56
**35**	98.20	98.15	98.31	98.30	98.35	98.28	98.28	98.45	98.21	98.48
**36**	98.35	98.39	98.36	98.36	98.42	98.40	98.41	98.49	98.34	98.52
**37**	98.15	98.14	98.19	98.19	98.33	98.21	98.22	98.41	98.24	98.42
**38**	98.35	98.33	98.36	98.35	98.46	98.35	98.32	98.46	98.37	98.53
**39**	98.37	98.34	98.38	98.37	98.43	98.37	98.42	98.46	98.38	98.57
**40**	98.44	98.45	98.47	98.41	98.61	98.46	98.52	98.58	98.46	98.55
**41**	100.00	99.85	99.90	99.89	99.95	99.92	98.96	99.14	98.89	99.17

**42**	99.85	100.00	99.91	99.92	99.94	99.91	99.00	99.15	98.96	99.18
**43**	99.90	99.91	100.00	99.90	99.92	99.91	98.98	99.18	98.94	99.19
**44**	99.89	99.92	99.90	100.00	99.93	99.88	99.00	99.18	98.93	99.16
**45**	99.95	99.94	99.92	99.93	100.00	99.93	99.04	99.18	99.01	99.20
**46**	99.92	99.91	99.91	99.88	99.93	100.00	98.98	99.18	98.91	99.18
**47**	98.96	99.00	98.98	99.00	99.04	98.98	100.00	99.54	98.97	99.21
**48**	99.14	99.15	99.18	99.18	99.18	99.18	99.54	100.00	99.12	99.26
**49**	98.89	98.96	98.94	98.93	99.01	98.91	98.97	99.12	100.00	99.12
**50**	99.17	99.18	99.19	99.16	99.20	99.18	99.21	99.26	99.12	100.00
**51**	99.03	99.03	99.01	99.02	99.06	99.03	99.02	99.14	99.00	99.16
**52**	98.83	98.82	98.81	98.80	98.84	98.82	98.92	99.02	98.89	99.08
**53**	99.11	99.12	99.07	99.06	99.09	99.09	99.12	99.17	99.06	99.22
**54**	98.93	98.95	98.96	98.94	98.95	98.95	98.99	99.00	99.04	99.06
**55**	98.83	98.79	98.73	98.82	98.78	98.81	98.84	98.90	98.84	99.03
**56**	98.78	98.76	98.72	98.78	98.80	98.77	98.76	98.86	98.78	98.97
**57**	99.01	98.96	98.99	98.99	99.03	99.02	99.00	99.02	98.99	99.10
**58**	99.01	98.96	99.00	99.00	99.04	99.01	98.99	98.98	98.99	99.09
**59**	98.70	98.63	98.63	98.64	98.77	98.64	98.67	98.75	98.68	98.78
**60**	98.70	98.63	98.63	98.64	98.77	98.64	98.67	98.75	98.68	98.78
**61**	98.68	98.73	98.71	98.70	98.80	98.74	98.71	98.78	98.67	98.78
**62**	98.71	98.65	98.71	98.68	98.75	98.69	98.67	98.76	98.64	98.75
**63**	98.62	98.72	98.72	98.71	98.75	98.71	98.62	98.79	98.69	98.77
**64**	98.71	98.72	98.69	98.74	98.78	98.73	98.70	98.79	98.69	98.78
**65**	98.64	98.72	98.73	98.76	98.75	98.72	98.63	98.80	98.71	98.78
**66**	98.72	98.71	98.66	98.67	98.75	98.71	98.61	98.76	98.57	98.74
**67**	98.62	98.70	98.65	98.68	98.72	98.68	98.63	98.72	98.54	98.75
**68**	98.62	98.65	98.63	98.64	98.72	98.63	98.66	98.77	98.60	98.75
**69**	98.59	98.70	98.64	98.70	98.66	98.68	98.71	98.77	98.70	98.76
**70**	98.67	98.74	98.71	98.71	98.75	98.71	98.70	98.77	98.68	98.79
**71**	98.49	98.52	98.47	98.52	98.64	98.50	98.67	98.77	98.61	98.78
**72**	98.50	98.57	98.56	98.56	98.71	98.55	98.62	98.78	98.63	98.77
**73**	98.76	98.80	98.76	98.77	98.75	98.80	98.80	98.78	98.74	98.79
**74**	98.56	98.61	98.62	98.65	98.68	98.63	98.68	98.76	98.63	98.78
**75**	98.77	98.73	98.75	98.71	98.78	98.75	98.73	98.86	98.86	98.83
**76**	98.64	98.61	98.61	98.59	98.70	98.60	98.64	98.82	98.66	98.80
**77**	98.61	98.57	98.60	98.60	98.70	98.58	98.64	98.76	98.62	98.78
**78**	98.77	98.67	98.67	98.69	98.73	98.69	98.68	98.75	98.70	98.79
**79**	98.68	98.65	98.68	98.67	98.78	98.66	98.66	98.75	98.68	98.74
**80**	98.74	98.70	98.70	98.68	98.70	98.70	98.68	98.76	98.77	98.78
**81**	98.67	98.67	98.65	98.68	98.75	98.68	98.66	98.76	98.72	98.77
**82**	98.56	98.53	98.56	98.56	98.71	98.54	98.60	98.77	98.66	98.69
**83**	98.54	98.55	98.53	98.51	98.66	98.47	98.65	98.72	98.57	98.80
**84**	98.63	98.63	98.63	98.63	98.69	98.65	98.64	98.76	98.65	98.78

**85**	98.64	98.57	98.63	98.61	98.70	98.64	98.71	98.72	98.66	98.73
**86**	98.67	98.61	98.61	98.61	98.75	98.63	98.65	98.72	98.65	98.75
**87**	98.63	98.64	98.63	98.59	98.67	98.62	98.66	98.74	98.68	98.78
**88**	98.61	98.66	98.62	98.62	98.69	98.65	98.65	98.79	98.61	98.79
**89**	98.60	98.60	98.64	98.62	98.68	98.67	98.72	98.80	98.65	98.78
**90**	98.54	98.56	98.57	98.55	98.67	98.59	98.59	98.74	98.58	98.73
**91**	98.51	98.50	98.51	98.51	98.59	98.52	98.58	98.68	98.52	98.66
**92**	98.60	98.57	98.60	98.57	98.67	98.58	98.61	98.75	98.59	98.75
**93**	98.45	98.46	98.48	98.46	98.58	98.46	98.55	98.67	98.54	98.63
**94**	98.43	98.48	98.52	98.49	98.64	98.46	98.53	98.65	98.45	98.64
**95**	98.67	98.67	98.68	98.68	98.74	98.69	98.69	98.74	98.67	98.78
**96**	98.74	98.74	98.73	98.75	98.78	98.76	98.75	98.79	98.78	98.76
**97**	98.60	98.62	98.66	98.62	98.77	98.64	98.67	98.79	98.61	98.78
**98**	98.74	98.72	98.74	98.76	98.77	98.77	98.74	98.78	98.79	98.83
**99**	98.64	98.63	98.63	98.61	98.72	98.66	98.71	98.74	98.73	98.76
**100**	98.56	98.57	98.60	98.58	98.73	98.58	98.63	98.73	98.64	98.75
**101**	98.60	98.59	98.65	98.62	98.70	98.61	98.64	98.73	98.62	98.75
**102**	98.65	98.65	98.62	98.61	98.71	98.62	98.67	98.79	98.72	98.80
**103**	98.66	98.62	98.65	98.63	98.73	98.64	98.61	98.68	98.62	98.71
**104**	98.66	98.63	98.65	98.66	98.77	98.68	98.65	98.83	98.76	98.78
**105**	98.63	98.65	98.64	98.63	98.73	98.64	98.78	98.81	98.72	98.78
**106**	98.73	98.72	98.72	98.70	98.78	98.70	98.78	98.86	98.78	98.83
**107**	98.71	98.68	98.75	98.76	98.83	98.75	98.62	98.81	98.63	98.81
**108**	98.65	98.70	98.73	98.71	98.81	98.74	98.65	98.80	98.56	98.80
**109**	98.69	98.74	98.80	98.78	98.85	98.69	98.75	98.85	98.67	98.89
**110**	98.77	98.76	98.81	98.84	98.89	98.82	98.72	98.85	98.70	98.77
**111**	98.75	98.77	98.80	98.80	98.79	98.79	98.72	98.80	98.70	98.78
**112**	98.83	98.80	98.82	98.84	98.84	98.83	98.76	98.83	98.69	98.79
**113**	98.70	98.73	98.75	98.77	98.77	98.79	98.72	98.82	98.69	98.81
**114**	98.78	98.81	98.81	98.82	98.87	98.86	98.73	98.80	98.67	98.80
**115**	98.82	98.84	98.84	98.88	98.90	98.88	98.77	98.82	98.68	98.77
**116**	98.79	98.83	98.80	98.79	98.87	98.81	98.79	98.84	98.72	98.82
**117**	98.65	98.66	98.63	98.61	98.69	98.60	98.68	98.84	98.72	98.82
**118**	98.70	98.67	98.65	98.70	98.73	98.69	98.75	98.79	98.74	98.74
**119**	97.03	96.99	97.00	97.00	97.08	97.01	97.08	97.05	97.02	97.07
**120**	97.05	97.02	97.04	97.01	97.09	97.03	97.04	97.05	96.97	97.06
**121**	96.28	96.15	96.20	96.15	96.19	96.20	96.33	96.29	96.13	96.24
**122**	96.94	96.96	96.91	96.96	97.08	96.96	96.94	97.03	96.86	96.98
**123**	97.16	97.16	97.13	97.12	97.30	97.14	97.19	97.32	97.19	97.28
**124**	97.16	97.14	97.14	97.15	97.30	97.15	97.24	97.35	97.19	97.31
**125**	97.23	97.23	97.22	97.21	97.30	97.20	97.25	97.37	97.24	97.28
**126**	97.30	97.27	97.31	97.24	97.32	97.31	97.29	97.33	97.27	97.36
**127**	97.29	97.25	97.29	97.25	97.32	97.27	97.19	97.26	97.30	97.23

**128**	96.89	96.83	96.87	96.89	97.01	96.91	96.90	96.93	96.77	96.93
**129**	97.02	97.02	97.04	96.97	96.98	97.04	96.93	96.97	96.89	97.01
**130**	96.99	97.02	96.97	97.01	97.15	96.98	96.97	96.99	96.91	97.00
**131**	96.90	96.91	96.95	96.97	97.10	96.91	96.87	96.97	96.84	96.98
**132**	96.96	96.93	96.96	96.99	97.05	96.99	96.88	97.01	96.89	97.04
**133**	97.11	97.10	97.06	97.09	97.14	97.08	97.01	97.06	97.08	97.08
**134**	97.05	97.01	96.97	97.05	97.09	97.03	96.98	97.04	96.88	97.02
**135**	97.05	96.99	96.98	97.01	97.07	97.02	96.94	97.03	96.89	97.00
**136**	96.94	96.89	96.93	96.91	97.03	96.95	96.87	96.95	96.84	96.94
**137**	96.47	96.39	96.41	96.41	96.50	96.40	96.45	96.57	96.42	96.58
**138**	96.47	96.47	96.54	96.52	96.59	96.49	96.47	96.52	96.49	96.55
**139**	96.45	96.50	96.48	96.48	96.59	96.52	96.49	96.59	96.52	96.60
**140**	96.54	96.50	96.54	96.51	96.60	96.54	96.49	96.58	96.56	96.62
**141**	96.26	96.30	96.27	96.27	96.35	96.28	96.22	96.35	96.28	96.35
**142**	96.54	96.53	96.60	96.50	96.62	96.55	96.53	96.63	96.52	96.69
**143**	96.59	96.62	96.57	96.62	96.68	96.63	96.60	96.68	96.60	96.67
**144**	95.50	95.48	95.48	95.44	95.52	95.49	95.43	95.48	95.56	95.47
**145**	95.32	95.29	95.30	95.29	95.30	95.32	95.21	95.41	95.41	95.40
**146**	95.61	95.70	95.67	95.68	95.86	95.68	95.63	95.69	95.65	95.69
**147**	96.36	96.45	96.44	96.44	96.34	96.36	96.55	96.46	96.48	96.49
**148**	91.96	91.96	91.95	91.94	91.96	91.97	91.89	91.92	91.96	91.96
**149**	92.01	92.04	92.02	92.04	92.02	92.02	91.92	91.97	92.03	92.01
**150**	91.94	91.92	92.00	91.93	91.96	91.97	91.89	91.95	91.95	91.97
**151**	91.99	92.01	91.95	91.97	91.89	91.94	91.92	91.91	91.95	91.96
**152**	91.99	92.01	91.95	91.97	91.89	91.94	91.92	91.91	91.95	91.96
**153**	91.92	91.95	91.90	91.93	91.85	91.92	91.81	91.84	91.84	91.94
**154**	91.89	91.92	91.89	91.86	91.89	91.91	91.79	91.83	91.88	91.83
**155**	91.82	91.89	91.85	91.83	91.83	91.85	91.82	91.82	91.83	91.88
**156**	91.89	91.91	91.93	91.91	91.91	91.93	91.82	91.81	91.91	91.90
**157**	91.87	91.88	91.90	91.86	91.88	91.87	91.80	91.80	91.84	91.86
**158**	92.11	92.10	92.12	92.08	92.22	92.08	92.15	92.16	92.15	92.22
**159**	94.10	94.06	94.13	94.09	94.17	94.14	94.12	94.15	94.09	94.21
**160**	93.70	93.65	93.62	93.73	93.68	93.65	93.64	93.69	93.89	93.75
**161**	93.71	93.62	93.60	93.68	93.60	93.63	93.60	93.68	93.66	93.67
**162**	83.77	83.76	83.82	83.78	83.79	83.78	83.84	83.86	83.86	83.86
**163**	83.80	83.82	83.82	83.83	83.78	83.74	83.83	83.86	83.85	83.87
**164**	83.91	83.93	83.96	83.86	83.91	83.87	83.96	84.02	83.87	83.94
**165**	83.96	83.89	83.86	83.86	83.87	83.89	83.93	84.02	83.86	83.87
**166**	83.89	83.96	83.96	83.94	83.94	83.87	83.95	83.90	83.82	83.91

Table S3. (continued)

**Isolate**	**51**	**52**	**53**	**54**	**55**	**56**	**57**	**58**	**59**	**60**
**1**	98.43	98.27	98.53	98.48	98.31	98.25	98.49	98.49	98.43	98.43
**2**	98.39	98.30	98.51	98.50	98.33	98.21	98.48	98.49	98.45	98.45
**3**	98.39	98.28	98.50	98.51	98.40	98.30	98.51	98.51	98.45	98.45
**4**	98.47	98.38	98.53	98.51	98.39	98.32	98.50	98.50	98.50	98.50
**5**	98.44	98.30	98.51	98.42	98.36	98.26	98.50	98.51	98.43	98.43
**6**	98.44	98.36	98.52	98.51	98.42	98.35	98.56	98.55	98.45	98.45
**7**	98.32	98.20	98.44	98.36	98.27	98.16	98.45	98.44	98.32	98.32
**8**	98.45	98.23	98.49	98.49	98.29	98.26	98.44	98.44	98.48	98.48
**9**	98.40	98.13	98.54	98.53	98.29	98.29	98.53	98.54	98.45	98.45
**10**	98.36	98.35	98.55	98.54	98.34	98.28	98.48	98.50	98.37	98.37
**11**	98.47	98.29	98.51	98.45	98.33	98.27	98.47	98.46	98.50	98.50
**12**	98.44	98.23	98.52	98.46	98.28	98.27	98.45	98.48	98.43	98.43
**13**	98.44	98.39	98.53	98.52	98.37	98.32	98.57	98.58	98.46	98.46
**14**	98.42	98.32	98.52	98.45	98.37	98.27	98.51	98.53	98.42	98.42
**15**	98.47	98.25	98.52	98.48	98.27	98.27	98.50	98.48	98.41	98.41
**16**	98.39	98.26	98.46	98.44	98.20	98.26	98.50	98.48	98.39	98.39
**17**	98.42	98.11	98.53	98.48	98.21	98.26	98.47	98.49	98.47	98.47
**18**	98.39	98.29	98.54	98.52	98.31	98.30	98.54	98.53	98.49	98.49
**19**	98.47	98.42	98.55	98.56	98.41	98.26	98.60	98.59	98.41	98.41
**20**	98.31	98.32	98.54	98.50	98.31	98.30	98.46	98.45	98.34	98.34
**21**	98.31	98.35	98.54	98.48	98.34	98.34	98.46	98.47	98.35	98.35
**22**	98.31	98.29	98.49	98.42	98.29	98.31	98.44	98.45	98.33	98.33
**23**	98.46	98.38	98.56	98.59	98.46	98.40	98.54	98.55	98.54	98.54
**24**	98.55	98.40	98.62	98.64	98.53	98.48	98.63	98.63	98.56	98.56
**25**	98.61	98.54	98.67	98.65	98.58	98.60	98.61	98.61	98.64	98.64
**26**	98.49	98.49	98.56	98.56	98.48	98.41	98.61	98.62	98.53	98.53
**27**	98.42	98.33	98.50	98.56	98.37	98.42	98.54	98.55	98.42	98.42
**28**	98.40	98.31	98.53	98.52	98.38	98.37	98.49	98.49	98.40	98.40
**29**	98.37	98.22	98.53	98.47	98.23	98.21	98.46	98.48	98.43	98.43
**30**	98.40	98.27	98.53	98.49	98.32	98.26	98.52	98.51	98.35	98.35
**31**	98.39	98.27	98.49	98.49	98.31	98.16	98.42	98.45	98.36	98.36
**32**	98.38	98.23	98.51	98.38	98.25	98.20	98.47	98.46	98.36	98.36
**33**	98.39	98.31	98.54	98.43	98.29	98.28	98.52	98.52	98.35	98.35
**34**	98.48	98.37	98.56	98.51	98.43	98.42	98.47	98.49	98.53	98.53
**35**	98.32	98.17	98.54	98.39	98.28	98.29	98.42	98.43	98.37	98.37
**36**	98.40	98.34	98.56	98.51	98.39	98.30	98.53	98.52	98.48	98.48
**37**	98.25	98.25	98.45	98.51	98.25	98.19	98.42	98.43	98.31	98.31
**38**	98.38	98.35	98.55	98.55	98.35	98.33	98.46	98.46	98.45	98.45
**39**	98.44	98.21	98.53	98.47	98.31	98.30	98.49	98.50	98.44	98.44
**40**	98.48	98.45	98.61	98.59	98.49	98.38	98.60	98.60	98.47	98.47
**41**	99.03	98.83	99.11	98.93	98.83	98.78	99.01	99.01	98.70	98.70

**42**	99.03	98.82	99.12	98.95	98.79	98.76	98.96	98.96	98.63	98.63
**43**	99.01	98.81	99.07	98.96	98.73	98.72	98.99	99.00	98.63	98.63
**44**	99.02	98.80	99.06	98.94	98.82	98.78	98.99	99.00	98.64	98.64
**45**	99.06	98.84	99.09	98.95	98.78	98.80	99.03	99.04	98.77	98.77
**46**	99.03	98.82	99.09	98.95	98.81	98.77	99.02	99.01	98.64	98.64
**47**	99.02	98.92	99.12	98.99	98.84	98.76	99.00	98.99	98.67	98.67
**48**	99.14	99.02	99.17	99.00	98.90	98.86	99.02	98.98	98.75	98.75
**49**	99.00	98.89	99.06	99.04	98.84	98.78	98.99	98.99	98.68	98.68
**50**	99.16	99.08	99.22	99.06	99.03	98.97	99.10	99.09	98.78	98.78
**51**	100.00	99.06	99.16	98.99	98.84	98.80	98.98	98.99	98.77	98.77
**52**	99.06	100.00	98.99	98.94	98.69	98.63	98.91	98.87	98.69	98.69
**53**	99.16	98.99	100.00	99.07	99.06	99.02	99.11	99.12	98.87	98.87
**54**	98.99	98.94	99.07	100.00	99.90	99.84	99.48	99.48	98.95	98.95
**55**	98.84	98.69	99.06	99.90	100.00	99.90	99.40	99.40	98.80	98.80
**56**	98.80	98.63	99.02	99.84	99.90	100.00	99.36	99.37	98.71	98.71
**57**	98.98	98.91	99.11	99.48	99.40	99.36	100.00	99.99	98.84	98.84
**58**	98.99	98.87	99.12	99.48	99.40	99.37	99.99	100.00	98.85	98.85
**59**	98.77	98.69	98.87	98.95	98.80	98.71	98.84	98.85	100.00	100.00
**60**	98.77	98.69	98.87	98.95	98.80	98.71	98.84	98.85	100.00	100.00
**61**	98.70	98.48	98.85	98.90	98.68	98.70	98.83	98.84	99.82	99.82
**62**	98.75	98.60	98.79	98.92	98.79	98.76	98.86	98.84	99.81	99.81
**63**	98.74	98.60	98.86	98.96	98.78	98.73	98.85	98.83	99.79	99.79
**64**	98.68	98.62	98.86	98.91	98.78	98.68	98.83	98.80	99.82	99.82
**65**	98.75	98.56	98.88	98.93	98.71	98.66	98.84	98.82	99.80	99.80
**66**	98.68	98.44	98.80	98.82	98.65	98.63	98.82	98.82	99.69	99.69
**67**	98.69	98.58	98.84	98.83	98.70	98.63	98.80	98.79	99.71	99.71
**68**	98.65	98.70	98.81	98.88	98.75	98.70	98.86	98.84	99.78	99.78
**69**	98.67	98.51	98.82	98.89	98.72	98.77	98.80	98.79	99.67	99.67
**70**	98.72	98.62	98.81	98.85	98.77	98.70	98.84	98.84	99.76	99.76
**71**	98.63	98.42	98.78	98.80	98.68	98.62	98.85	98.82	99.74	99.74
**72**	98.65	98.33	98.80	98.87	98.58	98.72	98.82	98.80	99.66	99.66
**73**	98.79	98.65	98.86	98.91	98.87	98.87	98.86	98.86	99.84	99.84
**74**	98.62	98.51	98.85	98.90	98.63	98.68	98.80	98.80	99.61	99.61
**75**	98.88	98.73	98.86	98.99	98.87	98.80	98.88	98.88	99.18	99.18
**76**	98.73	98.68	98.83	98.93	98.83	98.70	98.89	98.86	99.03	99.03
**77**	98.70	98.54	98.82	98.88	98.79	98.67	98.81	98.78	99.01	99.01
**78**	98.76	98.64	98.79	98.94	98.77	98.74	98.82	98.84	99.09	99.09
**79**	98.73	98.60	98.84	98.99	98.77	98.78	98.85	98.86	99.04	99.04
**80**	98.80	98.65	98.71	98.75	98.64	98.63	98.70	98.71	98.74	98.74
**81**	98.76	98.63	98.87	98.93	98.73	98.69	98.86	98.87	99.08	99.08
**82**	98.73	98.70	98.77	98.79	98.63	98.64	98.72	98.71	98.59	98.59
**83**	98.65	98.51	98.74	98.80	98.67	98.61	98.82	98.82	99.00	99.00
**84**	98.72	98.57	98.80	98.84	98.71	98.65	98.81	98.80	99.03	99.03

**85**	98.74	98.56	98.87	98.92	98.70	98.67	98.78	98.79	98.99	98.99
**86**	98.70	98.62	98.84	98.90	98.77	98.67	98.84	98.85	99.07	99.07
**87**	98.71	98.60	98.85	98.84	98.68	98.65	98.83	98.82	99.03	99.03
**88**	98.76	98.59	98.82	98.91	98.85	98.77	98.88	98.87	99.08	99.08
**89**	98.72	98.63	98.91	98.93	98.84	98.75	98.84	98.83	98.99	98.99
**90**	98.62	98.60	98.76	98.82	98.73	98.68	98.77	98.81	98.79	98.79
**91**	98.58	98.48	98.71	98.77	98.67	98.61	98.72	98.71	98.75	98.75
**92**	98.64	98.58	98.78	98.85	98.78	98.67	98.79	98.81	98.82	98.82
**93**	98.61	98.51	98.74	98.76	98.66	98.57	98.77	98.78	98.77	98.77
**94**	98.60	98.47	98.77	98.74	98.59	98.50	98.76	98.73	98.77	98.77
**95**	98.79	98.64	98.85	98.93	98.75	98.64	98.81	98.79	98.96	98.96
**96**	98.75	98.66	98.90	98.91	98.83	98.75	98.85	98.84	99.00	99.00
**97**	98.74	98.61	98.86	98.94	98.78	98.72	98.87	98.87	98.88	98.88
**98**	98.80	98.70	98.88	98.90	98.77	98.74	98.81	98.81	98.96	98.96
**99**	98.76	98.62	98.85	98.83	98.77	98.63	98.81	98.81	98.87	98.87
**100**	98.69	98.63	98.84	98.82	98.73	98.61	98.82	98.82	98.85	98.85
**101**	98.63	98.58	98.86	98.87	98.73	98.61	98.84	98.82	98.81	98.81
**102**	98.74	98.71	98.84	98.84	98.72	98.64	98.86	98.87	98.94	98.94
**103**	98.69	98.56	98.82	98.82	98.75	98.64	98.80	98.80	98.92	98.92
**104**	98.71	98.61	98.89	98.97	98.77	98.74	98.80	98.80	98.78	98.78
**105**	98.77	98.68	98.86	98.84	98.81	98.68	98.87	98.87	98.92	98.92
**106**	98.82	98.74	98.88	98.90	98.84	98.73	98.88	98.87	98.98	98.98
**107**	98.73	98.59	98.84	98.81	98.56	98.58	98.68	98.68	98.59	98.59
**108**	98.71	98.59	98.82	98.74	98.57	98.55	98.66	98.70	98.61	98.61
**109**	98.80	98.72	98.87	98.81	98.60	98.55	98.78	98.79	98.64	98.64
**110**	98.78	98.48	98.82	98.76	98.50	98.60	98.62	98.67	98.61	98.61
**111**	98.79	98.64	98.83	98.79	98.59	98.63	98.71	98.74	98.67	98.67
**112**	98.79	98.60	98.80	98.80	98.66	98.64	98.66	98.67	98.66	98.66
**113**	98.80	98.58	98.76	98.77	98.60	98.64	98.65	98.71	98.63	98.63
**114**	98.74	98.54	98.81	98.72	98.52	98.62	98.73	98.70	98.64	98.64
**115**	98.80	98.53	98.78	98.79	98.58	98.68	98.69	98.71	98.64	98.64
**116**	98.84	98.68	98.87	98.82	98.60	98.58	98.79	98.80	98.67	98.67
**117**	98.77	98.57	98.79	98.76	98.60	98.62	98.74	98.78	98.56	98.56
**118**	98.76	98.75	98.79	98.78	98.64	98.61	98.74	98.74	98.52	98.52
**119**	97.03	96.81	97.06	97.09	97.02	97.01	97.07	97.05	97.03	97.03
**120**	97.00	96.96	97.08	97.03	96.92	96.98	97.09	97.08	96.96	96.96
**121**	96.16	95.92	96.30	96.12	95.99	95.82	96.10	96.34	96.41	96.41
**122**	96.89	96.84	97.09	97.03	96.88	96.91	97.05	97.03	96.94	96.94
**123**	97.24	97.15	97.35	97.23	97.18	97.15	97.34	97.33	97.21	97.21
**124**	97.25	97.13	97.39	97.27	97.19	97.19	97.40	97.37	97.22	97.22
**125**	97.26	97.19	97.34	97.30	97.18	97.14	97.37	97.35	97.18	97.18
**126**	97.25	97.21	97.38	97.33	97.26	97.25	97.41	97.37	97.28	97.28
**127**	97.22	97.16	97.30	97.27	97.19	97.16	97.31	97.26	97.16	97.16

**128**	96.87	96.56	97.09	96.97	96.78	96.77	96.97	96.95	96.83	96.83
**129**	96.99	96.86	97.07	97.05	96.96	96.95	97.09	97.04	96.92	96.92
**130**	97.02	96.76	97.06	96.99	96.79	96.90	97.09	97.06	96.98	96.98
**131**	96.91	96.66	97.07	97.06	96.75	96.82	97.03	97.00	96.83	96.83
**132**	96.91	96.76	97.04	97.02	96.85	96.89	97.01	97.00	96.96	96.96
**133**	97.09	96.94	97.10	97.07	96.97	96.98	97.11	97.07	97.05	97.05
**134**	97.00	96.79	97.10	97.03	96.91	96.90	97.03	97.02	96.91	96.91
**135**	96.98	96.83	97.11	97.04	96.87	96.86	97.03	97.00	96.89	96.89
**136**	96.93	96.79	97.05	96.96	96.80	96.77	96.95	96.95	96.83	96.83
**137**	96.51	96.42	96.62	96.56	96.43	96.35	96.58	96.56	96.49	96.49
**138**	96.45	96.34	96.69	96.67	96.45	96.49	96.62	96.60	96.44	96.44
**139**	96.52	96.47	96.68	96.66	96.52	96.50	96.67	96.68	96.52	96.52
**140**	96.53	96.45	96.66	96.60	96.53	96.58	96.64	96.58	96.61	96.61
**141**	96.26	96.23	96.42	96.41	96.28	96.29	96.41	96.40	96.22	96.22
**142**	96.59	96.57	96.68	96.59	96.55	96.52	96.68	96.63	96.63	96.63
**143**	96.54	96.42	96.76	96.77	96.53	96.52	96.78	96.76	96.57	96.57
**144**	95.46	95.31	95.57	95.61	95.52	95.51	95.68	95.63	95.51	95.51
**145**	95.32	95.28	95.47	95.45	95.42	95.41	95.55	95.51	95.40	95.40
**146**	95.71	95.39	95.79	95.72	95.59	95.60	95.75	95.74	95.66	95.66
**147**	96.55	96.43	96.62	96.58	96.35	96.33	96.40	96.60	96.45	96.45
**148**	91.98	91.84	91.97	91.96	91.94	91.95	91.95	91.98	91.97	91.97
**149**	92.00	91.84	92.01	91.96	91.90	91.96	92.01	92.00	92.01	92.01
**150**	91.89	91.89	92.03	92.02	92.03	92.04	92.03	91.96	91.94	91.94
**151**	91.91	91.81	91.89	91.96	91.87	91.92	92.00	91.91	91.95	91.95
**152**	91.91	91.81	91.89	91.96	91.87	91.92	92.00	91.91	91.95	91.95
**153**	92.00	91.84	91.92	91.86	91.90	91.89	91.92	91.92	91.98	91.98
**154**	91.87	91.76	91.88	91.85	91.81	91.91	91.88	91.81	91.86	91.86
**155**	91.82	91.71	91.85	91.88	91.78	91.88	91.88	91.84	91.86	91.86
**156**	91.89	91.79	91.95	91.91	91.84	91.93	91.96	91.89	91.92	91.92
**157**	91.95	91.75	91.88	91.82	91.78	91.81	91.87	91.86	91.87	91.87
**158**	92.17	92.05	92.22	92.23	92.10	92.07	92.30	92.23	92.09	92.09
**159**	94.07	93.95	94.28	94.22	94.02	94.08	94.27	94.21	93.98	93.98
**160**	93.69	93.51	93.82	93.81	93.65	93.74	93.86	93.81	93.65	93.65
**161**	93.53	93.50	93.80	93.77	93.65	93.71	93.74	93.76	93.56	93.56
**162**	83.75	83.78	83.87	83.80	83.75	83.91	83.90	83.80	83.78	83.78
**163**	83.80	83.80	83.84	83.84	83.80	83.89	83.93	83.83	83.81	83.81
**164**	83.87	83.87	84.02	83.88	83.91	83.89	83.97	83.95	83.90	83.90
**165**	83.89	83.96	83.94	83.95	83.92	83.96	84.05	83.95	83.91	83.91
**166**	83.91	83.88	84.01	83.92	83.89	84.00	83.98	83.94	83.91	83.91

Table S3. (continued)

Isolate	61	62	63	64	65	66	67	68	69	70
**1**	98.46	98.46	98.43	98.45	98.42	98.36	98.35	98.41	98.37	98.43
**2**	98.48	98.40	98.38	98.46	98.40	98.38	98.38	98.35	98.32	98.41
**3**	98.43	98.40	98.48	98.39	98.43	98.35	98.37	98.40	98.33	98.43
**4**	98.50	98.46	98.48	98.48	98.48	98.36	98.42	98.47	98.44	98.48
**5**	98.44	98.39	98.46	98.41	98.45	98.37	98.33	98.37	98.37	98.40
**6**	98.46	98.45	98.51	98.46	98.47	98.38	98.37	98.42	98.47	98.48
**7**	98.36	98.28	98.39	98.33	98.37	98.26	98.27	98.34	98.27	98.39
**8**	98.42	98.41	98.42	98.42	98.39	98.33	98.33	98.33	98.40	98.36
**9**	98.46	98.37	98.40	98.34	98.39	98.39	98.32	98.41	98.37	98.42
**10**	98.50	98.47	98.39	98.44	98.39	98.37	98.37	98.42	98.40	98.45
**11**	98.44	98.45	98.50	98.47	98.48	98.42	98.40	98.37	98.43	98.43
**12**	98.53	98.44	98.42	98.41	98.43	98.37	98.33	98.41	98.43	98.42
**13**	98.48	98.50	98.49	98.47	98.46	98.39	98.36	98.45	98.48	98.49
**14**	98.40	98.47	98.51	98.38	98.47	98.40	98.38	98.41	98.39	98.42
**15**	98.47	98.44	98.42	98.38	98.41	98.36	98.35	98.39	98.44	98.42
**16**	98.46	98.41	98.42	98.40	98.39	98.38	98.36	98.39	98.39	98.40
**17**	98.49	98.45	98.44	98.47	98.41	98.38	98.31	98.33	98.35	98.42
**18**	98.46	98.44	98.44	98.41	98.42	98.36	98.30	98.40	98.37	98.39
**19**	98.39	98.48	98.50	98.47	98.46	98.40	98.40	98.44	98.48	98.43
**20**	98.38	98.43	98.47	98.38	98.43	98.36	98.39	98.35	98.49	98.42
**21**	98.38	98.38	98.48	98.37	98.42	98.30	98.31	98.36	98.46	98.42
**22**	98.41	98.38	98.41	98.36	98.39	98.30	98.32	98.31	98.38	98.40
**23**	98.56	98.52	98.50	98.57	98.50	98.47	98.41	98.46	98.38	98.49
**24**	98.53	98.53	98.54	98.51	98.52	98.50	98.54	98.55	98.46	98.54
**25**	98.67	98.66	98.65	98.65	98.66	98.62	98.62	98.65	98.62	98.62
**26**	98.55	98.52	98.53	98.54	98.51	98.48	98.49	98.48	98.45	98.52
**27**	98.43	98.42	98.47	98.41	98.46	98.36	98.39	98.39	98.54	98.49
**28**	98.40	98.38	98.35	98.42	98.36	98.44	98.33	98.39	98.31	98.30
**29**	98.43	98.44	98.39	98.32	98.37	98.40	98.36	98.34	98.34	98.39
**30**	98.37	98.44	98.34	98.40	98.34	98.38	98.33	98.39	98.39	98.32
**31**	98.32	98.45	98.37	98.37	98.36	98.29	98.36	98.34	98.32	98.41
**32**	98.38	98.44	98.37	98.35	98.32	98.29	98.33	98.32	98.32	98.35
**33**	98.37	98.43	98.38	98.37	98.35	98.27	98.32	98.36	98.37	98.34
**34**	98.52	98.53	98.53	98.48	98.48	98.47	98.45	98.46	98.50	98.49
**35**	98.34	98.34	98.41	98.37	98.34	98.33	98.24	98.36	98.24	98.29
**36**	98.47	98.38	98.47	98.47	98.46	98.41	98.31	98.38	98.31	98.45
**37**	98.27	98.30	98.32	98.30	98.35	98.23	98.23	98.33	98.36	98.36
**38**	98.40	98.42	98.38	98.43	98.38	98.30	98.34	98.40	98.43	98.45
**39**	98.48	98.42	98.47	98.44	98.44	98.31	98.35	98.39	98.39	98.46
**40**	98.48	98.54	98.48	98.43	98.47	98.41	98.41	98.49	98.54	98.51
**41**	98.68	98.71	98.62	98.71	98.64	98.72	98.62	98.62	98.59	98.67

**42**	98.73	98.65	98.72	98.72	98.72	98.71	98.70	98.65	98.70	98.74
**43**	98.71	98.71	98.72	98.69	98.73	98.66	98.65	98.63	98.64	98.71
**44**	98.70	98.68	98.71	98.74	98.76	98.67	98.68	98.64	98.70	98.71
**45**	98.80	98.75	98.75	98.78	98.75	98.75	98.72	98.72	98.66	98.75
**46**	98.74	98.69	98.71	98.73	98.72	98.71	98.68	98.63	98.68	98.71
**47**	98.71	98.67	98.62	98.70	98.63	98.61	98.63	98.66	98.71	98.70
**48**	98.78	98.76	98.79	98.79	98.80	98.76	98.72	98.77	98.77	98.77
**49**	98.67	98.64	98.69	98.69	98.71	98.57	98.54	98.60	98.70	98.68
**50**	98.78	98.75	98.77	98.78	98.78	98.74	98.75	98.75	98.76	98.79
**51**	98.70	98.75	98.74	98.68	98.75	98.68	98.69	98.65	98.67	98.72
**52**	98.48	98.60	98.60	98.62	98.56	98.44	98.58	98.70	98.51	98.62
**53**	98.85	98.79	98.86	98.86	98.88	98.80	98.84	98.81	98.82	98.81
**54**	98.90	98.92	98.96	98.91	98.93	98.82	98.83	98.88	98.89	98.85
**55**	98.68	98.79	98.78	98.78	98.71	98.65	98.70	98.75	98.72	98.77
**56**	98.70	98.76	98.73	98.68	98.66	98.63	98.63	98.70	98.77	98.70
**57**	98.83	98.86	98.85	98.83	98.84	98.82	98.80	98.86	98.80	98.84
**58**	98.84	98.84	98.83	98.80	98.82	98.82	98.79	98.84	98.79	98.84
**59**	99.82	99.81	99.79	99.82	99.80	99.69	99.71	99.78	99.67	99.76
**60**	99.82	99.81	99.79	99.82	99.80	99.69	99.71	99.78	99.67	99.76
**61**	100.00	99.76	99.70	99.83	99.65	99.74	99.73	99.69	99.63	99.72
**62**	99.76	100.00	99.73	99.82	99.72	99.75	99.78	99.77	99.75	99.82
**63**	99.70	99.73	100.00	99.75	99.99	99.65	99.73	99.75	99.74	99.81
**64**	99.83	99.82	99.75	100.00	99.73	99.75	99.74	99.73	99.69	99.80
**65**	99.65	99.72	99.99	99.73	100.00	99.63	99.72	99.70	99.75	99.79
**66**	99.74	99.75	99.65	99.75	99.63	100.00	99.68	99.70	99.70	99.72
**67**	99.73	99.78	99.73	99.74	99.72	99.68	100.00	99.61	99.63	99.70
**68**	99.69	99.77	99.75	99.73	99.70	99.70	99.61	100.00	99.72	99.75
**69**	99.63	99.75	99.74	99.69	99.75	99.70	99.63	99.72	100.00	99.80
**70**	99.72	99.82	99.81	99.80	99.79	99.72	99.70	99.75	99.80	100.00
**71**	99.60	99.69	99.62	99.64	99.59	99.54	99.51	99.69	99.74	99.77
**72**	99.59	99.68	99.72	99.62	99.69	99.58	99.53	99.64	99.80	99.76
**73**	99.80	99.80	99.86	99.84	99.88	99.79	99.77	99.84	99.87	99.89
**74**	99.65	99.77	99.67	99.71	99.67	99.62	99.62	99.74	99.81	99.86
**75**	99.13	99.07	99.17	99.18	99.16	99.13	99.11	99.13	99.12	99.16
**76**	99.04	99.03	99.05	99.06	99.03	98.97	98.99	99.09	99.01	99.08
**77**	98.99	98.99	98.98	99.00	98.98	98.90	98.95	99.08	98.97	99.05
**78**	99.12	99.06	99.12	99.12	99.09	99.03	99.02	99.08	99.07	99.09
**79**	99.11	99.07	99.11	99.09	99.03	99.06	98.95	99.02	98.98	99.06
**80**	98.78	98.74	98.76	98.73	98.76	98.69	98.70	98.69	98.71	98.73
**81**	99.04	99.04	98.98	99.01	98.97	98.99	98.95	98.99	99.01	99.08
**82**	98.64	98.66	98.66	98.68	98.67	98.57	98.57	98.67	98.65	98.71
**83**	98.93	99.01	98.98	98.97	98.91	98.86	98.88	98.98	98.92	98.96
**84**	99.05	99.12	99.09	99.02	99.05	98.94	99.00	99.02	99.08	99.11

**85**	99.00	99.01	99.02	98.93	99.00	98.94	98.94	99.08	98.99	99.01
**86**	99.02	99.03	99.05	99.06	99.04	99.02	99.04	98.99	98.98	99.06
**87**	98.96	99.03	99.01	99.03	99.04	98.90	99.02	99.01	99.00	99.06
**88**	99.09	99.04	99.17	99.11	99.12	98.95	99.04	99.09	99.08	99.12
**89**	99.09	99.09	99.14	99.11	99.12	98.98	99.05	99.11	99.13	99.15
**90**	98.85	98.79	98.81	98.87	98.81	98.77	98.82	98.83	98.79	98.82
**91**	98.82	98.76	98.75	98.83	98.76	98.76	98.75	98.74	98.73	98.75
**92**	98.88	98.83	98.81	98.89	98.81	98.80	98.81	98.82	98.82	98.84
**93**	98.81	98.84	98.76	98.78	98.80	98.81	98.73	98.81	98.75	98.80
**94**	98.72	98.83	98.78	98.77	98.73	98.74	98.72	98.77	98.71	98.72
**95**	99.00	98.98	98.93	99.02	98.97	98.90	98.90	98.86	98.90	98.91
**96**	98.99	98.96	98.98	99.01	99.00	98.93	98.95	98.86	98.86	98.99
**97**	98.90	98.94	98.87	98.91	98.86	98.83	98.83	98.91	98.84	98.91
**98**	98.98	98.97	98.98	98.97	98.98	98.93	98.96	98.93	98.92	98.96
**99**	98.93	98.88	98.88	98.93	98.88	98.91	98.86	98.79	98.77	98.87
**100**	98.88	98.86	98.82	98.90	98.81	98.85	98.77	98.79	98.79	98.85
**101**	98.83	98.86	98.79	98.83	98.77	98.79	98.80	98.80	98.84	98.78
**102**	98.92	98.97	98.87	98.88	98.87	98.87	98.87	98.89	98.92	98.93
**103**	98.92	98.89	98.90	98.93	98.90	98.92	98.89	98.89	98.84	98.90
**104**	98.77	98.76	98.77	98.71	98.75	98.69	98.68	98.76	98.71	98.74
**105**	98.84	98.84	98.87	98.82	98.91	98.82	98.82	98.89	98.83	98.86
**106**	98.93	98.94	98.97	98.92	98.99	98.93	98.91	98.93	98.84	98.95
**107**	98.55	98.57	98.55	98.56	98.57	98.55	98.45	98.67	98.50	98.62
**108**	98.54	98.58	98.52	98.57	98.54	98.63	98.49	98.62	98.58	98.62
**109**	98.62	98.66	98.63	98.66	98.62	98.66	98.63	98.63	98.71	98.68
**110**	98.62	98.56	98.65	98.63	98.63	98.63	98.57	98.67	98.56	98.56
**111**	98.60	98.60	98.63	98.65	98.61	98.63	98.55	98.66	98.56	98.62
**112**	98.58	98.59	98.60	98.62	98.61	98.65	98.63	98.69	98.51	98.64
**113**	98.64	98.54	98.69	98.61	98.65	98.66	98.59	98.65	98.58	98.64
**114**	98.59	98.57	98.60	98.60	98.58	98.68	98.56	98.62	98.55	98.55
**115**	98.65	98.60	98.68	98.62	98.65	98.69	98.62	98.68	98.55	98.61
**116**	98.62	98.70	98.64	98.62	98.64	98.63	98.56	98.63	98.67	98.66
**117**	98.51	98.62	98.58	98.56	98.58	98.37	98.51	98.56	98.52	98.59
**118**	98.48	98.57	98.48	98.49	98.51	98.51	98.55	98.56	98.55	98.57
**119**	97.03	97.00	97.04	97.06	97.04	96.97	97.03	97.06	97.00	97.06
**120**	97.06	97.03	97.02	97.00	97.02	97.03	97.04	97.02	97.00	97.01
**121**	96.28	96.22	96.26	96.29	96.21	96.38	96.28	96.20	96.32	96.36
**122**	96.91	96.94	96.93	96.91	96.90	96.87	96.85	96.97	96.81	96.92
**123**	97.18	97.15	97.22	97.18	97.23	97.17	97.20	97.19	97.24	97.21
**124**	97.17	97.14	97.22	97.16	97.20	97.16	97.17	97.18	97.21	97.23
**125**	97.21	97.25	97.23	97.25	97.22	97.22	97.13	97.16	97.15	97.20
**126**	97.23	97.24	97.28	97.26	97.27	97.25	97.24	97.25	97.21	97.28
**127**	97.23	97.23	97.23	97.22	97.24	97.21	97.19	97.19	97.18	97.22

**128**	96.79	96.83	96.79	96.79	96.75	96.71	96.73	96.84	96.80	96.88
**129**	96.86	96.99	96.97	96.94	96.92	96.92	96.89	96.99	96.86	96.91
**130**	97.01	97.03	96.96	97.02	96.93	97.05	96.96	96.94	96.89	96.89
**131**	96.95	96.88	96.88	96.86	96.87	96.93	96.75	96.93	96.87	96.86
**132**	96.92	96.95	96.93	96.94	96.88	96.88	96.83	96.96	96.90	96.91
**133**	97.06	97.00	97.12	97.03	97.08	97.00	97.01	97.04	97.00	97.02
**134**	96.88	96.91	96.94	96.97	96.87	96.93	96.84	96.96	96.89	96.90
**135**	96.90	96.94	96.90	96.93	96.84	96.92	96.84	96.94	96.89	96.91
**136**	96.80	96.83	96.85	96.82	96.78	96.85	96.83	96.86	96.86	96.88
**137**	96.41	96.44	96.48	96.50	96.46	96.44	96.47	96.40	96.44	96.36
**138**	96.37	96.47	96.50	96.44	96.45	96.43	96.35	96.51	96.41	96.41
**139**	96.51	96.49	96.50	96.50	96.45	96.48	96.49	96.51	96.53	96.57
**140**	96.50	96.48	96.58	96.56	96.51	96.52	96.47	96.60	96.53	96.54
**141**	96.25	96.29	96.22	96.23	96.22	96.21	96.21	96.24	96.28	96.24
**142**	96.57	96.57	96.64	96.63	96.56	96.56	96.55	96.55	96.60	96.58
**143**	96.50	96.54	96.57	96.58	96.53	96.43	96.52	96.55	96.55	96.59
**144**	95.43	95.40	95.49	95.47	95.48	95.40	95.36	95.47	95.38	95.44
**145**	95.33	95.30	95.30	95.42	95.32	95.38	95.33	95.28	95.34	95.33
**146**	95.73	95.70	95.68	95.70	95.67	95.75	95.56	95.62	95.68	95.64
**147**	96.45	96.44	96.48	96.50	96.45	96.46	96.44	96.54	96.39	96.49
**148**	91.91	91.85	91.98	91.97	91.97	91.91	91.87	91.85	91.93	91.95
**149**	91.91	91.98	92.03	91.92	92.04	91.94	91.90	91.95	91.93	91.97
**150**	91.94	91.91	91.97	91.94	91.94	91.94	91.81	91.91	91.89	91.97
**151**	91.93	91.83	91.97	91.92	91.95	91.88	91.90	91.87	91.83	91.92
**152**	91.93	91.83	91.97	91.92	91.95	91.88	91.90	91.87	91.83	91.92
**153**	91.90	91.87	91.92	91.97	91.96	91.95	91.93	91.92	91.92	91.89
**154**	91.80	91.79	91.85	91.84	91.85	91.82	91.75	91.81	91.80	91.77
**155**	91.85	91.83	91.84	91.77	91.80	91.83	91.79	91.88	91.79	91.87
**156**	91.88	91.87	91.90	91.89	91.88	91.95	91.88	91.94	91.87	91.93
**157**	91.82	91.90	91.94	91.93	91.93	91.89	91.93	91.83	91.89	91.81
**158**	92.17	92.18	92.09	92.11	92.09	92.11	92.08	92.12	92.12	92.13
**159**	94.08	94.12	94.13	94.05	94.11	94.10	93.99	94.18	94.03	94.14
**160**	93.66	93.61	93.68	93.54	93.60	93.66	93.59	93.66	93.55	93.56
**161**	93.63	93.61	93.62	93.59	93.62	93.58	93.60	93.58	93.58	93.60
**162**	83.77	83.81	83.74	83.79	83.77	83.83	83.81	83.78	83.79	83.80
**163**	83.77	83.81	83.75	83.79	83.80	83.84	83.83	83.85	83.76	83.80
**164**	83.95	83.93	83.87	83.90	83.92	83.91	83.91	84.00	83.99	83.96
**165**	83.93	83.90	83.88	83.89	83.93	83.91	83.92	83.91	83.95	83.95
**166**	83.90	83.91	83.86	83.86	83.83	83.96	83.91	83.96	83.85	83.98

Table S3. (continued)

**Isolate**	**71**	**72**	**73**	**74**	**75**	**76**	**77**	**78**	**79**	**80**
**1**	98.33	98.28	98.49	98.28	98.52	98.39	98.30	98.39	98.44	98.39
**2**	98.32	98.33	98.45	98.23	98.46	98.36	98.30	98.43	98.40	98.38
**3**	98.24	98.26	98.48	98.33	98.44	98.46	98.37	98.45	98.43	98.33
**4**	98.32	98.35	98.53	98.34	98.52	98.44	98.38	98.40	98.45	98.42
**5**	98.21	98.24	98.49	98.33	98.49	98.38	98.32	98.38	98.36	98.41
**6**	98.39	98.37	98.53	98.42	98.54	98.33	98.40	98.51	98.45	98.38
**7**	98.16	98.18	98.41	98.24	98.45	98.29	98.26	98.32	98.32	98.33
**8**	98.24	98.27	98.44	98.29	98.49	98.35	98.34	98.44	98.38	98.33
**9**	98.27	98.32	98.46	98.40	98.46	98.35	98.29	98.45	98.46	98.40
**10**	98.35	98.33	98.47	98.31	98.52	98.36	98.29	98.46	98.36	98.45
**11**	98.27	98.37	98.49	98.36	98.49	98.38	98.36	98.37	98.43	98.38
**12**	98.31	98.37	98.47	98.37	98.52	98.40	98.30	98.39	98.45	98.39
**13**	98.35	98.35	98.53	98.41	98.54	98.39	98.37	98.47	98.43	98.42
**14**	98.25	98.34	98.49	98.33	98.53	98.40	98.40	98.45	98.43	98.38
**15**	98.30	98.43	98.44	98.36	98.50	98.37	98.31	98.41	98.44	98.38
**16**	98.28	98.29	98.46	98.34	98.47	98.33	98.26	98.38	98.43	98.34
**17**	98.22	98.27	98.48	98.26	98.49	98.35	98.27	98.41	98.44	98.38
**18**	98.35	98.22	98.50	98.29	98.51	98.41	98.31	98.40	98.35	98.38
**19**	98.39	98.43	98.52	98.41	98.56	98.46	98.39	98.48	98.43	98.43
**20**	98.27	98.38	98.49	98.44	98.44	98.36	98.35	98.40	98.36	98.43
**21**	98.32	98.30	98.51	98.40	98.48	98.39	98.34	98.41	98.37	98.45
**22**	98.21	98.21	98.47	98.32	98.50	98.35	98.27	98.38	98.36	98.37
**23**	98.42	98.34	98.49	98.45	98.53	98.44	98.39	98.52	98.45	98.46
**24**	98.45	98.47	98.54	98.51	98.56	98.54	98.55	98.50	98.54	98.44
**25**	98.66	98.63	98.66	98.65	98.68	98.65	98.64	98.65	98.63	98.53
**26**	98.46	98.42	98.55	98.43	98.55	98.51	98.49	98.52	98.53	98.46
**27**	98.39	98.37	98.55	98.42	98.47	98.37	98.35	98.54	98.46	98.42
**28**	98.34	98.30	98.44	98.30	98.49	98.44	98.41	98.45	98.41	98.38
**29**	98.33	98.38	98.48	98.25	98.53	98.35	98.31	98.51	98.44	98.44
**30**	98.32	98.35	98.45	98.31	98.50	98.36	98.36	98.54	98.38	98.48
**31**	98.20	98.31	98.47	98.25	98.55	98.28	98.27	98.45	98.42	98.43
**32**	98.23	98.21	98.40	98.25	98.48	98.34	98.28	98.33	98.37	98.42
**33**	98.29	98.27	98.43	98.34	98.48	98.37	98.32	98.37	98.37	98.42
**34**	98.42	98.42	98.55	98.42	98.59	98.48	98.45	98.51	98.53	98.47
**35**	98.25	98.19	98.44	98.16	98.42	98.40	98.39	98.33	98.37	98.34
**36**	98.26	98.27	98.47	98.35	98.47	98.46	98.43	98.46	98.43	98.41
**37**	98.20	98.26	98.44	98.27	98.43	98.26	98.17	98.37	98.33	98.31
**38**	98.32	98.35	98.50	98.35	98.51	98.35	98.35	98.49	98.45	98.47
**39**	98.35	98.38	98.44	98.38	98.46	98.35	98.31	98.48	98.43	98.44
**40**	98.41	98.42	98.57	98.46	98.59	98.48	98.45	98.57	98.43	98.52
**41**	98.49	98.50	98.76	98.56	98.77	98.64	98.61	98.77	98.68	98.74

**42**	98.52	98.57	98.80	98.61	98.73	98.61	98.57	98.67	98.65	98.70
**43**	98.47	98.56	98.76	98.62	98.75	98.61	98.60	98.67	98.68	98.70
**44**	98.52	98.56	98.77	98.65	98.71	98.59	98.60	98.69	98.67	98.68
**45**	98.64	98.71	98.75	98.68	98.78	98.70	98.70	98.73	98.78	98.70
**46**	98.50	98.55	98.80	98.63	98.75	98.60	98.58	98.69	98.66	98.70
**47**	98.67	98.62	98.80	98.68	98.73	98.64	98.64	98.68	98.66	98.68
**48**	98.77	98.78	98.78	98.76	98.86	98.82	98.76	98.75	98.75	98.76
**49**	98.61	98.63	98.74	98.63	98.86	98.66	98.62	98.70	98.68	98.77
**50**	98.78	98.77	98.79	98.78	98.83	98.80	98.78	98.79	98.74	98.78
**51**	98.63	98.65	98.79	98.62	98.88	98.73	98.70	98.76	98.73	98.80
**52**	98.42	98.33	98.65	98.51	98.73	98.68	98.54	98.64	98.60	98.65
**53**	98.78	98.80	98.86	98.85	98.86	98.83	98.82	98.79	98.84	98.71
**54**	98.80	98.87	98.91	98.90	98.99	98.93	98.88	98.94	98.99	98.75
**55**	98.68	98.58	98.87	98.63	98.87	98.83	98.79	98.77	98.77	98.64
**56**	98.62	98.72	98.87	98.68	98.80	98.70	98.67	98.74	98.78	98.63
**57**	98.85	98.82	98.86	98.80	98.88	98.89	98.81	98.82	98.85	98.70
**58**	98.82	98.80	98.86	98.80	98.88	98.86	98.78	98.84	98.86	98.71
**59**	99.74	99.66	99.84	99.61	99.18	99.03	99.01	99.09	99.04	98.74
**60**	99.74	99.66	99.84	99.61	99.18	99.03	99.01	99.09	99.04	98.74
**61**	99.60	99.59	99.80	99.65	99.13	99.04	98.99	99.12	99.11	98.78
**62**	99.69	99.68	99.80	99.77	99.07	99.03	98.99	99.06	99.07	98.74
**63**	99.62	99.72	99.86	99.67	99.17	99.05	98.98	99.12	99.11	98.76
**64**	99.64	99.62	99.84	99.71	99.18	99.06	99.00	99.12	99.09	98.73
**65**	99.59	99.69	99.88	99.67	99.16	99.03	98.98	99.09	99.03	98.76
**66**	99.54	99.58	99.79	99.62	99.13	98.97	98.90	99.03	99.06	98.69
**67**	99.51	99.53	99.77	99.62	99.11	98.99	98.95	99.02	98.95	98.70
**68**	99.69	99.64	99.84	99.74	99.13	99.09	99.08	99.08	99.02	98.69
**69**	99.74	99.80	99.87	99.81	99.12	99.01	98.97	99.07	98.98	98.71
**70**	99.77	99.76	99.89	99.86	99.16	99.08	99.05	99.09	99.06	98.73
**71**	100.00	99.65	99.83	99.73	99.03	98.94	98.85	99.03	99.00	98.60
**72**	99.65	100.00	99.89	99.76	99.02	99.00	98.87	99.04	98.96	98.67
**73**	99.83	99.89	100.00	99.94	99.19	99.09	99.12	99.14	99.12	98.77
**74**	99.73	99.76	99.94	100.00	99.12	98.97	98.93	99.04	98.98	98.70
**75**	99.03	99.02	99.19	99.12	100.00	99.74	99.83	99.08	99.01	98.74
**76**	98.94	99.00	99.09	98.97	99.74	100.00	99.71	98.96	98.98	98.66
**77**	98.85	98.87	99.12	98.93	99.83	99.71	100.00	98.97	98.95	98.63
**78**	99.03	99.04	99.14	99.04	99.08	98.96	98.97	100.00	99.79	98.72
**79**	99.00	98.96	99.12	98.98	99.01	98.98	98.95	99.79	100.00	98.69
**80**	98.60	98.67	98.77	98.70	98.74	98.66	98.63	98.72	98.69	100.00
**81**	98.98	98.95	99.08	99.02	99.19	98.96	98.97	99.06	98.95	98.80
**82**	98.63	98.60	98.70	98.61	98.79	98.65	98.58	98.66	98.58	98.90
**83**	98.88	98.84	99.09	98.82	99.05	98.95	98.93	98.92	98.95	98.59
**84**	99.01	98.96	99.13	99.03	99.10	98.94	98.89	98.95	98.93	98.68

**85**	98.99	99.00	99.09	98.92	99.05	99.04	98.89	98.91	98.93	98.68
**86**	98.95	98.93	99.14	99.00	99.10	98.93	98.95	99.01	98.89	98.73
**87**	98.92	98.92	99.12	99.02	99.16	99.07	99.03	98.97	98.95	98.70
**88**	98.91	99.00	99.17	99.08	98.98	99.00	98.94	98.94	99.03	98.67
**89**	99.02	99.08	99.23	99.06	99.01	98.99	98.96	98.97	99.03	98.68
**90**	98.75	98.73	98.85	98.79	98.89	98.80	98.80	98.84	98.75	98.71
**91**	98.64	98.67	98.85	98.73	98.85	98.75	98.74	98.80	98.71	98.65
**92**	98.76	98.73	98.89	98.85	98.92	98.79	98.83	98.85	98.80	98.71
**93**	98.70	98.63	98.81	98.68	98.92	98.75	98.76	98.80	98.75	98.63
**94**	98.61	98.61	98.85	98.67	98.95	98.75	98.71	98.82	98.69	98.61
**95**	98.82	98.91	98.95	98.86	99.02	98.93	98.88	98.94	98.86	98.75
**96**	98.88	98.85	98.97	98.93	99.01	98.97	98.95	98.98	98.95	98.77
**97**	98.76	98.79	98.95	98.91	99.00	98.88	98.87	98.91	98.84	98.78
**98**	98.89	98.91	98.99	98.88	99.06	98.95	98.98	99.00	98.91	98.80
**99**	98.73	98.72	98.91	98.79	98.98	98.86	98.85	98.88	98.79	98.74
**100**	98.68	98.71	98.87	98.85	99.01	98.83	98.84	98.90	98.78	98.78
**101**	98.74	98.71	98.87	98.77	98.98	98.84	98.83	98.84	98.85	98.75
**102**	98.89	98.89	98.92	98.91	99.10	98.85	98.86	98.93	98.91	98.74
**103**	98.79	98.82	98.94	98.80	99.01	98.88	98.88	98.90	98.84	98.72
**104**	98.69	98.58	98.85	98.68	98.94	98.78	98.74	98.82	98.72	98.72
**105**	98.75	98.76	98.96	98.85	98.99	98.94	98.92	98.91	98.89	98.72
**106**	98.78	98.83	98.96	98.90	99.01	99.00	98.97	98.89	98.92	98.75
**107**	98.49	98.50	98.69	98.48	98.67	98.58	98.54	98.62	98.58	98.56
**108**	98.44	98.46	98.68	98.48	98.66	98.57	98.55	98.62	98.56	98.56
**109**	98.58	98.63	98.71	98.57	98.78	98.63	98.60	98.71	98.56	98.68
**110**	98.47	98.54	98.70	98.54	98.70	98.64	98.58	98.63	98.66	98.58
**111**	98.48	98.52	98.65	98.56	98.65	98.66	98.61	98.67	98.62	98.62
**112**	98.55	98.54	98.68	98.55	98.70	98.65	98.63	98.67	98.67	98.59
**113**	98.49	98.50	98.71	98.55	98.65	98.62	98.58	98.60	98.65	98.51
**114**	98.56	98.54	98.67	98.56	98.69	98.63	98.63	98.63	98.66	98.59
**115**	98.49	98.61	98.72	98.62	98.69	98.67	98.67	98.69	98.69	98.60
**116**	98.51	98.64	98.69	98.65	98.73	98.63	98.58	98.66	98.61	98.64
**117**	98.49	98.41	98.69	98.51	98.65	98.52	98.52	98.59	98.50	98.63
**118**	98.56	98.51	98.61	98.59	98.63	98.59	98.60	98.57	98.52	98.59
**119**	97.00	97.00	97.11	97.01	97.04	97.00	96.92	96.98	97.08	97.07
**120**	96.98	96.95	97.03	97.00	97.03	96.92	96.97	97.02	96.96	97.13
**121**	96.05	96.18	96.22	96.21	96.05	96.02	95.91	96.26	96.27	96.22
**122**	96.91	96.79	96.98	96.78	97.04	96.84	96.85	97.01	96.93	97.09
**123**	97.16	97.16	97.23	97.14	97.23	97.15	97.17	97.13	97.13	97.26
**124**	97.16	97.16	97.26	97.21	97.23	97.17	97.17	97.15	97.16	97.26
**125**	97.22	97.19	97.20	97.17	97.26	97.20	97.18	97.23	97.22	97.35
**126**	97.27	97.20	97.27	97.26	97.34	97.27	97.25	97.34	97.26	97.30
**127**	97.16	97.18	97.27	97.16	97.26	97.19	97.14	97.29	97.16	97.28

**128**	96.71	96.65	96.90	96.71	96.90	96.80	96.72	96.90	96.87	96.99
**129**	96.98	96.84	96.94	96.91	97.03	96.91	96.96	97.02	96.95	97.05
**130**	96.96	97.00	96.94	96.89	97.04	96.98	96.96	97.01	97.04	97.03
**131**	96.78	96.87	96.94	96.82	96.92	96.88	96.84	96.96	96.95	96.99
**132**	96.88	96.81	96.94	96.83	97.05	96.93	96.82	97.00	96.95	97.03
**133**	97.00	97.02	97.08	96.99	97.08	96.99	96.97	97.10	96.99	97.08
**134**	96.86	96.86	96.98	96.84	97.00	96.92	96.89	97.03	96.86	97.07
**135**	96.91	96.83	96.96	96.80	97.02	96.90	96.88	97.00	96.86	97.06
**136**	96.82	96.74	96.89	96.77	96.95	96.79	96.78	96.92	96.81	97.00
**137**	96.33	96.38	96.42	96.35	96.47	96.44	96.38	96.51	96.49	96.58
**138**	96.40	96.36	96.46	96.37	96.61	96.46	96.47	96.65	96.44	96.60
**139**	96.47	96.47	96.58	96.45	96.56	96.46	96.43	96.57	96.55	96.64
**140**	96.46	96.46	96.59	96.54	96.57	96.53	96.56	96.56	96.57	96.63
**141**	96.21	96.20	96.31	96.20	96.30	96.29	96.23	96.38	96.23	96.32
**142**	96.51	96.51	96.62	96.50	96.67	96.58	96.60	96.67	96.56	96.63
**143**	96.47	96.51	96.60	96.55	96.67	96.49	96.51	96.77	96.60	96.79
**144**	95.41	95.38	95.46	95.41	95.54	95.40	95.45	95.64	95.49	95.57
**145**	95.29	95.34	95.37	95.31	95.28	95.28	95.32	95.48	95.42	95.36
**146**	95.56	95.72	95.67	95.57	95.71	95.55	95.55	95.77	95.74	95.74
**147**	96.50	96.54	96.47	96.42	96.51	96.48	96.59	96.59	96.46	96.37
**148**	91.89	91.90	91.91	91.89	92.01	91.85	91.92	91.97	91.83	91.98
**149**	91.87	91.95	92.01	91.92	92.10	91.92	91.97	92.05	91.93	92.03
**150**	91.95	91.87	91.93	91.95	91.95	91.95	92.03	92.00	91.88	91.92
**151**	91.84	91.93	91.88	91.85	91.95	91.88	91.90	92.02	91.93	91.95
**152**	91.84	91.93	91.88	91.85	91.95	91.88	91.90	92.02	91.93	91.95
**153**	91.81	91.90	91.92	91.84	91.84	91.91	91.90	91.90	91.83	91.83
**154**	91.73	91.78	91.77	91.76	91.93	91.81	91.81	91.91	91.77	91.93
**155**	91.79	91.86	91.90	91.82	91.89	91.82	91.73	91.94	91.80	91.88
**156**	91.90	91.89	91.90	91.83	91.94	91.86	91.85	92.04	91.91	91.94
**157**	91.73	91.83	91.86	91.80	91.89	91.82	91.89	91.92	91.77	91.88
**158**	92.05	92.12	92.22	92.09	92.20	92.11	92.07	92.18	92.12	92.13
**159**	94.10	93.99	94.17	94.04	94.17	94.11	94.03	94.13	94.08	94.19
**160**	93.67	93.54	93.73	93.59	93.69	93.64	93.67	93.94	93.73	93.80
**161**	93.60	93.47	93.62	93.56	93.64	93.55	93.64	93.71	93.62	93.71
**162**	83.78	83.78	83.77	83.77	83.87	83.80	83.80	83.88	83.81	83.80
**163**	83.81	83.83	83.78	83.72	83.84	83.79	83.80	83.87	83.82	83.83
**164**	83.93	83.93	83.96	83.93	83.88	83.82	83.81	83.83	83.91	83.92
**165**	83.94	83.94	83.96	83.99	83.95	83.93	83.97	83.92	83.92	83.87
**166**	83.89	83.93	83.98	83.92	83.96	83.88	83.90	83.90	83.86	83.86

Table S3. (continued)

**Isolate**	**81**	**82**	**83**	**84**	**85**	**86**	**87**	**88**	**89**	**90**
**1**	98.46	98.30	98.28	98.36	98.42	98.41	98.35	98.40	98.36	98.37
**2**	98.41	98.28	98.30	98.35	98.38	98.45	98.38	98.40	98.34	98.38
**3**	98.39	98.31	98.21	98.34	98.38	98.41	98.38	98.43	98.43	98.35
**4**	98.49	98.34	98.35	98.43	98.41	98.52	98.42	98.42	98.34	98.46
**5**	98.45	98.33	98.24	98.31	98.35	98.38	98.32	98.36	98.34	98.35
**6**	98.48	98.37	98.37	98.38	98.47	98.47	98.35	98.43	98.43	98.39
**7**	98.40	98.23	98.23	98.26	98.26	98.32	98.30	98.34	98.30	98.29
**8**	98.42	98.37	98.22	98.35	98.37	98.38	98.35	98.37	98.36	98.35
**9**	98.40	98.26	98.28	98.32	98.39	98.40	98.35	98.41	98.40	98.38
**10**	98.46	98.30	98.39	98.35	98.34	98.42	98.41	98.38	98.35	98.43
**11**	98.44	98.35	98.25	98.44	98.41	98.42	98.36	98.39	98.37	98.40
**12**	98.47	98.30	98.28	98.38	98.34	98.43	98.36	98.39	98.31	98.39
**13**	98.48	98.34	98.34	98.38	98.48	98.49	98.44	98.43	98.43	98.40
**14**	98.47	98.34	98.27	98.35	98.41	98.38	98.32	98.44	98.40	98.34
**15**	98.48	98.29	98.30	98.33	98.38	98.40	98.34	98.40	98.33	98.35
**16**	98.38	98.32	98.28	98.30	98.31	98.37	98.31	98.35	98.33	98.34
**17**	98.43	98.38	98.25	98.40	98.42	98.43	98.34	98.38	98.37	98.42
**18**	98.41	98.36	98.33	98.34	98.44	98.39	98.35	98.43	98.42	98.38
**19**	98.47	98.35	98.38	98.35	98.44	98.44	98.41	98.44	98.46	98.35
**20**	98.35	98.24	98.32	98.28	98.35	98.36	98.31	98.46	98.46	98.36
**21**	98.38	98.28	98.29	98.35	98.35	98.38	98.35	98.43	98.46	98.36
**22**	98.38	98.23	98.28	98.29	98.33	98.33	98.29	98.39	98.43	98.29
**23**	98.49	98.36	98.47	98.45	98.44	98.55	98.43	98.50	98.46	98.49
**24**	98.55	98.44	98.47	98.53	98.52	98.53	98.52	98.58	98.59	98.47
**25**	98.69	98.54	98.64	98.66	98.63	98.68	98.63	98.72	98.72	98.62
**26**	98.56	98.47	98.45	98.51	98.49	98.53	98.45	98.55	98.51	98.46
**27**	98.41	98.33	98.37	98.39	98.39	98.43	98.38	98.48	98.50	98.44
**28**	98.44	98.37	98.39	98.41	98.33	98.42	98.36	98.47	98.42	98.40
**29**	98.44	98.36	98.22	98.34	98.38	98.42	98.34	98.44	98.31	98.39
**30**	98.42	98.32	98.33	98.33	98.29	98.41	98.32	98.39	98.35	98.38
**31**	98.42	98.30	98.20	98.32	98.30	98.41	98.33	98.40	98.31	98.38
**32**	98.42	98.25	98.30	98.31	98.35	98.31	98.32	98.41	98.35	98.43
**33**	98.44	98.25	98.26	98.31	98.41	98.32	98.33	98.43	98.35	98.44
**34**	98.57	98.44	98.39	98.42	98.45	98.53	98.46	98.51	98.47	98.54
**35**	98.40	98.31	98.25	98.32	98.36	98.33	98.31	98.38	98.41	98.42
**36**	98.42	98.35	98.27	98.41	98.47	98.39	98.37	98.46	98.47	98.43
**37**	98.40	98.28	98.33	98.25	98.30	98.32	98.36	98.27	98.31	98.32
**38**	98.41	98.29	98.34	98.36	98.36	98.40	98.39	98.40	98.41	98.42
**39**	98.50	98.32	98.39	98.41	98.31	98.40	98.36	98.44	98.41	98.43
**40**	98.54	98.49	98.40	98.42	98.45	98.51	98.50	98.49	98.48	98.49
**41**	98.67	98.56	98.54	98.63	98.64	98.67	98.63	98.61	98.60	98.54

**42**	98.67	98.53	98.55	98.63	98.57	98.61	98.64	98.66	98.60	98.56
**43**	98.65	98.56	98.53	98.63	98.63	98.61	98.63	98.62	98.64	98.57
**44**	98.68	98.56	98.51	98.63	98.61	98.61	98.59	98.62	98.62	98.55
**45**	98.75	98.71	98.66	98.69	98.70	98.75	98.67	98.69	98.68	98.67
**46**	98.68	98.54	98.47	98.65	98.64	98.63	98.62	98.65	98.67	98.59
**47**	98.66	98.60	98.65	98.64	98.71	98.65	98.66	98.65	98.72	98.59
**48**	98.76	98.77	98.72	98.76	98.72	98.72	98.74	98.79	98.80	98.74
**49**	98.72	98.66	98.57	98.65	98.66	98.65	98.68	98.61	98.65	98.58
**50**	98.77	98.69	98.80	98.78	98.73	98.75	98.78	98.79	98.78	98.73
**51**	98.76	98.73	98.65	98.72	98.74	98.70	98.71	98.76	98.72	98.62
**52**	98.63	98.70	98.51	98.57	98.56	98.62	98.60	98.59	98.63	98.60
**53**	98.87	98.77	98.74	98.80	98.87	98.84	98.85	98.82	98.91	98.76
**54**	98.93	98.79	98.80	98.84	98.92	98.90	98.84	98.91	98.93	98.82
**55**	98.73	98.63	98.67	98.71	98.70	98.77	98.68	98.85	98.84	98.73
**56**	98.69	98.64	98.61	98.65	98.67	98.67	98.65	98.77	98.75	98.68
**57**	98.86	98.72	98.82	98.81	98.78	98.84	98.83	98.88	98.84	98.77
**58**	98.87	98.71	98.82	98.80	98.79	98.85	98.82	98.87	98.83	98.81
**59**	99.08	98.59	99.00	99.03	98.99	99.07	99.03	99.08	98.99	98.79
**60**	99.08	98.59	99.00	99.03	98.99	99.07	99.03	99.08	98.99	98.79
**61**	99.04	98.64	98.93	99.05	99.00	99.02	98.96	99.09	99.09	98.85
**62**	99.04	98.66	99.01	99.12	99.01	99.03	99.03	99.04	99.09	98.79
**63**	98.98	98.66	98.98	99.09	99.02	99.05	99.01	99.17	99.14	98.81
**64**	99.01	98.68	98.97	99.02	98.93	99.06	99.03	99.11	99.11	98.87
**65**	98.97	98.67	98.91	99.05	99.00	99.04	99.04	99.12	99.12	98.81
**66**	98.99	98.57	98.86	98.94	98.94	99.02	98.90	98.95	98.98	98.77
**67**	98.95	98.57	98.88	99.00	98.94	99.04	99.02	99.04	99.05	98.82
**68**	98.99	98.67	98.98	99.02	99.08	98.99	99.01	99.09	99.11	98.83
**69**	99.01	98.65	98.92	99.08	98.99	98.98	99.00	99.08	99.13	98.79
**70**	99.08	98.71	98.96	99.11	99.01	99.06	99.06	99.12	99.15	98.82
**71**	98.98	98.63	98.88	99.01	98.99	98.95	98.92	98.91	99.02	98.75
**72**	98.95	98.60	98.84	98.96	99.00	98.93	98.92	99.00	99.08	98.73
**73**	99.08	98.70	99.09	99.13	99.09	99.14	99.12	99.17	99.23	98.85
**74**	99.02	98.61	98.82	99.03	98.92	99.00	99.02	99.08	99.06	98.79
**75**	99.19	98.79	99.05	99.10	99.05	99.10	99.16	98.98	99.01	98.89
**76**	98.96	98.65	98.95	98.94	99.04	98.93	99.07	99.00	98.99	98.80
**77**	98.97	98.58	98.93	98.89	98.89	98.95	99.03	98.94	98.96	98.80
**78**	99.06	98.66	98.92	98.95	98.91	99.01	98.97	98.94	98.97	98.84
**79**	98.95	98.58	98.95	98.93	98.93	98.89	98.95	99.03	99.03	98.75
**80**	98.80	98.90	98.59	98.68	98.68	98.73	98.70	98.67	98.68	98.71
**81**	100.00	98.73	99.00	99.02	98.97	99.03	99.02	98.99	99.00	98.83
**82**	98.73	100.00	98.66	98.57	98.63	98.62	98.68	98.62	98.54	98.50
**83**	99.00	98.66	100.00	98.89	98.83	98.89	98.92	98.96	98.96	98.79
**84**	99.02	98.57	98.89	100.00	99.01	99.07	98.98	98.94	98.95	98.76

**85**	98.97	98.63	98.83	99.01	100.00	99.02	99.05	99.04	98.99	98.91
**86**	99.03	98.62	98.89	99.07	99.02	100.00	99.04	99.06	99.09	98.89
**87**	99.02	98.68	98.92	98.98	99.05	99.04	100.00	98.99	99.01	98.88
**88**	98.99	98.62	98.96	98.94	99.04	99.06	98.99	100.00	99.90	98.84
**89**	99.00	98.54	98.96	98.95	98.99	99.09	99.01	99.90	100.00	98.79
**90**	98.83	98.50	98.79	98.76	98.91	98.89	98.88	98.84	98.79	100.00
**91**	98.76	98.42	98.72	98.72	98.80	98.83	98.82	98.81	98.76	99.92
**92**	98.88	98.53	98.77	98.76	98.90	98.91	98.91	98.88	98.77	99.99
**93**	98.86	98.52	98.71	98.74	98.86	98.84	98.82	98.77	98.80	99.66
**94**	98.80	98.51	98.67	98.72	98.86	98.89	98.80	98.81	98.78	99.72
**95**	98.97	98.66	98.80	98.93	99.00	98.97	98.97	98.86	98.89	99.54
**96**	99.00	98.71	98.88	98.94	99.03	99.09	99.03	98.96	98.97	99.61
**97**	98.88	98.61	98.83	98.87	98.96	98.95	98.93	98.90	98.90	99.23
**98**	99.05	98.76	98.95	98.97	98.92	99.05	98.99	99.00	98.99	99.09
**99**	98.96	98.68	98.87	98.87	98.85	99.01	98.90	98.90	98.91	99.04
**100**	98.91	98.58	98.82	98.84	98.86	98.94	98.89	98.88	98.86	98.95
**101**	98.96	98.59	98.81	98.76	98.86	98.93	98.88	98.90	98.82	98.94
**102**	99.05	98.73	98.86	98.82	98.93	98.97	98.91	98.90	98.85	98.94
**103**	98.98	98.64	98.88	98.87	98.88	98.93	98.87	98.96	98.91	99.06
**104**	98.81	98.65	98.75	98.71	98.76	98.80	98.80	98.76	98.71	98.71
**105**	98.86	98.68	98.83	98.69	98.90	98.90	98.87	98.91	98.92	98.90
**106**	98.95	98.74	98.84	98.88	98.96	98.95	98.90	98.94	98.93	98.93
**107**	98.54	98.45	98.44	98.55	98.44	98.55	98.58	98.58	98.55	98.52
**108**	98.55	98.45	98.42	98.58	98.54	98.57	98.54	98.59	98.65	98.54
**109**	98.63	98.55	98.57	98.62	98.55	98.61	98.63	98.66	98.62	98.61
**110**	98.61	98.48	98.42	98.59	98.54	98.55	98.54	98.65	98.58	98.53
**111**	98.60	98.55	98.44	98.65	98.57	98.59	98.57	98.66	98.65	98.59
**112**	98.62	98.55	98.49	98.63	98.56	98.57	98.56	98.62	98.62	98.53
**113**	98.62	98.51	98.47	98.65	98.56	98.58	98.55	98.60	98.64	98.55
**114**	98.61	98.56	98.45	98.62	98.53	98.61	98.55	98.65	98.67	98.56
**115**	98.62	98.50	98.47	98.64	98.60	98.62	98.57	98.68	98.67	98.58
**116**	98.67	98.57	98.49	98.57	98.56	98.62	98.60	98.61	98.58	98.59
**117**	98.60	98.53	98.50	98.47	98.59	98.54	98.56	98.56	98.51	98.48
**118**	98.52	98.57	98.53	98.49	98.56	98.55	98.62	98.62	98.60	98.41
**119**	97.04	97.10	96.94	97.02	96.94	97.02	97.03	96.98	97.01	97.01
**120**	97.05	97.07	96.90	97.08	96.96	96.95	96.96	96.98	96.97	96.95
**121**	96.37	96.29	95.79	96.12	96.05	96.13	96.14	96.14	96.24	96.16
**122**	96.93	97.02	96.86	96.86	96.80	96.97	96.92	96.95	96.87	96.90
**123**	97.16	97.14	97.14	97.17	97.19	97.17	97.18	97.19	97.14	97.19
**124**	97.21	97.18	97.12	97.14	97.13	97.21	97.19	97.22	97.16	97.25
**125**	97.27	97.20	97.16	97.18	97.22	97.20	97.11	97.23	97.19	97.24
**126**	97.31	97.29	97.18	97.30	97.28	97.31	97.28	97.27	97.29	97.26
**127**	97.30	97.20	97.22	97.22	97.19	97.28	97.24	97.15	97.16	97.22

**128**	96.83	96.77	96.79	96.85	96.81	96.82	96.80	96.82	96.79	96.96
**129**	96.98	96.98	96.91	97.02	96.99	96.97	96.87	97.02	96.95	97.05
**130**	96.97	96.92	96.91	97.02	96.96	97.04	96.88	96.96	96.93	97.05
**131**	96.89	96.81	96.80	96.90	96.94	96.88	96.89	96.91	96.89	96.99
**132**	96.98	96.85	96.84	96.92	96.92	96.92	96.89	96.91	96.89	97.02
**133**	97.08	96.98	96.97	97.02	97.02	97.07	97.05	97.01	96.96	97.22
**134**	96.97	96.94	96.90	96.92	96.90	96.92	96.94	96.93	96.86	97.03
**135**	96.96	96.93	96.88	96.87	96.89	96.91	96.89	96.94	96.87	97.05
**136**	96.86	96.85	96.80	96.78	96.82	96.82	96.83	96.89	96.78	96.99
**137**	96.49	96.42	96.34	96.42	96.50	96.39	96.39	96.48	96.49	96.49
**138**	96.56	96.51	96.35	96.45	96.50	96.41	96.44	96.52	96.48	96.49
**139**	96.57	96.51	96.55	96.49	96.56	96.52	96.53	96.56	96.56	96.57
**140**	96.58	96.54	96.44	96.57	96.60	96.48	96.58	96.55	96.57	96.61
**141**	96.29	96.28	96.22	96.16	96.27	96.23	96.28	96.32	96.31	96.36
**142**	96.58	96.55	96.43	96.58	96.58	96.54	96.54	96.58	96.57	96.56
**143**	96.58	96.61	96.48	96.51	96.61	96.55	96.57	96.60	96.60	96.69
**144**	95.61	95.56	95.41	95.47	95.51	95.49	95.46	95.48	95.49	95.56
**145**	95.44	95.40	95.26	95.35	95.40	95.29	95.33	95.37	95.46	95.45
**146**	95.66	95.66	95.56	95.65	95.60	95.66	95.62	95.58	95.67	95.70
**147**	96.58	96.54	96.47	96.50	96.47	96.53	96.48	96.54	96.42	96.54
**148**	91.94	92.01	91.80	91.90	91.89	91.91	91.96	91.95	91.89	92.00
**149**	91.99	91.95	91.79	91.99	91.90	91.96	91.93	91.93	91.93	91.97
**150**	91.93	91.98	91.86	91.96	91.96	91.89	91.97	91.98	91.99	91.96
**151**	91.96	91.94	91.79	91.91	91.89	91.90	91.90	91.88	91.83	91.95
**152**	91.96	91.94	91.79	91.91	91.89	91.90	91.90	91.88	91.83	91.95
**153**	91.84	91.77	91.83	91.99	91.79	91.93	91.97	91.87	91.94	91.87
**154**	91.89	91.89	91.73	91.80	91.78	91.80	91.82	91.77	91.77	91.79
**155**	91.82	91.84	91.75	91.88	91.83	91.84	91.90	91.83	91.81	91.79
**156**	92.01	91.94	91.81	91.92	91.90	91.89	91.93	91.95	91.86	91.86
**157**	91.91	91.87	91.71	91.94	91.82	91.87	91.84	91.77	91.79	91.86
**158**	92.13	92.07	92.11	92.15	92.09	92.15	92.15	92.15	92.14	92.14
**159**	94.11	94.12	94.05	94.08	94.01	94.07	94.18	94.09	94.10	94.10
**160**	93.79	93.83	93.51	93.68	93.69	93.57	93.64	93.80	93.85	93.79
**161**	93.70	93.68	93.56	93.62	93.67	93.75	93.68	93.71	93.65	93.67
**162**	83.84	83.78	83.80	83.76	83.85	83.76	83.83	83.75	83.82	83.88
**163**	83.89	83.80	83.83	83.79	83.84	83.78	83.83	83.73	83.78	83.85
**164**	83.88	83.86	83.96	83.83	83.87	83.90	83.89	83.87	83.96	83.93
**165**	83.89	83.91	83.82	83.88	83.85	83.88	83.93	83.87	83.84	83.94
**166**	83.92	83.86	84.04	83.90	83.86	83.98	83.91	83.84	83.86	83.95

Table S3. (continued)

Isolate	91	92	93	94	95	96	97	98	99	100
**1**	98.32	98.38	98.32	98.27	98.47	98.45	98.44	98.54	98.47	98.47
**2**	98.30	98.38	98.25	98.27	98.38	98.45	98.40	98.49	98.46	98.44
**3**	98.33	98.36	98.25	98.28	98.49	98.48	98.47	98.55	98.46	98.45
**4**	98.35	98.45	98.35	98.36	98.48	98.51	98.50	98.57	98.49	98.49
**5**	98.28	98.36	98.21	98.19	98.49	98.45	98.41	98.57	98.49	98.40
**6**	98.33	98.41	98.37	98.35	98.55	98.52	98.45	98.60	98.56	98.50
**7**	98.22	98.26	98.19	98.14	98.45	98.39	98.38	98.49	98.37	98.36
**8**	98.30	98.36	98.30	98.31	98.42	98.40	98.45	98.50	98.52	98.47
**9**	98.29	98.36	98.26	98.26	98.49	98.47	98.45	98.54	98.42	98.35
**10**	98.33	98.41	98.33	98.38	98.50	98.55	98.47	98.59	98.54	98.50
**11**	98.37	98.44	98.38	98.35	98.50	98.47	98.49	98.53	98.51	98.44
**12**	98.32	98.36	98.31	98.27	98.48	98.49	98.48	98.57	98.50	98.42
**13**	98.32	98.41	98.31	98.29	98.49	98.52	98.45	98.61	98.51	98.47
**14**	98.28	98.36	98.34	98.30	98.51	98.51	98.42	98.55	98.50	98.43
**15**	98.28	98.35	98.29	98.25	98.48	98.50	98.46	98.53	98.53	98.41
**16**	98.24	98.32	98.21	98.23	98.45	98.47	98.43	98.48	98.36	98.38
**17**	98.33	98.42	98.32	98.24	98.44	98.49	98.46	98.55	98.47	98.45
**18**	98.31	98.41	98.35	98.24	98.42	98.45	98.41	98.53	98.52	98.46
**19**	98.33	98.34	98.36	98.37	98.55	98.53	98.46	98.62	98.53	98.49
**20**	98.32	98.36	98.30	98.33	98.48	98.43	98.39	98.49	98.48	98.39
**21**	98.31	98.36	98.30	98.27	98.50	98.46	98.41	98.52	98.42	98.42
**22**	98.24	98.35	98.29	98.25	98.47	98.48	98.39	98.50	98.40	98.35
**23**	98.41	98.51	98.48	98.46	98.55	98.62	98.52	98.62	98.53	98.51
**24**	98.45	98.51	98.45	98.49	98.67	98.55	98.56	98.67	98.62	98.57
**25**	98.57	98.65	98.59	98.61	98.72	98.71	98.70	98.73	98.73	98.72
**26**	98.42	98.49	98.48	98.43	98.57	98.60	98.55	98.63	98.58	98.57
**27**	98.38	98.44	98.32	98.38	98.52	98.58	98.49	98.61	98.51	98.48
**28**	98.35	98.42	98.36	98.35	98.49	98.48	98.43	98.61	98.46	98.46
**29**	98.36	98.39	98.39	98.33	98.45	98.55	98.45	98.58	98.54	98.49
**30**	98.33	98.35	98.36	98.31	98.50	98.51	98.38	98.60	98.54	98.44
**31**	98.36	98.40	98.38	98.29	98.45	98.54	98.42	98.58	98.55	98.43
**32**	98.38	98.44	98.32	98.29	98.43	98.49	98.48	98.60	98.46	98.45
**33**	98.36	98.42	98.30	98.22	98.48	98.48	98.47	98.63	98.49	98.47
**34**	98.50	98.57	98.51	98.49	98.55	98.59	98.62	98.61	98.62	98.64
**35**	98.37	98.43	98.35	98.22	98.41	98.46	98.40	98.56	98.45	98.41
**36**	98.39	98.45	98.31	98.29	98.50	98.45	98.42	98.56	98.49	98.45
**37**	98.23	98.33	98.30	98.24	98.41	98.46	98.39	98.52	98.46	98.39
**38**	98.40	98.45	98.35	98.32	98.48	98.55	98.48	98.58	98.55	98.50
**39**	98.38	98.44	98.31	98.34	98.55	98.44	98.48	98.63	98.52	98.47
**40**	98.43	98.48	98.41	98.42	98.60	98.62	98.55	98.69	98.64	98.56
**41**	98.51	98.60	98.45	98.43	98.67	98.74	98.60	98.74	98.64	98.56

**42**	98.50	98.57	98.46	98.48	98.67	98.74	98.62	98.72	98.63	98.57
**43**	98.51	98.60	98.48	98.52	98.68	98.73	98.66	98.74	98.63	98.60
**44**	98.51	98.57	98.46	98.49	98.68	98.75	98.62	98.76	98.61	98.58
**45**	98.59	98.67	98.58	98.64	98.74	98.78	98.77	98.77	98.72	98.73
**46**	98.52	98.58	98.46	98.46	98.69	98.76	98.64	98.77	98.66	98.58
**47**	98.58	98.61	98.55	98.53	98.69	98.75	98.67	98.74	98.71	98.63
**48**	98.68	98.75	98.67	98.65	98.74	98.79	98.79	98.78	98.74	98.73
**49**	98.52	98.59	98.54	98.45	98.67	98.78	98.61	98.79	98.73	98.64
**50**	98.66	98.75	98.63	98.64	98.78	98.76	98.78	98.83	98.76	98.75
**51**	98.58	98.64	98.61	98.60	98.79	98.75	98.74	98.80	98.76	98.69
**52**	98.48	98.58	98.51	98.47	98.64	98.66	98.61	98.70	98.62	98.63
**53**	98.71	98.78	98.74	98.77	98.85	98.90	98.86	98.88	98.85	98.84
**54**	98.77	98.85	98.76	98.74	98.93	98.91	98.94	98.90	98.83	98.82
**55**	98.67	98.78	98.66	98.59	98.75	98.83	98.78	98.77	98.77	98.73
**56**	98.61	98.67	98.57	98.50	98.64	98.75	98.72	98.74	98.63	98.61
**57**	98.72	98.79	98.77	98.76	98.81	98.85	98.87	98.81	98.81	98.82
**58**	98.71	98.81	98.78	98.73	98.79	98.84	98.87	98.81	98.81	98.82
**59**	98.75	98.82	98.77	98.77	98.96	99.00	98.88	98.96	98.87	98.85
**60**	98.75	98.82	98.77	98.77	98.96	99.00	98.88	98.96	98.87	98.85
**61**	98.82	98.88	98.81	98.72	99.00	98.99	98.90	98.98	98.93	98.88
**62**	98.76	98.83	98.84	98.83	98.98	98.96	98.94	98.97	98.88	98.86
**63**	98.75	98.81	98.76	98.78	98.93	98.98	98.87	98.98	98.88	98.82
**64**	98.83	98.89	98.78	98.77	99.02	99.01	98.91	98.97	98.93	98.90
**65**	98.76	98.81	98.80	98.73	98.97	99.00	98.86	98.98	98.88	98.81
**66**	98.76	98.80	98.81	98.74	98.90	98.93	98.83	98.93	98.91	98.85
**67**	98.75	98.81	98.73	98.72	98.90	98.95	98.83	98.96	98.86	98.77
**68**	98.74	98.82	98.81	98.77	98.86	98.86	98.91	98.93	98.79	98.79
**69**	98.73	98.82	98.75	98.71	98.90	98.86	98.84	98.92	98.77	98.79
**70**	98.75	98.84	98.80	98.72	98.91	98.99	98.91	98.96	98.87	98.85
**71**	98.64	98.76	98.70	98.61	98.82	98.88	98.76	98.89	98.73	98.68
**72**	98.67	98.73	98.63	98.61	98.91	98.85	98.79	98.91	98.72	98.71
**73**	98.85	98.89	98.81	98.85	98.95	98.97	98.95	98.99	98.91	98.87
**74**	98.73	98.85	98.68	98.67	98.86	98.93	98.91	98.88	98.79	98.85
**75**	98.85	98.92	98.92	98.95	99.02	99.01	99.00	99.06	98.98	99.01
**76**	98.75	98.79	98.75	98.75	98.93	98.97	98.88	98.95	98.86	98.83
**77**	98.74	98.83	98.76	98.71	98.88	98.95	98.87	98.98	98.85	98.84
**78**	98.80	98.85	98.80	98.82	98.94	98.98	98.91	99.00	98.88	98.90
**79**	98.71	98.80	98.75	98.69	98.86	98.95	98.84	98.91	98.79	98.78
**80**	98.65	98.71	98.63	98.61	98.75	98.77	98.78	98.80	98.74	98.78
**81**	98.76	98.88	98.86	98.80	98.97	99.00	98.88	99.05	98.96	98.91
**82**	98.42	98.53	98.52	98.51	98.66	98.71	98.61	98.76	98.68	98.58
**83**	98.72	98.77	98.71	98.67	98.80	98.88	98.83	98.95	98.87	98.82
**84**	98.72	98.76	98.74	98.72	98.93	98.94	98.87	98.97	98.87	98.84

**85**	98.80	98.90	98.86	98.86	99.00	99.03	98.96	98.92	98.85	98.86
**86**	98.83	98.91	98.84	98.89	98.97	99.09	98.95	99.05	99.01	98.94
**87**	98.82	98.91	98.82	98.80	98.97	99.03	98.93	98.99	98.90	98.89
**88**	98.81	98.88	98.77	98.81	98.86	98.96	98.90	99.00	98.90	98.88
**89**	98.76	98.77	98.80	98.78	98.89	98.97	98.90	98.99	98.91	98.86
**90**	99.92	99.99	99.66	99.72	99.54	99.61	99.23	99.09	99.04	98.95
**91**	100.00	99.92	99.63	99.67	99.48	99.52	99.16	99.06	98.97	98.92
**92**	99.92	100.00	99.70	99.72	99.55	99.59	99.22	99.13	99.03	98.96
**93**	99.63	99.70	100.00	99.83	99.53	99.56	99.14	99.09	98.98	98.91
**94**	99.67	99.72	99.83	100.00	99.50	99.56	99.13	99.08	99.01	98.87
**95**	99.48	99.55	99.53	99.50	100.00	99.84	99.37	99.21	99.16	99.10
**96**	99.52	99.59	99.56	99.56	99.84	100.00	99.44	99.29	99.13	99.15
**97**	99.16	99.22	99.14	99.13	99.37	99.44	100.00	99.24	99.13	99.12
**98**	99.06	99.13	99.09	99.08	99.21	99.29	99.24	100.00	99.97	99.94
**99**	98.97	99.03	98.98	99.01	99.16	99.13	99.13	99.97	100.00	99.91
**100**	98.92	98.96	98.91	98.87	99.10	99.15	99.12	99.94	99.91	100.00
**101**	98.91	98.95	98.95	98.84	99.10	99.19	99.07	99.92	99.89	99.88
**102**	98.89	98.94	99.01	99.02	99.14	99.19	99.06	99.58	99.50	99.48
**103**	98.96	99.04	99.04	98.99	99.16	99.20	99.06	99.15	99.10	99.08
**104**	98.66	98.72	98.71	98.71	98.83	98.89	98.88	98.88	98.80	98.78
**105**	98.83	98.88	98.89	98.88	99.02	99.00	98.97	99.06	99.05	98.97
**106**	98.86	98.93	98.96	98.90	99.05	99.04	99.02	99.08	99.01	99.01
**107**	98.48	98.56	98.54	98.49	98.60	98.70	98.57	98.72	98.63	98.62
**108**	98.49	98.55	98.53	98.50	98.62	98.66	98.55	98.68	98.62	98.51
**109**	98.60	98.65	98.58	98.56	98.76	98.75	98.66	98.74	98.73	98.71
**110**	98.50	98.55	98.54	98.48	98.60	98.66	98.59	98.69	98.60	98.59
**111**	98.52	98.61	98.59	98.56	98.66	98.69	98.62	98.76	98.63	98.63
**112**	98.52	98.57	98.59	98.59	98.61	98.70	98.60	98.71	98.63	98.60
**113**	98.54	98.61	98.52	98.52	98.62	98.72	98.60	98.69	98.64	98.61
**114**	98.50	98.56	98.59	98.51	98.63	98.64	98.59	98.74	98.60	98.61
**115**	98.54	98.60	98.55	98.55	98.65	98.74	98.62	98.73	98.65	98.62
**116**	98.51	98.65	98.59	98.56	98.71	98.70	98.63	98.71	98.61	98.64
**117**	98.38	98.44	98.45	98.43	98.57	98.63	98.55	98.67	98.56	98.53
**118**	98.39	98.46	98.44	98.47	98.55	98.57	98.52	98.62	98.50	98.51
**119**	96.91	97.01	96.97	96.98	96.97	97.04	97.00	97.05	97.00	97.06
**120**	96.87	96.91	96.97	96.93	97.03	97.03	96.97	97.10	96.96	97.02
**121**	96.10	96.02	96.18	96.31	96.02	96.27	96.27	96.37	96.26	96.28
**122**	96.85	96.95	96.89	96.88	96.93	96.99	96.86	97.05	97.02	96.97
**123**	97.16	97.16	97.17	97.18	97.22	97.24	97.22	97.26	97.25	97.17
**124**	97.16	97.18	97.14	97.16	97.19	97.21	97.19	97.26	97.21	97.15
**125**	97.13	97.21	97.21	97.15	97.22	97.20	97.25	97.33	97.21	97.28
**126**	97.20	97.25	97.31	97.28	97.26	97.33	97.25	97.35	97.24	97.27
**127**	97.14	97.19	97.19	97.24	97.26	97.25	97.24	97.26	97.18	97.23

**128**	96.87	96.99	96.93	96.85	96.84	96.87	96.77	96.96	96.91	96.82
**129**	96.98	97.04	97.10	97.05	96.95	96.91	96.93	97.07	96.94	96.97
**130**	97.02	97.08	97.08	97.05	96.98	96.96	96.96	97.08	97.06	97.01
**131**	96.90	96.98	96.95	96.93	96.89	96.93	96.87	97.00	96.92	96.86
**132**	96.95	97.05	97.03	96.93	96.91	96.94	96.92	97.04	97.00	96.99
**133**	97.08	97.17	97.17	97.16	97.05	97.06	97.06	97.08	97.09	97.10
**134**	96.95	97.03	97.07	97.01	96.92	96.93	96.93	97.03	97.02	96.95
**135**	96.94	97.03	97.07	96.98	96.88	96.95	96.94	97.03	96.99	96.95
**136**	96.91	96.93	96.98	96.92	96.87	96.86	96.82	96.97	96.92	96.86
**137**	96.48	96.54	96.48	96.49	96.56	96.50	96.49	96.59	96.57	96.46
**138**	96.42	96.45	96.54	96.52	96.57	96.51	96.51	96.68	96.52	96.46
**139**	96.53	96.59	96.54	96.46	96.58	96.63	96.58	96.66	96.61	96.53
**140**	96.52	96.60	96.58	96.56	96.58	96.63	96.59	96.62	96.54	96.55
**141**	96.27	96.31	96.29	96.26	96.28	96.35	96.32	96.31	96.30	96.31
**142**	96.52	96.57	96.55	96.56	96.61	96.56	96.50	96.61	96.64	96.49
**143**	96.63	96.74	96.65	96.63	96.66	96.69	96.69	96.79	96.68	96.68
**144**	95.45	95.47	95.52	95.57	95.51	95.54	95.58	95.61	95.53	95.55
**145**	95.40	95.39	95.42	95.41	95.41	95.48	95.43	95.45	95.41	95.36
**146**	95.67	95.73	95.70	95.67	95.72	95.69	95.69	95.83	95.84	95.61
**147**	96.51	96.45	96.46	96.53	96.47	96.48	96.68	96.48	96.57	96.48
**148**	91.87	91.93	91.91	91.95	91.91	91.98	91.97	92.00	92.03	91.92
**149**	91.94	92.01	92.04	92.01	92.03	92.02	92.01	92.04	91.98	91.94
**150**	91.88	91.94	91.94	91.95	91.94	91.87	91.93	91.97	91.94	91.96
**151**	91.86	91.91	91.92	91.89	91.92	91.91	91.97	91.90	91.94	91.92
**152**	91.86	91.91	91.92	91.89	91.92	91.91	91.97	91.90	91.94	91.92
**153**	91.86	91.84	91.93	91.90	91.98	91.92	91.88	91.87	92.01	91.83
**154**	91.76	91.80	91.83	91.79	91.84	91.86	91.80	91.88	91.82	91.85
**155**	91.67	91.80	91.83	91.74	91.84	91.87	91.82	91.87	91.86	91.81
**156**	91.77	91.91	91.95	91.97	91.87	91.94	91.92	91.94	91.89	91.90
**157**	91.85	91.81	91.87	91.94	91.94	91.90	91.85	91.86	91.97	91.76
**158**	92.05	92.11	92.08	92.07	92.10	92.17	92.11	92.19	92.15	92.07
**159**	94.03	94.06	94.03	94.05	94.10	94.07	94.07	94.19	94.07	94.02
**160**	93.77	93.73	93.75	93.73	93.76	93.72	93.71	93.83	93.90	93.81
**161**	93.61	93.65	93.72	93.65	93.68	93.74	93.71	93.73	93.68	93.72
**162**	83.77	83.79	83.75	83.85	83.83	83.77	83.84	83.80	83.74	83.78
**163**	83.71	83.81	83.81	83.86	83.81	83.82	83.82	83.87	83.85	83.81
**164**	83.85	83.90	83.91	83.91	83.94	84.04	83.80	83.95	83.90	83.87
**165**	83.86	83.89	83.89	83.86	83.91	83.91	83.88	83.98	83.88	83.86
**166**	83.82	83.93	83.83	83.95	83.85	83.82	83.88	83.93	83.82	83.86

Table S3. (continued)

**Isolate**	**101**	**102**	**103**	**104**	**105**	**106**	**107**	**108**	**109**	**110**
**1**	98.42	98.46	98.43	98.33	98.50	98.49	98.35	98.30	98.44	98.32
**2**	98.42	98.41	98.45	98.29	98.48	98.45	98.30	98.30	98.38	98.29
**3**	98.42	98.45	98.37	98.36	98.45	98.50	98.34	98.36	98.45	98.36
**4**	98.47	98.51	98.44	98.43	98.49	98.50	98.42	98.40	98.52	98.40
**5**	98.38	98.43	98.35	98.38	98.39	98.44	98.25	98.20	98.41	98.32
**6**	98.50	98.47	98.45	98.39	98.49	98.54	98.31	98.31	98.43	98.39
**7**	98.25	98.34	98.36	98.27	98.29	98.39	98.22	98.14	98.35	98.27
**8**	98.39	98.47	98.46	98.35	98.46	98.48	98.29	98.29	98.46	98.32
**9**	98.34	98.44	98.39	98.35	98.39	98.50	98.24	98.24	98.42	98.32
**10**	98.50	98.55	98.42	98.39	98.50	98.49	98.29	98.31	98.47	98.34
**11**	98.42	98.50	98.44	98.40	98.48	98.50	98.31	98.38	98.51	98.35
**12**	98.41	98.43	98.47	98.41	98.43	98.47	98.32	98.29	98.43	98.31
**13**	98.47	98.47	98.44	98.40	98.50	98.51	98.37	98.38	98.46	98.36
**14**	98.41	98.45	98.43	98.35	98.49	98.49	98.38	98.35	98.42	98.36
**15**	98.40	98.44	98.44	98.37	98.43	98.52	98.31	98.35	98.43	98.36
**16**	98.34	98.46	98.46	98.36	98.46	98.48	98.26	98.29	98.44	98.37
**17**	98.42	98.46	98.44	98.39	98.42	98.49	98.28	98.29	98.45	98.33
**18**	98.41	98.43	98.40	98.32	98.49	98.53	98.29	98.25	98.43	98.31
**19**	98.42	98.46	98.51	98.37	98.51	98.58	98.35	98.34	98.47	98.37
**20**	98.36	98.44	98.39	98.28	98.41	98.48	98.26	98.35	98.42	98.34
**21**	98.34	98.38	98.37	98.35	98.36	98.42	98.30	98.33	98.41	98.39
**22**	98.32	98.39	98.42	98.32	98.35	98.40	98.35	98.35	98.41	98.41
**23**	98.51	98.55	98.51	98.45	98.53	98.55	98.42	98.39	98.56	98.40
**24**	98.59	98.62	98.50	98.42	98.64	98.63	98.50	98.53	98.55	98.48
**25**	98.72	98.73	98.67	98.64	98.67	98.66	98.63	98.60	98.63	98.60
**26**	98.55	98.54	98.51	98.50	98.58	98.59	98.49	98.44	98.52	98.50
**27**	98.50	98.50	98.51	98.43	98.47	98.52	98.35	98.40	98.57	98.44
**28**	98.40	98.50	98.41	98.38	98.51	98.50	98.41	98.40	98.45	98.42
**29**	98.45	98.46	98.44	98.35	98.41	98.51	98.37	98.34	98.49	98.38
**30**	98.34	98.49	98.48	98.33	98.37	98.45	98.34	98.23	98.44	98.36
**31**	98.42	98.42	98.45	98.34	98.40	98.51	98.31	98.33	98.50	98.36
**32**	98.44	98.45	98.40	98.35	98.37	98.44	98.25	98.27	98.45	98.26
**33**	98.43	98.43	98.43	98.33	98.39	98.44	98.34	98.27	98.45	98.30
**34**	98.62	98.57	98.52	98.49	98.47	98.50	98.41	98.42	98.52	98.38
**35**	98.45	98.49	98.38	98.29	98.40	98.48	98.30	98.23	98.36	98.31
**36**	98.40	98.44	98.45	98.41	98.43	98.50	98.35	98.29	98.46	98.38
**37**	98.36	98.40	98.40	98.27	98.43	98.51	98.27	98.26	98.38	98.25
**38**	98.52	98.52	98.46	98.33	98.44	98.52	98.32	98.30	98.45	98.35
**39**	98.49	98.53	98.44	98.34	98.50	98.51	98.35	98.29	98.50	98.26
**40**	98.54	98.52	98.53	98.47	98.55	98.58	98.45	98.44	98.51	98.50
**41**	98.60	98.65	98.66	98.66	98.63	98.73	98.71	98.65	98.69	98.77

**42**	98.59	98.65	98.62	98.63	98.65	98.72	98.68	98.70	98.74	98.76
**43**	98.65	98.62	98.65	98.65	98.64	98.72	98.75	98.73	98.80	98.81
**44**	98.62	98.61	98.63	98.66	98.63	98.70	98.76	98.71	98.78	98.84
**45**	98.70	98.71	98.73	98.77	98.73	98.78	98.83	98.81	98.85	98.89
**46**	98.61	98.62	98.64	98.68	98.64	98.70	98.75	98.74	98.69	98.82
**47**	98.64	98.67	98.61	98.65	98.78	98.78	98.62	98.65	98.75	98.72
**48**	98.73	98.79	98.68	98.83	98.81	98.86	98.81	98.80	98.85	98.85
**49**	98.62	98.72	98.62	98.76	98.72	98.78	98.63	98.56	98.67	98.70
**50**	98.75	98.80	98.71	98.78	98.78	98.83	98.81	98.80	98.89	98.77
**51**	98.63	98.74	98.69	98.71	98.77	98.82	98.73	98.71	98.80	98.78
**52**	98.58	98.71	98.56	98.61	98.68	98.74	98.59	98.59	98.72	98.48
**53**	98.86	98.84	98.82	98.89	98.86	98.88	98.84	98.82	98.87	98.82
**54**	98.87	98.84	98.82	98.97	98.84	98.90	98.81	98.74	98.81	98.76
**55**	98.73	98.72	98.75	98.77	98.81	98.84	98.56	98.57	98.60	98.50
**56**	98.61	98.64	98.64	98.74	98.68	98.73	98.58	98.55	98.55	98.60
**57**	98.84	98.86	98.80	98.80	98.87	98.88	98.68	98.66	98.78	98.62
**58**	98.82	98.87	98.80	98.80	98.87	98.87	98.68	98.70	98.79	98.67
**59**	98.81	98.94	98.92	98.78	98.92	98.98	98.59	98.61	98.64	98.61
**60**	98.81	98.94	98.92	98.78	98.92	98.98	98.59	98.61	98.64	98.61
**61**	98.83	98.92	98.92	98.77	98.84	98.93	98.55	98.54	98.62	98.62
**62**	98.86	98.97	98.89	98.76	98.84	98.94	98.57	98.58	98.66	98.56
**63**	98.79	98.87	98.90	98.77	98.87	98.97	98.55	98.52	98.63	98.65
**64**	98.83	98.88	98.93	98.71	98.82	98.92	98.56	98.57	98.66	98.63
**65**	98.77	98.87	98.90	98.75	98.91	98.99	98.57	98.54	98.62	98.63
**66**	98.79	98.87	98.92	98.69	98.82	98.93	98.55	98.63	98.66	98.63
**67**	98.80	98.87	98.89	98.68	98.82	98.91	98.45	98.49	98.63	98.57
**68**	98.80	98.89	98.89	98.76	98.89	98.93	98.67	98.62	98.63	98.67
**69**	98.84	98.92	98.84	98.71	98.83	98.84	98.50	98.58	98.71	98.56
**70**	98.78	98.93	98.90	98.74	98.86	98.95	98.62	98.62	98.68	98.56
**71**	98.74	98.89	98.79	98.69	98.75	98.78	98.49	98.44	98.58	98.47
**72**	98.71	98.89	98.82	98.58	98.76	98.83	98.50	98.46	98.63	98.54
**73**	98.87	98.92	98.94	98.85	98.96	98.96	98.69	98.68	98.71	98.70
**74**	98.77	98.91	98.80	98.68	98.85	98.90	98.48	98.48	98.57	98.54
**75**	98.98	99.10	99.01	98.94	98.99	99.01	98.67	98.66	98.78	98.70
**76**	98.84	98.85	98.88	98.78	98.94	99.00	98.58	98.57	98.63	98.64
**77**	98.83	98.86	98.88	98.74	98.92	98.97	98.54	98.55	98.60	98.58
**78**	98.84	98.93	98.90	98.82	98.91	98.89	98.62	98.62	98.71	98.63
**79**	98.85	98.91	98.84	98.72	98.89	98.92	98.58	98.56	98.56	98.66
**80**	98.75	98.74	98.72	98.72	98.72	98.75	98.56	98.56	98.68	98.58
**81**	98.96	99.05	98.98	98.81	98.86	98.95	98.54	98.55	98.63	98.61
**82**	98.59	98.73	98.64	98.65	98.68	98.74	98.45	98.45	98.55	98.48
**83**	98.81	98.86	98.88	98.75	98.83	98.84	98.44	98.42	98.57	98.42
**84**	98.76	98.82	98.87	98.71	98.69	98.88	98.55	98.58	98.62	98.59

**85**	98.86	98.93	98.88	98.76	98.90	98.96	98.44	98.54	98.55	98.54
**86**	98.93	98.97	98.93	98.80	98.90	98.95	98.55	98.57	98.61	98.55
**87**	98.88	98.91	98.87	98.80	98.87	98.90	98.58	98.54	98.63	98.54
**88**	98.90	98.90	98.96	98.76	98.91	98.94	98.58	98.59	98.66	98.65
**89**	98.82	98.85	98.91	98.71	98.92	98.93	98.55	98.65	98.62	98.58
**90**	98.94	98.94	99.06	98.71	98.90	98.93	98.52	98.54	98.61	98.53
**91**	98.91	98.89	98.96	98.66	98.83	98.86	98.48	98.49	98.60	98.50
**92**	98.95	98.94	99.04	98.72	98.88	98.93	98.56	98.55	98.65	98.55
**93**	98.95	99.01	99.04	98.71	98.89	98.96	98.54	98.53	98.58	98.54
**94**	98.84	99.02	98.99	98.71	98.88	98.90	98.49	98.50	98.56	98.48
**95**	99.10	99.14	99.16	98.83	99.02	99.05	98.60	98.62	98.76	98.60
**96**	99.19	99.19	99.20	98.89	99.00	99.04	98.70	98.66	98.75	98.66
**97**	99.07	99.06	99.06	98.88	98.97	99.02	98.57	98.55	98.66	98.59
**98**	99.92	99.58	99.15	98.88	99.06	99.08	98.72	98.68	98.74	98.69
**99**	99.89	99.50	99.10	98.80	99.05	99.01	98.63	98.62	98.73	98.60
**100**	99.88	99.48	99.08	98.78	98.97	99.01	98.62	98.51	98.71	98.59
**101**	100.00	99.46	99.00	98.80	98.96	98.98	98.64	98.52	98.67	98.59
**102**	99.46	100.00	99.11	98.88	98.94	99.03	98.65	98.58	98.70	98.64
**103**	99.00	99.11	100.00	98.80	98.90	98.94	98.53	98.51	98.66	98.52
**104**	98.80	98.88	98.80	100.00	98.84	98.90	98.56	98.53	98.61	98.57
**105**	98.96	98.94	98.90	98.84	100.00	99.99	98.64	98.67	98.62	98.58
**106**	98.98	99.03	98.94	98.90	99.99	100.00	98.69	98.67	98.70	98.66
**107**	98.64	98.65	98.53	98.56	98.64	98.69	100.00	99.78	99.72	99.87
**108**	98.52	98.58	98.51	98.53	98.67	98.67	99.78	100.00	99.72	99.88
**109**	98.67	98.70	98.66	98.61	98.62	98.70	99.72	99.72	100.00	99.77
**110**	98.59	98.64	98.52	98.57	98.58	98.66	99.87	99.88	99.77	100.00
**111**	98.67	98.67	98.54	98.61	98.66	98.69	99.88	99.89	99.77	99.92
**112**	98.62	98.67	98.54	98.58	98.68	98.70	99.92	99.95	99.79	99.89
**113**	98.60	98.69	98.57	98.59	98.64	98.66	99.87	99.88	99.75	99.96
**114**	98.56	98.68	98.50	98.58	98.63	98.64	99.90	99.92	99.79	99.96
**115**	98.65	98.65	98.59	98.67	98.63	98.70	99.91	99.92	99.79	99.97
**116**	98.67	98.69	98.63	98.63	98.68	98.68	99.81	99.79	99.94	99.80
**117**	98.51	98.54	98.54	98.51	98.55	98.67	98.50	98.46	98.52	98.48
**118**	98.54	98.55	98.51	98.51	98.59	98.64	98.52	98.53	98.52	98.49
**119**	97.03	97.08	97.01	97.00	97.07	97.07	96.98	96.96	97.14	96.95
**120**	96.97	97.05	96.94	96.97	97.03	97.03	97.07	97.03	97.17	97.03
**121**	96.25	96.15	96.19	96.23	96.30	96.36	96.25	96.12	96.02	96.15
**122**	96.92	96.97	96.92	96.89	96.92	97.01	96.96	97.00	97.07	96.98
**123**	97.09	97.14	97.20	97.12	97.25	97.33	97.13	97.22	97.30	97.21
**124**	97.08	97.17	97.18	97.11	97.21	97.26	97.18	97.18	97.32	97.21
**125**	97.20	97.27	97.21	97.18	97.30	97.26	97.19	97.15	97.33	97.20
**126**	97.21	97.30	97.23	97.26	97.27	97.32	97.34	97.31	97.37	97.33
**127**	97.17	97.25	97.25	97.23	97.25	97.26	97.23	97.22	97.29	97.21

**128**	96.80	96.89	96.78	96.78	96.85	96.92	96.80	96.83	96.93	96.85
**129**	96.89	97.00	96.96	96.94	97.00	97.01	97.06	97.03	97.12	96.92
**130**	97.02	97.00	96.98	96.99	96.99	97.04	96.96	96.96	97.07	97.06
**131**	96.90	96.95	96.88	96.83	96.95	96.97	96.91	96.92	96.96	97.01
**132**	96.94	96.99	96.94	96.86	96.92	96.94	96.99	96.93	97.09	96.94
**133**	97.06	97.07	97.04	97.03	97.10	97.05	97.07	97.06	97.13	97.04
**134**	96.95	96.92	96.98	96.96	96.95	97.03	96.95	97.01	97.02	97.00
**135**	96.96	96.95	96.92	96.91	96.97	96.98	96.91	97.01	97.01	96.97
**136**	96.90	96.92	96.90	96.82	96.90	96.95	96.82	96.91	96.94	96.88
**137**	96.51	96.49	96.45	96.51	96.53	96.58	96.45	96.45	96.60	96.44
**138**	96.44	96.62	96.43	96.51	96.52	96.58	96.49	96.45	96.57	96.55
**139**	96.52	96.58	96.52	96.54	96.54	96.64	96.46	96.46	96.61	96.52
**140**	96.50	96.63	96.61	96.64	96.62	96.60	96.50	96.45	96.68	96.49
**141**	96.26	96.34	96.25	96.30	96.34	96.35	96.22	96.20	96.39	96.22
**142**	96.54	96.59	96.60	96.57	96.59	96.61	96.56	96.55	96.60	96.56
**143**	96.65	96.68	96.62	96.62	96.66	96.72	96.48	96.44	96.68	96.44
**144**	95.53	95.61	95.58	95.55	95.52	95.57	95.45	95.42	95.64	95.42
**145**	95.39	95.46	95.46	95.44	95.50	95.49	95.34	95.30	95.47	95.35
**146**	95.64	95.70	95.69	95.66	95.61	95.65	95.53	95.59	95.81	95.69
**147**	96.56	96.61	96.47	96.51	96.57	96.56	96.49	96.43	96.38	96.37
**148**	91.92	91.92	91.91	91.96	91.83	91.94	91.88	91.92	92.11	91.86
**149**	91.91	91.99	91.96	92.02	91.92	91.96	91.94	91.95	92.09	91.97
**150**	91.90	91.93	91.83	92.01	91.87	91.96	91.93	91.93	92.04	91.92
**151**	91.90	91.92	91.85	92.04	91.83	91.86	91.87	91.86	92.06	91.85
**152**	91.90	91.92	91.85	92.04	91.83	91.86	91.87	91.86	92.06	91.85
**153**	91.82	91.82	91.83	91.89	91.89	91.94	91.92	91.94	91.96	91.89
**154**	91.82	91.85	91.76	91.89	91.82	91.85	91.84	91.79	92.09	91.84
**155**	91.76	91.84	91.79	91.86	91.78	91.75	91.82	91.82	91.98	91.82
**156**	91.90	91.99	91.92	92.00	91.90	91.94	91.84	91.92	92.03	91.85
**157**	91.80	91.83	91.83	91.92	91.89	91.89	91.77	91.89	92.01	91.85
**158**	92.11	92.14	92.09	92.07	92.13	92.20	92.11	92.13	92.26	92.20
**159**	94.01	94.20	94.03	94.04	94.14	94.15	94.14	94.18	94.29	94.13
**160**	93.81	93.80	93.72	93.63	93.81	93.76	93.78	93.62	93.82	93.71
**161**	93.63	93.68	93.72	93.68	93.75	93.75	93.63	93.59	93.78	93.63
**162**	83.82	83.84	83.82	83.85	83.80	83.86	83.83	83.89	83.97	83.79
**163**	83.84	83.83	83.80	83.85	83.84	83.87	83.81	83.86	83.99	83.78
**164**	83.95	83.87	83.84	83.90	83.93	84.01	83.87	83.89	83.97	83.85
**165**	83.92	83.83	83.84	83.94	83.88	83.95	83.95	83.97	84.09	83.92
**166**	83.91	83.83	83.91	83.89	83.88	83.93	83.91	83.90	84.03	83.88

Table S3. (continued)

**Isolate**	**111**	**112**	**113**	**114**	**115**	**116**	**117**	**118**	**119**	**120**
**1**	98.44	98.42	98.31	98.38	98.38	98.43	98.30	98.35	97.01	97.01
**2**	98.36	98.34	98.26	98.32	98.33	98.35	98.24	98.30	96.93	96.98
**3**	98.41	98.41	98.33	98.39	98.41	98.40	98.25	98.34	96.99	97.03
**4**	98.49	98.42	98.34	98.42	98.43	98.44	98.37	98.35	97.01	97.06
**5**	98.32	98.35	98.31	98.28	98.31	98.37	98.30	98.29	97.00	96.96
**6**	98.40	98.37	98.37	98.36	98.40	98.40	98.31	98.38	97.02	97.03
**7**	98.29	98.31	98.29	98.29	98.31	98.30	98.18	98.21	96.97	96.96
**8**	98.35	98.34	98.28	98.35	98.38	98.44	98.30	98.31	96.92	97.03
**9**	98.34	98.38	98.18	98.34	98.34	98.41	98.21	98.23	97.01	96.99
**10**	98.41	98.34	98.33	98.36	98.41	98.47	98.26	98.30	97.02	97.06
**11**	98.47	98.36	98.28	98.40	98.41	98.45	98.33	98.36	96.94	97.00
**12**	98.39	98.38	98.36	98.37	98.41	98.44	98.25	98.31	96.98	97.05
**13**	98.42	98.43	98.34	98.39	98.39	98.42	98.27	98.32	97.02	97.03
**14**	98.40	98.39	98.38	98.37	98.42	98.42	98.26	98.29	97.01	97.01
**15**	98.42	98.38	98.34	98.39	98.43	98.40	98.24	98.29	96.93	97.02
**16**	98.40	98.36	98.32	98.46	98.40	98.37	98.16	98.30	96.97	97.08
**17**	98.40	98.36	98.28	98.37	98.42	98.39	98.22	98.31	96.97	97.01
**18**	98.36	98.27	98.25	98.29	98.31	98.36	98.32	98.28	96.93	97.00
**19**	98.42	98.41	98.39	98.37	98.37	98.44	98.30	98.32	97.02	97.01
**20**	98.35	98.37	98.40	98.35	98.42	98.39	98.23	98.27	96.99	96.96
**21**	98.39	98.38	98.38	98.36	98.42	98.40	98.30	98.29	97.02	97.03
**22**	98.43	98.46	98.46	98.45	98.49	98.43	98.18	98.25	97.06	96.96
**23**	98.45	98.41	98.42	98.41	98.43	98.51	98.39	98.36	97.03	97.01
**24**	98.53	98.51	98.49	98.47	98.49	98.52	98.39	98.45	97.02	97.00
**25**	98.62	98.61	98.58	98.61	98.61	98.62	98.50	98.52	97.06	97.09
**26**	98.51	98.52	98.53	98.49	98.53	98.49	98.41	98.40	97.05	97.03
**27**	98.43	98.42	98.44	98.47	98.46	98.54	98.29	98.32	97.01	97.01
**28**	98.38	98.42	98.46	98.37	98.45	98.44	98.39	98.29	96.98	96.95
**29**	98.43	98.41	98.42	98.47	98.52	98.42	98.24	98.29	97.00	96.98
**30**	98.38	98.36	98.34	98.34	98.33	98.43	98.31	98.29	97.03	97.02
**31**	98.41	98.39	98.36	98.37	98.46	98.45	98.24	98.28	96.97	96.97
**32**	98.34	98.31	98.29	98.28	98.32	98.34	98.24	98.25	96.91	96.89
**33**	98.37	98.38	98.35	98.32	98.32	98.39	98.20	98.29	97.01	96.94
**34**	98.46	98.40	98.41	98.41	98.42	98.54	98.37	98.40	97.00	97.01
**35**	98.30	98.32	98.35	98.30	98.28	98.40	98.26	98.29	96.89	96.96
**36**	98.37	98.41	98.31	98.32	98.40	98.41	98.34	98.33	96.98	97.01
**37**	98.33	98.32	98.29	98.30	98.34	98.34	98.12	98.23	96.87	96.93
**38**	98.41	98.46	98.33	98.39	98.40	98.49	98.26	98.25	96.96	97.00
**39**	98.36	98.29	98.28	98.31	98.31	98.46	98.33	98.31	96.94	96.99
**40**	98.52	98.51	98.49	98.47	98.51	98.52	98.34	98.46	97.04	97.02
**41**	98.75	98.83	98.70	98.78	98.82	98.79	98.65	98.70	97.03	97.05

**42**	98.77	98.80	98.73	98.81	98.84	98.83	98.66	98.67	96.99	97.02
**43**	98.80	98.82	98.75	98.81	98.84	98.80	98.63	98.65	97.00	97.04
**44**	98.80	98.84	98.77	98.82	98.88	98.79	98.61	98.70	97.00	97.01
**45**	98.79	98.84	98.77	98.87	98.90	98.87	98.69	98.73	97.08	97.09
**46**	98.79	98.83	98.79	98.86	98.88	98.81	98.60	98.69	97.01	97.03
**47**	98.72	98.76	98.72	98.73	98.77	98.79	98.68	98.75	97.08	97.04
**48**	98.80	98.83	98.82	98.80	98.82	98.84	98.84	98.79	97.05	97.05
**49**	98.70	98.69	98.69	98.67	98.68	98.72	98.72	98.74	97.02	96.97
**50**	98.78	98.79	98.81	98.80	98.77	98.82	98.82	98.74	97.07	97.06
**51**	98.79	98.79	98.80	98.74	98.80	98.84	98.77	98.76	97.03	97.00
**52**	98.64	98.60	98.58	98.54	98.53	98.68	98.57	98.75	96.81	96.96
**53**	98.83	98.80	98.76	98.81	98.78	98.87	98.79	98.79	97.06	97.08
**54**	98.79	98.80	98.77	98.72	98.79	98.82	98.76	98.78	97.09	97.03
**55**	98.59	98.66	98.60	98.52	98.58	98.60	98.60	98.64	97.02	96.92
**56**	98.63	98.64	98.64	98.62	98.68	98.58	98.62	98.61	97.01	96.98
**57**	98.71	98.66	98.65	98.73	98.69	98.79	98.74	98.74	97.07	97.09
**58**	98.74	98.67	98.71	98.70	98.71	98.80	98.78	98.74	97.05	97.08
**59**	98.67	98.66	98.63	98.64	98.64	98.67	98.56	98.52	97.03	96.96
**60**	98.67	98.66	98.63	98.64	98.64	98.67	98.56	98.52	97.03	96.96
**61**	98.60	98.58	98.64	98.59	98.65	98.62	98.51	98.48	97.03	97.06
**62**	98.60	98.59	98.54	98.57	98.60	98.70	98.62	98.57	97.00	97.03
**63**	98.63	98.60	98.69	98.60	98.68	98.64	98.58	98.48	97.04	97.02
**64**	98.65	98.62	98.61	98.60	98.62	98.62	98.56	98.49	97.06	97.00
**65**	98.61	98.61	98.65	98.58	98.65	98.64	98.58	98.51	97.04	97.02
**66**	98.63	98.65	98.66	98.68	98.69	98.63	98.37	98.51	96.97	97.03
**67**	98.55	98.63	98.59	98.56	98.62	98.56	98.51	98.55	97.03	97.04
**68**	98.66	98.69	98.65	98.62	98.68	98.63	98.56	98.56	97.06	97.02
**69**	98.56	98.51	98.58	98.55	98.55	98.67	98.52	98.55	97.00	97.00
**70**	98.62	98.64	98.64	98.55	98.61	98.66	98.59	98.57	97.06	97.01
**71**	98.48	98.55	98.49	98.56	98.49	98.51	98.49	98.56	97.00	96.98
**72**	98.52	98.54	98.50	98.54	98.61	98.64	98.41	98.51	97.00	96.95
**73**	98.65	98.68	98.71	98.67	98.72	98.69	98.69	98.61	97.11	97.03
**74**	98.56	98.55	98.55	98.56	98.62	98.65	98.51	98.59	97.01	97.00
**75**	98.65	98.70	98.65	98.69	98.69	98.73	98.65	98.63	97.04	97.03
**76**	98.66	98.65	98.62	98.63	98.67	98.63	98.52	98.59	97.00	96.92
**77**	98.61	98.63	98.58	98.63	98.67	98.58	98.52	98.60	96.92	96.97
**78**	98.67	98.67	98.60	98.63	98.69	98.66	98.59	98.57	96.98	97.02
**79**	98.62	98.67	98.65	98.66	98.69	98.61	98.50	98.52	97.08	96.96
**80**	98.62	98.59	98.51	98.59	98.60	98.64	98.63	98.59	97.07	97.13
**81**	98.60	98.62	98.62	98.61	98.62	98.67	98.60	98.52	97.04	97.05
**82**	98.55	98.55	98.51	98.56	98.50	98.57	98.53	98.57	97.10	97.07
**83**	98.44	98.49	98.47	98.45	98.47	98.49	98.50	98.53	96.94	96.90
**84**	98.65	98.63	98.65	98.62	98.64	98.57	98.47	98.49	97.02	97.08

**85**	98.57	98.56	98.56	98.53	98.60	98.56	98.59	98.56	96.94	96.96
**86**	98.59	98.57	98.58	98.61	98.62	98.62	98.54	98.55	97.02	96.95
**87**	98.57	98.56	98.55	98.55	98.57	98.60	98.56	98.62	97.03	96.96
**88**	98.66	98.62	98.60	98.65	98.68	98.61	98.56	98.62	96.98	96.98
**89**	98.65	98.62	98.64	98.67	98.67	98.58	98.51	98.60	97.01	96.97
**90**	98.59	98.53	98.55	98.56	98.58	98.59	98.48	98.41	97.01	96.95
**91**	98.52	98.52	98.54	98.50	98.54	98.51	98.38	98.39	96.91	96.87
**92**	98.61	98.57	98.61	98.56	98.60	98.65	98.44	98.46	97.01	96.91
**93**	98.59	98.59	98.52	98.59	98.55	98.59	98.45	98.44	96.97	96.97
**94**	98.56	98.59	98.52	98.51	98.55	98.56	98.43	98.47	96.98	96.93
**95**	98.66	98.61	98.62	98.63	98.65	98.71	98.57	98.55	96.97	97.03
**96**	98.69	98.70	98.72	98.64	98.74	98.70	98.63	98.57	97.04	97.03
**97**	98.62	98.60	98.60	98.59	98.62	98.63	98.55	98.52	97.00	96.97
**98**	98.76	98.71	98.69	98.74	98.73	98.71	98.67	98.62	97.05	97.10
**99**	98.63	98.63	98.64	98.60	98.65	98.61	98.56	98.50	97.00	96.96
**100**	98.63	98.60	98.61	98.61	98.62	98.64	98.53	98.51	97.06	97.02
**101**	98.67	98.62	98.60	98.56	98.65	98.67	98.51	98.54	97.03	96.97
**102**	98.67	98.67	98.69	98.68	98.65	98.69	98.54	98.55	97.08	97.05
**103**	98.54	98.54	98.57	98.50	98.59	98.63	98.54	98.51	97.01	96.94
**104**	98.61	98.58	98.59	98.58	98.67	98.63	98.51	98.51	97.00	96.97
**105**	98.66	98.68	98.64	98.63	98.63	98.68	98.55	98.59	97.07	97.03
**106**	98.69	98.70	98.66	98.64	98.70	98.68	98.67	98.64	97.07	97.03
**107**	99.88	99.92	99.87	99.90	99.91	99.81	98.50	98.52	96.98	97.07
**108**	99.89	99.95	99.88	99.92	99.92	99.79	98.46	98.53	96.96	97.03
**109**	99.77	99.79	99.75	99.79	99.79	99.94	98.52	98.52	97.14	97.17
**110**	99.92	99.89	99.96	99.96	99.97	99.80	98.48	98.49	96.95	97.03
**111**	100.00	99.95	99.92	99.88	99.94	99.84	98.56	98.52	97.01	97.09
**112**	99.95	100.00	99.89	99.95	99.96	99.83	98.52	98.56	96.99	97.06
**113**	99.92	99.89	100.00	99.96	99.94	99.81	98.48	98.55	96.89	97.08
**114**	99.88	99.95	99.96	100.00	99.95	99.79	98.50	98.54	97.00	97.04
**115**	99.94	99.96	99.94	99.95	100.00	99.83	98.47	98.54	96.97	97.06
**116**	99.84	99.83	99.81	99.79	99.83	100.00	98.58	98.59	97.05	97.10
**117**	98.56	98.52	98.48	98.50	98.47	98.58	100.00	99.03	97.03	97.01
**118**	98.52	98.56	98.55	98.54	98.54	98.59	99.03	100.00	97.13	97.11
**119**	97.01	96.99	96.89	97.00	96.97	97.05	97.03	97.13	100.00	98.46
**120**	97.09	97.06	97.08	97.04	97.06	97.10	97.01	97.11	98.46	100.00
**121**	96.19	96.20	96.01	96.26	96.31	96.25	96.22	96.12	97.02	97.26
**122**	97.08	97.02	97.00	96.98	97.01	97.02	96.93	97.05	98.41	98.32
**123**	97.26	97.26	97.24	97.28	97.23	97.17	97.17	97.18	97.04	97.02
**124**	97.26	97.28	97.24	97.27	97.24	97.19	97.16	97.20	97.02	96.98
**125**	97.17	97.19	97.19	97.17	97.17	97.23	97.20	97.26	97.07	97.04
**126**	97.31	97.34	97.33	97.34	97.34	97.35	97.29	97.31	97.06	97.06
**127**	97.24	97.24	97.23	97.22	97.23	97.22	97.24	97.17	96.92	96.92

**128**	96.92	96.86	96.81	96.85	96.83	96.90	96.84	96.85	96.41	96.36
**129**	97.05	97.07	97.04	96.91	97.01	97.04	96.98	96.96	96.49	96.50
**130**	96.97	97.01	97.02	97.07	97.04	96.95	96.91	96.98	96.51	96.47
**131**	96.97	96.98	96.87	97.12	97.05	96.95	96.83	96.84	96.41	96.45
**132**	96.99	97.00	97.00	96.97	96.97	96.98	96.90	96.91	96.47	96.48
**133**	97.08	97.10	97.07	97.06	97.08	97.04	97.04	96.97	96.56	96.52
**134**	97.04	97.01	96.99	96.99	97.02	96.97	96.89	96.94	96.49	96.56
**135**	96.99	97.01	96.99	96.99	97.02	96.99	96.89	96.89	96.47	96.46
**136**	96.92	96.93	96.93	96.89	96.95	96.95	96.86	96.84	96.42	96.41
**137**	96.49	96.43	96.42	96.46	96.43	96.45	96.48	96.46	96.45	96.36
**138**	96.56	96.52	96.54	96.55	96.51	96.56	96.52	96.53	96.53	96.52
**139**	96.58	96.54	96.53	96.56	96.56	96.49	96.50	96.55	96.47	96.50
**140**	96.50	96.49	96.49	96.50	96.51	96.50	96.54	96.58	96.50	96.50
**141**	96.26	96.30	96.25	96.23	96.26	96.32	96.33	96.38	96.18	96.15
**142**	96.60	96.58	96.56	96.54	96.57	96.51	96.48	96.59	96.50	96.49
**143**	96.49	96.53	96.47	96.46	96.51	96.60	96.61	96.63	96.64	96.58
**144**	95.41	95.42	95.38	95.48	95.42	95.49	95.58	95.59	95.51	95.52
**145**	95.29	95.32	95.29	95.30	95.26	95.35	95.37	95.46	95.42	95.39
**146**	95.62	95.66	95.61	95.75	95.75	95.70	95.62	95.70	95.62	95.63
**147**	96.41	96.43	96.51	96.48	96.52	96.43	96.41	96.46	96.34	96.23
**148**	91.93	91.90	91.88	91.89	91.86	91.89	91.93	91.89	91.80	91.79
**149**	92.00	92.00	91.96	91.97	92.01	91.97	91.95	91.90	91.88	91.83
**150**	91.90	91.90	91.91	91.92	91.88	91.86	91.90	91.94	91.85	91.78
**151**	91.85	91.86	91.85	91.88	91.87	91.89	91.99	91.88	91.79	91.77
**152**	91.85	91.86	91.85	91.88	91.87	91.89	91.99	91.88	91.79	91.77
**153**	91.91	91.93	91.91	91.91	91.91	91.80	91.83	91.87	91.74	91.71
**154**	91.78	91.87	91.80	91.77	91.81	91.80	91.86	91.74	91.71	91.69
**155**	91.82	91.85	91.81	91.80	91.81	91.80	91.79	91.86	91.74	91.73
**156**	91.90	91.86	91.86	91.86	91.85	91.87	91.88	91.94	91.82	91.89
**157**	91.86	91.81	91.79	91.79	91.79	91.81	91.80	91.82	91.73	91.74
**158**	92.16	92.21	92.14	92.16	92.17	92.11	92.13	92.10	92.17	92.12
**159**	94.21	94.21	94.14	94.14	94.19	94.26	94.05	94.16	94.05	94.05
**160**	93.66	93.71	93.71	93.80	93.73	93.68	93.75	93.86	93.72	93.78
**161**	93.66	93.65	93.62	93.64	93.64	93.62	93.65	93.69	93.59	93.61
**162**	83.88	83.81	83.83	83.81	83.76	83.82	83.80	83.77	83.66	83.76
**163**	83.86	83.81	83.80	83.82	83.78	83.81	83.80	83.79	83.70	83.75
**164**	83.86	83.89	83.91	83.87	83.85	83.85	83.90	83.85	83.78	83.72
**165**	83.97	83.91	83.94	83.83	83.88	83.89	83.86	83.87	83.85	83.74
**166**	83.93	83.91	83.93	83.89	83.93	83.90	83.89	83.90	83.76	83.72

Table S3. (continued)

Isolate	121	122	123	124	125	126	127	128	129	130
**1**	96.29	96.99	97.11	97.15	97.16	97.25	97.22	96.82	97.01	96.97
**2**	96.18	96.93	97.14	97.14	97.19	97.21	97.15	96.80	96.97	96.93
**3**	96.09	96.94	97.15	97.09	97.22	97.20	97.13	96.82	97.06	96.96
**4**	96.36	97.05	97.14	97.16	97.19	97.29	97.25	96.89	97.06	96.99
**5**	96.10	96.95	97.15	97.12	97.18	97.19	97.21	96.73	96.97	97.00
**6**	96.17	96.94	97.21	97.23	97.28	97.29	97.28	96.92	97.03	97.02
**7**	95.83	96.98	97.11	97.12	97.14	97.19	97.22	96.80	96.98	96.98
**8**	95.99	96.92	97.18	97.15	97.19	97.27	97.25	96.80	97.03	96.98
**9**	95.98	96.97	97.18	97.14	97.18	97.23	97.22	96.78	96.93	97.07
**10**	96.32	96.97	97.15	97.23	97.24	97.27	97.28	96.84	97.12	97.04
**11**	96.24	96.96	97.21	97.16	97.21	97.26	97.23	96.87	97.05	97.02
**12**	96.21	96.96	97.08	97.13	97.21	97.26	97.20	96.86	96.99	97.06
**13**	96.33	97.03	97.16	97.20	97.19	97.28	97.24	96.90	97.05	96.96
**14**	96.34	96.92	97.18	97.14	97.16	97.22	97.20	96.77	97.02	96.97
**15**	96.27	96.94	97.15	97.17	97.21	97.26	97.20	96.82	96.98	97.11
**16**	95.95	96.95	97.10	97.13	97.25	97.27	97.26	96.84	96.99	97.05
**17**	96.14	96.98	97.12	97.20	97.19	97.30	97.21	96.76	96.99	97.04
**18**	96.03	97.00	97.19	97.17	97.19	97.27	97.25	96.77	96.97	96.99
**19**	96.36	96.98	97.17	97.17	97.21	97.25	97.28	96.86	97.01	97.08
**20**	96.15	96.82	97.17	97.17	97.17	97.19	97.21	96.91	97.10	97.15
**21**	96.07	96.90	97.13	97.20	97.19	97.19	97.21	96.91	97.15	97.15
**22**	96.15	96.88	97.13	97.18	97.21	97.25	97.21	96.91	97.16	97.13
**23**	96.31	97.04	97.20	97.23	97.21	97.32	97.24	96.96	97.03	97.04
**24**	96.12	96.95	97.26	97.29	97.26	97.26	97.28	97.00	97.09	97.03
**25**	96.42	97.03	97.36	97.39	97.38	97.35	97.32	97.07	97.15	97.13
**26**	96.14	96.95	97.19	97.20	97.27	97.27	97.23	96.98	97.05	97.03
**27**	95.98	96.89	97.15	97.19	97.25	97.26	97.24	96.85	97.03	97.00
**28**	96.07	97.00	97.19	97.21	97.20	97.27	97.23	96.90	97.06	96.98
**29**	96.14	96.90	97.14	97.17	97.20	97.26	97.22	96.79	96.94	97.07
**30**	96.21	96.95	97.15	97.11	97.17	97.21	97.22	96.80	96.97	97.01
**31**	96.29	96.88	97.10	97.10	97.18	97.23	97.24	96.74	97.05	96.98
**32**	96.08	96.87	97.14	97.16	97.17	97.18	97.18	96.79	96.95	96.93
**33**	96.11	96.85	97.14	97.20	97.17	97.21	97.21	96.83	96.99	96.91
**34**	96.28	96.97	97.25	97.26	97.27	97.30	97.27	96.95	97.08	97.01
**35**	96.10	96.89	97.09	97.16	97.10	97.23	97.16	96.76	97.00	96.94
**36**	96.12	96.94	97.22	97.16	97.19	97.28	97.20	96.89	96.99	96.95
**37**	96.15	96.84	97.09	97.10	97.14	97.17	97.13	96.72	96.93	96.98
**38**	96.16	96.91	97.04	97.12	97.27	97.25	97.23	96.80	97.01	96.94
**39**	96.01	96.88	97.14	97.20	97.15	97.28	97.18	96.87	96.94	96.94
**40**	96.36	96.95	97.14	97.14	97.22	97.26	97.29	96.81	97.01	97.02
**41**	96.28	96.94	97.16	97.16	97.23	97.30	97.29	96.89	97.02	96.99

**42**	96.15	96.96	97.16	97.14	97.23	97.27	97.25	96.83	97.02	97.02
**43**	96.20	96.91	97.13	97.14	97.22	97.31	97.29	96.87	97.04	96.97
**44**	96.15	96.96	97.12	97.15	97.21	97.24	97.25	96.89	96.97	97.01
**45**	96.19	97.08	97.30	97.30	97.30	97.32	97.32	97.01	96.98	97.15
**46**	96.20	96.96	97.14	97.15	97.20	97.31	97.27	96.91	97.04	96.98
**47**	96.33	96.94	97.19	97.24	97.25	97.29	97.19	96.90	96.93	96.97
**48**	96.29	97.03	97.32	97.35	97.37	97.33	97.26	96.93	96.97	96.99
**49**	96.13	96.86	97.19	97.19	97.24	97.27	97.30	96.77	96.89	96.91
**50**	96.24	96.98	97.28	97.31	97.28	97.36	97.23	96.93	97.01	97.00
**51**	96.16	96.89	97.24	97.25	97.26	97.25	97.22	96.87	96.99	97.02
**52**	95.92	96.84	97.15	97.13	97.19	97.21	97.16	96.56	96.86	96.76
**53**	96.30	97.09	97.35	97.39	97.34	97.38	97.30	97.09	97.07	97.06
**54**	96.12	97.03	97.23	97.27	97.30	97.33	97.27	96.97	97.05	96.99
**55**	95.99	96.88	97.18	97.19	97.18	97.26	97.19	96.78	96.96	96.79
**56**	95.82	96.91	97.15	97.19	97.14	97.25	97.16	96.77	96.95	96.90
**57**	96.10	97.05	97.34	97.40	97.37	97.41	97.31	96.97	97.09	97.09
**58**	96.34	97.03	97.33	97.37	97.35	97.37	97.26	96.95	97.04	97.06
**59**	96.41	96.94	97.21	97.22	97.18	97.28	97.16	96.83	96.92	96.98
**60**	96.41	96.94	97.21	97.22	97.18	97.28	97.16	96.83	96.92	96.98
**61**	96.28	96.91	97.18	97.17	97.21	97.23	97.23	96.79	96.86	97.01
**62**	96.22	96.94	97.15	97.14	97.25	97.24	97.23	96.83	96.99	97.03
**63**	96.26	96.93	97.22	97.22	97.23	97.28	97.23	96.79	96.97	96.96
**64**	96.29	96.91	97.18	97.16	97.25	97.26	97.22	96.79	96.94	97.02
**65**	96.21	96.90	97.23	97.20	97.22	97.27	97.24	96.75	96.92	96.93
**66**	96.38	96.87	97.17	97.16	97.22	97.25	97.21	96.71	96.92	97.05
**67**	96.28	96.85	97.20	97.17	97.13	97.24	97.19	96.73	96.89	96.96
**68**	96.20	96.97	97.19	97.18	97.16	97.25	97.19	96.84	96.99	96.94
**69**	96.32	96.81	97.24	97.21	97.15	97.21	97.18	96.80	96.86	96.89
**70**	96.36	96.92	97.21	97.23	97.20	97.28	97.22	96.88	96.91	96.89
**71**	96.05	96.91	97.16	97.16	97.22	97.27	97.16	96.71	96.98	96.96
**72**	96.18	96.79	97.16	97.16	97.19	97.20	97.18	96.65	96.84	97.00
**73**	96.22	96.98	97.23	97.26	97.20	97.27	97.27	96.90	96.94	96.94
**74**	96.21	96.78	97.14	97.21	97.17	97.26	97.16	96.71	96.91	96.89
**75**	96.05	97.04	97.23	97.23	97.26	97.34	97.26	96.90	97.03	97.04
**76**	96.02	96.84	97.15	97.17	97.20	97.27	97.19	96.80	96.91	96.98
**77**	95.91	96.85	97.17	97.17	97.18	97.25	97.14	96.72	96.96	96.96
**78**	96.26	97.01	97.13	97.15	97.23	97.34	97.29	96.90	97.02	97.01
**79**	96.27	96.93	97.13	97.16	97.22	97.26	97.16	96.87	96.95	97.04
**80**	96.22	97.09	97.26	97.26	97.35	97.30	97.28	96.99	97.05	97.03
**81**	96.37	96.93	97.16	97.21	97.27	97.31	97.30	96.83	96.98	96.97
**82**	96.29	97.02	97.14	97.18	97.20	97.29	97.20	96.77	96.98	96.92
**83**	95.79	96.86	97.14	97.12	97.16	97.18	97.22	96.79	96.91	96.91
**84**	96.12	96.86	97.17	97.14	97.18	97.30	97.22	96.85	97.02	97.02

**85**	96.05	96.80	97.19	97.13	97.22	97.28	97.19	96.81	96.99	96.96
**86**	96.13	96.97	97.17	97.21	97.20	97.31	97.28	96.82	96.97	97.04
**87**	96.14	96.92	97.18	97.19	97.11	97.28	97.24	96.80	96.87	96.88
**88**	96.14	96.95	97.19	97.22	97.23	97.27	97.15	96.82	97.02	96.96
**89**	96.24	96.87	97.14	97.16	97.19	97.29	97.16	96.79	96.95	96.93
**90**	96.16	96.90	97.19	97.25	97.24	97.26	97.22	96.96	97.05	97.05
**91**	96.10	96.85	97.16	97.16	97.13	97.20	97.14	96.87	96.98	97.02
**92**	96.02	96.95	97.16	97.18	97.21	97.25	97.19	96.99	97.04	97.08
**93**	96.18	96.89	97.17	97.14	97.21	97.31	97.19	96.93	97.10	97.08
**94**	96.31	96.88	97.18	97.16	97.15	97.28	97.24	96.85	97.05	97.05
**95**	96.02	96.93	97.22	97.19	97.22	97.26	97.26	96.84	96.95	96.98
**96**	96.27	96.99	97.24	97.21	97.20	97.33	97.25	96.87	96.91	96.96
**97**	96.27	96.86	97.22	97.19	97.25	97.25	97.24	96.77	96.93	96.96
**98**	96.37	97.05	97.26	97.26	97.33	97.35	97.26	96.96	97.07	97.08
**99**	96.26	97.02	97.25	97.21	97.21	97.24	97.18	96.91	96.94	97.06
**100**	96.28	96.97	97.17	97.15	97.28	97.27	97.23	96.82	96.97	97.01
**101**	96.25	96.92	97.09	97.08	97.20	97.21	97.17	96.80	96.89	97.02
**102**	96.15	96.97	97.14	97.17	97.27	97.30	97.25	96.89	97.00	97.00
**103**	96.19	96.92	97.20	97.18	97.21	97.23	97.25	96.78	96.96	96.98
**104**	96.23	96.89	97.12	97.11	97.18	97.26	97.23	96.78	96.94	96.99
**105**	96.30	96.92	97.25	97.21	97.30	97.27	97.25	96.85	97.00	96.99
**106**	96.36	97.01	97.33	97.26	97.26	97.32	97.26	96.92	97.01	97.04
**107**	96.25	96.96	97.13	97.18	97.19	97.34	97.23	96.80	97.06	96.96
**108**	96.12	97.00	97.22	97.18	97.15	97.31	97.22	96.83	97.03	96.96
**109**	96.02	97.07	97.30	97.32	97.33	97.37	97.29	96.93	97.12	97.07
**110**	96.15	96.98	97.21	97.21	97.20	97.33	97.21	96.85	96.92	97.06
**111**	96.19	97.08	97.26	97.26	97.17	97.31	97.24	96.92	97.05	96.97
**112**	96.20	97.02	97.26	97.28	97.19	97.34	97.24	96.86	97.07	97.01
**113**	96.01	97.00	97.24	97.24	97.19	97.33	97.23	96.81	97.04	97.02
**114**	96.26	96.98	97.28	97.27	97.17	97.34	97.22	96.85	96.91	97.07
**115**	96.31	97.01	97.23	97.24	97.17	97.34	97.23	96.83	97.01	97.04
**116**	96.25	97.02	97.17	97.19	97.23	97.35	97.22	96.90	97.04	96.95
**117**	96.22	96.93	97.17	97.16	97.20	97.29	97.24	96.84	96.98	96.91
**118**	96.12	97.05	97.18	97.20	97.26	97.31	97.17	96.85	96.96	96.98
**119**	97.02	98.41	97.04	97.02	97.07	97.06	96.92	96.41	96.49	96.51
**120**	97.26	98.32	97.02	96.98	97.04	97.06	96.92	96.36	96.50	96.47
**121**	100.00	97.32	95.97	96.00	95.94	96.27	96.08	95.38	95.49	95.40
**122**	97.32	100.00	96.92	96.88	96.94	96.99	96.88	96.32	96.47	96.41
**123**	95.97	96.92	100.00	99.99	99.05	98.33	97.39	96.91	97.01	97.03
**124**	96.00	96.88	99.99	100.00	99.03	98.29	97.37	96.94	96.99	97.05
**125**	95.94	96.94	99.05	99.03	100.00	98.29	97.45	96.90	96.91	97.01
**126**	96.27	96.99	98.33	98.29	98.29	100.00	97.49	96.83	96.86	96.92
**127**	96.08	96.88	97.39	97.37	97.45	97.49	100.00	96.82	96.88	96.85

**128**	95.38	96.32	96.91	96.94	96.90	96.83	96.82	100.00	99.29	99.19
**129**	95.49	96.47	97.01	96.99	96.91	96.86	96.88	99.29	100.00	99.31
**130**	95.40	96.41	97.03	97.05	97.01	96.92	96.85	99.19	99.31	100.00
**131**	95.61	96.39	96.90	96.94	96.87	96.90	96.81	99.16	99.30	99.38
**132**	95.48	96.40	96.98	96.99	97.01	96.89	96.88	99.01	99.40	99.41
**133**	95.57	96.57	97.05	97.07	97.00	96.90	96.90	99.37	99.48	99.49
**134**	95.48	96.45	96.97	97.00	97.01	96.89	96.86	99.03	99.45	99.46
**135**	95.26	96.38	96.97	96.97	96.99	96.92	96.87	99.07	99.45	99.44
**136**	95.43	96.44	96.89	96.88	96.96	96.84	96.82	98.99	99.39	99.47
**137**	95.39	96.27	96.69	96.64	96.64	96.66	96.70	96.23	96.35	96.36
**138**	95.54	96.43	96.63	96.60	96.64	96.71	96.76	96.22	96.43	96.45
**139**	95.46	96.32	96.68	96.68	96.71	96.68	96.76	96.30	96.45	96.41
**140**	95.27	96.47	96.75	96.73	96.73	96.72	96.78	96.41	96.58	96.58
**141**	95.26	96.15	96.32	96.36	96.39	96.38	96.38	96.06	96.16	96.23
**142**	95.30	96.41	96.69	96.63	96.65	96.64	96.78	96.36	96.51	96.55
**143**	95.78	96.45	96.69	96.74	96.81	96.76	96.86	96.34	96.49	96.47
**144**	95.75	95.55	95.51	95.48	95.54	95.54	95.57	95.36	95.47	95.44
**145**	95.44	95.31	95.39	95.41	95.36	95.52	95.42	95.15	95.27	95.41
**146**	95.78	95.58	95.73	95.68	95.67	95.68	95.73	95.44	95.57	95.77
**147**	96.26	96.14	96.22	96.21	96.21	96.39	96.08	95.88	95.79	95.99
**148**	90.84	91.81	91.89	91.97	91.87	91.86	91.90	91.69	91.84	91.87
**149**	90.90	91.87	91.95	91.90	91.93	91.88	91.95	91.75	91.82	91.95
**150**	90.82	91.77	91.90	91.93	91.87	91.90	91.82	91.87	91.81	91.83
**151**	90.45	91.85	91.88	91.87	91.89	91.86	91.88	91.72	91.83	91.78
**152**	90.45	91.85	91.88	91.87	91.89	91.86	91.88	91.72	91.83	91.78
**153**	90.53	91.78	91.82	91.74	91.76	91.81	91.80	91.63	91.77	91.88
**154**	90.89	91.79	91.77	91.84	91.81	91.81	91.85	91.67	91.74	91.73
**155**	91.04	91.70	91.78	91.80	91.80	91.82	91.86	91.65	91.77	91.81
**156**	90.83	91.87	91.88	91.86	91.84	91.85	91.90	91.76	91.82	91.90
**157**	90.61	91.73	91.96	91.86	91.79	91.79	91.85	91.69	91.72	91.83
**158**	90.92	92.09	92.17	92.17	92.19	92.15	92.16	92.04	92.12	92.08
**159**	92.91	94.09	94.01	93.96	94.11	94.14	94.18	93.91	94.17	94.07
**160**	91.97	93.61	93.82	93.71	93.80	93.67	93.82	93.50	93.76	93.77
**161**	92.41	93.60	93.57	93.67	93.66	93.54	93.71	93.44	93.63	93.62
**162**	83.07	83.70	83.76	83.72	83.78	83.79	83.83	83.74	83.79	83.77
**163**	83.13	83.77	83.81	83.76	83.76	83.76	83.83	83.82	83.82	83.78
**164**	83.20	83.80	83.77	83.77	83.78	83.85	83.81	83.78	83.82	83.72
**165**	83.44	83.73	83.84	83.82	83.79	83.79	83.85	83.81	83.83	83.83
**166**	83.20	83.81	83.81	83.79	83.79	83.77	83.79	83.85	83.85	83.78

Table S3. (continued)

**Isolate**	**131**	**132**	**133**	**134**	**135**	**136**	**137**	**138**	**139**	**140**
**1**	96.85	96.90	97.07	96.89	96.93	96.85	96.38	96.50	96.49	96.56
**2**	96.85	96.90	97.04	96.87	96.86	96.81	96.43	96.44	96.44	96.47
**3**	96.89	96.92	97.05	96.99	96.95	96.89	96.42	96.49	96.53	96.51
**4**	96.91	96.95	97.10	97.00	97.00	96.93	96.43	96.61	96.50	96.52
**5**	96.83	96.88	97.07	96.86	96.88	96.79	96.46	96.49	96.46	96.61
**6**	96.88	96.96	97.12	97.01	97.01	96.99	96.51	96.53	96.56	96.58
**7**	96.92	96.88	97.05	96.79	96.81	96.73	96.43	96.47	96.51	96.52
**8**	96.86	96.87	97.05	96.94	96.91	96.85	96.43	96.52	96.49	96.54
**9**	96.90	96.89	97.11	96.96	96.91	96.85	96.34	96.41	96.46	96.51
**10**	96.91	96.93	97.14	97.00	96.94	96.88	96.45	96.47	96.50	96.53
**11**	96.91	96.97	97.06	96.99	96.98	96.92	96.49	96.58	96.51	96.55
**12**	96.97	96.93	97.11	96.95	96.96	96.82	96.43	96.51	96.51	96.54
**13**	96.93	96.97	97.10	96.99	96.99	96.92	96.43	96.56	96.53	96.56
**14**	96.93	96.90	97.08	96.93	96.91	96.84	96.50	96.52	96.49	96.55
**15**	96.92	96.88	97.10	96.89	96.92	96.82	96.44	96.52	96.51	96.52
**16**	96.94	96.84	97.06	96.93	96.95	96.92	96.46	96.53	96.46	96.53
**17**	96.95	96.92	97.09	96.95	96.93	96.89	96.39	96.52	96.48	96.51
**18**	96.83	96.92	97.11	96.96	96.94	96.86	96.47	96.46	96.54	96.56
**19**	96.88	96.98	97.10	96.98	96.96	96.93	96.47	96.53	96.52	96.60
**20**	97.00	97.03	97.17	97.07	97.08	96.98	96.49	96.40	96.45	96.51
**21**	96.98	97.04	97.17	97.10	97.11	97.01	96.42	96.44	96.47	96.52
**22**	97.08	97.08	97.15	97.10	97.12	97.05	96.42	96.51	96.49	96.55
**23**	96.95	97.03	97.11	97.05	97.04	96.95	96.48	96.53	96.57	96.59
**24**	97.07	97.00	97.14	97.08	97.08	96.99	96.59	96.60	96.61	96.68
**25**	97.16	97.11	97.18	97.16	97.14	97.08	96.65	96.62	96.65	96.66
**26**	97.02	97.04	97.08	97.07	97.07	96.95	96.53	96.57	96.65	96.61
**27**	96.94	97.01	97.14	96.95	96.95	96.90	96.46	96.48	96.51	96.56
**28**	97.02	97.01	97.13	97.02	96.99	96.93	96.51	96.55	96.57	96.53
**29**	96.97	96.95	97.08	96.92	96.87	96.82	96.37	96.48	96.50	96.54
**30**	96.83	96.95	97.08	96.95	96.91	96.84	96.48	96.43	96.52	96.56
**31**	96.89	96.92	97.07	96.87	96.91	96.77	96.40	96.47	96.49	96.48
**32**	96.86	96.93	97.09	96.86	96.88	96.77	96.42	96.45	96.51	96.49
**33**	96.91	96.94	97.07	96.88	96.91	96.83	96.43	96.44	96.53	96.46
**34**	97.00	97.08	97.13	97.03	97.01	96.95	96.52	96.56	96.57	96.56
**35**	96.84	96.88	97.07	96.91	96.89	96.76	96.34	96.49	96.49	96.45
**36**	96.94	96.89	97.04	96.98	96.96	96.93	96.47	96.46	96.56	96.50
**37**	96.75	96.84	97.02	96.84	96.81	96.77	96.35	96.42	96.47	96.53
**38**	96.83	96.92	97.07	96.90	96.91	96.82	96.44	96.48	96.50	96.49
**39**	96.86	96.90	97.09	96.92	96.91	96.86	96.49	96.47	96.55	96.53
**40**	96.94	96.93	97.08	96.99	96.96	96.92	96.41	96.53	96.54	96.57
**41**	96.90	96.96	97.11	97.05	97.05	96.94	96.47	96.47	96.45	96.54

**42**	96.91	96.93	97.10	97.01	96.99	96.89	96.39	96.47	96.50	96.50
**43**	96.95	96.96	97.06	96.97	96.98	96.93	96.41	96.54	96.48	96.54
**44**	96.97	96.99	97.09	97.05	97.01	96.91	96.41	96.52	96.48	96.51
**45**	97.10	97.05	97.14	97.09	97.07	97.03	96.50	96.59	96.59	96.60
**46**	96.91	96.99	97.08	97.03	97.02	96.95	96.40	96.49	96.52	96.54
**47**	96.87	96.88	97.01	96.98	96.94	96.87	96.45	96.47	96.49	96.49
**48**	96.97	97.01	97.06	97.04	97.03	96.95	96.57	96.52	96.59	96.58
**49**	96.84	96.89	97.08	96.88	96.89	96.84	96.42	96.49	96.52	96.56
**50**	96.98	97.04	97.08	97.02	97.00	96.94	96.58	96.55	96.60	96.62
**51**	96.91	96.91	97.09	97.00	96.98	96.93	96.51	96.45	96.52	96.53
**52**	96.66	96.76	96.94	96.79	96.83	96.79	96.42	96.34	96.47	96.45
**53**	97.07	97.04	97.10	97.10	97.11	97.05	96.62	96.69	96.68	96.66
**54**	97.06	97.02	97.07	97.03	97.04	96.96	96.56	96.67	96.66	96.60
**55**	96.75	96.85	96.97	96.91	96.87	96.80	96.43	96.45	96.52	96.53
**56**	96.82	96.89	96.98	96.90	96.86	96.77	96.35	96.49	96.50	96.58
**57**	97.03	97.01	97.11	97.03	97.03	96.95	96.58	96.62	96.67	96.64
**58**	97.00	97.00	97.07	97.02	97.00	96.95	96.56	96.60	96.68	96.58
**59**	96.83	96.96	97.05	96.91	96.89	96.83	96.49	96.44	96.52	96.61
**60**	96.83	96.96	97.05	96.91	96.89	96.83	96.49	96.44	96.52	96.61
**61**	96.95	96.92	97.06	96.88	96.90	96.80	96.41	96.37	96.51	96.50
**62**	96.88	96.95	97.00	96.91	96.94	96.83	96.44	96.47	96.49	96.48
**63**	96.88	96.93	97.12	96.94	96.90	96.85	96.48	96.50	96.50	96.58
**64**	96.86	96.94	97.03	96.97	96.93	96.82	96.50	96.44	96.50	96.56
**65**	96.87	96.88	97.08	96.87	96.84	96.78	96.46	96.45	96.45	96.51
**66**	96.93	96.88	97.00	96.93	96.92	96.85	96.44	96.43	96.48	96.52
**67**	96.75	96.83	97.01	96.84	96.84	96.83	96.47	96.35	96.49	96.47
**68**	96.93	96.96	97.04	96.96	96.94	96.86	96.40	96.51	96.51	96.60
**69**	96.87	96.90	97.00	96.89	96.89	96.86	96.44	96.41	96.53	96.53
**70**	96.86	96.91	97.02	96.90	96.91	96.88	96.36	96.41	96.57	96.54
**71**	96.78	96.88	97.00	96.86	96.91	96.82	96.33	96.40	96.47	96.46
**72**	96.87	96.81	97.02	96.86	96.83	96.74	96.38	96.36	96.47	96.46
**73**	96.94	96.94	97.08	96.98	96.96	96.89	96.42	96.46	96.58	96.59
**74**	96.82	96.83	96.99	96.84	96.80	96.77	96.35	96.37	96.45	96.54
**75**	96.92	97.05	97.08	97.00	97.02	96.95	96.47	96.61	96.56	96.57
**76**	96.88	96.93	96.99	96.92	96.90	96.79	96.44	96.46	96.46	96.53
**77**	96.84	96.82	96.97	96.89	96.88	96.78	96.38	96.47	96.43	96.56
**78**	96.96	97.00	97.10	97.03	97.00	96.92	96.51	96.65	96.57	96.56
**79**	96.95	96.95	96.99	96.86	96.86	96.81	96.49	96.44	96.55	96.57
**80**	96.99	97.03	97.08	97.07	97.06	97.00	96.58	96.60	96.64	96.63
**81**	96.89	96.98	97.08	96.97	96.96	96.86	96.49	96.56	96.57	96.58
**82**	96.81	96.85	96.98	96.94	96.93	96.85	96.42	96.51	96.51	96.54
**83**	96.80	96.84	96.97	96.90	96.88	96.80	96.34	96.35	96.55	96.44
**84**	96.90	96.92	97.02	96.92	96.87	96.78	96.42	96.45	96.49	96.57

**85**	96.94	96.92	97.02	96.90	96.89	96.82	96.50	96.50	96.56	96.60
**86**	96.88	96.92	97.07	96.92	96.91	96.82	96.39	96.41	96.52	96.48
**87**	96.89	96.89	97.05	96.94	96.89	96.83	96.39	96.44	96.53	96.58
**88**	96.91	96.91	97.01	96.93	96.94	96.89	96.48	96.52	96.56	96.55
**89**	96.89	96.89	96.96	96.86	96.87	96.78	96.49	96.48	96.56	96.57
**90**	96.99	97.02	97.22	97.03	97.05	96.99	96.49	96.49	96.57	96.61
**91**	96.90	96.95	97.08	96.95	96.94	96.91	96.48	96.42	96.53	96.52
**92**	96.98	97.05	97.17	97.03	97.03	96.93	96.54	96.45	96.59	96.60
**93**	96.95	97.03	97.17	97.07	97.07	96.98	96.48	96.54	96.54	96.58
**94**	96.93	96.93	97.16	97.01	96.98	96.92	96.49	96.52	96.46	96.56
**95**	96.89	96.91	97.05	96.92	96.88	96.87	96.56	96.57	96.58	96.58
**96**	96.93	96.94	97.06	96.93	96.95	96.86	96.50	96.51	96.63	96.63
**97**	96.87	96.92	97.06	96.93	96.94	96.82	96.49	96.51	96.58	96.59
**98**	97.00	97.04	97.08	97.03	97.03	96.97	96.59	96.68	96.66	96.62
**99**	96.92	97.00	97.09	97.02	96.99	96.92	96.57	96.52	96.61	96.54
**100**	96.86	96.99	97.10	96.95	96.95	96.86	96.46	96.46	96.53	96.55
**101**	96.90	96.94	97.06	96.95	96.96	96.90	96.51	96.44	96.52	96.50
**102**	96.95	96.99	97.07	96.92	96.95	96.92	96.49	96.62	96.58	96.63
**103**	96.88	96.94	97.04	96.98	96.92	96.90	96.45	96.43	96.52	96.61
**104**	96.83	96.86	97.03	96.96	96.91	96.82	96.51	96.51	96.54	96.64
**105**	96.95	96.92	97.10	96.95	96.97	96.90	96.53	96.52	96.54	96.62
**106**	96.97	96.94	97.05	97.03	96.98	96.95	96.58	96.58	96.64	96.60
**107**	96.91	96.99	97.07	96.95	96.91	96.82	96.45	96.49	96.46	96.50
**108**	96.92	96.93	97.06	97.01	97.01	96.91	96.45	96.45	96.46	96.45
**109**	96.96	97.09	97.13	97.02	97.01	96.94	96.60	96.57	96.61	96.68
**110**	97.01	96.94	97.04	97.00	96.97	96.88	96.44	96.55	96.52	96.49
**111**	96.97	96.99	97.08	97.04	96.99	96.92	96.49	96.56	96.58	96.50
**112**	96.98	97.00	97.10	97.01	97.01	96.93	96.43	96.52	96.54	96.49
**113**	96.87	97.00	97.07	96.99	96.99	96.93	96.42	96.54	96.53	96.49
**114**	97.12	96.97	97.06	96.99	96.99	96.89	96.46	96.55	96.56	96.50
**115**	97.05	96.97	97.08	97.02	97.02	96.95	96.43	96.51	96.56	96.51
**116**	96.95	96.98	97.04	96.97	96.99	96.95	96.45	96.56	96.49	96.50
**117**	96.83	96.90	97.04	96.89	96.89	96.86	96.48	96.52	96.50	96.54
**118**	96.84	96.91	96.97	96.94	96.89	96.84	96.46	96.53	96.55	96.58
**119**	96.41	96.47	96.56	96.49	96.47	96.42	96.45	96.53	96.47	96.50
**120**	96.45	96.48	96.52	96.56	96.46	96.41	96.36	96.52	96.50	96.50
**121**	95.61	95.48	95.57	95.48	95.26	95.43	95.39	95.54	95.46	95.27
**122**	96.39	96.40	96.57	96.45	96.38	96.44	96.27	96.43	96.32	96.47
**123**	96.90	96.98	97.05	96.97	96.97	96.89	96.69	96.63	96.68	96.75
**124**	96.94	96.99	97.07	97.00	96.97	96.88	96.64	96.60	96.68	96.73
**125**	96.87	97.01	97.00	97.01	96.99	96.96	96.64	96.64	96.71	96.73
**126**	96.90	96.89	96.90	96.89	96.92	96.84	96.66	96.71	96.68	96.72
**127**	96.81	96.88	96.90	96.86	96.87	96.82	96.70	96.76	96.76	96.78

**128**	99.16	99.01	99.37	99.03	99.07	98.99	96.23	96.22	96.30	96.41
**129**	99.30	99.40	99.48	99.45	99.45	99.39	96.35	96.43	96.45	96.58
**130**	99.38	99.41	99.49	99.46	99.44	99.47	96.36	96.45	96.41	96.58
**131**	100.00	99.69	99.85	99.34	99.33	99.23	96.27	96.34	96.27	96.44
**132**	99.69	100.00	99.87	99.32	99.30	99.24	96.26	96.35	96.35	96.52
**133**	99.85	99.87	100.00	99.49	99.48	99.42	96.42	96.52	96.48	96.61
**134**	99.34	99.32	99.49	100.00	99.99	99.93	96.26	96.34	96.32	96.56
**135**	99.33	99.30	99.48	99.99	100.00	99.94	96.23	96.38	96.28	96.53
**136**	99.23	99.24	99.42	99.93	99.94	100.00	96.18	96.29	96.29	96.42
**137**	96.27	96.26	96.42	96.26	96.23	96.18	100.00	98.97	98.74	98.52
**138**	96.34	96.35	96.52	96.34	96.38	96.29	98.97	100.00	98.72	98.59
**139**	96.27	96.35	96.48	96.32	96.28	96.29	98.74	98.72	100.00	98.77
**140**	96.44	96.52	96.61	96.56	96.53	96.42	98.52	98.59	98.77	100.00
**141**	96.14	96.11	96.18	96.17	96.17	96.14	96.54	96.58	96.56	96.48
**142**	96.40	96.47	96.49	96.45	96.41	96.37	96.84	96.81	96.85	96.88
**143**	96.47	96.46	96.59	96.42	96.41	96.36	96.72	96.84	96.83	96.84
**144**	95.33	95.41	95.53	95.46	95.44	95.39	95.45	95.61	95.64	95.57
**145**	95.19	95.32	95.39	95.36	95.30	95.24	95.30	95.24	95.32	95.37
**146**	95.59	95.51	95.71	95.54	95.50	95.49	95.46	95.43	95.55	95.47
**147**	95.93	95.71	95.93	95.80	95.89	95.52	96.00	95.92	96.03	96.14
**148**	91.78	91.85	91.94	91.72	91.74	91.74	92.13	92.07	92.03	91.98
**149**	91.79	91.86	91.98	91.83	91.82	91.78	92.11	92.11	92.07	92.03
**150**	91.85	91.83	91.89	91.80	91.78	91.78	92.11	92.04	92.11	92.00
**151**	91.79	91.81	91.85	91.84	91.79	91.79	91.98	92.08	92.08	91.98
**152**	91.79	91.81	91.85	91.84	91.79	91.79	91.98	92.08	92.08	91.98
**153**	91.72	91.81	91.82	91.84	91.75	91.75	92.17	91.96	92.07	92.00
**154**	91.66	91.78	91.77	91.73	91.76	91.73	91.94	91.98	91.97	91.95
**155**	91.64	91.70	91.73	91.70	91.65	91.75	91.97	91.94	91.97	91.93
**156**	91.74	91.85	91.88	91.86	91.89	91.84	92.00	91.99	91.97	91.99
**157**	91.68	91.76	91.82	91.81	91.76	91.78	92.07	91.88	91.90	91.94
**158**	92.03	92.03	92.17	92.05	92.08	92.05	92.09	92.11	92.14	92.10
**159**	94.05	94.14	94.18	94.14	94.11	94.09	93.92	93.97	93.99	94.00
**160**	93.73	93.75	93.78	93.71	93.68	93.69	93.51	93.50	93.57	93.55
**161**	93.56	93.63	93.74	93.59	93.55	93.50	93.50	93.45	93.53	93.51
**162**	83.79	83.79	83.84	83.75	83.77	83.80	83.76	83.78	83.78	83.79
**163**	83.84	83.84	83.84	83.82	83.83	83.84	83.80	83.80	83.84	83.83
**164**	83.81	83.77	83.79	83.86	83.90	83.95	83.81	83.82	83.77	83.85
**165**	83.81	83.84	83.85	83.71	83.79	83.83	83.83	83.83	83.96	83.91
**166**	83.82	83.90	83.86	83.82	83.80	83.86	83.82	83.94	83.89	83.87

Table S3. (continued)

**Isolate**	**141**	**142**	**143**	**144**	**145**	**146**	**147**	**148**	**149**	**150**
**1**	96.22	96.54	96.63	95.48	95.26	95.62	96.59	91.99	91.97	91.95
**2**	96.17	96.52	96.55	95.37	95.22	95.57	96.56	91.86	91.89	91.83
**3**	96.20	96.49	96.61	95.40	95.21	95.53	96.54	91.94	91.88	91.86
**4**	96.21	96.56	96.61	95.45	95.30	95.65	96.45	92.01	91.99	91.91
**5**	96.23	96.51	96.51	95.41	95.26	95.60	96.59	91.93	91.94	91.91
**6**	96.27	96.56	96.64	95.54	95.29	95.69	96.65	91.91	92.02	91.89
**7**	96.23	96.49	96.52	95.57	95.30	95.71	96.53	92.00	92.04	91.96
**8**	96.18	96.55	96.54	95.48	95.21	95.64	96.50	91.99	91.95	91.89
**9**	96.21	96.54	96.61	95.42	95.21	95.77	96.64	91.96	91.96	91.93
**10**	96.21	96.57	96.56	95.48	95.39	95.72	96.58	91.94	91.94	91.93
**11**	96.16	96.61	96.63	95.46	95.28	95.68	96.55	91.95	91.98	91.88
**12**	96.21	96.53	96.62	95.49	95.31	95.71	96.58	91.98	92.01	91.93
**13**	96.24	96.55	96.62	95.51	95.32	95.62	96.56	91.96	92.01	91.92
**14**	96.20	96.53	96.63	95.44	95.27	95.64	96.50	91.92	91.95	91.91
**15**	96.17	96.53	96.62	95.50	95.34	95.73	96.64	91.90	91.97	91.91
**16**	96.25	96.52	96.61	95.54	95.40	95.74	96.50	91.99	92.05	91.99
**17**	96.20	96.55	96.60	95.48	95.22	95.68	96.36	91.95	91.98	91.92
**18**	96.24	96.52	96.57	95.45	95.30	95.57	96.55	91.95	92.00	91.91
**19**	96.28	96.56	96.68	95.61	95.46	95.69	96.51	91.95	91.98	91.86
**20**	96.19	96.59	96.57	95.46	95.30	95.62	96.52	91.87	91.92	91.93
**21**	96.21	96.57	96.60	95.44	95.30	95.62	96.44	91.93	91.93	91.93
**22**	96.23	96.51	96.50	95.45	95.29	95.66	96.46	91.94	91.91	91.98
**23**	96.27	96.65	96.67	95.43	95.38	95.71	96.63	91.91	92.01	91.89
**24**	96.32	96.67	96.63	95.53	95.41	95.79	96.58	91.97	91.96	91.88
**25**	96.34	96.69	96.68	95.60	95.48	95.80	96.58	91.92	91.97	91.93
**26**	96.29	96.66	96.62	95.47	95.36	95.69	96.39	91.93	91.96	91.95
**27**	96.20	96.55	96.59	95.46	95.30	95.67	96.50	91.92	91.98	91.89
**28**	96.24	96.63	96.55	95.46	95.36	95.67	96.62	91.89	91.92	91.94
**29**	96.21	96.52	96.60	95.49	95.33	95.83	96.52	91.95	92.00	91.97
**30**	96.19	96.53	96.55	95.45	95.38	95.75	96.58	91.89	91.89	91.85
**31**	96.19	96.51	96.63	95.45	95.35	95.69	96.58	91.91	91.94	91.90
**32**	96.21	96.47	96.55	95.42	95.26	95.58	96.63	91.94	91.95	91.91
**33**	96.22	96.53	96.57	95.41	95.25	95.64	96.62	91.91	91.91	91.94
**34**	96.26	96.60	96.67	95.49	95.46	95.79	96.53	91.98	92.00	91.96
**35**	96.17	96.47	96.46	95.33	95.28	95.58	96.62	91.84	91.84	91.93
**36**	96.26	96.57	96.54	95.43	95.27	95.60	96.52	91.93	91.99	91.96
**37**	96.18	96.51	96.50	95.40	95.31	95.59	96.59	91.83	91.80	91.90
**38**	96.21	96.53	96.60	95.46	95.26	95.67	96.51	91.88	91.93	91.90
**39**	96.22	96.61	96.63	95.40	95.22	95.72	96.61	91.92	92.00	91.95
**40**	96.29	96.54	96.66	95.50	95.33	95.71	96.51	91.91	91.97	91.90
**41**	96.26	96.54	96.59	95.50	95.32	95.61	96.36	91.96	92.01	91.94

**42**	96.30	96.53	96.62	95.48	95.29	95.70	96.45	91.96	92.04	91.92
**43**	96.27	96.60	96.57	95.48	95.30	95.67	96.44	91.95	92.02	92.00
**44**	96.27	96.50	96.62	95.44	95.29	95.68	96.44	91.94	92.04	91.93
**45**	96.35	96.62	96.68	95.52	95.30	95.86	96.34	91.96	92.02	91.96
**46**	96.28	96.55	96.63	95.49	95.32	95.68	96.36	91.97	92.02	91.97
**47**	96.22	96.53	96.60	95.43	95.21	95.63	96.55	91.89	91.92	91.89
**48**	96.35	96.63	96.68	95.48	95.41	95.69	96.46	91.92	91.97	91.95
**49**	96.28	96.52	96.60	95.56	95.41	95.65	96.48	91.96	92.03	91.95
**50**	96.35	96.69	96.67	95.47	95.40	95.69	96.49	91.96	92.01	91.97
**51**	96.26	96.59	96.54	95.46	95.32	95.71	96.55	91.98	92.00	91.89
**52**	96.23	96.57	96.42	95.31	95.28	95.39	96.43	91.84	91.84	91.89
**53**	96.42	96.68	96.76	95.57	95.47	95.79	96.62	91.97	92.01	92.03
**54**	96.41	96.59	96.77	95.61	95.45	95.72	96.58	91.96	91.96	92.02
**55**	96.28	96.55	96.53	95.52	95.42	95.59	96.35	91.94	91.90	92.03
**56**	96.29	96.52	96.52	95.51	95.41	95.60	96.33	91.95	91.96	92.04
**57**	96.41	96.68	96.78	95.68	95.55	95.75	96.40	91.95	92.01	92.03
**58**	96.40	96.63	96.76	95.63	95.51	95.74	96.60	91.98	92.00	91.96
**59**	96.22	96.63	96.57	95.51	95.40	95.66	96.45	91.97	92.01	91.94
**60**	96.22	96.63	96.57	95.51	95.40	95.66	96.45	91.97	92.01	91.94
**61**	96.25	96.57	96.50	95.43	95.33	95.73	96.45	91.91	91.91	91.94
**62**	96.29	96.57	96.54	95.40	95.30	95.70	96.44	91.85	91.98	91.91
**63**	96.22	96.64	96.57	95.49	95.30	95.68	96.48	91.98	92.03	91.97
**64**	96.23	96.63	96.58	95.47	95.42	95.70	96.50	91.97	91.92	91.94
**65**	96.22	96.56	96.53	95.48	95.32	95.67	96.45	91.97	92.04	91.94
**66**	96.21	96.56	96.43	95.40	95.38	95.75	96.46	91.91	91.94	91.94
**67**	96.21	96.55	96.52	95.36	95.33	95.56	96.44	91.87	91.90	91.81
**68**	96.24	96.55	96.55	95.47	95.28	95.62	96.54	91.85	91.95	91.91
**69**	96.28	96.60	96.55	95.38	95.34	95.68	96.39	91.93	91.93	91.89
**70**	96.24	96.58	96.59	95.44	95.33	95.64	96.49	91.95	91.97	91.97
**71**	96.21	96.51	96.47	95.41	95.29	95.56	96.50	91.89	91.87	91.95
**72**	96.20	96.51	96.51	95.38	95.34	95.72	96.54	91.90	91.95	91.87
**73**	96.31	96.62	96.60	95.46	95.37	95.67	96.47	91.91	92.01	91.93
**74**	96.20	96.50	96.55	95.41	95.31	95.57	96.42	91.89	91.92	91.95
**75**	96.30	96.67	96.67	95.54	95.28	95.71	96.51	92.01	92.10	91.95
**76**	96.29	96.58	96.49	95.40	95.28	95.55	96.48	91.85	91.92	91.95
**77**	96.23	96.60	96.51	95.45	95.32	95.55	96.59	91.92	91.97	92.03
**78**	96.38	96.67	96.77	95.64	95.48	95.77	96.59	91.97	92.05	92.00
**79**	96.23	96.56	96.60	95.49	95.42	95.74	96.46	91.83	91.93	91.88
**80**	96.32	96.63	96.79	95.57	95.36	95.74	96.37	91.98	92.03	91.92
**81**	96.29	96.58	96.58	95.61	95.44	95.66	96.58	91.94	91.99	91.93
**82**	96.28	96.55	96.61	95.56	95.40	95.66	96.54	92.01	91.95	91.98
**83**	96.22	96.43	96.48	95.41	95.26	95.56	96.47	91.80	91.79	91.86
**84**	96.16	96.58	96.51	95.47	95.35	95.65	96.50	91.90	91.99	91.96

**85**	96.27	96.58	96.61	95.51	95.40	95.60	96.47	91.89	91.90	91.96
**86**	96.23	96.54	96.55	95.49	95.29	95.66	96.53	91.91	91.96	91.89
**87**	96.28	96.54	96.57	95.46	95.33	95.62	96.48	91.96	91.93	91.97
**88**	96.32	96.58	96.60	95.48	95.37	95.58	96.54	91.95	91.93	91.98
**89**	96.31	96.57	96.60	95.49	95.46	95.67	96.42	91.89	91.93	91.99
**90**	96.36	96.56	96.69	95.56	95.45	95.70	96.54	92.00	91.97	91.96
**91**	96.27	96.52	96.63	95.45	95.40	95.67	96.51	91.87	91.94	91.88
**92**	96.31	96.57	96.74	95.47	95.39	95.73	96.45	91.93	92.01	91.94
**93**	96.29	96.55	96.65	95.52	95.42	95.70	96.46	91.91	92.04	91.94
**94**	96.26	96.56	96.63	95.57	95.41	95.67	96.53	91.95	92.01	91.95
**95**	96.28	96.61	96.66	95.51	95.41	95.72	96.47	91.91	92.03	91.94
**96**	96.35	96.56	96.69	95.54	95.48	95.69	96.48	91.98	92.02	91.87
**97**	96.32	96.50	96.69	95.58	95.43	95.69	96.68	91.97	92.01	91.93
**98**	96.31	96.61	96.79	95.61	95.45	95.83	96.48	92.00	92.04	91.97
**99**	96.30	96.64	96.68	95.53	95.41	95.84	96.57	92.03	91.98	91.94
**100**	96.31	96.49	96.68	95.55	95.36	95.61	96.48	91.92	91.94	91.96
**101**	96.26	96.54	96.65	95.53	95.39	95.64	96.56	91.92	91.91	91.90
**102**	96.34	96.59	96.68	95.61	95.46	95.70	96.61	91.92	91.99	91.93
**103**	96.25	96.60	96.62	95.58	95.46	95.69	96.47	91.91	91.96	91.83
**104**	96.30	96.57	96.62	95.55	95.44	95.66	96.51	91.96	92.02	92.01
**105**	96.34	96.59	96.66	95.52	95.50	95.61	96.57	91.83	91.92	91.87
**106**	96.35	96.61	96.72	95.57	95.49	95.65	96.56	91.94	91.96	91.96
**107**	96.22	96.56	96.48	95.45	95.34	95.53	96.49	91.88	91.94	91.93
**108**	96.20	96.55	96.44	95.42	95.30	95.59	96.43	91.92	91.95	91.93
**109**	96.39	96.60	96.68	95.64	95.47	95.81	96.38	92.11	92.09	92.04
**110**	96.22	96.56	96.44	95.42	95.35	95.69	96.37	91.86	91.97	91.92
**111**	96.26	96.60	96.49	95.41	95.29	95.62	96.41	91.93	92.00	91.90
**112**	96.30	96.58	96.53	95.42	95.32	95.66	96.43	91.90	92.00	91.90
**113**	96.25	96.56	96.47	95.38	95.29	95.61	96.51	91.88	91.96	91.91
**114**	96.23	96.54	96.46	95.48	95.30	95.75	96.48	91.89	91.97	91.92
**115**	96.26	96.57	96.51	95.42	95.26	95.75	96.52	91.86	92.01	91.88
**116**	96.32	96.51	96.60	95.49	95.35	95.70	96.43	91.89	91.97	91.86
**117**	96.33	96.48	96.61	95.58	95.37	95.62	96.41	91.93	91.95	91.90
**118**	96.38	96.59	96.63	95.59	95.46	95.70	96.46	91.89	91.90	91.94
**119**	96.18	96.50	96.64	95.51	95.42	95.62	96.34	91.80	91.88	91.85
**120**	96.15	96.49	96.58	95.52	95.39	95.63	96.23	91.79	91.83	91.78
**121**	95.26	95.30	95.78	95.75	95.44	95.78	96.26	90.84	90.90	90.82
**122**	96.15	96.41	96.45	95.55	95.31	95.58	96.14	91.81	91.87	91.77
**123**	96.32	96.69	96.69	95.51	95.39	95.73	96.22	91.89	91.95	91.90
**124**	96.36	96.63	96.74	95.48	95.41	95.68	96.21	91.97	91.90	91.93
**125**	96.39	96.65	96.81	95.54	95.36	95.67	96.21	91.87	91.93	91.87
**126**	96.38	96.64	96.76	95.54	95.52	95.68	96.39	91.86	91.88	91.90
**127**	96.38	96.78	96.86	95.57	95.42	95.73	96.08	91.90	91.95	91.82

**128**	96.06	96.36	96.34	95.36	95.15	95.44	95.88	91.69	91.75	91.87
**129**	96.16	96.51	96.49	95.47	95.27	95.57	95.79	91.84	91.82	91.81
**130**	96.23	96.55	96.47	95.44	95.41	95.77	95.99	91.87	91.95	91.83
**131**	96.14	96.40	96.47	95.33	95.19	95.59	95.93	91.78	91.79	91.85
**132**	96.11	96.47	96.46	95.41	95.32	95.51	95.71	91.85	91.86	91.83
**133**	96.18	96.49	96.59	95.53	95.39	95.71	95.93	91.94	91.98	91.89
**134**	96.17	96.45	96.42	95.46	95.36	95.54	95.80	91.72	91.83	91.80
**135**	96.17	96.41	96.41	95.44	95.30	95.50	95.89	91.74	91.82	91.78
**136**	96.14	96.37	96.36	95.39	95.24	95.49	95.52	91.74	91.78	91.78
**137**	96.54	96.84	96.72	95.45	95.30	95.46	96.00	92.13	92.11	92.11
**138**	96.58	96.81	96.84	95.61	95.24	95.43	95.92	92.07	92.11	92.04
**139**	96.56	96.85	96.83	95.64	95.32	95.55	96.03	92.03	92.07	92.11
**140**	96.48	96.88	96.84	95.57	95.37	95.47	96.14	91.98	92.03	92.00
**141**	100.00	96.35	96.28	95.45	95.32	95.38	95.63	92.03	92.09	92.11
**142**	96.35	100.00	97.46	95.58	95.44	95.63	95.80	91.90	92.00	91.94
**143**	96.28	97.46	100.00	95.60	95.26	95.69	95.85	91.91	91.89	91.83
**144**	95.45	95.58	95.60	100.00	97.40	96.25	95.26	92.35	92.30	92.21
**145**	95.32	95.44	95.26	97.40	100.00	96.04	95.19	91.86	91.91	91.91
**146**	95.38	95.63	95.69	96.25	96.04	100.00	95.24	91.97	92.02	91.91
**147**	95.63	95.80	95.85	95.26	95.19	95.24	100.00	94.36	94.39	94.17
**148**	92.03	91.90	91.91	92.35	91.86	91.97	94.36	100.00	99.08	98.71
**149**	92.09	92.00	91.89	92.30	91.91	92.02	94.39	99.08	100.00	98.71
**150**	92.11	91.94	91.83	92.21	91.91	91.91	94.17	98.71	98.71	100.00
**151**	92.04	91.85	91.91	92.33	91.88	91.90	93.79	98.72	98.73	98.72
**152**	92.04	91.85	91.91	92.33	91.88	91.90	93.79	98.72	98.73	98.72
**153**	92.00	91.84	91.78	92.10	91.94	91.88	94.05	98.50	98.55	98.66
**154**	91.95	91.77	91.85	92.15	91.79	91.83	93.86	97.36	97.29	97.31
**155**	91.98	91.77	91.79	92.12	91.85	91.77	93.83	97.20	97.22	97.23
**156**	92.06	91.88	91.83	92.16	91.90	91.94	93.75	97.34	97.31	97.28
**157**	91.93	91.94	91.74	92.11	91.86	91.91	94.01	97.47	97.45	97.45
**158**	92.17	92.08	92.10	92.13	92.04	92.17	92.56	92.90	92.94	92.89
**159**	93.90	93.91	93.93	94.43	94.27	94.30	93.89	91.50	91.58	91.58
**160**	93.51	93.59	93.42	93.98	93.84	93.91	92.99	91.22	91.31	91.34
**161**	93.46	93.57	93.48	94.09	93.99	93.96	93.17	91.96	91.99	91.97
**162**	83.78	83.79	83.64	83.86	83.84	83.73	84.14	83.40	83.45	83.48
**163**	83.79	83.87	83.65	83.86	83.83	83.81	84.14	83.37	83.46	83.47
**164**	83.76	83.79	83.76	83.70	83.75	83.77	84.39	83.44	83.47	83.48
**165**	83.78	83.85	83.86	83.74	83.81	83.83	84.09	83.56	83.61	83.59
**166**	83.86	83.83	83.84	83.68	83.82	83.78	84.41	83.53	83.55	83.48

Table S3. (continued)

**Isolate**	**151**	**152**	**153**	**154**	**155**	**156**	**157**	**158**	**159**	**160**
**1**	91.94	91.94	91.88	91.88	91.84	91.89	91.86	92.15	94.10	93.63
**2**	91.85	91.85	91.83	91.73	91.70	91.78	91.70	92.04	94.00	93.59
**3**	91.84	91.84	91.88	91.77	91.75	91.79	91.78	92.11	94.04	93.52
**4**	91.94	91.94	91.93	91.85	91.84	91.90	91.88	92.12	94.10	93.69
**5**	91.89	91.89	91.86	91.84	91.79	91.83	91.78	92.08	93.97	93.58
**6**	91.98	91.98	91.84	91.88	91.77	91.90	91.86	92.08	94.06	93.49
**7**	91.96	91.96	91.98	91.90	91.81	91.95	91.85	92.20	94.15	93.64
**8**	91.95	91.95	91.86	91.88	91.79	91.84	91.86	92.09	94.09	93.55
**9**	91.92	91.92	91.87	91.82	91.77	91.83	91.78	92.08	93.99	93.58
**10**	91.91	91.91	91.90	91.81	91.83	91.88	91.84	92.14	94.11	93.62
**11**	91.94	91.94	91.89	91.85	91.77	91.88	91.92	92.19	94.10	93.58
**12**	91.98	91.98	91.87	91.87	91.81	91.86	91.83	92.14	94.09	93.56
**13**	91.97	91.97	91.90	91.86	91.87	91.89	91.87	92.14	94.12	93.67
**14**	91.92	91.92	91.94	91.82	91.78	91.84	91.92	92.07	94.02	93.65
**15**	91.95	91.95	91.89	91.84	91.81	91.88	91.90	92.10	94.04	93.59
**16**	91.93	91.93	91.90	91.91	91.86	91.92	91.91	92.16	94.10	93.64
**17**	91.96	91.96	91.88	91.82	91.83	91.86	91.81	92.17	94.02	93.61
**18**	92.00	92.00	91.89	91.87	91.86	91.89	91.85	92.13	94.03	93.48
**19**	91.96	91.96	91.87	91.77	91.82	91.92	91.90	92.13	94.06	93.70
**20**	91.85	91.85	91.90	91.80	91.81	91.86	91.82	92.10	94.01	93.52
**21**	91.89	91.89	91.88	91.80	91.87	91.86	91.73	92.09	94.05	93.54
**22**	91.87	91.87	91.95	91.83	91.87	91.89	91.78	92.17	94.03	93.54
**23**	91.94	91.94	91.87	91.87	91.85	91.91	91.84	92.12	94.12	93.59
**24**	91.92	91.92	91.96	91.84	91.88	91.91	91.91	92.22	94.21	93.72
**25**	91.91	91.91	91.92	91.81	91.85	91.89	91.84	92.23	94.25	93.80
**26**	91.91	91.91	91.86	91.81	91.83	91.86	91.83	92.19	94.11	93.70
**27**	91.93	91.93	91.84	91.83	91.80	91.85	91.80	92.09	94.03	93.60
**28**	91.88	91.88	91.94	91.77	91.86	91.88	91.86	92.14	94.13	93.72
**29**	91.95	91.95	91.84	91.80	91.80	91.87	91.81	92.16	94.01	93.66
**30**	91.93	91.93	91.80	91.78	91.74	91.85	91.84	92.05	94.01	93.55
**31**	91.94	91.94	91.86	91.83	91.78	91.87	91.92	92.10	93.98	93.54
**32**	91.89	91.89	91.83	91.89	91.78	91.83	91.85	92.09	94.04	93.52
**33**	91.90	91.90	91.81	91.80	91.76	91.81	91.77	92.10	94.04	93.64
**34**	91.90	91.90	91.86	91.84	91.82	91.87	91.86	92.16	94.16	93.66
**35**	91.82	91.82	91.85	91.70	91.76	91.79	91.75	92.12	94.08	93.59
**36**	91.89	91.89	91.90	91.79	91.81	91.83	91.79	92.14	94.11	93.58
**37**	91.86	91.86	91.87	91.72	91.75	91.83	91.83	92.07	94.04	93.59
**38**	91.90	91.90	91.80	91.82	91.73	91.82	91.81	92.10	94.09	93.59
**39**	91.92	91.92	91.86	91.80	91.75	91.77	91.86	92.21	93.95	93.61
**40**	91.96	91.96	91.84	91.85	91.76	91.89	91.85	92.16	94.11	93.65
**41**	91.99	91.99	91.92	91.89	91.82	91.89	91.87	92.11	94.10	93.70

**42**	92.01	92.01	91.95	91.92	91.89	91.91	91.88	92.10	94.06	93.65
**43**	91.95	91.95	91.90	91.89	91.85	91.93	91.90	92.12	94.13	93.62
**44**	91.97	91.97	91.93	91.86	91.83	91.91	91.86	92.08	94.09	93.73
**45**	91.89	91.89	91.85	91.89	91.83	91.91	91.88	92.22	94.17	93.68
**46**	91.94	91.94	91.92	91.91	91.85	91.93	91.87	92.08	94.14	93.65
**47**	91.92	91.92	91.81	91.79	91.82	91.82	91.80	92.15	94.12	93.64
**48**	91.91	91.91	91.84	91.83	91.82	91.81	91.80	92.16	94.15	93.69
**49**	91.95	91.95	91.84	91.88	91.83	91.91	91.84	92.15	94.09	93.89
**50**	91.96	91.96	91.94	91.83	91.88	91.90	91.86	92.22	94.21	93.75
**51**	91.91	91.91	92.00	91.87	91.82	91.89	91.95	92.17	94.07	93.69
**52**	91.81	91.81	91.84	91.76	91.71	91.79	91.75	92.05	93.95	93.51
**53**	91.89	91.89	91.92	91.88	91.85	91.95	91.88	92.22	94.28	93.82
**54**	91.96	91.96	91.86	91.85	91.88	91.91	91.82	92.23	94.22	93.81
**55**	91.87	91.87	91.90	91.81	91.78	91.84	91.78	92.10	94.02	93.65
**56**	91.92	91.92	91.89	91.91	91.88	91.93	91.81	92.07	94.08	93.74
**57**	92.00	92.00	91.92	91.88	91.88	91.96	91.87	92.30	94.27	93.86
**58**	91.91	91.91	91.92	91.81	91.84	91.89	91.86	92.23	94.21	93.81
**59**	91.95	91.95	91.98	91.86	91.86	91.92	91.87	92.09	93.98	93.65
**60**	91.95	91.95	91.98	91.86	91.86	91.92	91.87	92.09	93.98	93.65
**61**	91.93	91.93	91.90	91.80	91.85	91.88	91.82	92.17	94.08	93.66
**62**	91.83	91.83	91.87	91.79	91.83	91.87	91.90	92.18	94.12	93.61
**63**	91.97	91.97	91.92	91.85	91.84	91.90	91.94	92.09	94.13	93.68
**64**	91.92	91.92	91.97	91.84	91.77	91.89	91.93	92.11	94.05	93.54
**65**	91.95	91.95	91.96	91.85	91.80	91.88	91.93	92.09	94.11	93.60
**66**	91.88	91.88	91.95	91.82	91.83	91.95	91.89	92.11	94.10	93.66
**67**	91.90	91.90	91.93	91.75	91.79	91.88	91.93	92.08	93.99	93.59
**68**	91.87	91.87	91.92	91.81	91.88	91.94	91.83	92.12	94.18	93.66
**69**	91.83	91.83	91.92	91.80	91.79	91.87	91.89	92.12	94.03	93.55
**70**	91.92	91.92	91.89	91.77	91.87	91.93	91.81	92.13	94.14	93.56
**71**	91.84	91.84	91.81	91.73	91.79	91.90	91.73	92.05	94.10	93.67
**72**	91.93	91.93	91.90	91.78	91.86	91.89	91.83	92.12	93.99	93.54
**73**	91.88	91.88	91.92	91.77	91.90	91.90	91.86	92.22	94.17	93.73
**74**	91.85	91.85	91.84	91.76	91.82	91.83	91.80	92.09	94.04	93.59
**75**	91.95	91.95	91.84	91.93	91.89	91.94	91.89	92.20	94.17	93.69
**76**	91.88	91.88	91.91	91.81	91.82	91.86	91.82	92.11	94.11	93.64
**77**	91.90	91.90	91.90	91.81	91.73	91.85	91.89	92.07	94.03	93.67
**78**	92.02	92.02	91.90	91.91	91.94	92.04	91.92	92.18	94.13	93.94
**79**	91.93	91.93	91.83	91.77	91.80	91.91	91.77	92.12	94.08	93.73
**80**	91.95	91.95	91.83	91.93	91.88	91.94	91.88	92.13	94.19	93.80
**81**	91.96	91.96	91.84	91.89	91.82	92.01	91.91	92.13	94.11	93.79
**82**	91.94	91.94	91.77	91.89	91.84	91.94	91.87	92.07	94.12	93.83
**83**	91.79	91.79	91.83	91.73	91.75	91.81	91.71	92.11	94.05	93.51
**84**	91.91	91.91	91.99	91.80	91.88	91.92	91.94	92.15	94.08	93.68

**85**	91.89	91.89	91.79	91.78	91.83	91.90	91.82	92.09	94.01	93.69
**86**	91.90	91.90	91.93	91.80	91.84	91.89	91.87	92.15	94.07	93.57
**87**	91.90	91.90	91.97	91.82	91.90	91.93	91.84	92.15	94.18	93.64
**88**	91.88	91.88	91.87	91.77	91.83	91.95	91.77	92.15	94.09	93.80
**89**	91.83	91.83	91.94	91.77	91.81	91.86	91.79	92.14	94.10	93.85
**90**	91.95	91.95	91.87	91.79	91.79	91.86	91.86	92.14	94.10	93.79
**91**	91.86	91.86	91.86	91.76	91.67	91.77	91.85	92.05	94.03	93.77
**92**	91.91	91.91	91.84	91.80	91.80	91.91	91.81	92.11	94.06	93.73
**93**	91.92	91.92	91.93	91.83	91.83	91.95	91.87	92.08	94.03	93.75
**94**	91.89	91.89	91.90	91.79	91.74	91.97	91.94	92.07	94.05	93.73
**95**	91.92	91.92	91.98	91.84	91.84	91.87	91.94	92.10	94.10	93.76
**96**	91.91	91.91	91.92	91.86	91.87	91.94	91.90	92.17	94.07	93.72
**97**	91.97	91.97	91.88	91.80	91.82	91.92	91.85	92.11	94.07	93.71
**98**	91.90	91.90	91.87	91.88	91.87	91.94	91.86	92.19	94.19	93.83
**99**	91.94	91.94	92.01	91.82	91.86	91.89	91.97	92.15	94.07	93.90
**100**	91.92	91.92	91.83	91.85	91.81	91.90	91.76	92.07	94.02	93.81
**101**	91.90	91.90	91.82	91.82	91.76	91.90	91.80	92.11	94.01	93.81
**102**	91.92	91.92	91.82	91.85	91.84	91.99	91.83	92.14	94.20	93.80
**103**	91.85	91.85	91.83	91.76	91.79	91.92	91.83	92.09	94.03	93.72
**104**	92.04	92.04	91.89	91.89	91.86	92.00	91.92	92.07	94.04	93.63
**105**	91.83	91.83	91.89	91.82	91.78	91.90	91.89	92.13	94.14	93.81
**106**	91.86	91.86	91.94	91.85	91.75	91.94	91.89	92.20	94.15	93.76
**107**	91.87	91.87	91.92	91.84	91.82	91.84	91.77	92.11	94.14	93.78
**108**	91.86	91.86	91.94	91.79	91.82	91.92	91.89	92.13	94.18	93.62
**109**	92.06	92.06	91.96	92.09	91.98	92.03	92.01	92.26	94.29	93.82
**110**	91.85	91.85	91.89	91.84	91.82	91.85	91.85	92.20	94.13	93.71
**111**	91.85	91.85	91.91	91.78	91.82	91.90	91.86	92.16	94.21	93.66
**112**	91.86	91.86	91.93	91.87	91.85	91.86	91.81	92.21	94.21	93.71
**113**	91.85	91.85	91.91	91.80	91.81	91.86	91.79	92.14	94.14	93.71
**114**	91.88	91.88	91.91	91.77	91.80	91.86	91.79	92.16	94.14	93.80
**115**	91.87	91.87	91.91	91.81	91.81	91.85	91.79	92.17	94.19	93.73
**116**	91.89	91.89	91.80	91.80	91.80	91.87	91.81	92.11	94.26	93.68
**117**	91.99	91.99	91.83	91.86	91.79	91.88	91.80	92.13	94.05	93.75
**118**	91.88	91.88	91.87	91.74	91.86	91.94	91.82	92.10	94.16	93.86
**119**	91.79	91.79	91.74	91.71	91.74	91.82	91.73	92.17	94.05	93.72
**120**	91.77	91.77	91.71	91.69	91.73	91.89	91.74	92.12	94.05	93.78
**121**	90.45	90.45	90.53	90.89	91.04	90.83	90.61	90.92	92.91	91.97
**122**	91.85	91.85	91.78	91.79	91.70	91.87	91.73	92.09	94.09	93.61
**123**	91.88	91.88	91.82	91.77	91.78	91.88	91.96	92.17	94.01	93.82
**124**	91.87	91.87	91.74	91.84	91.80	91.86	91.86	92.17	93.96	93.71
**125**	91.89	91.89	91.76	91.81	91.80	91.84	91.79	92.19	94.11	93.80
**126**	91.86	91.86	91.81	91.81	91.82	91.85	91.79	92.15	94.14	93.67
**127**	91.88	91.88	91.80	91.85	91.86	91.90	91.85	92.16	94.18	93.82

**128**	91.72	91.72	91.63	91.67	91.65	91.76	91.69	92.04	93.91	93.50
**129**	91.83	91.83	91.77	91.74	91.77	91.82	91.72	92.12	94.17	93.76
**130**	91.78	91.78	91.88	91.73	91.81	91.90	91.83	92.08	94.07	93.77
**131**	91.79	91.79	91.72	91.66	91.64	91.74	91.68	92.03	94.05	93.73
**132**	91.81	91.81	91.81	91.78	91.70	91.85	91.76	92.03	94.14	93.75
**133**	91.85	91.85	91.82	91.77	91.73	91.88	91.82	92.17	94.18	93.78
**134**	91.84	91.84	91.84	91.73	91.70	91.86	91.81	92.05	94.14	93.71
**135**	91.79	91.79	91.75	91.76	91.65	91.89	91.76	92.08	94.11	93.68
**136**	91.79	91.79	91.75	91.73	91.75	91.84	91.78	92.05	94.09	93.69
**137**	91.98	91.98	92.17	91.94	91.97	92.00	92.07	92.09	93.92	93.51
**138**	92.08	92.08	91.96	91.98	91.94	91.99	91.88	92.11	93.97	93.50
**139**	92.08	92.08	92.07	91.97	91.97	91.97	91.90	92.14	93.99	93.57
**140**	91.98	91.98	92.00	91.95	91.93	91.99	91.94	92.10	94.00	93.55
**141**	92.04	92.04	92.00	91.95	91.98	92.06	91.93	92.17	93.90	93.51
**142**	91.85	91.85	91.84	91.77	91.77	91.88	91.94	92.08	93.91	93.59
**143**	91.91	91.91	91.78	91.85	91.79	91.83	91.74	92.10	93.93	93.42
**144**	92.33	92.33	92.10	92.15	92.12	92.16	92.11	92.13	94.43	93.98
**145**	91.88	91.88	91.94	91.79	91.85	91.90	91.86	92.04	94.27	93.84
**146**	91.90	91.90	91.88	91.83	91.77	91.94	91.91	92.17	94.30	93.91
**147**	93.79	93.79	94.05	93.86	93.83	93.75	94.01	92.56	93.89	92.99
**148**	98.72	98.72	98.50	97.36	97.20	97.34	97.47	92.90	91.50	91.22
**149**	98.73	98.73	98.55	97.29	97.22	97.31	97.45	92.94	91.58	91.31
**150**	98.72	98.72	98.66	97.31	97.23	97.28	97.45	92.89	91.58	91.34
**151**	100.00	100.00	98.57	97.30	97.21	97.26	97.46	92.85	91.69	91.25
**152**	100.00	100.00	98.57	97.30	97.21	97.26	97.46	92.85	91.69	91.25
**153**	98.57	98.57	100.00	97.25	97.22	97.28	97.38	92.79	91.57	91.26
**154**	97.30	97.30	97.25	100.00	99.70	98.08	97.31	92.92	91.51	91.20
**155**	97.21	97.21	97.22	99.70	100.00	98.04	97.20	92.91	91.60	91.18
**156**	97.26	97.26	97.28	98.08	98.04	100.00	97.41	92.99	91.64	91.24
**157**	97.46	97.46	97.38	97.31	97.20	97.41	100.00	92.92	91.48	91.18
**158**	92.85	92.85	92.79	92.92	92.91	92.99	92.92	100.00	91.68	91.50
**159**	91.69	91.69	91.57	91.51	91.60	91.64	91.48	91.68	100.00	98.15
**160**	91.25	91.25	91.26	91.20	91.18	91.24	91.18	91.50	98.15	100.00
**161**	91.96	91.96	91.92	91.99	91.86	92.05	91.96	91.74	95.68	95.12
**162**	83.59	83.59	83.40	83.35	83.41	83.44	83.38	83.57	83.56	83.43
**163**	83.56	83.56	83.39	83.33	83.39	83.48	83.45	83.62	83.52	83.43
**164**	83.56	83.56	83.44	83.41	83.48	83.48	83.50	83.57	83.54	83.54
**165**	83.54	83.54	83.55	83.47	83.54	83.51	83.51	83.63	83.57	83.59
**166**	83.65	83.65	83.57	83.54	83.65	83.55	83.62	83.70	83.58	83.52

Table S3. (continued)

**Isolate**	**161**	**162**	**163**	**164**	**165**	**166**
**1**	93.60	83.80	83.75	83.82	83.85	83.87
**2**	93.52	83.67	83.62	83.78	83.82	83.85
**3**	93.51	83.79	83.74	83.84	83.89	83.96
**4**	93.59	83.79	83.74	83.87	83.93	83.91
**5**	93.53	83.79	83.80	83.89	83.93	83.94
**6**	93.62	83.80	83.80	83.83	83.91	83.95
**7**	93.61	83.81	83.82	83.88	83.93	83.94
**8**	93.57	83.76	83.78	83.83	83.90	83.86
**9**	93.53	83.73	83.72	83.88	83.91	83.91
**10**	93.61	83.78	83.77	83.75	83.89	83.91
**11**	93.52	83.78	83.80	83.88	83.90	83.86
**12**	93.57	83.79	83.79	83.87	83.94	83.85
**13**	93.60	83.81	83.81	83.86	83.97	83.90
**14**	93.52	83.82	83.84	83.86	83.81	83.89
**15**	93.53	83.79	83.76	83.78	83.92	83.87
**16**	93.61	83.79	83.80	83.85	83.97	83.97
**17**	93.55	83.79	83.75	83.86	83.86	83.87
**18**	93.57	83.83	83.86	83.89	83.94	83.86
**19**	93.64	83.79	83.82	83.76	83.86	83.81
**20**	93.50	83.75	83.75	83.86	83.89	83.89
**21**	93.52	83.83	83.76	83.93	83.90	83.98
**22**	93.56	83.81	83.75	83.92	83.87	83.97
**23**	93.60	83.80	83.82	83.86	83.88	83.87
**24**	93.67	83.82	83.89	83.85	83.95	83.93
**25**	93.68	83.90	83.87	83.91	84.04	84.04
**26**	93.59	83.82	83.80	83.86	83.86	83.83
**27**	93.58	83.78	83.78	83.82	83.85	83.87
**28**	93.56	83.82	83.78	83.86	84.05	83.91
**29**	93.51	83.71	83.70	83.85	83.87	83.91
**30**	93.56	83.73	83.79	83.88	83.88	83.91
**31**	93.48	83.76	83.82	83.91	83.92	83.99
**32**	93.60	83.73	83.77	83.90	83.89	83.89
**33**	93.57	83.70	83.68	83.82	83.91	83.94
**34**	93.57	83.73	83.78	83.88	83.98	83.97
**35**	93.51	83.75	83.73	83.95	84.05	83.98
**36**	93.56	83.77	83.78	83.92	83.88	83.93
**37**	93.52	83.80	83.79	83.85	83.89	83.96
**38**	93.55	83.78	83.77	83.81	83.94	83.93
**39**	93.57	83.75	83.79	83.79	83.91	83.86
**40**	93.59	83.86	83.82	83.90	83.88	83.89
**41**	93.71	83.77	83.80	83.91	83.96	83.89

**42**	93.62	83.76	83.82	83.93	83.89	83.96
**43**	93.60	83.82	83.82	83.96	83.86	83.96
**44**	93.68	83.78	83.83	83.86	83.86	83.94
**45**	93.60	83.79	83.78	83.91	83.87	83.94
**46**	93.63	83.78	83.74	83.87	83.89	83.87
**47**	93.60	83.84	83.83	83.96	83.93	83.95
**48**	93.68	83.86	83.86	84.02	84.02	83.90
**49**	93.66	83.86	83.85	83.87	83.86	83.82
**50**	93.67	83.86	83.87	83.94	83.87	83.91
**51**	93.53	83.75	83.80	83.87	83.89	83.91
**52**	93.50	83.78	83.80	83.87	83.96	83.88
**53**	93.80	83.87	83.84	84.02	83.94	84.01
**54**	93.77	83.80	83.84	83.88	83.95	83.92
**55**	93.65	83.75	83.80	83.91	83.92	83.89
**56**	93.71	83.91	83.89	83.89	83.96	84.00
**57**	93.74	83.90	83.93	83.97	84.05	83.98
**58**	93.76	83.80	83.83	83.95	83.95	83.94
**59**	93.56	83.78	83.81	83.90	83.91	83.91
**60**	93.56	83.78	83.81	83.90	83.91	83.91
**61**	93.63	83.77	83.77	83.95	83.93	83.90
**62**	93.61	83.81	83.81	83.93	83.90	83.91
**63**	93.62	83.74	83.75	83.87	83.88	83.86
**64**	93.59	83.79	83.79	83.90	83.89	83.86
**65**	93.62	83.77	83.80	83.92	83.93	83.83
**66**	93.58	83.83	83.84	83.91	83.91	83.96
**67**	93.60	83.81	83.83	83.91	83.92	83.91
**68**	93.58	83.78	83.85	84.00	83.91	83.96
**69**	93.58	83.79	83.76	83.99	83.95	83.85
**70**	93.60	83.80	83.80	83.96	83.95	83.98
**71**	93.60	83.78	83.81	83.93	83.94	83.89
**72**	93.47	83.78	83.83	83.93	83.94	83.93
**73**	93.62	83.77	83.78	83.96	83.96	83.98
**74**	93.56	83.77	83.72	83.93	83.99	83.92
**75**	93.64	83.87	83.84	83.88	83.95	83.96
**76**	93.55	83.80	83.79	83.82	83.93	83.88
**77**	93.64	83.80	83.80	83.81	83.97	83.90
**78**	93.71	83.88	83.87	83.83	83.92	83.90
**79**	93.62	83.81	83.82	83.91	83.92	83.86
**80**	93.71	83.80	83.83	83.92	83.87	83.86
**81**	93.70	83.84	83.89	83.88	83.89	83.92
**82**	93.68	83.78	83.80	83.86	83.91	83.86
**83**	93.56	83.80	83.83	83.96	83.82	84.04
**84**	93.62	83.76	83.79	83.83	83.88	83.90

**85**	93.67	83.85	83.84	83.87	83.85	83.86
**86**	93.75	83.76	83.78	83.90	83.88	83.98
**87**	93.68	83.83	83.83	83.89	83.93	83.91
**88**	93.71	83.75	83.73	83.87	83.87	83.84
**89**	93.65	83.82	83.78	83.96	83.84	83.86
**90**	93.67	83.88	83.85	83.93	83.94	83.95
**91**	93.61	83.77	83.71	83.85	83.86	83.82
**92**	93.65	83.79	83.81	83.90	83.89	83.93
**93**	93.72	83.75	83.81	83.91	83.89	83.83
**94**	93.65	83.85	83.86	83.91	83.86	83.95
**95**	93.68	83.83	83.81	83.94	83.91	83.85
**96**	93.74	83.77	83.82	84.04	83.91	83.82
**97**	93.71	83.84	83.82	83.80	83.88	83.88
**98**	93.73	83.80	83.87	83.95	83.98	83.93
**99**	93.68	83.74	83.85	83.90	83.88	83.82
**100**	93.72	83.78	83.81	83.87	83.86	83.86
**101**	93.63	83.82	83.84	83.95	83.92	83.91
**102**	93.68	83.84	83.83	83.87	83.83	83.83
**103**	93.72	83.82	83.80	83.84	83.84	83.91
**104**	93.68	83.85	83.85	83.90	83.94	83.89
**105**	93.75	83.80	83.84	83.93	83.88	83.88
**106**	93.75	83.86	83.87	84.01	83.95	83.93
**107**	93.63	83.83	83.81	83.87	83.95	83.91
**108**	93.59	83.89	83.86	83.89	83.97	83.90
**109**	93.78	83.97	83.99	83.97	84.09	84.03
**110**	93.63	83.79	83.78	83.85	83.92	83.88
**111**	93.66	83.88	83.86	83.86	83.97	83.93
**112**	93.65	83.81	83.81	83.89	83.91	83.91
**113**	93.62	83.83	83.80	83.91	83.94	83.93
**114**	93.64	83.81	83.82	83.87	83.83	83.89
**115**	93.64	83.76	83.78	83.85	83.88	83.93
**116**	93.62	83.82	83.81	83.85	83.89	83.90
**117**	93.65	83.80	83.80	83.90	83.86	83.89
**118**	93.69	83.77	83.79	83.85	83.87	83.90
**119**	93.59	83.66	83.70	83.78	83.85	83.76
**120**	93.61	83.76	83.75	83.72	83.74	83.72
**121**	92.41	83.07	83.13	83.20	83.44	83.20
**122**	93.60	83.70	83.77	83.80	83.73	83.81
**123**	93.57	83.76	83.81	83.77	83.84	83.81
**124**	93.67	83.72	83.76	83.77	83.82	83.79
**125**	93.66	83.78	83.76	83.78	83.79	83.79
**126**	93.54	83.79	83.76	83.85	83.79	83.77
**127**	93.71	83.83	83.83	83.81	83.85	83.79

**128**	93.44	83.74	83.82	83.78	83.81	83.85
**129**	93.63	83.79	83.82	83.82	83.83	83.85
**130**	93.62	83.77	83.78	83.72	83.83	83.78
**131**	93.56	83.79	83.84	83.81	83.81	83.82
**132**	93.63	83.79	83.84	83.77	83.84	83.90
**133**	93.74	83.84	83.84	83.79	83.85	83.86
**134**	93.59	83.75	83.82	83.86	83.71	83.82
**135**	93.55	83.77	83.83	83.90	83.79	83.80
**136**	93.50	83.80	83.84	83.95	83.83	83.86
**137**	93.50	83.76	83.80	83.81	83.83	83.82
**138**	93.45	83.78	83.80	83.82	83.83	83.94
**139**	93.53	83.78	83.84	83.77	83.96	83.89
**140**	93.51	83.79	83.83	83.85	83.91	83.87
**141**	93.46	83.78	83.79	83.76	83.78	83.86
**142**	93.57	83.79	83.87	83.79	83.85	83.83
**143**	93.48	83.64	83.65	83.76	83.86	83.84
**144**	94.09	83.86	83.86	83.70	83.74	83.68
**145**	93.99	83.84	83.83	83.75	83.81	83.82
**146**	93.96	83.73	83.81	83.77	83.83	83.78
**147**	93.17	84.14	84.14	84.39	84.09	84.41
**148**	91.96	83.40	83.37	83.44	83.56	83.53
**149**	91.99	83.45	83.46	83.47	83.61	83.55
**150**	91.97	83.48	83.47	83.48	83.59	83.48
**151**	91.96	83.59	83.56	83.56	83.54	83.65
**152**	91.96	83.59	83.56	83.56	83.54	83.65
**153**	91.92	83.40	83.39	83.44	83.55	83.57
**154**	91.99	83.35	83.33	83.41	83.47	83.54
**155**	91.86	83.41	83.39	83.48	83.54	83.65
**156**	92.05	83.44	83.48	83.48	83.51	83.55
**157**	91.96	83.38	83.45	83.50	83.51	83.62
**158**	91.74	83.57	83.62	83.57	83.63	83.70
**159**	95.68	83.56	83.52	83.54	83.57	83.58
**160**	95.12	83.43	83.43	83.54	83.59	83.52
**161**	100.00	83.61	83.58	83.59	83.58	83.51
**162**	83.61	100.00	99.96	95.28	93.38	92.97
**163**	83.58	99.96	100.00	95.29	93.36	92.99
**164**	83.59	95.28	95.29	100.00	93.31	92.96
**165**	83.58	93.38	93.36	93.31	100.00	95.92
**166**	83.51	92.97	92.99	92.96	95.92	100.00

Table S4. The prevalence of the 88 ARGs in 166 global *Morganella* isolates

Antimicrobial resistance phenotype	ARGs	Genospecies	Number of isolates	Total number of isolates
Tetracycline resistance	*tetA*(B)	*Morganella morganii*	64	99
	tetA(D)	*M. morganii*	22	
		M. sibonii	9	
		M. chanii	2	
	tetA(A)	*M. morganii*	11	
	tetA(L)	*M. morganii*	1	
	tetA(Y)	*M. sibonii*	1	
Aminoglycoside resistance	*aphA1*	*M. morganii*	29	70
		M. sibonii	1	
	aadA1	*M. morganii*	25	
		M. sibonii	3	
	aacA4cr	*M. morganii*	25	
		M. sibonii	1	
	aacC2d	*M. morganii*	19	
	aadA2	*M. morganii*	15	
		M. sibonii	1	
	aadA5	*M. morganii*	16	
	aph(4)-Ia	*M. morganii*	12	
		M. sibonii	1	
	aacC4	*M. morganii*	10	
		M. sibonii	1	
	aadB	*M. morganii*	10	
		M. sibonii	1	
	aadA2b	*M. morganii*	9	
	aacC2	*M. morganii*	8	
	aacA4	*M. morganii*	5	
	aphA6	*M. morganii*	4	
	aadA16	*M. morganii*	3	
	aadA13	*M. morganii*	2	
	ant(3")-Ia	*M. morganii*	2	
	aacC2e	*M. morganii*	1	
	aadA7	*M. morganii*	1	
	aphD	*M. morganii*	1	
	strA	*M. morganii*	22	
		M. sibonii	1	
	strB	*M. morganii*	23	
		M. sibonii	1	
	armA	*M. morganii*	2	
	rmtB	*M. morganii*	4	
	rmtC	*M. morganii*	1	

Sulphonamide resistance	*sul1*	*M. morganii*	53	62
		M. sibonii	1	
	sul2	*M. morganii*	35	
		M. sibonii	2	
	sul3	*M. morganii*	1	
Trimethoprim resistance	*dfrA1*	*M. morganii*	15	56
		M. sibonii	2	
	dfrA17	*M. morganii*	17	
	dfrA12	*M. morganii*	14	
		M. sibonii	1	
	dfrA19	*M. morganii*	5	
	dfrA14	*M. morganii*	3	
	dfrA15	*M. morganii*	3	
	dfrA27	*M. morganii*	3	
	dfrA7	*M. morganii*	1	
	dfrA10	*M. morganii*	1	
β-lactam resistance	bla_{KPC-2}	*M. morganii*	5	54
	bla_{OXA-1}	*M. morganii*	29	
		M. sibonii	1	
	bla_{TEM-1B}	*M. morganii*	22	
	bla_{NDM-1}	*M. morganii*	8	
	bla_{CARB-2}	*M. morganii*	7	
	bla_{OXA-10}	*M. morganii*	4	
		M. sibonii	1	
	$bla_{CTX-M-3}$	*M. morganii*	5	
	$bla_{CTX-M-15}$	*M. morganii*	5	
	bla_{TEM-1A}	*M. morganii*	2	
	bla_{GES-5}	*M. morganii*	1	
	bla_{NDM-5}	*M. morganii*	1	
	bla_{IMP-1}	*M. morganii*	1	
	bla_{IMP-27}	*M. morganii*	1	
	$bla_{OXA-181}$	*M. morganii*	1	
	$bla_{CTX-M-55}$	*M. morganii*	1	
	$bla_{CTX-M-65}$	*M. morganii*	1	
	$bla_{TEM-110}$	*M. morganii*	1	
	bla_{SHV-12}	*M. morganii*	1	
	bla_{SCO-1}	*M. morganii*	1	
	bla_{CMY-4}	*M. morganii*	1	
Chloramphenicol resistance	*catB3*	*M. morganii*	32	53
		M. sibonii	1	
	catA1	*M. morganii*	26	
		M. chanii	1	
	floR	*M. morganii*	19	

		M. sibonii	2	
	catB2	*M. morganii*	3	
		M. sibonii	1	
	catB8	*M. morganii*	3	
		M. sibonii	1	
	cmlA1	*M. morganii*	2	
	catB5	*M. morganii*	1	
Quaternary ammonium compound resistance	*qnrD1*	*M. morganii*	25	53
		M. sibonii	2	
	aacA4cr	*M. morganii*	25	
		M. sibonii	1	
	qnrVC1	*M. morganii*	3	
	qnrS1	*M. morganii*	2	
	qnrB1	*M. morganii*	1	
	qnrB9	*M. morganii*	1	
	qnrD2	*M. morganii*	1	
	qepA1	*M. morganii*	1	
Quaternary ammonium compound resistance	*qacE*	*M. morganii*	51	52
		M. sibonii	1	
	qacH2	*M. morganii*	1	
Macrolide resistance	*mph*(A)	*M. morganii*	26	34
	mph(E)	*M. morganii*	7	
	ere(B)	*M. morganii*	5	
	erm(42)	*M. morganii*	2	
Rifampicin resistance	*arr-3*	*M. morganii*	22	22
Lincosamide resistance	*lnu*(F)	*M. morganii*	6	7
	lnu(G)	*M. morganii*	1	
Polymycin resistince	*mcr-1.1*	*M. morganii*	3	4
	mcr-5.1	*M. morganii*	1	
Fosfomycin resistance	*fosA3*	*M. morganii*	2	2
Phenicol/lincosamide/oxazolidinone/pleuromutilin/stretogramin A	*cfr*	*M. morganii*	1	1

Table S5. Sub-regions and ARGs in the AGEs characterized in this study

Group	AGEs	Sub-region harbored	ARGs	Antimicrobial resistance phenotype	Nucleotide position
IS*26*/IS*15DI*-composite transposons	Tn*6759*	Disrupted IS*CR1*–*floR* unit	*floR*	Chloramphenicol resistance	3664622..3665836
	Tn*6760*	Type A In37-like element	*catB3*	Chloramphenicol resistance	2992347..4740721
			bla$_{OXA-1}$	β-lactam resistance	2993117..2993992
			aacA4cr	Aminoglycoside resistance/Quniolone resistace	2994078..2994677
		In54	*dfrA17*	Trimethoprim resistance	2996257..2996856
			aadA5	Aminoglycoside resistance	2996987..2997775
			qacED1	Sulphonamide resistance	2997981..2998328
			sul1	Quaternary ammonium compound resistance	2998322..2999161
Tn*10*-related elements	Tn*10*$_{ZJC25}$	Backbone	*tetA*(B)	Tetracycline resistance	1488675..1489880
	Tn*10*$_{229813}$	Backbone	*tetA*(B)	Tetracycline resistance	1521797..1523002
	Tn*10*$_{516602}$	Backbone	*tetA*(B)	Tetracycline resistance	2610485..2609280
	Tn*10*$_{11759}$	Backbone	*tetA*(B)	Tetracycline resistance	1722760..1723965
	Tn*6798*	Backbone	*tetA*(B)	Tetracycline resistance	1804608..1805813
	Tn*6799*	Backbone	*tetA*(B)	Tetracycline resistance	1992791..1993996
		Tn*6970*	*catA1*	Chloramphenicol resistance	1998612..1999271
			sul1	Sulphonamide resistance	2007803..2008345
			qacED1	Quaternary ammonium compound resistance	2008339..2008686
			catB3	Chloramphenicol resistance	2008843..2009475
			aadB	Aminoglycoside resistance	2009558..2010091
			dfrA19	Trimethoprim resistance	2011853..2012422
			sul1	Sulphonamide resistance	2015874..2016713
			qacED1	Quaternary ammonium compound resistance	2016707..2017054
			aadA2	Aminoglycoside resistance	2017218..2017997
			bla$_{CARB-2}$	β-lactam resistance	2018127..2018993
	T10$_{REGN28}$	Backbone	*tetA*(B)	Tetracycline resistance	2308289..2309494
		34.9-kb T2670RE$_{GN28}$	*catA1*	Chloramphenicol resistance	2314110..2314769
			sul1	Sulphonamide resistance	2323002..2323841

			qacED1	Quaternary ammonium compound resistance	2323835..2324182
			arr-3	Rifampicin resistance	2324405..2324857
			catB3	Chloramphenicol resistance	2324942..2325574
			bla$_{OXA-1}$	β-lactam resistance	2325712..2326587
			aacA4cr	Aminoglycoside resistance/Quniolone resistace	2326673..2327272
			aacC2	Aminoglycoside resistance	2328488..2329348
			tmrB	Tunicamycin resistance	2329361..2329903
			mph(A)	Macrolide resistance	2332689..2333594
			chrA	Chromate resistance	2337213..2338418
			sul1	Sulphonamide resistance	2338918..2339757
			qacED1	Quaternary ammonium compound resistance	2339751..2340098
			aadA2	Aminoglycoside resistance	2340262..2341041
			bla$_{CARB-2}$	β-lactam resistance	2341171..2342037
			ereB	Macrolide resistance	2344978..2346237
Tn*7*-related elements	Tn*7*$_{ZJC25}$	In2-4	*dfrA1*	Trimethoprim resistance	21751..22224
			aadA1	Aminoglycoside resistance	22901..23689
	Tn*7*$_{229813}$	In2-4	*dfrA1*	Trimethoprim resistance	21714..22187
			aadA1	Aminoglycoside resistance	22864..23652
	Tn*6800*	In2-77	*lnu*(F)*1b*	Lincosamide resistance	24877..25698
			catB2	Chloramphenicol resistance	25784..26416
			aadA1	Aminoglycoside resistance	27070..27858
	T7RE$_{621164}$	In2-16	*lnu*(F)*1b*	Lincosamide resistance	22955..23800
			dfrA1	Trimethoprim resistance	23797..24270
			aadA1a	Aminoglycoside resistance	24275..25078
			qacED1	Quaternary ammonium compound resistance	25242..25589
			sul1	Sulphonamide resistance	25583..26422
		31.1-kb MDR region	*mph*(E)	Macrolide resistance	29089..29973
			rmtB	Aminoglycoside resistance	32879..33634
			qnrVC1	Quniolone resistace	37668..38324

			sul1	Sulphonamide resistance	42575..43414
			bla$_{CTX-M-3}$	β-lactam resistance	44024..44899
			bla$_{TEM-1}$	β-lactam resistance	45681..46541
			arr-3	Rifampicin resistance	50754..51206
			catB3	Chloramphenicol resistance	51291..51923
			bla$_{OXA-1}$	β-lactam resistance	52061..52891
			aacA4	Aminoglycoside resistance	53022..53540
Tn*1696*-related elements	Tn*1696*	In4	*aacC1*	Aminoglycoside resistance	4987..5520
			aadA2	Aminoglycoside resistance	5901..6680
			cmlA1	Chloramphenicol resistance	6942..8201
			qacED1	Quaternary ammonium compound resistance	8393..8740
			sul1	Dihydropteroate synthase	8734..9573
	Tn*6913a*	38.9-kb T2670RE$_{ZJG812}$	*aadB*	Aminoglycoside resistance	1921928..1922461
			catB5	Chloramphenicol resistance	1922542..1923174
			bla$_{OXA-10}$	β-lactam resistance	1923243..1924043
			aadA1a	Aminoglycoside resistance	1924060..1924851
			qacED1	Quaternary ammonium compound resistance	1925015..1925362
			sul1	Sulphonamide resistance	1925356..1926195
			qnrVC1	Quniolone resistace	1930446..1931102
			qacED1	Quaternary ammonium compound resistance	1935553..1935900
			sul1	Sulphonamide resistance	1935894..1936733
			tetA(B)	Tetracycline resistance	1951297..1952502
			catA1	Chloramphenicol resistance	1954566..1955225
	Tn*6913b*	38.5-kb T2670RE$_{ZJG944}$	*aadB*	Aminoglycoside resistance	2076103..2076636
			catB5	Chloramphenicol resistance	2076717..2077349
			bla$_{OXA-10}$	β-lactam resistance	2077418..2078218
			aadA1a	Aminoglycoside resistance	2078235..2079026
			qacED1	Quaternary ammonium compound resistance	2079190..2079537
			sul1	Sulphonamide resistance	2079531..2080370

			qnrVC1	Quniolone resistace	2084621..2085277
			qacED1	Quaternary ammonium compound resistance	2089728..2090075
			sul1	Sulphonamide resistance	2090069..2090908
			tetA(B)	Tetracycline resistance	2105073..2106278
			catA1	Chloramphenicol resistance	2108342..2109001
	Tn*6914*	In1396	*aadA13*	Aminoglycoside resistance	1973577..1974374
			qacED1	Quaternary ammonium compound resistance	2089728..2090075
			sul1	Sulphonamide resistance	1975192..1976031
	Tn*6915*	In1396	*aadA13*	Aminoglycoside resistance	2006697..2007494
			qacED1	Quaternary ammonium compound resistance	2007971..2008318
			sul1	Sulphonamide resistance	2008312..2009151
		37.9-kb MDR region	*sul3*	Sulphonamide resistance	2012257..2013048
			qacH2	Quaternary ammonium compound resistance	2014228..2014560
			aadA1a	Aminoglycoside resistance	2014730..2015521
			cmlA1a	Chloramphenicol resistance	2015614..2016873
			aadA2	Aminoglycoside resistance	2017135..2017914
			floR	Chloramphenicol resistance	2028128..2029342
			sul2	Sulphonamide resistance	2031845..2032660
			aph(4)-Ia	Aminoglycoside resistance	2038021..2039046
			aacA4cr	Aminoglycoside resistance/Quniolone resistace	2040968..2041567
			bla$_{OXA-1}$	β-lactam resistance	2041653..2042528
			catB3	Chloramphenicol resistance	2042666..2043298
			arr-3	Rifampicin resistance	2043383..2043835
			qacED1	Quaternary ammonium compound resistance	2044058..2044405
			sul1	Sulphonamide resistance	2044399..2045238
	T1696RE$_{ZJD581}$	39.6-kb MDR region	*qacED1*	Quaternary ammonium compound resistance	2056949..2057296
			arr-3	Rifampicin resistance	2057519..2057971
			catB3	Chloramphenicol resistance	2058056..2058688

			bla$_{\text{OXA-1}}$	β-lactam resistance	2058826..2059656
			aacA4cr	Aminoglycoside resistance/Quniolone resistace	2059787..2060386
			aacC4	Aminoglycoside resistance	2062301..2063077
			aph(4)-Ia	Aminoglycoside resistance	2063306..2064331
			sul2	Sulphonamide resistance	2069178..2069993
			floR	Chloramphenicol resistance	2072496..2073710
			dfrA12	Trimethoprim resistance	2082286..2082783
			aadA2	Aminoglycoside resistance	2083203..2083982
			aphA1	Aminoglycoside resistance	2087731..2088558
		In1785	*aphA6*	Aminoglycoside resistance	2095123..2095902
			sul1	Sulphonamide resistance	2098667..2099506
			qacED1	Quaternary ammonium compound resistance	2099500..2099847
			aadA1dx	Aminoglycoside resistance	2100011..2100826
			bla$_{\text{OXA-10}}$	β-lactam resistance	2100819..2101619
			catB5f	Chloramphenicol resistance	2101688..2102320
			aadB	Aminoglycoside resistance	2102401..2102934
		Tn*6260*:IS*Pmi3*	*lnu*(G)	Lincosamide resistance	2106291..2107094
Tn*6963*-related transposable prophage	Tn*6964*	Tn*6965*	*dfrA12*	Trimethoprim resistance	2308673..2309170
			aadA2	Aminoglycoside resistance	2309590..2310369
			aphA1	Aminoglycoside resistance	2314278..2315093
			aacC4	Aminoglycoside resistance	2316101..2316877
			aph(4)-Ia	Aminoglycoside resistance	2317106..2318131
			sul2	Sulphonamide resistance	2322978..2323793
			floR	Chloramphenicol resistance	2326296..2327510
			aacA4cr 12	Aminoglycoside resistance/Quniolone resistace	2336186..2336785
			bla$_{\text{OXA-1}}$	β-lactam resistance	2336871..2337746
			catB3	Chloramphenicol resistance	2337884..2338516
			dfrA27	Trimethoprim resistance	2339186..2339659
			aadA16	Aminoglycoside resistance	2339840..2340685

			qacED1	Quaternary ammonium compound resistance	2340802..2341149
			sul1	Sulphonamide resistance	2341143..2341982
			tetA(B)	Tetracycline resistance	2346062..2347267
Tn6872-related IME	Tn*6966*	Tn*6971*	*tetA(B)*	Tetracycline resistance	3385515..3386720
			sul1	Sulphonamide resistance	3390800..3391639
			qacED1	Quaternary ammonium compound resistance	3391633..3391980
			aadA16	Aminoglycoside resistance	3392097..3392942
			dfrA27	Trimethoprim resistance	3393123..3393596
			arr-3	Rifampicin resistance	3393729..3394181
			catB3	Chloramphenicol resistance	3394266..3394898
			bla$_{OXA-1}$	β-lactam resistance	3395036..3395866
			aacA4cr	Aminoglycoside resistance/Quniolone resistace	3395997..3396596
		23.2-kb MDR region	*floR*	Chloramphenicol resistance	3405274..3406488
			sul2	Sulphonamide resistance	3408991..3409806
			aph(4)-Ia	Aminoglycoside resistance	3414653..3415678
			aacC4	Aminoglycoside resistance	3415907..3416683
			aphA1	Aminoglycoside resistance	3418506..3417691
			aadA2	Aminoglycoside resistance	3422415..3423194
			dfrA12	Trimethoprim resistance	3423614..3424111
Tn*6397*-family ICEs	Tn*6397*	63.7-kb LAM	*bla*$_{IMP-8}$	β-lactam resistance	57286..58026
			aacA4'-3	Aminoglycoside resistance	58125..58679
			macB	Macrolide resistance	66171..67376
	Tn*6967*	48.5-kb LAM	*macB*	Macrolide resistance	682063..687709
			floR	Chloramphenicol resistance	694575..695789
			sul2	Sulphonamide resistance	698292..699107
			aph(4)-Ia	Aminoglycoside resistance	703954..704979
			aacC4	Aminoglycoside resistance	705208..705984
			aacA4cr	Aminoglycoside resistance/Quniolone resistace	706899..707498
			bla$_{OXA-1}$	β-lactam resistance	707584..708459

			catB3	Chloramphenicol resistance	708597..709229
			arr-3	Rifampicin resistance	709314..709766
			qacED1	Quaternary ammonium compound resistance	709989..710336
			sul1	Sulphonamide resistance	710330..711168
Tn*2670*-related elements	Tn*2670*	Tn*9*-like backbone	*catA1*	Chloramphenicol resistance	992..1651
		Tn*21*	*aadA1a*	Aminoglycoside resistance	7678.. 8457
			qacED1	Quaternary ammonium compound resistance	8621..8968
			sul1	Sulphonamide resistance	8935..9801
	$T2670RE_{11759}$	Type A In37-like element	*aacA4cr*	Aminoglycoside resistance/Quniolone resistace	2290371..2290970
			bla$_{OXA-1}$	β-lactam resistance	2291101..2291931
			catB3	Chloramphenicol resistance	2292069..2292701
		Truncated type A IS*26*–*fosA3*–*IS26* unit	*fosA3*	Fosfomycin resistance	2294175..2294591
		Interrupted Tn*6029*	*strB*	Aminoglycoside resistance	2296214..2297044
			strA	Aminoglycoside resistance	2297050..2297853
			sul2	Sulphonamide resistance	2297914..2298729
		Tn*4352*	*aphA1*	Aminoglycoside resistance	2301499..2302314
		Interrupted Tn*6029*	*bla*$_{TEM-1}$	β-lactam resistance	2303792..2304652
		Truncated *aacC2*–*tmrB* region	*tmrB*	Tunicamycin resistance	2306350..2306892
			aacC2	Aminoglycoside resistance	2306905..2307765
		IS*26*–*mph*(A)–IS*6100* unit	*mph*(A)	Macrolide resistance	2308698..2309603
		chrA–*orf98* unit	*chrA*	Chromate resistance	2313222..2314427
		In54	*sul1*	Sulphonamide resistance	2314914..2315753
			qacED1	Quaternary ammonium compound resistance	2315747..2316094
			aadA5	Aminoglycoside resistance	2316300..2317088
			dfrA17	Trimethoprim resistance	2317219..2317692
		Tn*9*-like backbone	*catA1*	Chloramphenicol resistance	2323716..2324375

Table S6. Major features of 11 plasmids identified in this study

Designation	Isolate	Accession number	Assembled bases (bp)	Inc group	ARGs	AGEs
p11759-FII	11759	MZ848139	38,408	IncFII		
p11759-1		MZ848136	33,731	IncX1		
p11759-2		MZ848137	2,683	Col3M	*qnrD1*	
p11759-3		MZ848138	8,439	Col156	*bla*$_{CTX-M-55}$	
p516602-FII	516602	MN310367	52,180	IncFII	*bla*$_{KPC-2}$ and *bla*$_{TEM-1B}$	ΔTn*6296* and ΔTn*2*
p229813-FII	229813	MN310368	50,842	IncFII	*bla*$_{KPC-2}$ and *bla*$_{TEM-1B}$	ΔTn*6296* and ΔTn*2*
p621164-FII	621164	MT701512	54,395	IncFII		
pGN28-KPC	GN28	MF156712	46,193	IncX6	*bla*$_{KPC-2}$ and *bla*$_{TEM-1A}$	ΔTn*6296*
p81703-FII	81703	MT679665	73,394	IncFII		
p81703-2		MZ848141	8,282	unclassified		
p12304-1	12304	MZ848140	2,671	Col3M	*qnrD1*	

Table S7. Percentage of the 692 *Morganella* isolates resistant to 18 antimicrobials in China of 2021[a]

Class	Antimicrobials	%Percent
Penicillins	Ampicillin	97.3
	Ampicillin/sulbactam	59
	Piperacillin	22.4
	Piperacillin/tazobactam	4.2
Cephalosporin, second generation	Cefuroxime	87.3
Cephalosporins, third generation	Cefotaxime	28.5
	Ceftriaxone	14.3
	Ceftazidime	14.9
	Cefoperazone/sulbactam	3.5
Cephalosporin, fourth generation	Cefepime	4.1
Carbapenems	Ertapenem	0.9
	Meropenem	1.4
	Imipenem	23.3
Aminoglycosides	Amikacin	1
	Gentamicin	19.6
Fluoroquinolones	Levofloxacin	10.5
	Ciprofloxacin	21.5
Sulphonamides	Sulfamethoxazole/trimethoprim	39.5

[a]Data are derived from China Antimicrobial Surveillance Network (http://www.chinets.com/)
